# Supplementary material for: Clinical relevance of zebrafish for gene variants testing. Proof-of-principle with SMN1/SMA
Source: EMBO Mol Med. 2025 Dec 15;18(1):41–54. doi: 10.1038/s44321-025-00355-8 (PMC12808650; doi:10.1038/s44321-025-00355-8)
Supplement: Supplementary file 1 — Appendix [file 44321_2025_355_MOESM1_ESM.pdf]

Clinical relevance of zebrafish for gene variants testing. Proof-of-principle with *SMN1*/SMA.

Brett W. Stringer[1], Yougang Zhang[1], Afsaneh Taghipour-Sheshdeh[1], Shuxiang Goh[3], Heike Kölbel[4], Michelle A Farrar[3,5], Brunhilde Wirth[6,7,8], and Jean Giacomotto[1,2,9,10]

Appendix Figure S1. **Page 2**
Appendix Table S1. **Page 3**
Appendix Table S2. **Page 4**
Appendix Figure S2. **Page 5**
Appendix Figure S3. **Page 7**
Appendix Figure S4. **Page 8**
Appendix Figure S5. **Page 9**
Appendix Figure S6. **Page 11**
Appendix Figure S7. **Page 12**
Appendix Figure S8. **Page 13**

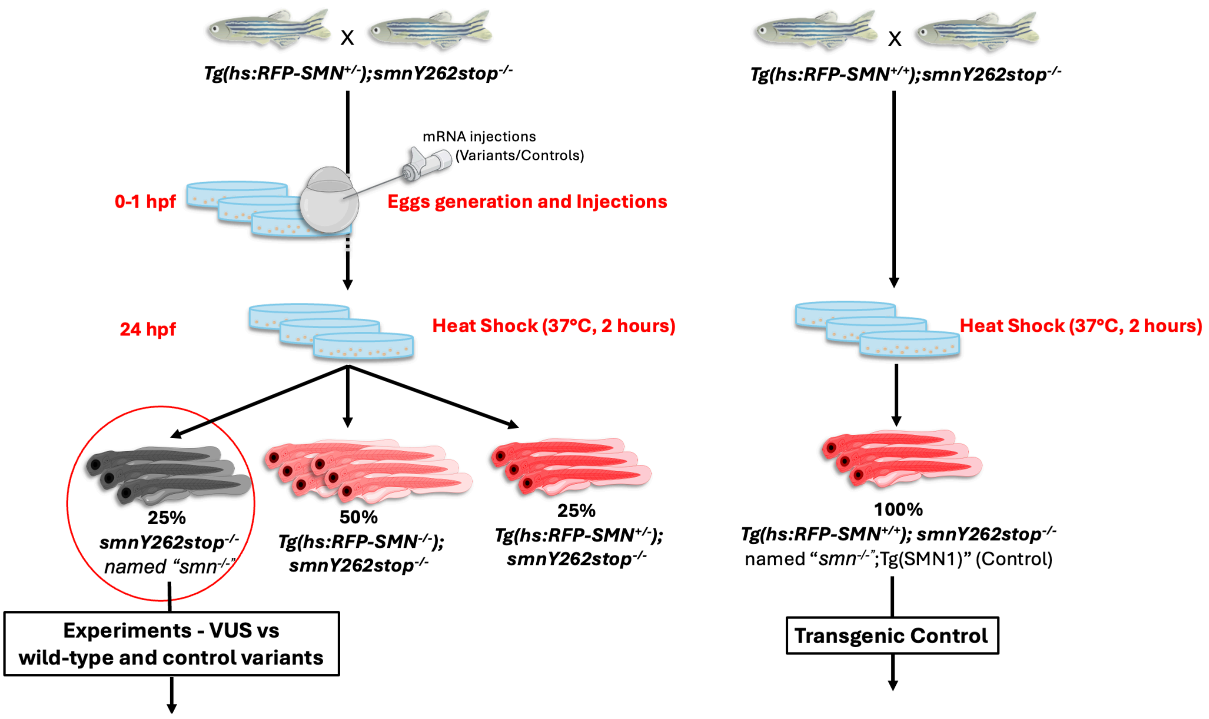

Appendix Figure S1. Methodological framework to produce maternal zygotic *smn1* knockout mutants depleted in SMN protein. Zebrafish embryos/larvae named *smn*$^{-/-}$ in this study are produced from *Tg(hs:RFP-SMN*$^{+/-}$*);smn*$^{Y262stop-/-}$ zebrafish (PMID 19592581 & 23459934). Incrossing this line produces 100% maternal zygotic *smnY262stop*$^{-/-}$ mutants, of which 25% do not inherit the rescue transgene *Tg*(*hs:RFP-SMN*), so do not express any form of SMN protein. To select these 25% maternal zygotic animals *smn*$^{Y262stop-/-}$ (named *smn*$^{-/-}$ in this manuscript) from their transgenic siblings *Tg(hs:RFP-SMN);smn*$^{Y262stop-/-}$, the clutches were heat-shocked at 24 hpf (hours post fertilisation) for 2 hours at 37°C and subsequently identified by the absence of detectable red fluorescence.

Zebrafish clinical value for VUS testing. Appendix/Supplemental file.

Appendix Table S1. Plasmids created for *SMN1* VUS analysis.

Plasmid name	Purpose
249-pME-hsa_SMN1-wt	contains *SMN1* wild-type cDNA
250-pME-c.861_864_SMN1	contains *SMN1* variant cDNA (861VUS)
252-pME-c.855_858_SMN1	contains *SMN1* variant cDNA (855VUS)
253-pME-c.549del_SMN1	contains *SMN1* variant cDNA (path)
254-pME-c.462A>G_SMN1	contains *SMN1* variant cDNA (non-path)
260-pT3-hsa_SMN1-WT_polyA	*in vitro* transcription of *SMN1* wild-type mRNA
261-pT3-c.861_864_hsa_SMN1_polyA	*in vitro* transcription of *SMN1* 861VUS mRNA
263-pT3-c.855_858_hsa_SMN1_polyA	*in vitro* transcription of *SMN1* 855VUS mRNA
264-pT3-c.549del_hsa_SMN1_polyA	*in vitro* transcription of *SMN1* path mRNA
265-T3-c.462A>G_hsa_SMN1_polyA	*in vitro* transcription of *SMN1* non-path mRNA
279-hsa-SMN1c.5C>G(p.Ala2Gly)	contains *SMN1* variant cDNA A2G
280-hsa-SMN1c.5C>T(pAla2Val)	contains *SMN1* variant cDNA A2V
281-hsa-SMN1c.84C>T(p.Ser28=)	contains *SMN1* variant cDNA S28= (non-path 2)
282-hsa-SMN1c.131A>T(p.Asp44Val)	contains *SMN1* variant cDNA D44V
283-hsa-SMN1c.734C>T(p.Pro245Leu)	contains *SMN1* variant cDNA P245L
284-hsa-SMN1c.785G>T(p.Ser262Ile)	contains *SMN1* variant cDNA S262I
285-hsa-SMN1c.821C>T(p.Thr274Ile)	contains *SMN1* variant cDNA T274I
287-pT3-hsa_SMN1_A2G	*in vitro* transcription of *SMN1* variant A2G mRNA
288-pT3-hsa_SMN1_A2V	*in vitro* transcription of *SMN1* variant A2V mRNA
289-pT3-hsa_SMN1_S28=	*in vitro* transcription of *SMN1* variant S28= mRNA
290-pT3-hsa_SMN1_D44V	*in vitro* transcription of *SMN1* variant D44V mRNA
291-pT3-hsa_SMN1_P245L	*in vitro* transcription of *SMN1* variant P245L mRNA
292-pT3-hsa_SMN1_S262I	*in vitro* transcription of *SMN1* variant S262I mRNA
293-pT3-hsa_SMN1_T274I	*in vitro* transcription of *SMN1* variant T274I mRNA
298-pT3-hsa_SMN1:c.43C>T (p.Gln15Ter)	*in vitro* transcription of SMN1 variant Q15* mRNA (path 2)

Appendix Table S2. Q-PCR primers for measuring *hsa-SMN1* mRNA expression.

Primer name	Reference	Sequence
310-dre_eef1a-qF1	PMID: 32728128	5’-GGGCAAGGGCTCCTTCAA-3’
311-dre_eef1a-qR1	PMID: 32728128	5’-CGCTCGGCCTTCAGTTTG-3’
312-hsa_SMN1-qF1	This study	5’-TCTTTTCTCCCTCCACCACC-3’
313-hsa_SMN1-qR1	This study	5’-CAGAAGGAAATGGAGGCAGC-3’

Replicate 1

A

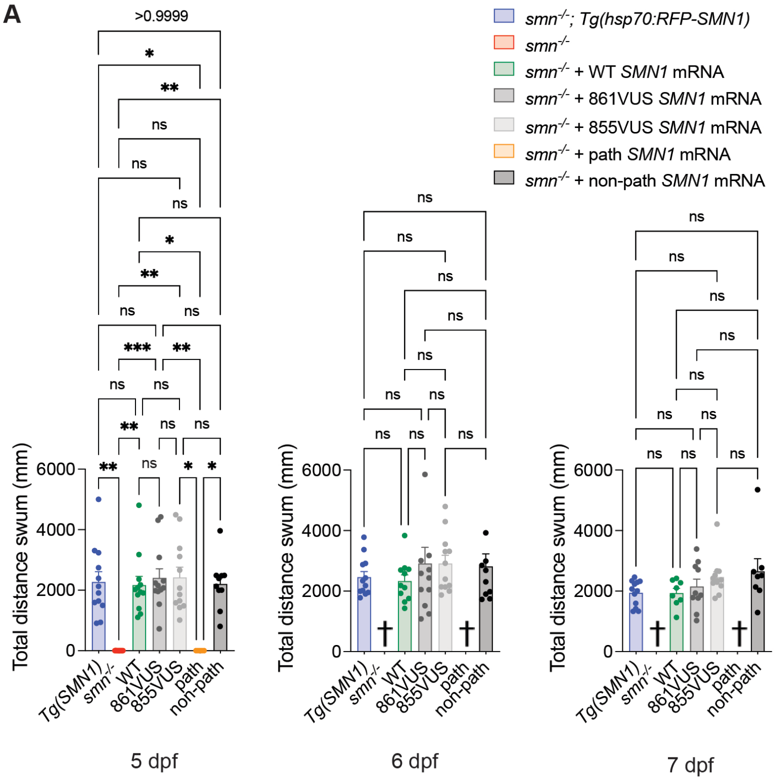

B

Dunn's multiple comparisons test	Summary 5dpf	Adjusted P-Value
WT vs. 861VUS	ns	>0.9999
WT vs. 855VUS	ns	>0.9999
WT vs. path	*	0.0455
WT vs. non-path	ns	>0.9999
WT vs. smn-/-	**	0.0056
WT vs. Tg(SMN1)	ns	>0.9999
861VUS vs. 855VUS	ns	>0.9999
861VUS vs. path	**	0.0094
861VUS vs. non-path	ns	>0.9999
861VUS vs. smn-/-	***	0.0006
861VUS vs. Tg(SMN1)	ns	>0.9999
855VUS vs. path	*	0.0172
855VUS vs. non-path	ns	>0.9999
855VUS vs. smn-/-	**	0.0014
855VUS vs. Tg(SMN1)	ns	>0.9999
path vs. non-path	*	0.0159
path vs. smn-/-	ns	>0.9999
path vs. Tg(SMN1)	*	0.0235
non-path vs. smn-/-	**	0.0015
non-path vs. Tg(SMN1)	ns	>0.9999
smn-/- vs. Tg(SMN1)	**	0.0021

C

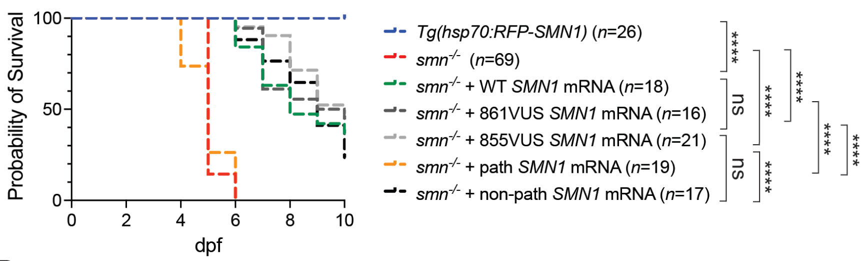

D

Variant		SMA type	Median survival (dpf)	vs $smn^{-/-}$	vs WT
Tg(hsp70:RFP-SMN1)	*Tg(SMN1)*		Undefined		
smn[-]	*smn*[-]		5		*P*<0.0001
SMN1 WT	WT		8	*P*<0.0001	
SMN1 c.861-864del (p.Arg288Alafs*5)	861VUS	VUS	9.5	*P*<0.0001	*P*=0.5973
SMN1 c.855-858del (p.Arg288Alafs*5)	855VUS	VUS	10	*P*<0.0001	*P*=0.2618
SMN1 c.549del (p.Lys184fs)	path	I	5	*P*=0.3638	*P*<0.0001
SMN1 c.462A>G (p.Gln154=)	non-path	benign	9	*P*<0.0001	*P*=0.0523

Appendix Figure S2. 861VUS and 855VUS mRNA injections rescue SMN-deficient *smn*$^{-/-}$ zebrafish movement and survival (Details/Statistics for Figure 3, Replicate 1). First of three replicate experiments assessing the ability of injected *SMN1* mRNAs to rescue the swimming ability and survival of SMN-deficient *smn*$^{-/-}$ fish. **(A)** SMN-deficient *smn*$^{-/-}$ zebrafish present dramatic motor function loss at 5 dpf and die by 6 dpf. Human wt-*SMN1* transgenic ubiquitous expression (*Tg(SMN1)*) or injected wt-*SMN1* mRNA (WT) rescue this motor function loss. The non-pathogenic *SMN1* variant (non-path) and both 861VUS and

855VUS efficiently restore motor function with no detectable significant difference. In contrast, mRNA from the pathogenic *SMN1* variant (path) failed to improve motor function with no difference from negative control (*smn*$^{-/-}$). Graphs represent comparisons of the total distance swum by each cohort of fish over 24 minutes at 5, 6 and 7 dpf. Each data point represents one fish. Cross symbols indicate 100% mortality. Error bars represent standard error of the mean. Statistical significance was evaluated using the Kruskal-Wallace test with Dunn's correction for multiple comparisons. ***, $P<0.001$; **, $P<0.01$; *, $P<0.05$; ns, not significant. Source data are available online for this Figure. **(B)** Actual *P*-values for the comparisons shown in (A). **(C)** SMN-deficient *smn*$^{-/-}$ zebrafish (*smn*$^{-/-}$, red dashed line) had a median survival of 5 days. Injected mRNA from the known pathogenic *SMN1* variant (path, orange dashed line) did not have any effect on survival. In contrast, demonstrating restoration of Smn/SMN function, injected mRNA from 861VUS (dark grey dashed line) and 855VUS (light grey dashed line) extended survival of *smn*$^{-/-}$ larvae similar to wt-mRNA (WT, green dashed line) and non-pathogenic variant (non-path, black dashed line) animals. ****, $P<0.0001$; ns, not significant. Statistical significance was evaluated using the log-rank (Mantel-Cox) test. **(D)** Exact *P*-values and median survival data for the analysis shown in (C). Source data are available online for this Figure.

Replicate 2

A

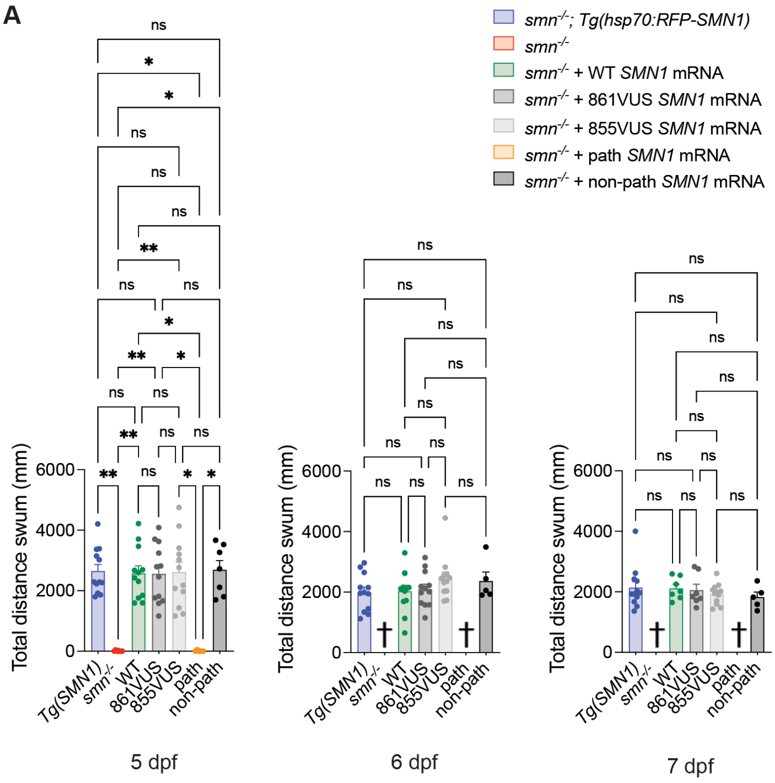

B

Dunn's multiple comparisons test	Summary 5 dpf	Adjusted *P*-Value
WT vs. 861VUS	ns	>0.9999
WT vs. 855VUS	ns	>0.9999
WT vs. path	*	0.0348
WT vs. non-path	ns	>0.9999
WT vs. smn-/-	**	0.0068
WT vs. Tg(SMN1)	ns	>0.9999
861VUS vs. 855VUS	ns	>0.9999
861VUS vs. path	**	0.0421
861VUS vs. non-path	ns	>0.9999
861VUS vs. smn-/-	***	0.0087
861VUS vs. Tg(SMN1)	ns	>0.9999
855VUS vs. path	*	0.0378
855VUS vs. non-path	ns	>0.9999
855VUS vs. smn-/-	**	0.0076
855VUS vs. Tg(SMN1)	ns	>0.9999
path vs. non-path	*	0.0455
path vs. smn-/-	ns	>0.9999
path vs. Tg(SMN1)	*	0.0193
non-path vs. smn-/-	**	0.0129
non-path vs. Tg(SMN1)	ns	>0.9999
smn-/- vs. Tg(SMN1)	**	0.0032

C

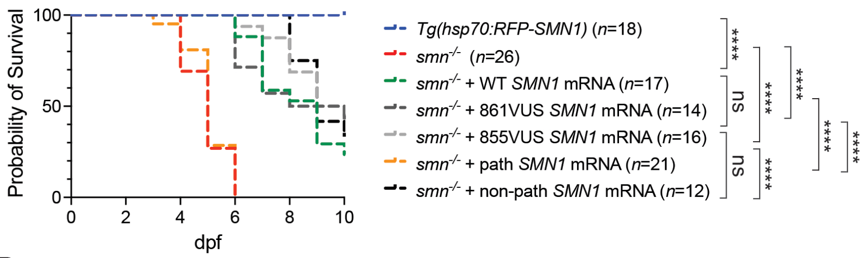

D

Variant		SMA type	Median survival (dpf)	vs *smn*$^{-/-}$	vs WT
Tg(hsp70:RFP-SMN1)	*Tg(SMN1)*		Undefined		
smn$^{-/-}$	*smn*$^{-/-}$		5		*P*<0.0001
SMN1 WT	WT		8	*P*<0.0001	
SMN1 c.861-864del (p.Arg288Alafs*5)	861VUS	VUS	9.5	*P*<0.0001	*P*=0.5207
SMN1 c.855-858del (p.Arg288Alafs*5)	855VUS	VUS	10	*P*<0.0001	*P*=0.1719
SMN1 c.549del (p.Lys184fs)	path	I	5	*P*=0.6645	*P*<0.0001
SMN1 c.462A>G (p.Gln154=)	non-path	benign	9	*P*<0.0001	*P*=0.2635

Appendix Figure S3. 861VUS and 855VUS mRNA injections rescue SMN-deficient *smn*$^{-/-}$ zebrafish movement and survival (related to Figure 3, replicate 2). Second of three replicate experiments assessing the ability of injected *SMN1* mRNAs to rescue the swimming ability and survival of SMN-deficient *smn*$^{-/-}$ fish. **(A)**, **(B)**, **(C)** and **(D)** are as in **Appendix Figure S2.**

Replicate 3

A

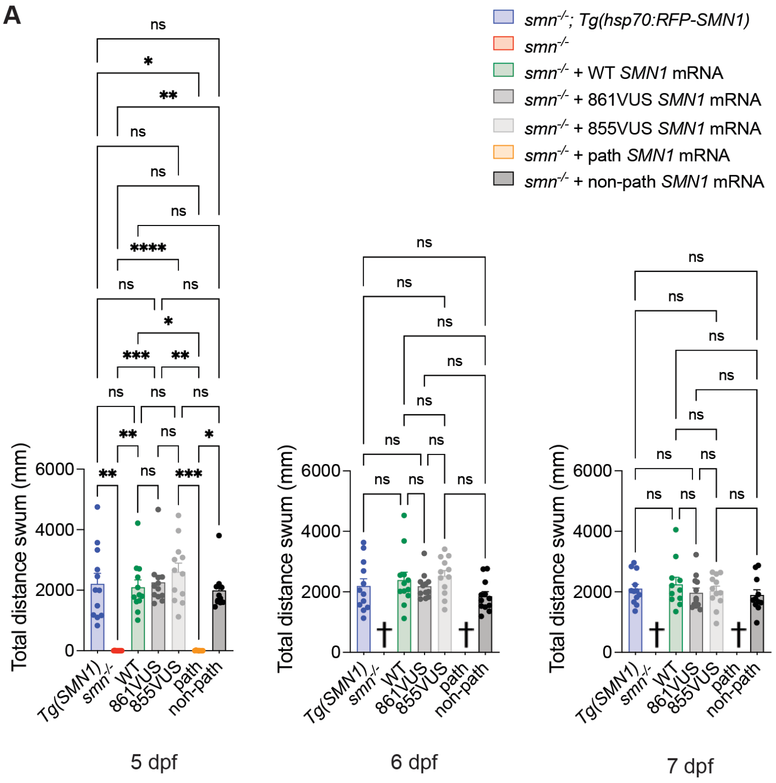

B

Dunn's multiple comparisons test	Summary 5dpf	Adjusted *P*-Value
WT vs. 861VUS	ns	>0.9999
WT vs. 855VUS	ns	>0.9999
WT vs. path	*	0.0193
WT vs. non-path	ns	>0.9999
WT vs. smn-/-	**	0.0034
WT vs. Tg(SMN1)	ns	>0.9999
861VUS vs. 855VUS	ns	>0.9999
861VUS vs. path	**	0.0042
861VUS vs. non-path	ns	>0.9999
861VUS vs. smn-/-	***	0.0005
861VUS vs. Tg(SMN1)	ns	>0.9999
855VUS vs. path	*	0.0006
855VUS vs. non-path	ns	>0.9999
855VUS vs. smn-/-	**	<0.0001
855VUS vs. Tg(SMN1)	ns	>0.9999
path vs. non-path	*	0.0452
path vs. smn-/-	ns	>0.9999
path vs. Tg(SMN1)	*	0.0187
non-path vs. smn-/-	**	0.0096
non-path vs. Tg(SMN1)	ns	>0.9999
smn-/- vs. Tg(SMN1)	**	0.0033

C

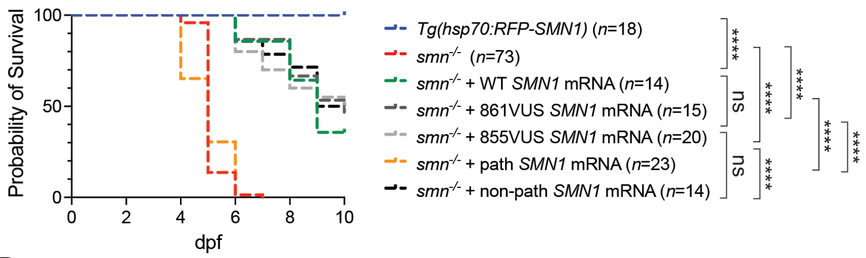

D

Variant		SMA type	Median survival (dpf)	vs *smn*$^{-/-}$	vs WT
Tg(hsp70:RFP-SMN1)	*Tg(SMN1)*		Undefined		
smn$^{-/-}$	*smn*$^{-/-}$		5		*P*<0.0001
SMN1 WT	WT		8	*P*<0.0001	
SMN1 c.861-864del (p.Arg288Alafs*5)	861VUS	VUS	9.5	*P*<0.0001	*P*=0.6015
SMN1 c.855-858del (p.Arg288Alafs*5)	855VUS	VUS	10	*P*<0.0001	*P*=0.6470
SMN1 c.549del (p.Lys184fs)	path	I	5	*P*=0.3765	*P*<0.0001
SMN1 c.462A>G (p.Gln154=)	non-path	benign	9	*P*<0.0001	*P*=0.5373

Appendix Figure S4. 861VUS and 855VUS mRNA injections rescue SMN-deficient *smn*$^{-/-}$ zebrafish movement and survival (related to Figure 3, replicate 3). Third of three replicate experiments assessing the ability of injected *SMN1* mRNAs to rescue the swimming ability and survival of SMN-deficient *smn*$^{-/-}$ fish. **(A)**, **(B)**, **(C)** and **(D)** are as in **Appendix Figure S2.**

A

	Variant		SMA type	*SMN2*	Median survival (dpf)	vs *smn*-/-	vs WT
1	*SMN1* c.861-864del (p.Arg288Alafs*5)	861VUS			8	**** *P*<0.0001	*ns*, *P*=0.5792
2	*SMN1* c.855-858del (p.Arg288Alafs*5)	855VUS			9	**** *P*<0.0001	*ns*, *P*=0.7204
3	*SMN1* c.549del (p.Lys184fs)	path 1	I	*n*=1	5	*ns*, *P*=0.1265	**** *P*<0.0001
4	*SMN1* c.43C>T (p.Gln15*)	path 2	I	*n*=1	5	*ns*, *P*=0.1823	**** *P*<0.0001
5	*SMN1* c.462A>G (p.Gln154=)	non-path 1	benign	*n*=1	8	**** *P*<0.0001	*ns*, *P*=0.1575
6	*SMN1* c.84C>T (p.Ser28=)	non-path 2	benign	*n*=1	8	**** *P*<0.0001	*ns*, *P*=0.7600
7	*SMN1* c.5C>G (p.Ala2Gly)	A2G	II	*n*=1	6	**** *P*=0.0001	**** *P*<0.0001
8	*SMN1* c.5C>T (p.Ala2Val)	A2V	III	*n*=1	5	**** *P*<0.0001	**** *P*=0.0001
9	*SMN1* c.131A>T (p.Asp44Val)	D44V	III	*n*=1	6	**** *P*<0.0001	**** *P*<0.0001
10	*SMN1* c.734C>T (p.Pro245Leu)	P245L	III	*n*=1	5	**** *P*<0.0001	**** *P*<0.0001
11	*SMN1* c.785G>T (p.Ser262Ile)	S262I	III	*n*=1	6	**** *P*<0.0001	**** *P*<0.0001
12	*SMN1* c.821C>T (p.Thr274Ile)	T274I	II	*n*=1	6	**** *P*<0.0001	**** *P*<0.0001

B

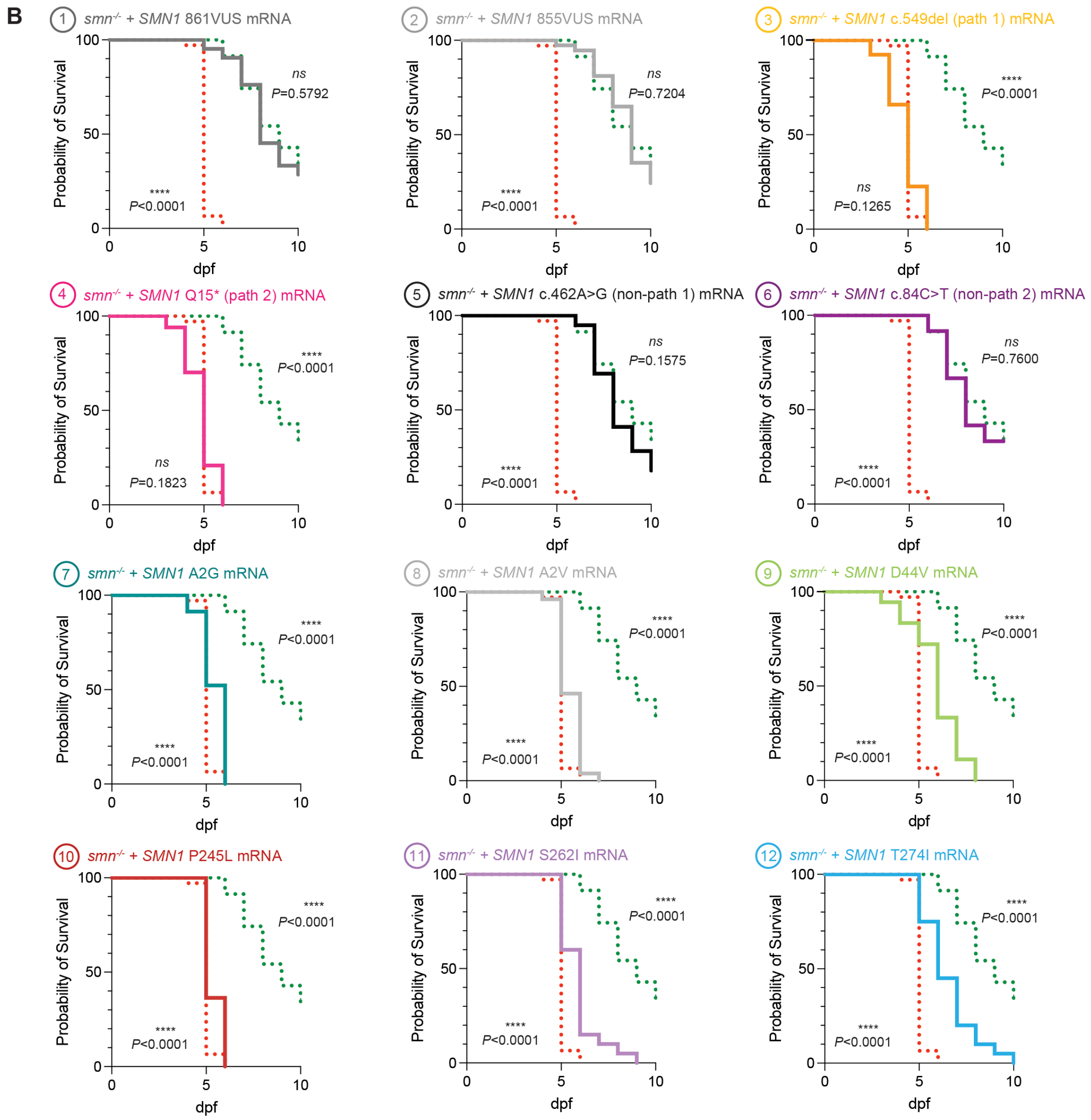

Appendix Figure S5. mRNA injections of *SMN1* hypomorphic variants extend the survival of SMN-deficient zebrafish, but only partially compared with wild-type or non-pathogenic controls (Related to Figure 5, second of three replicate experiments). (A) Median survival of *smn*$^{-/-}$ larvae injected with each of the *SMN1* variants associated with SMA type II or III, was compared with both VUS and with known pathogenic and non-pathogenic

SMN1 variants. Kaplan-Meier analysis was used to determine median survival. Statistical significance, compared to *smn*$^{-/-}$ mock-injected control fish (smn$^{-/-}$) and wt-*SMN1* mRNA injected animals (WT), was determined by log-rank (Mantel-Cox) test. ****, P<0.0001; ns, not significant. Exact P-values are also shown. **(B)** Survival curves for each of the tested *SMN1* variants (solid coloured line) shown relative to those for *smn*$^{-/-}$ mock-injected control fish (dotted red line) and wt-*SMN1* mRNA-injected animals (dotted green line). P-values are as in (A). All tested missense variants, associated with mild forms of the disease, significantly extended the survival of SMN-deficient *smn*$^{-/-}$ animals, though none matched the rescue efficiency of wt-*SMN1* mRNA, suggesting that the presented methodology would also be useful to detect hypomorphs. These findings, combined with the data presented in Figure 3 and 4, support that the tested 861VUS and 855VUS variants are neither pathogenic nor hypomorphic.

Zebrafish clinical value for VUS testing. Appendix/Supplemental file.

A

	Variant		SMA type	*SMN2*	Median survival (dpf)	vs *smn*-/-	vs WT
1	*SMN1* c.861-864del (p.Arg288Alafs*5)	861VUS			8	**** *P*<0.0001	*ns*, *P*=0.6115
2	*SMN1* c.855-858del (p.Arg288Alafs*5)	855VUS			9.5	**** *P*<0.0001	*ns*, *P*=0.2643
3	*SMN1* c.549del (p.Lys184fs)	path 1	I	*n*=1	5	*ns*, *P*=0.5751	**** *P*<0.0001
4	*SMN1* c.43C>T (p.Gln15*)	path 2	I	*n*=1	5.5	*ns*, *P*=0.0646	**** *P*<0.0001
5	*SMN1* c.462A>G (p.Gln154=)	non-path 1	benign	*n*=1	9	**** *P*<0.0001	*ns*, *P*=0.3895
6	*SMN1* c.84C>T (p.Ser28=)	non-path 2	benign	*n*=1	8	**** *P*<0.0001	*ns*, *P*=0.8153
7	*SMN1* c.5C>G (p.Ala2Gly)	A2G	II	*n*=1	6	**** *P*<0.0001	**** *P*<0.0001
8	*SMN1* c.5C>T (p.Ala2Val)	A2V	III	*n*=1	6.5	**** *P*<0.0001	*** *P*=0.0002
9	*SMN1* c.131A>T (p.Asp44Val)	D44V	III	*n*=1	6	**** *P*<0.0001	*** *P*=0.0008
10	*SMN1* c.734C>T (p.Pro245Leu)	P245L	III	*n*=1	6	**** *P*<0.0001	**** *P*<0.0001
11	*SMN1* c.785G>T (p.Ser262Ile)	S262I	III	*n*=1	7	**** *P*<0.0001	*** *P*=0.0005
12	*SMN1* c.821C>T (p.Thr274Ile)	T274I	II	*n*=1	7	**** *P*<0.0001	** *P*=0.0034

B

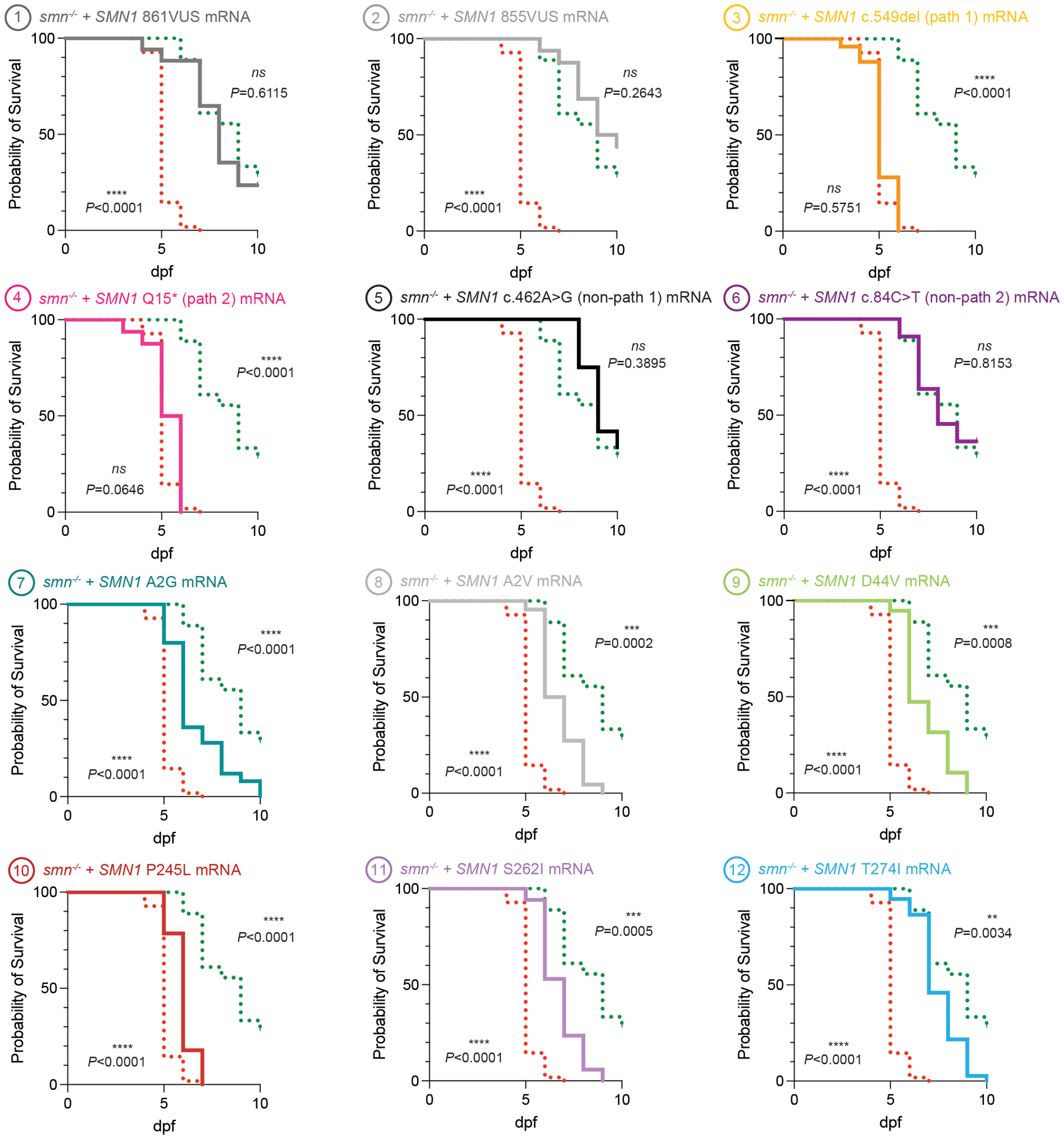

Appendix Figure S6. mRNA injections of *SMN1* hypomorphic variants extend the survival of SMN-deficient zebrafish, but only partially compared with wild-type or non-pathogenic controls (Related to Figure 5, third of three replicate experiments). (A) and **(B)**, as in Appendix Figure S5.

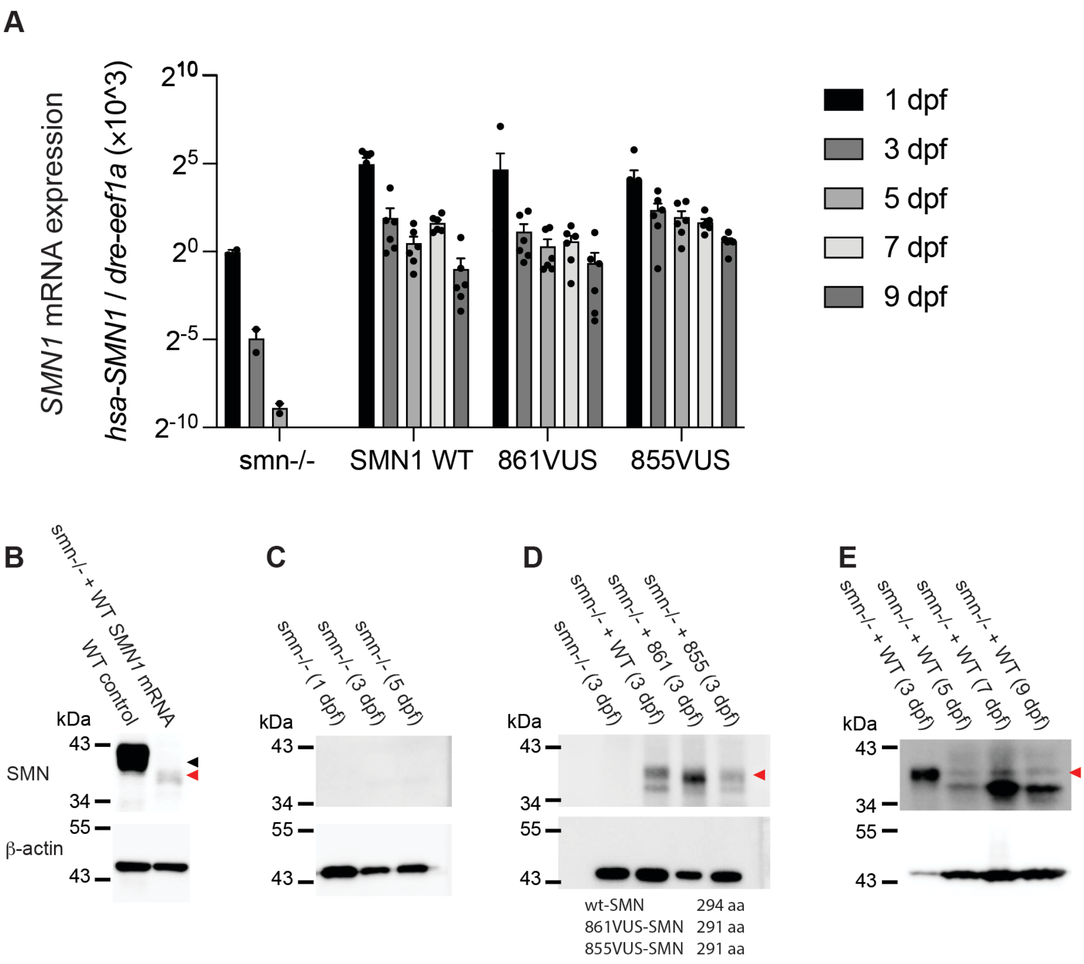

Appendix Figure S7. RT-qPCR and western blot analysis following injections of wt, 861VUS and 855VUS mRNAs in zebrafish. A, RT-qPCR analysis showing *SMN1* mRNA expression relative to endogenous dre-eef1a mRNA. Expression from wt, 861VUS, and 855VUS mRNAs shows a sharp decline between 1 and 3 dpf but remains detectable at low levels up to 9 dpf. Low levels of human *SMN1* mRNA are also detected in uninjected $smn^{-/-}$ control animals (without associated detectable protein on western blots; see panels C and Hao *et al.,* 2013, Fig. 3). This is likely due to minor maternal deposition of transgenic human *SMN1* mRNA from the incross of maternal zygotic *Tg(hs:RFP-SMN);*$smn^{Y262stop-/-}$. *n*=2 fish for $smn^{-/-}$, 1 fish per time point from 2 biological replicates; *n*=6 fish for *SMN1* WT and *SMN1* 861VUS, 3 fish per time point from 2 biological replicates. **B,** Western blot analysis of wild-type control and maternal zygotic $smn^{-/-}$ animals complemented with 250 pg of human *SMN1* mRNA at 3 dpf. Black arrowhead, Zebrafish SMN. Red arrowhead, human SMN from mRNA injections. Human SMN has a predicted molecular weight of 32 kDa with an apparent weight of 38 kDa on the gels (https://dshb.biology.uiowa.edu/MANSMA12-2E6). **C,** Western blot showing absence of detectable SMN protein in maternal zygotic $smn^{-/-}$ controls across all analysed time points. **D,** Western blot comparing $smn^{-/-}$ control and $smn^{-/-}$ animals injected with 250 pg of wt, 861VUS or 855VUS at 3 dpf, showing detectable expression of the respective SMN proteins. The shorter length of 861VUS and 855VUS proteins results in a slight shift in migration. **E,** Temporal analysis of SMN protein levels after 250 pg mRNA injections. SMN levels decrease sharply after 3 dpf but remain detectable at low levels until at least 9 dpf. Protein loading was 15 µg per lane, except for wt-mRNA at 5, 7, and 9 dpf, where 100 µg was loaded per lane. Protein extractions were performed using 40 embryos at 1 dpf, 10 larvae at 3 dpf, and 20 larvae at 5, 7 and 9 dpf.

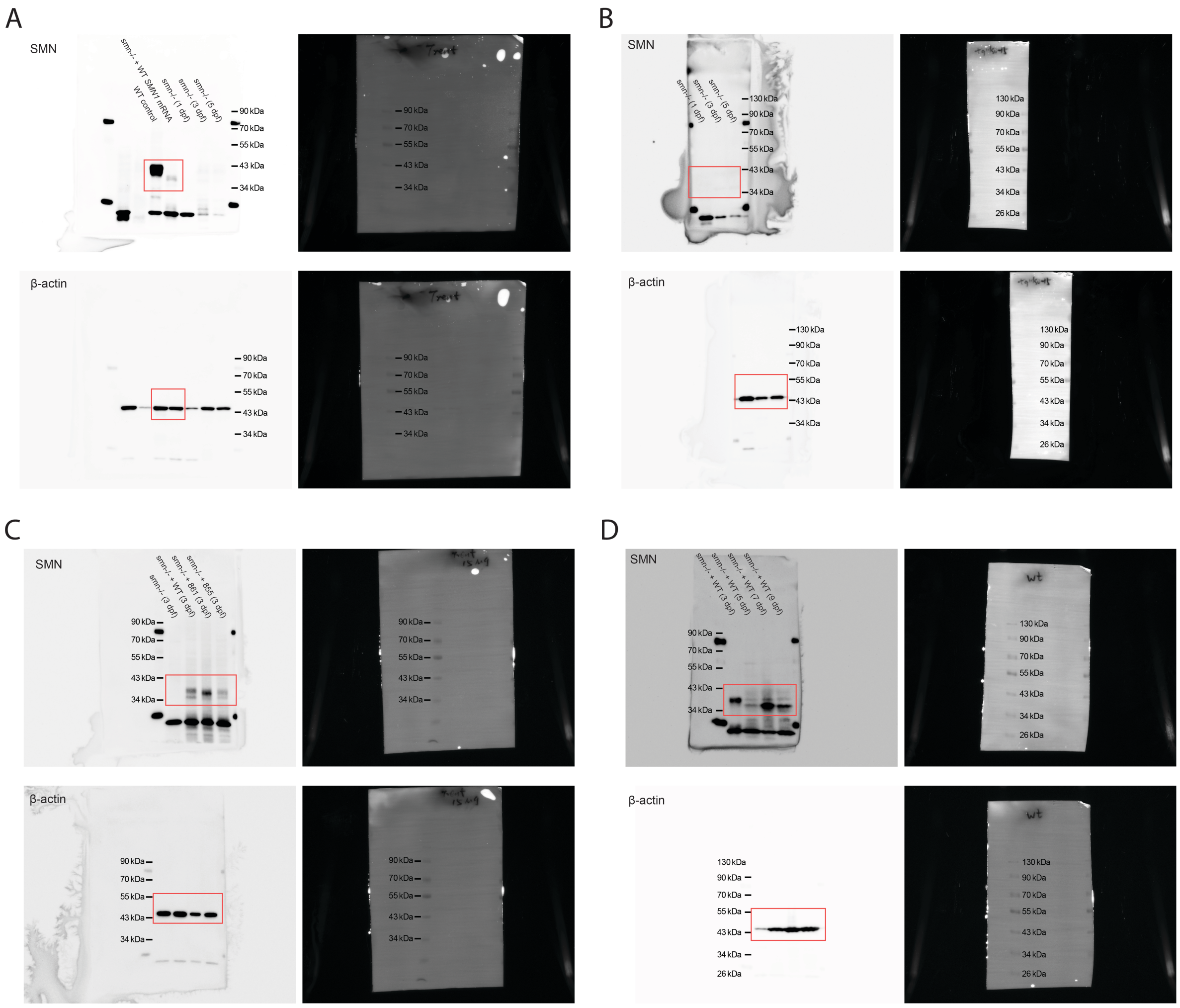

Appendix Figure S8. Native western blot snapshots associated with Appendix Figure S2. A, blots associated with Appendix Figure S2B. **B,** blots associated with Appendix Figure S2C. **C,** blots associated with Appendix Figure S2D. **D,** blots associated with Appendix Figure S2E.
