# Supplementary figures and images for: Clinical relevance of zebrafish for gene variants testing. Proof-of-principle with SMN1/SMA (part 1 of 2)
Source: EMBO Mol Med. 2025 Dec 15;18(1):41–54. doi: 10.1038/s44321-025-00355-8 (PMC12808650; doi:10.1038/s44321-025-00355-8)

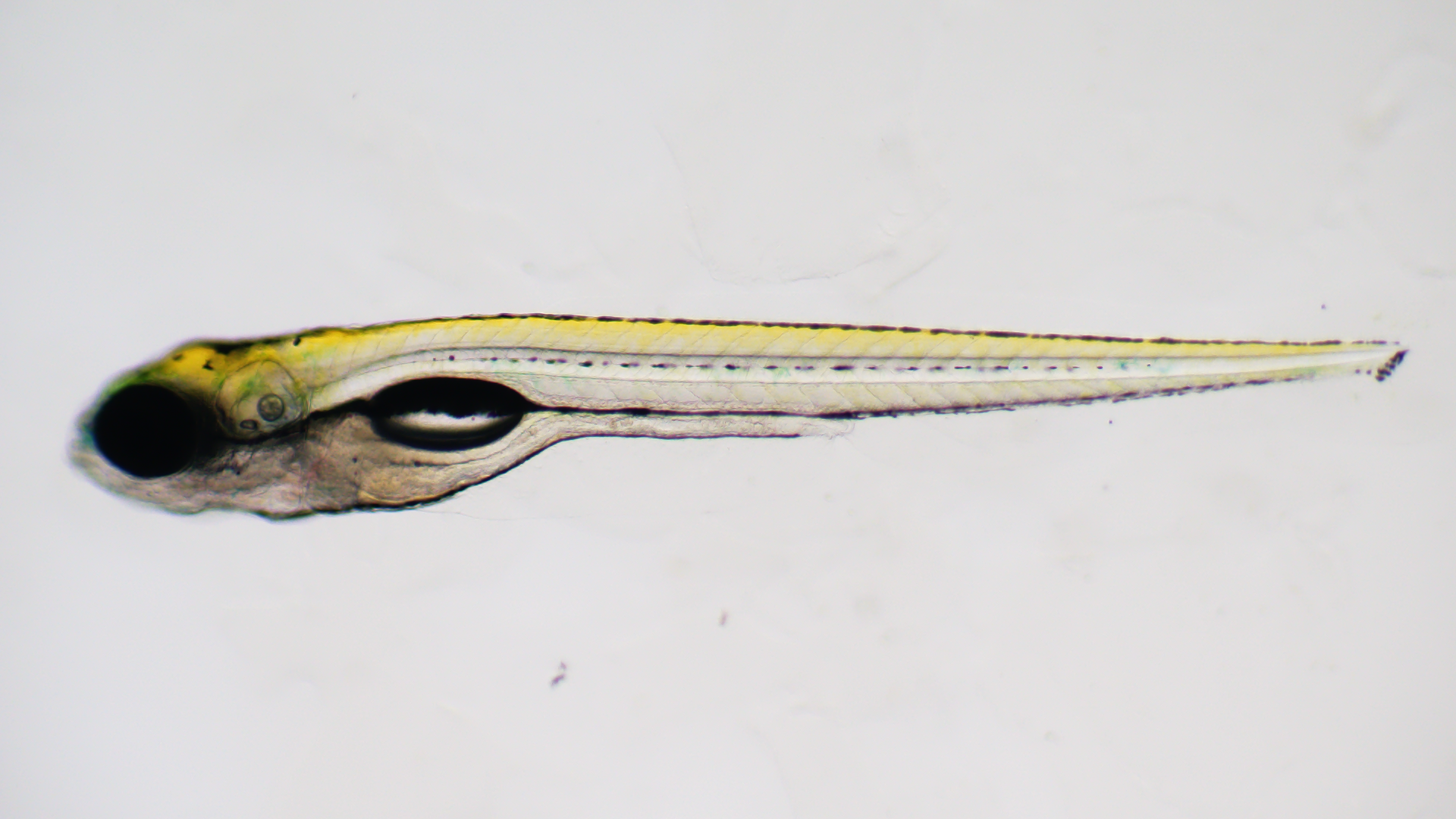

Supplement: Supplementary file 3 — Source data Fig. 3 [file 44321_2025_355_MOESM3_ESM.zip › Figure 3/3A/861VUS_6dpf.tif]

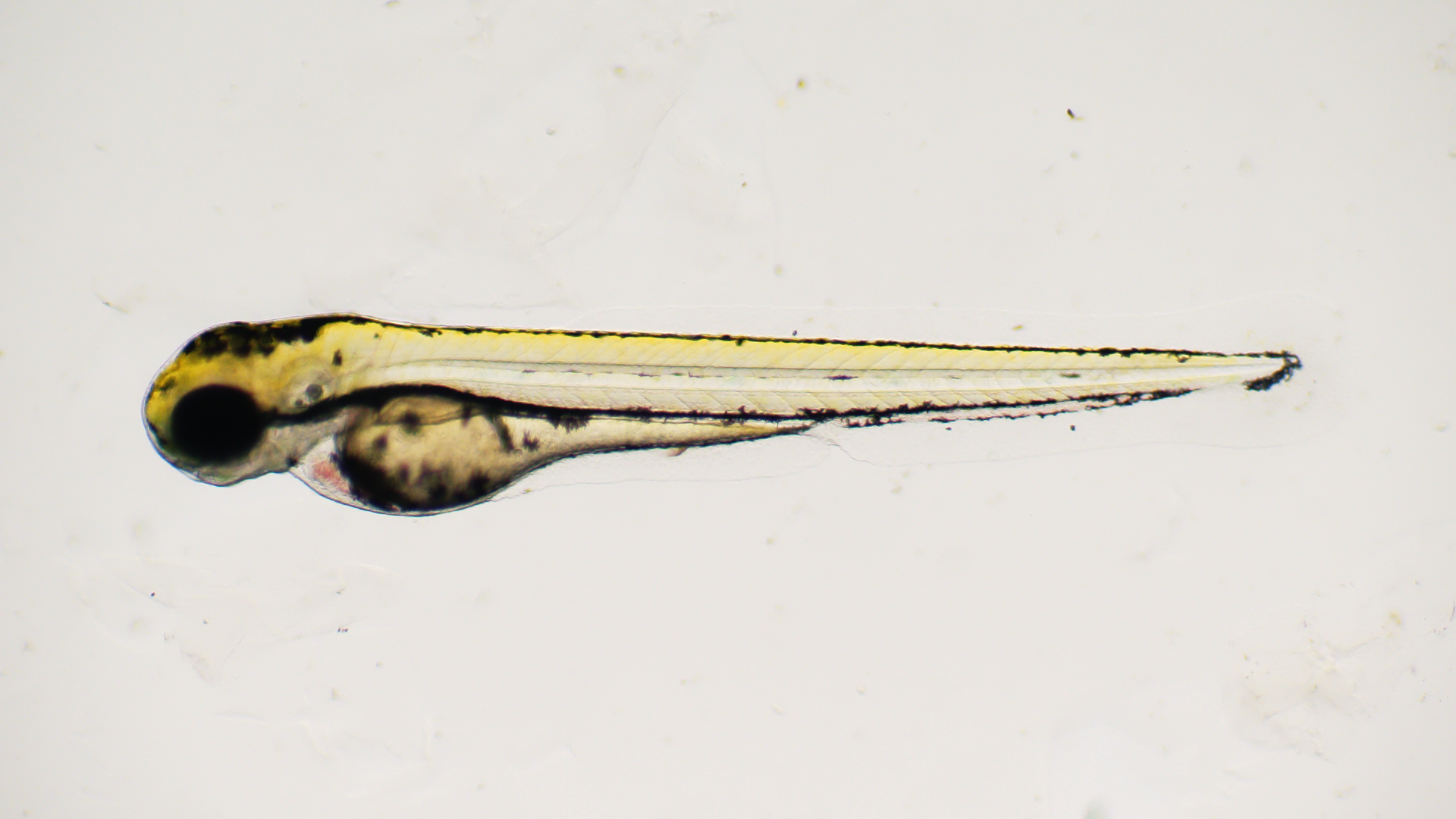

Supplement: Supplementary file 3 — Source data Fig. 3 [file 44321_2025_355_MOESM3_ESM.zip › Figure 3/3A/WT_3dpf.tif]

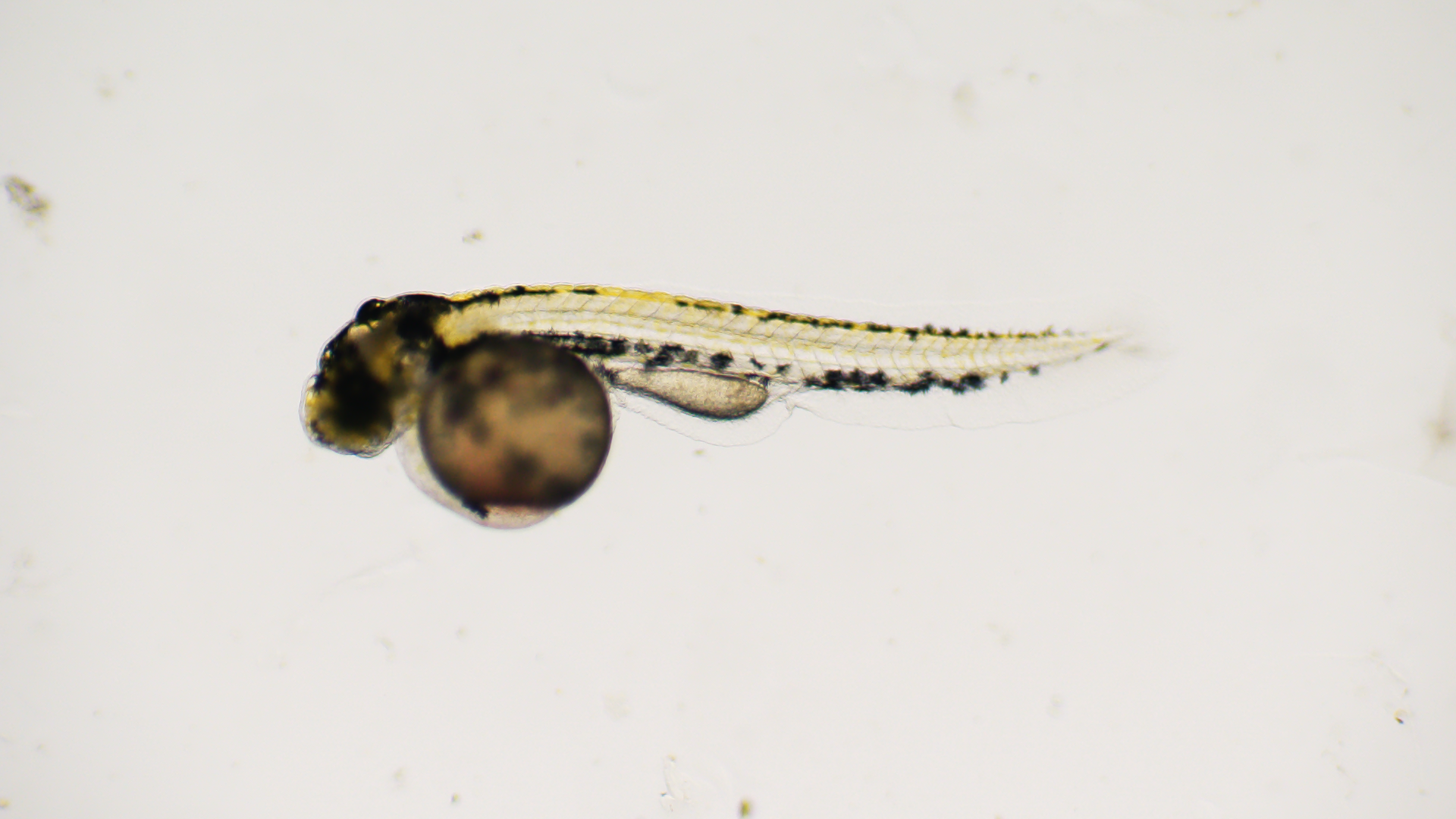

Supplement: Supplementary file 3 — Source data Fig. 3 [file 44321_2025_355_MOESM3_ESM.zip › Figure 3/3A/smn_null_3dpf.tif]

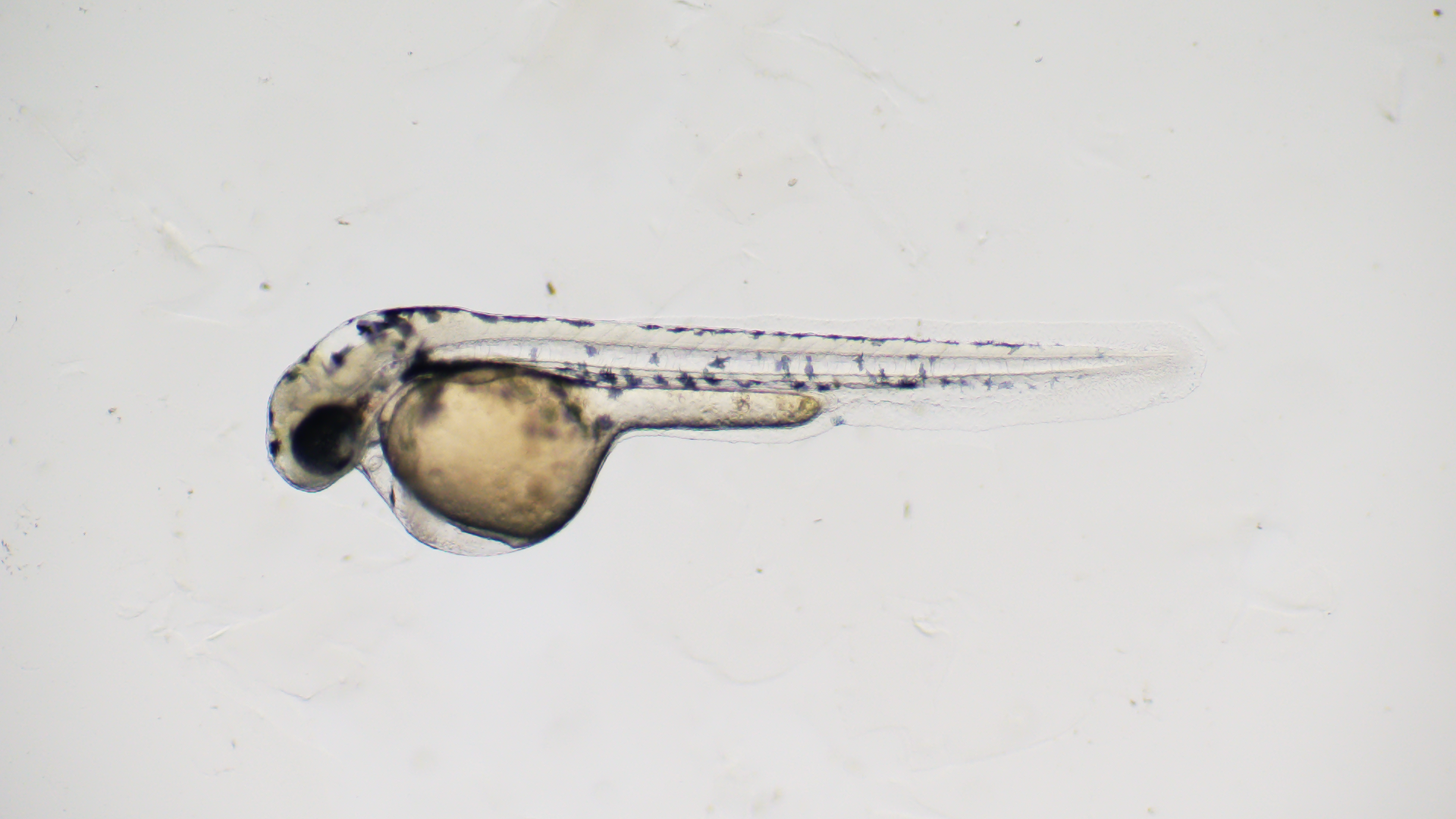

Supplement: Supplementary file 3 — Source data Fig. 3 [file 44321_2025_355_MOESM3_ESM.zip › Figure 3/3A/path_2dpf.tif]

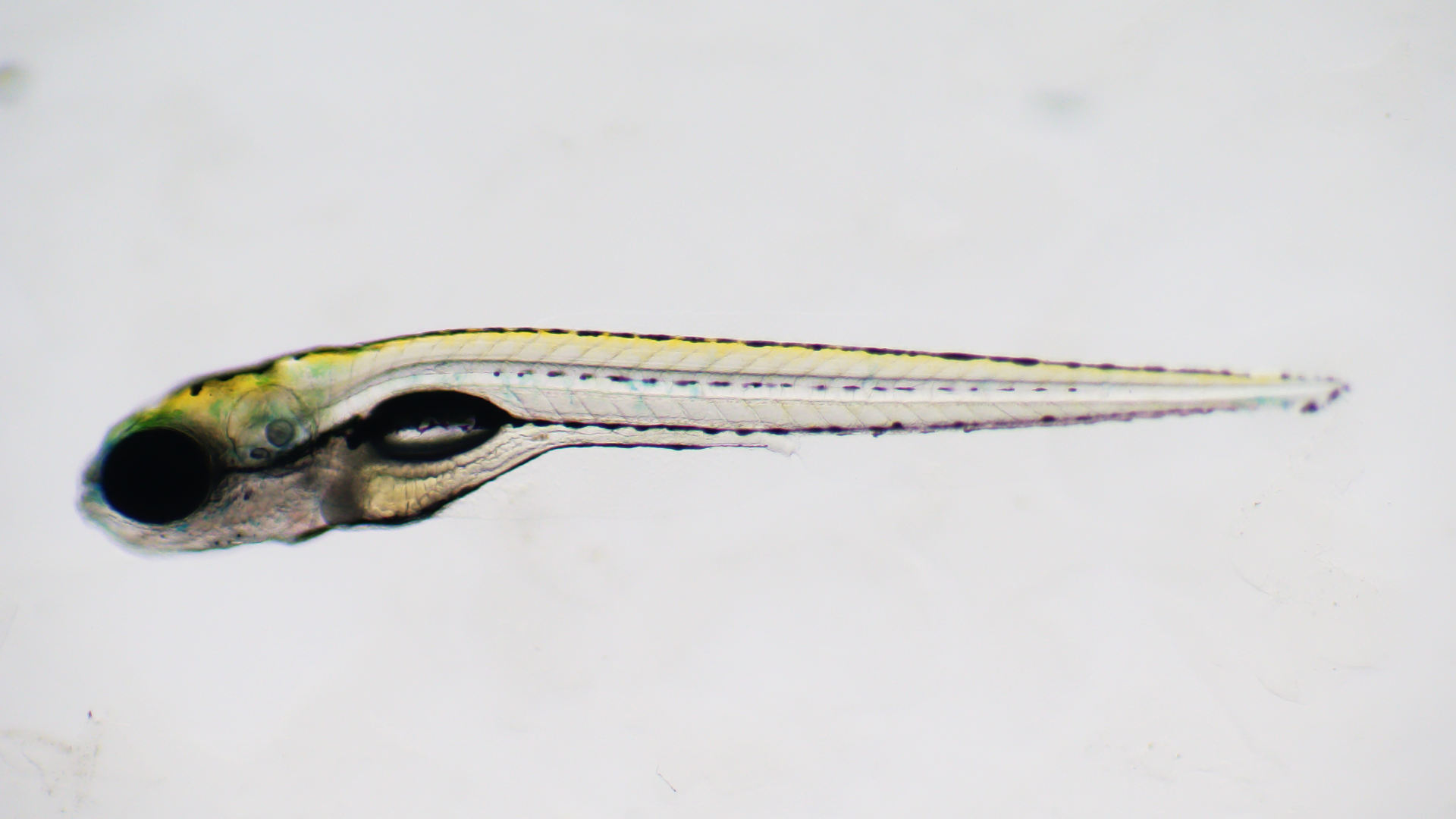

Supplement: Supplementary file 3 — Source data Fig. 3 [file 44321_2025_355_MOESM3_ESM.zip › Figure 3/3A/Tg(SMN1)_8dpf.tif]

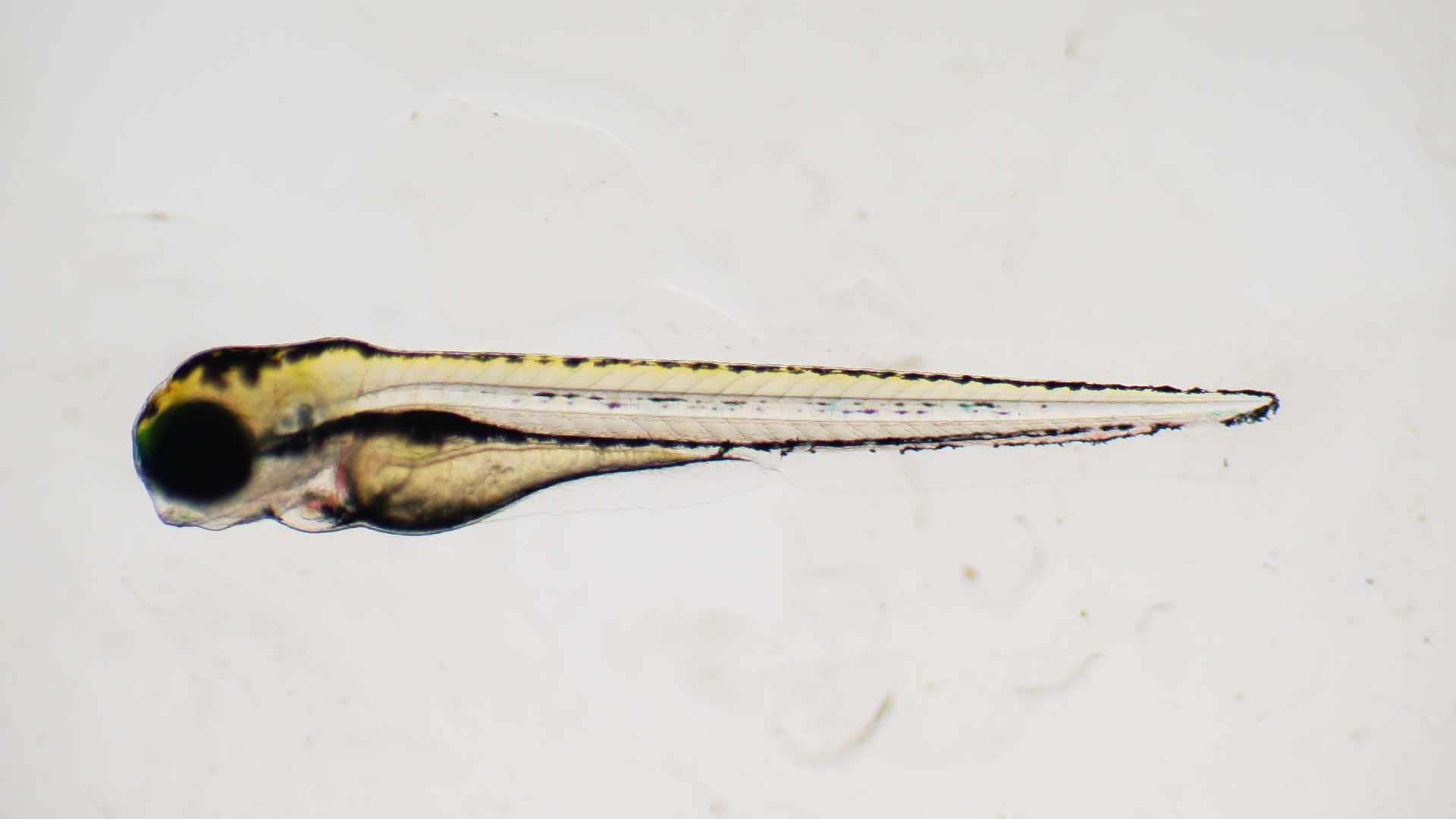

Supplement: Supplementary file 3 — Source data Fig. 3 [file 44321_2025_355_MOESM3_ESM.zip › Figure 3/3A/Tg(SMN1)_4dpf.tif]

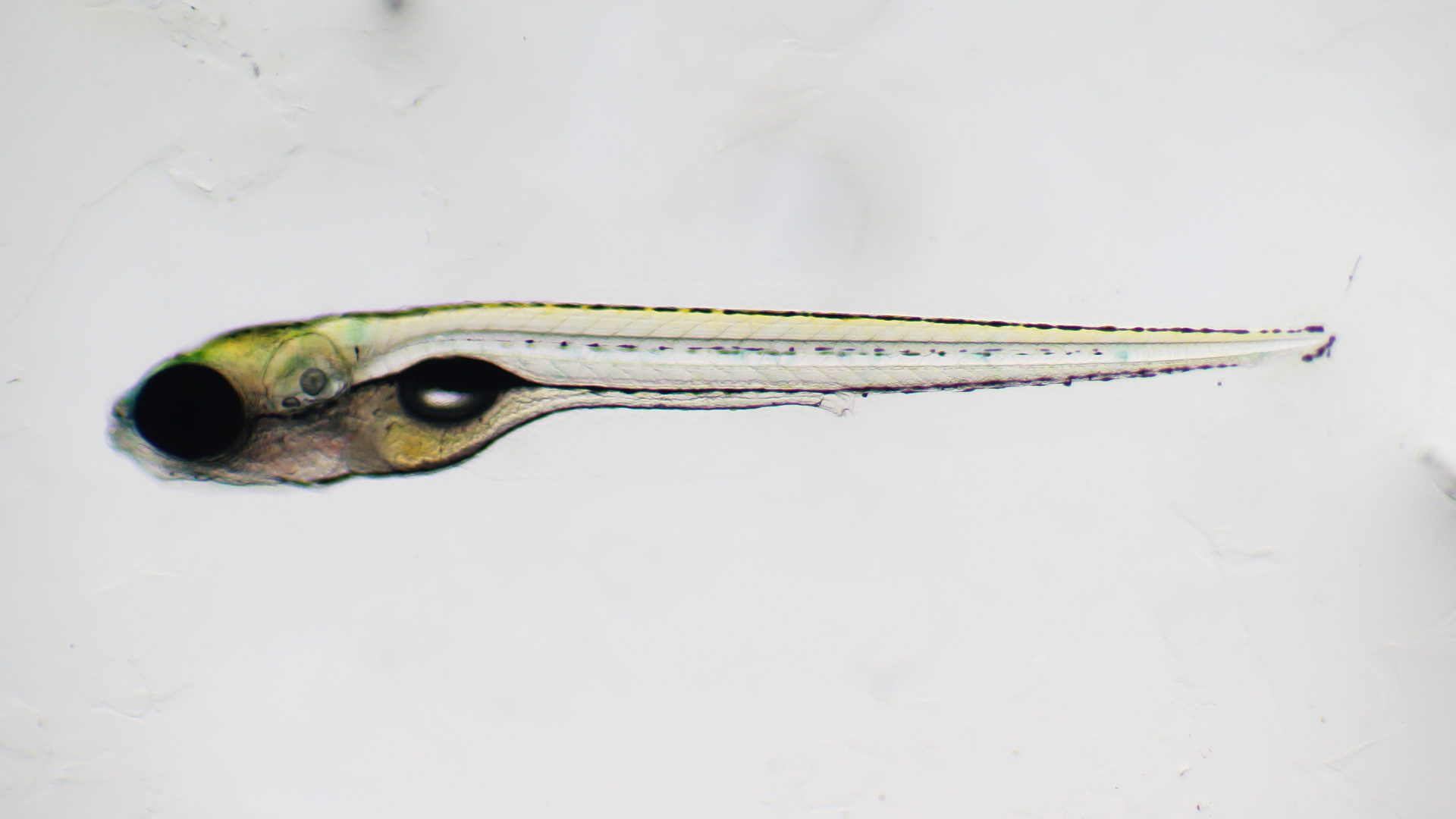

Supplement: Supplementary file 3 — Source data Fig. 3 [file 44321_2025_355_MOESM3_ESM.zip › Figure 3/3A/control_7dpf.tif]

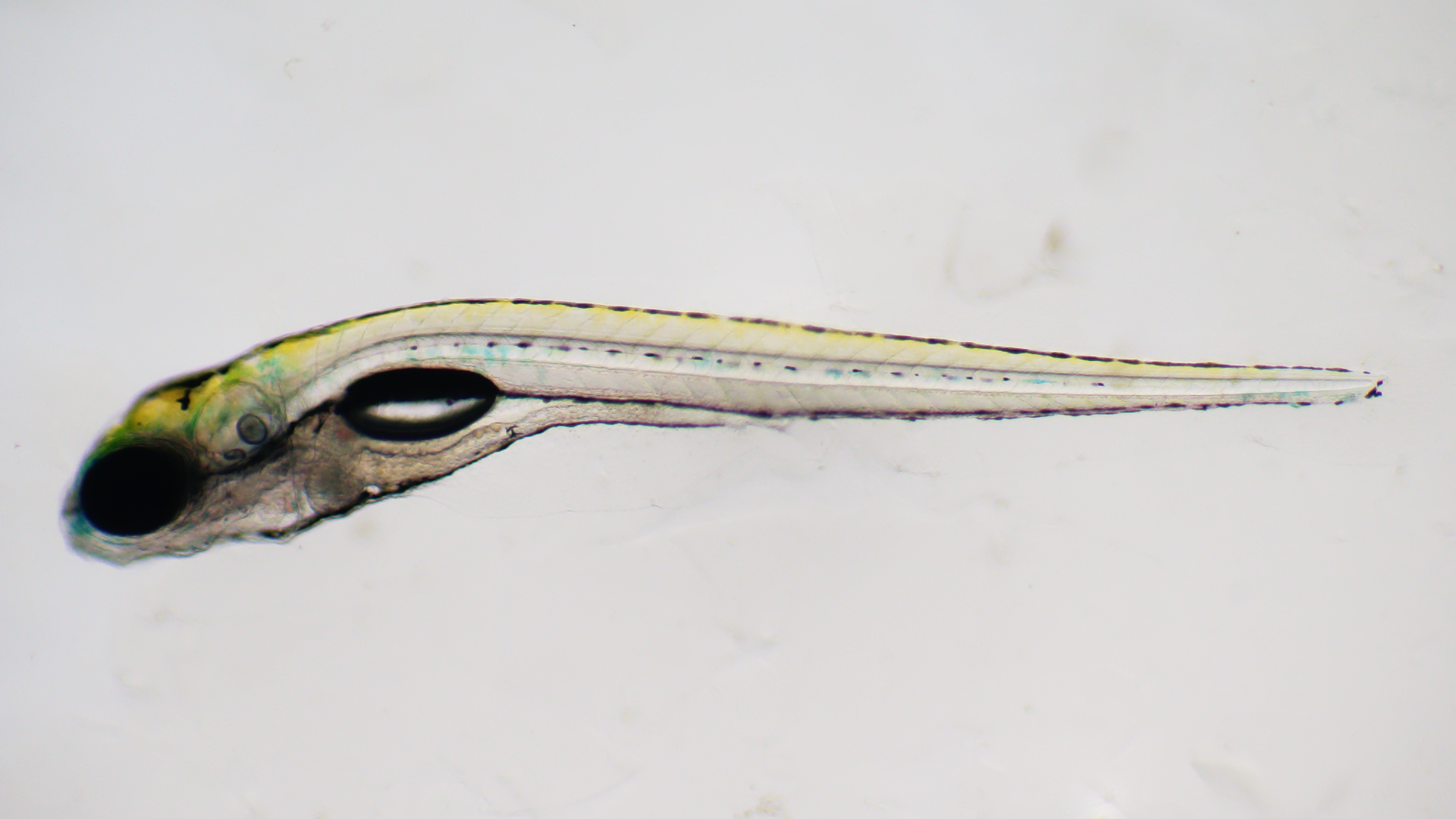

Supplement: Supplementary file 3 — Source data Fig. 3 [file 44321_2025_355_MOESM3_ESM.zip › Figure 3/3A/non-path_7dpf.tif]

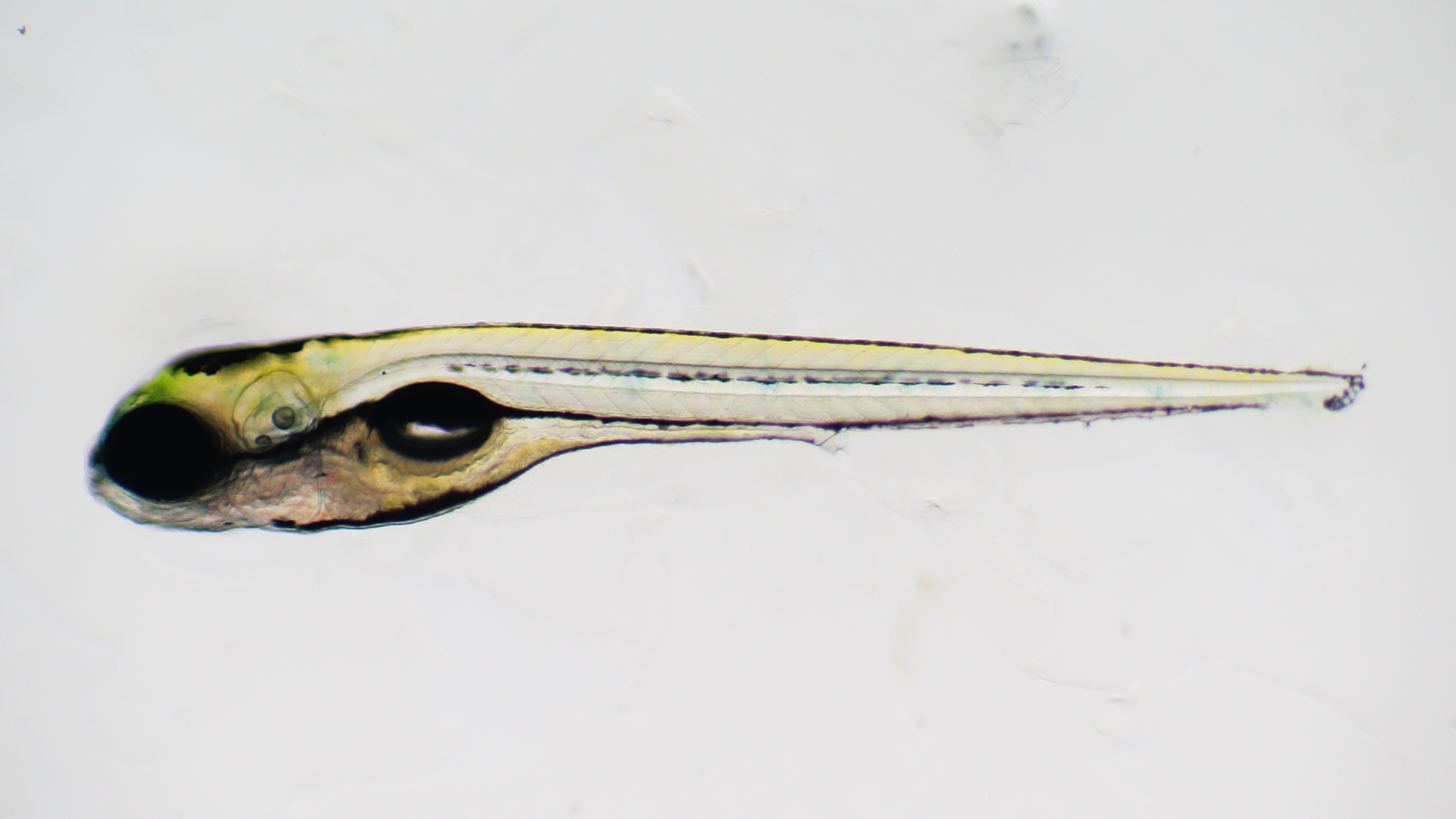

Supplement: Supplementary file 3 — Source data Fig. 3 [file 44321_2025_355_MOESM3_ESM.zip › Figure 3/3A/control_5dpf.tif]

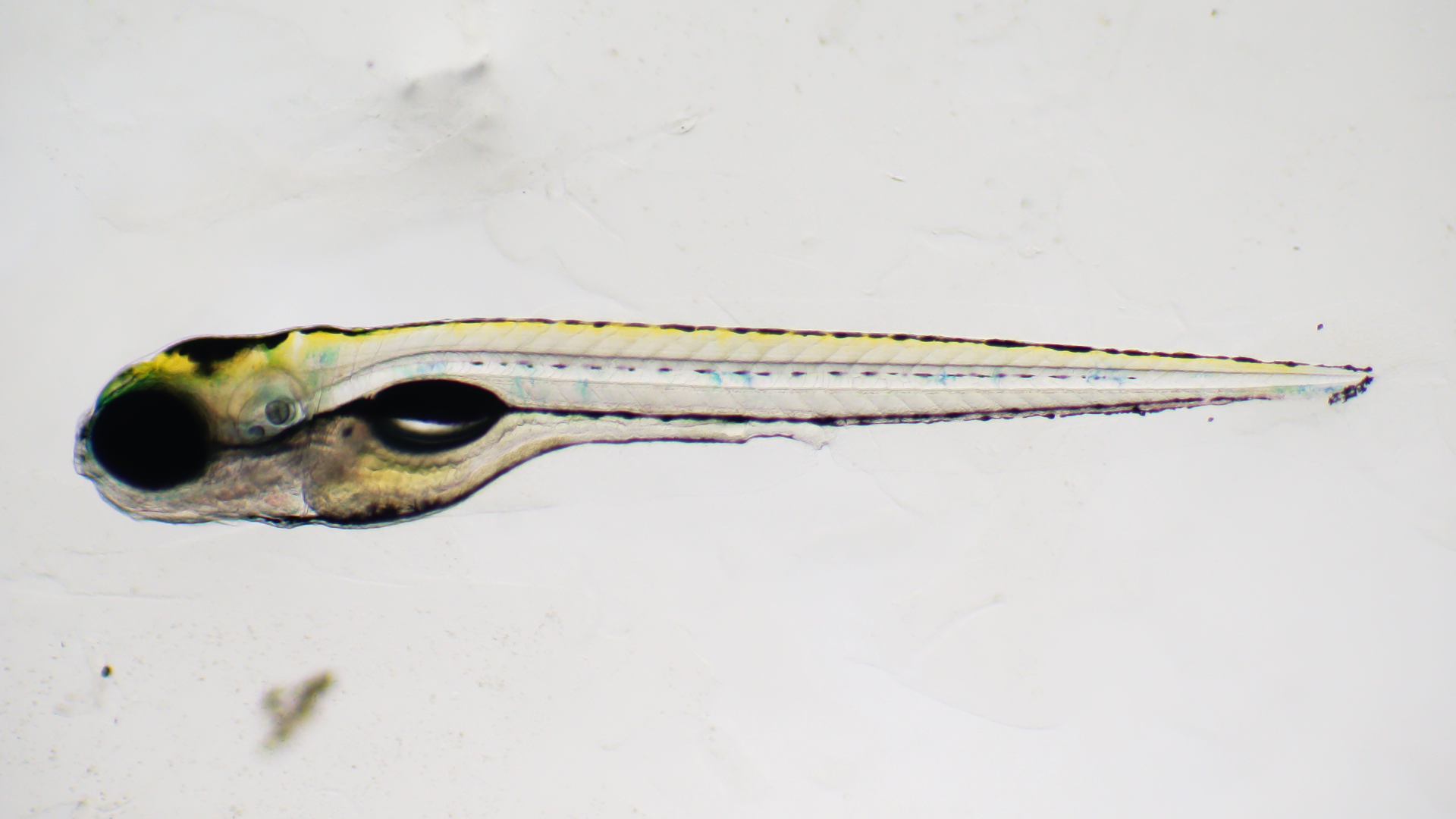

Supplement: Supplementary file 3 — Source data Fig. 3 [file 44321_2025_355_MOESM3_ESM.zip › Figure 3/3A/Tg(SMN1)_6dpf.tif]

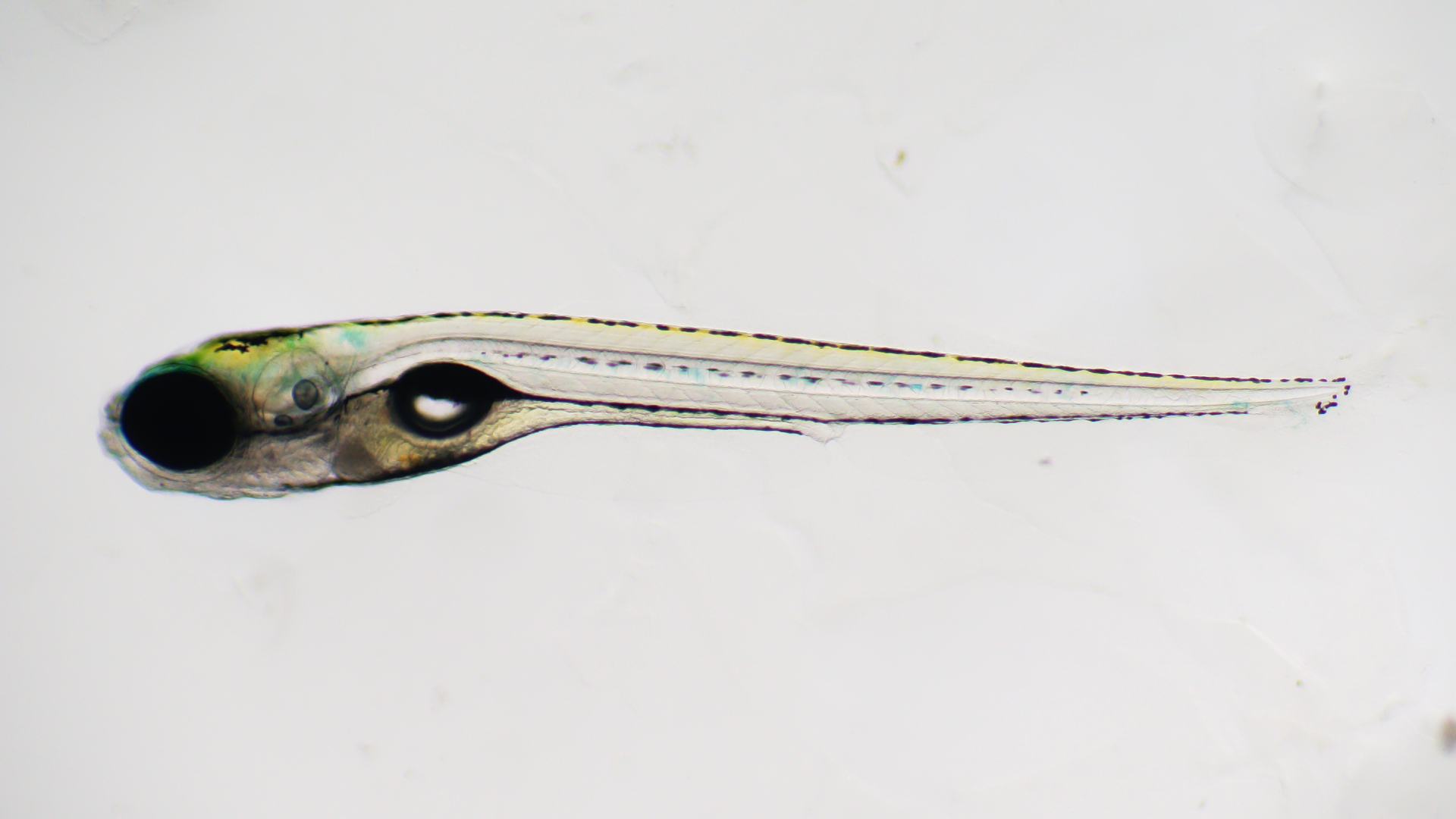

Supplement: Supplementary file 3 — Source data Fig. 3 [file 44321_2025_355_MOESM3_ESM.zip › Figure 3/3A/control_9dpf.tif]

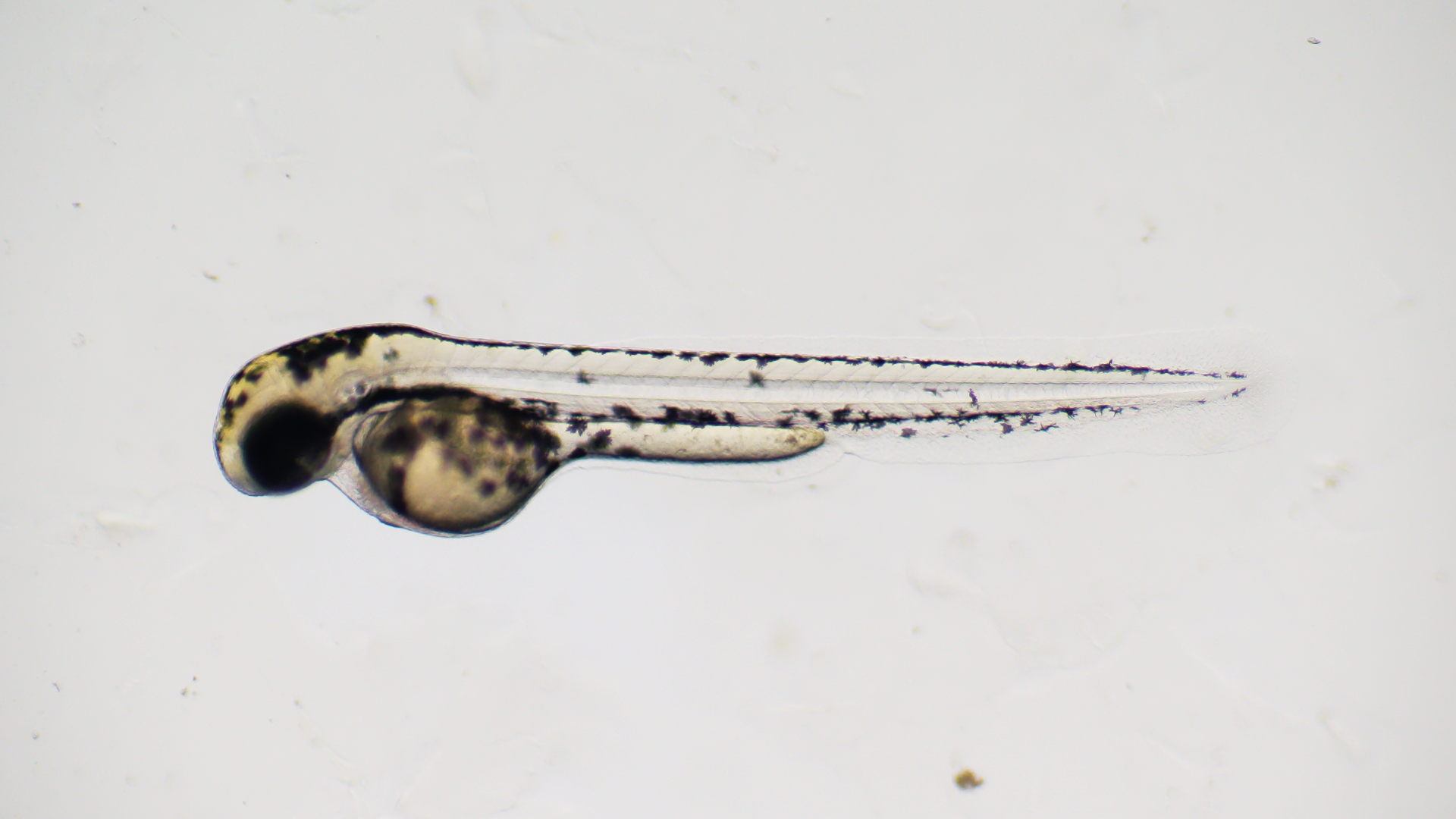

Supplement: Supplementary file 3 — Source data Fig. 3 [file 44321_2025_355_MOESM3_ESM.zip › Figure 3/3A/855VUS_2dpf.tif]

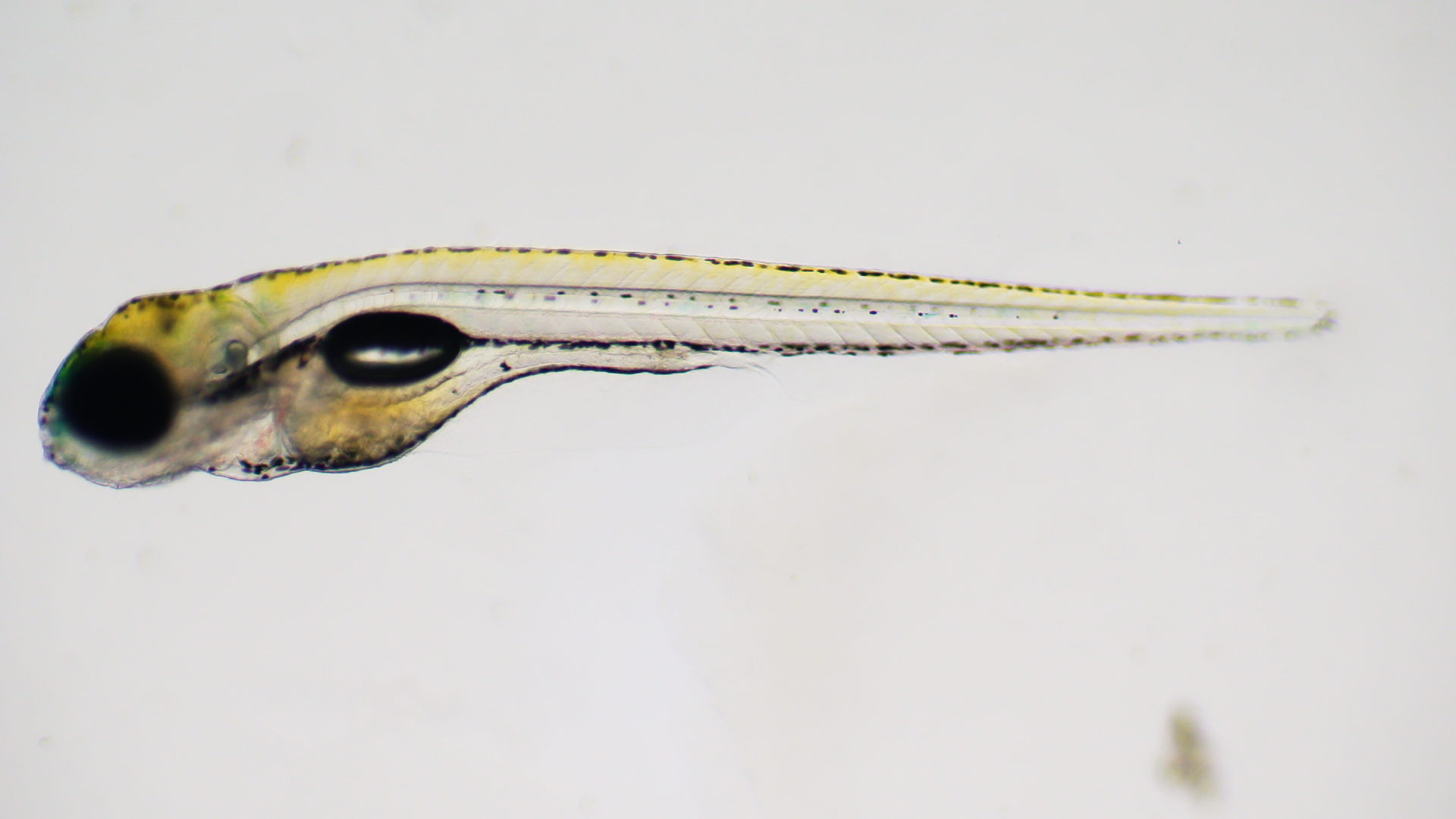

Supplement: Supplementary file 3 — Source data Fig. 3 [file 44321_2025_355_MOESM3_ESM.zip › Figure 3/3A/non-path_5dpf.tif]

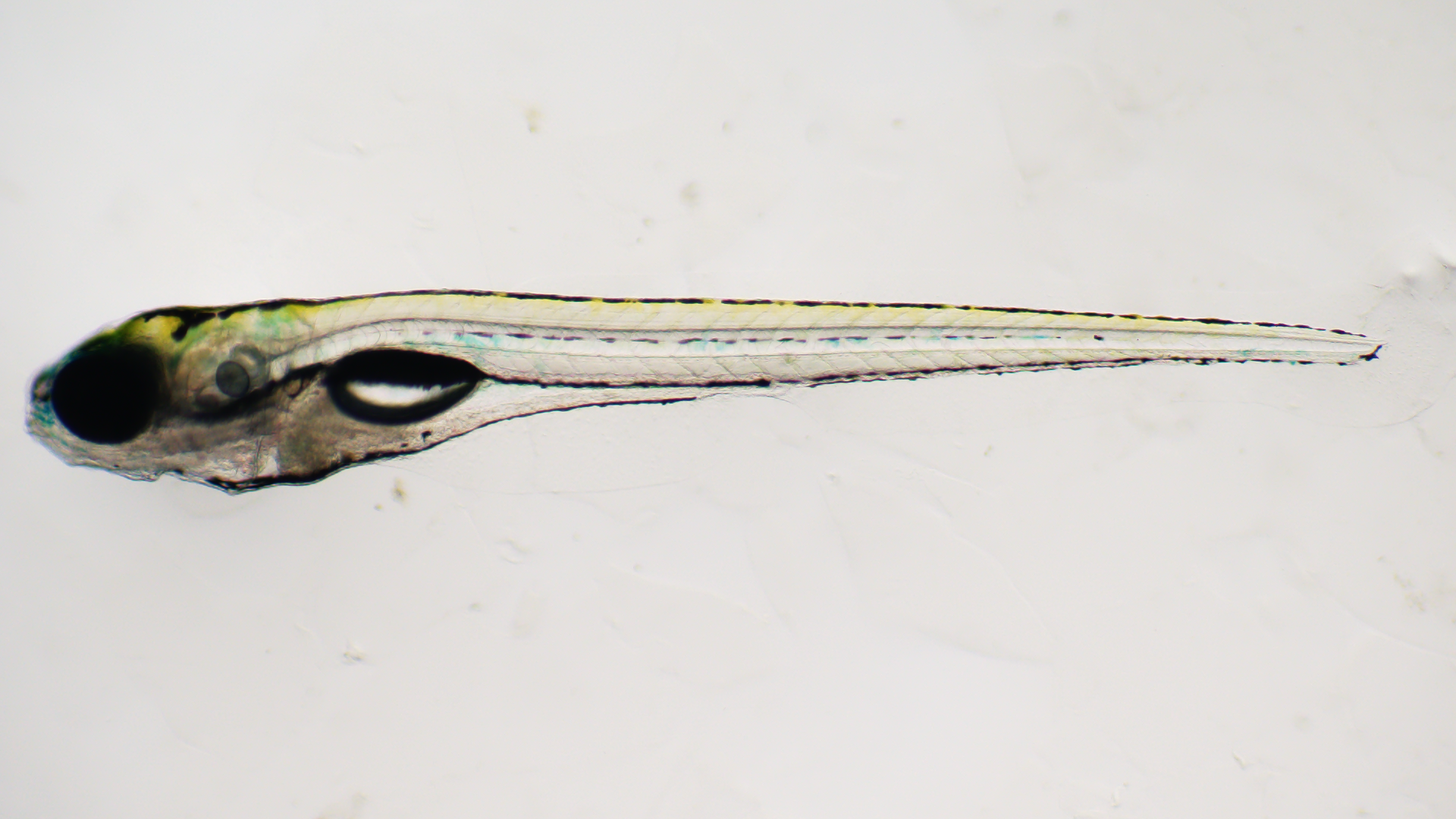

Supplement: Supplementary file 3 — Source data Fig. 3 [file 44321_2025_355_MOESM3_ESM.zip › Figure 3/3A/non-path_9dpf.tif]

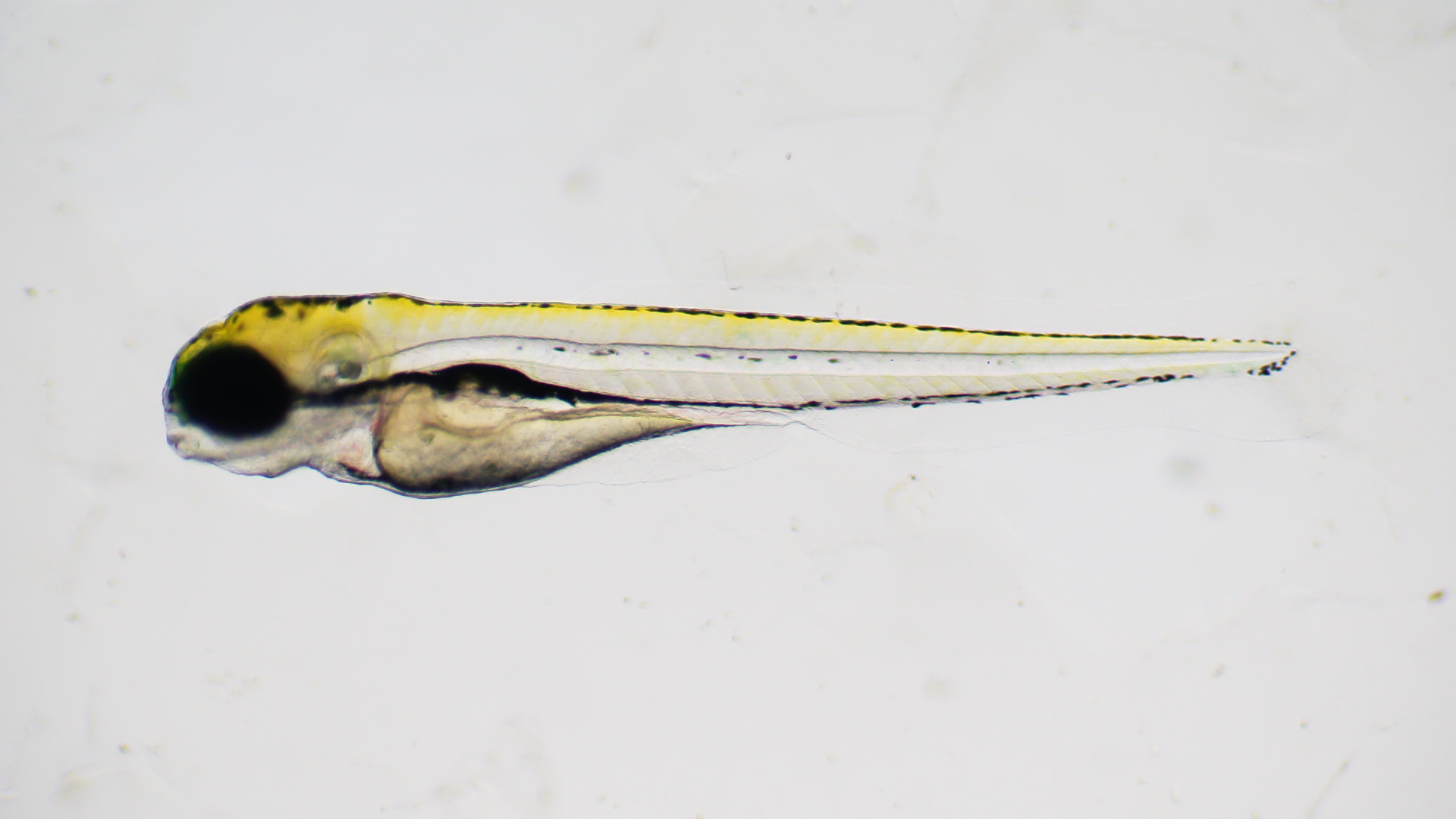

Supplement: Supplementary file 3 — Source data Fig. 3 [file 44321_2025_355_MOESM3_ESM.zip › Figure 3/3A/861VUS_4dpf.tif]

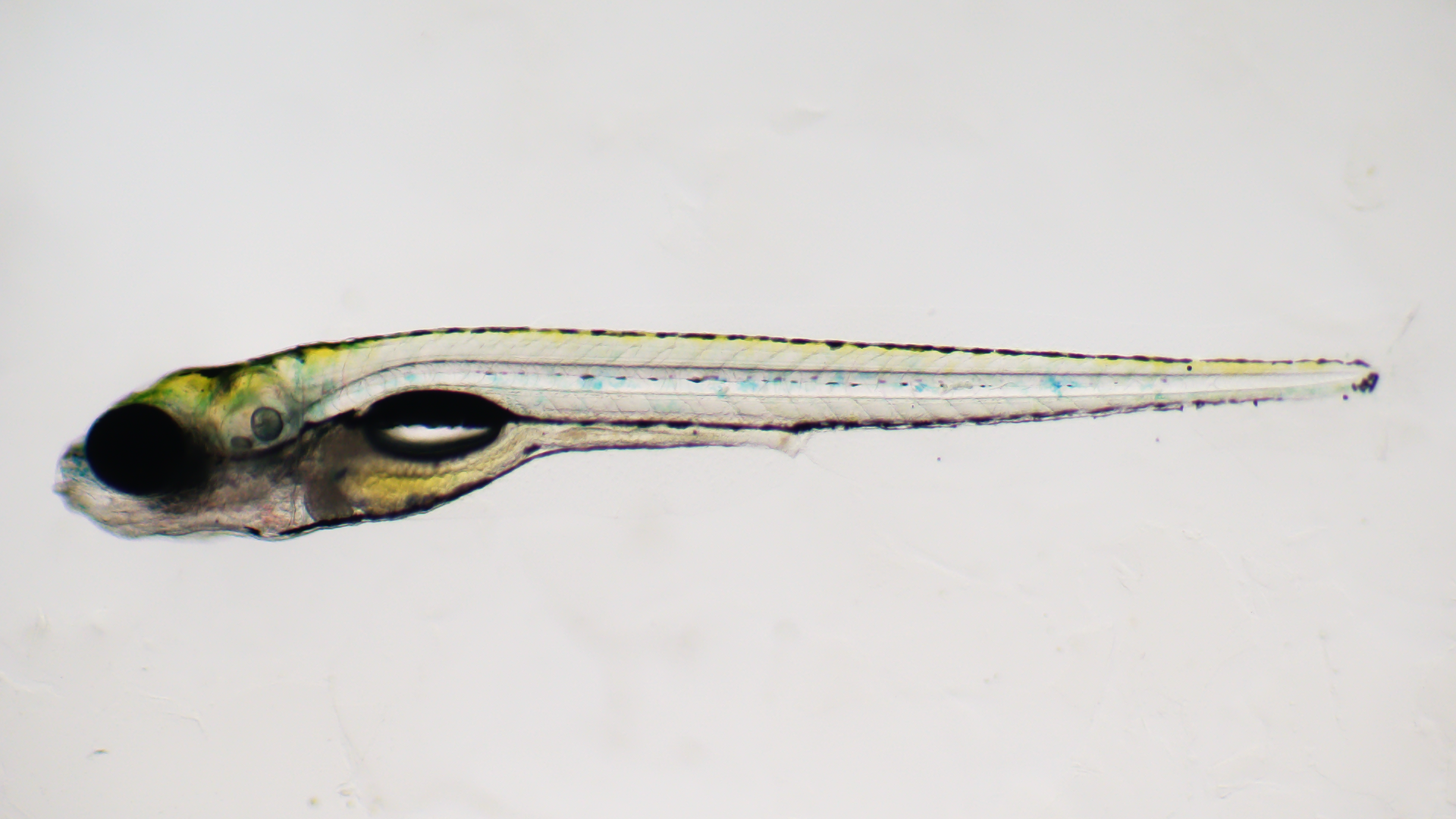

Supplement: Supplementary file 3 — Source data Fig. 3 [file 44321_2025_355_MOESM3_ESM.zip › Figure 3/3A/861VUS_8dpf.tif]

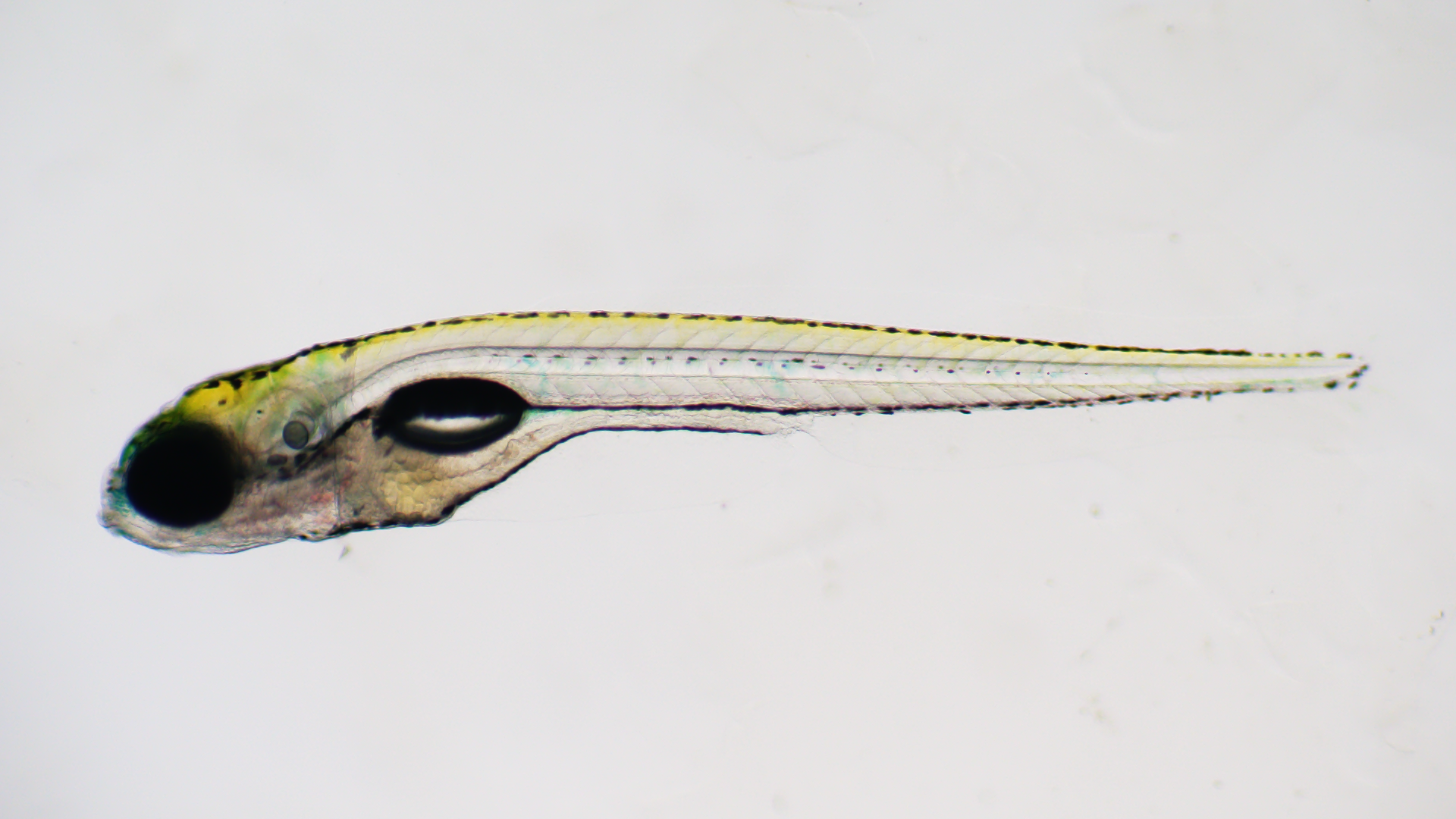

Supplement: Supplementary file 3 — Source data Fig. 3 [file 44321_2025_355_MOESM3_ESM.zip › Figure 3/3A/855VUS_6dpf.tif]

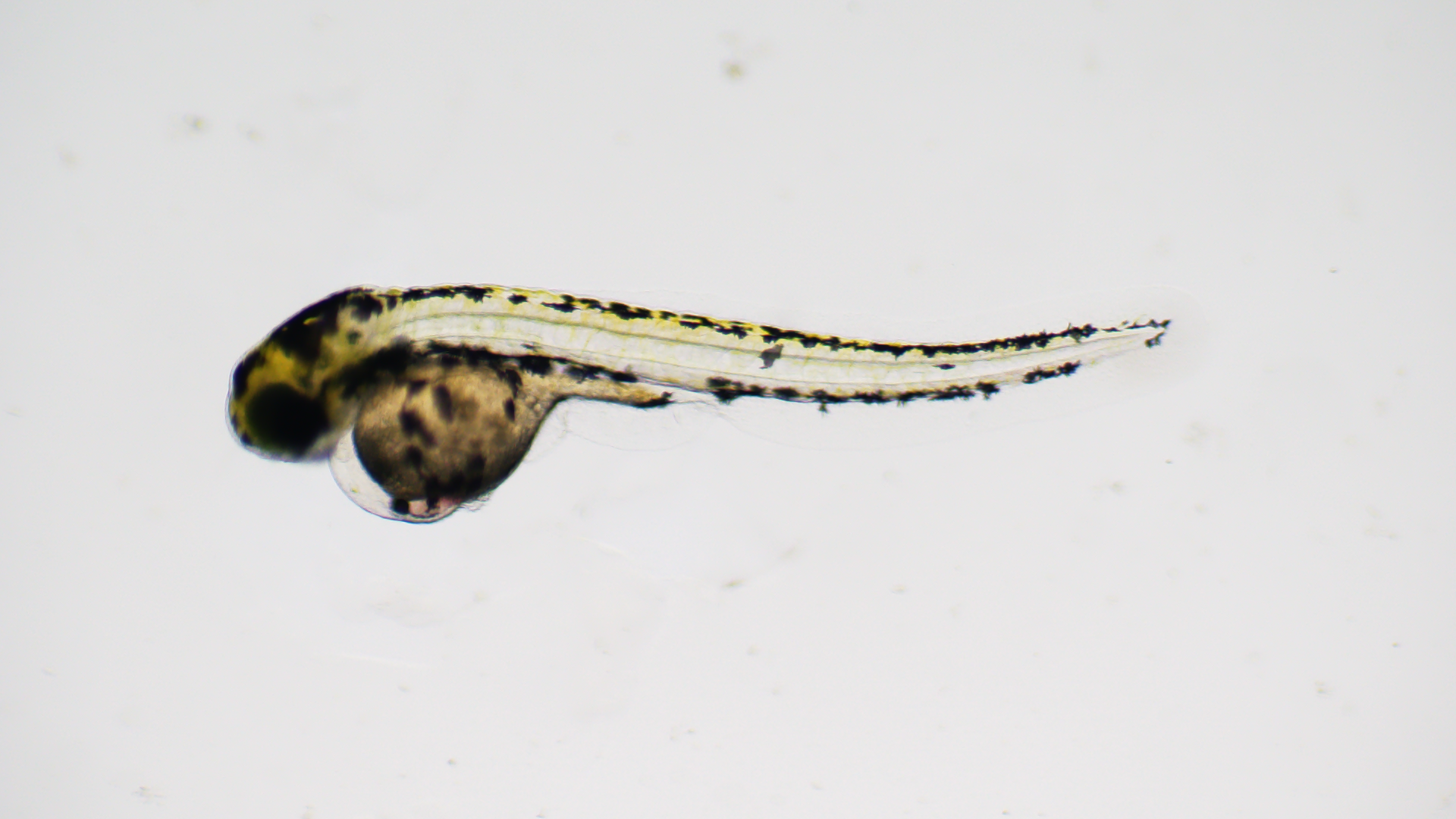

Supplement: Supplementary file 3 — Source data Fig. 3 [file 44321_2025_355_MOESM3_ESM.zip › Figure 3/3A/path_4dpf.tif]

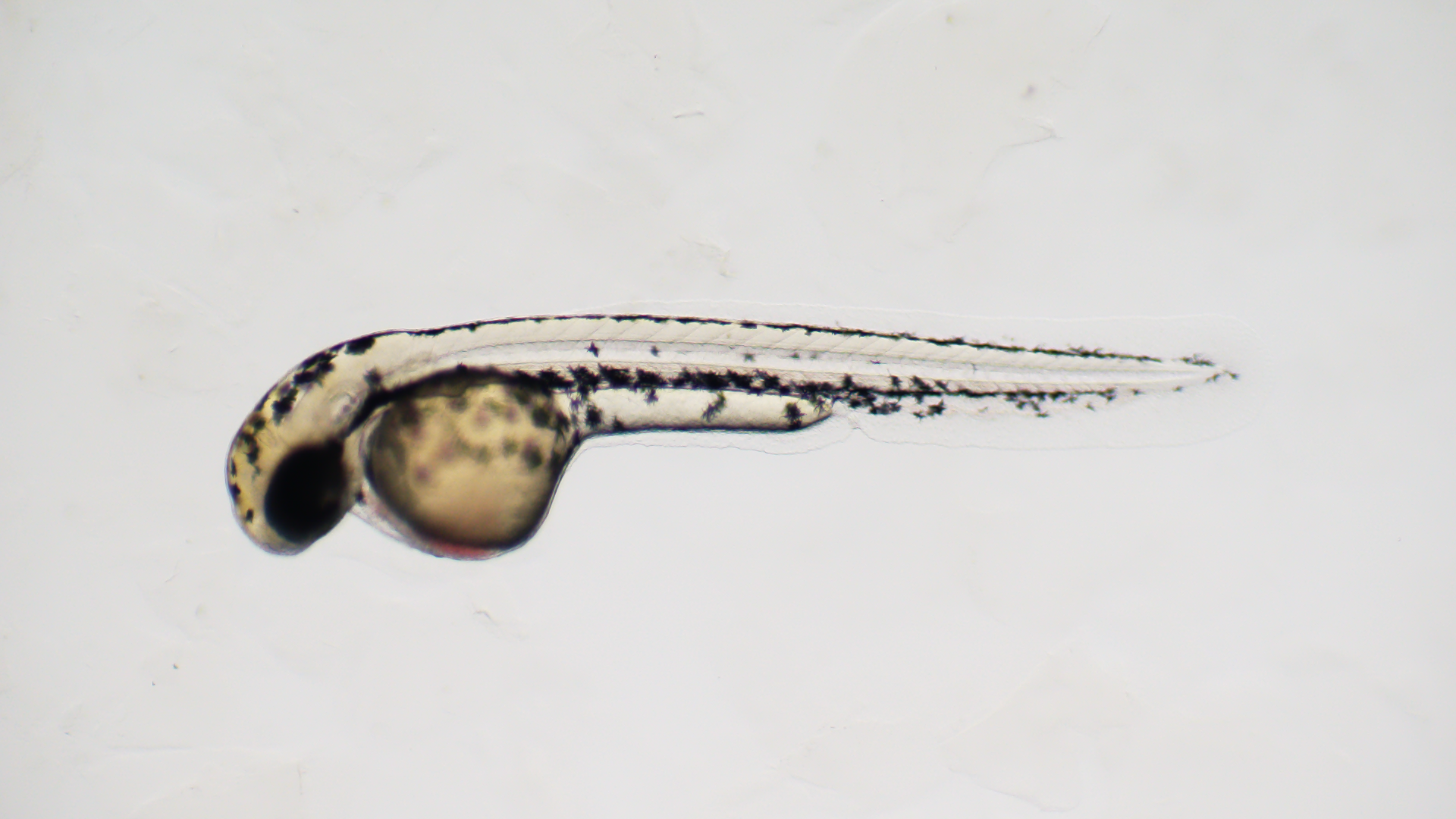

Supplement: Supplementary file 3 — Source data Fig. 3 [file 44321_2025_355_MOESM3_ESM.zip › Figure 3/3A/Tg(SMN1)_2dpf.tif]

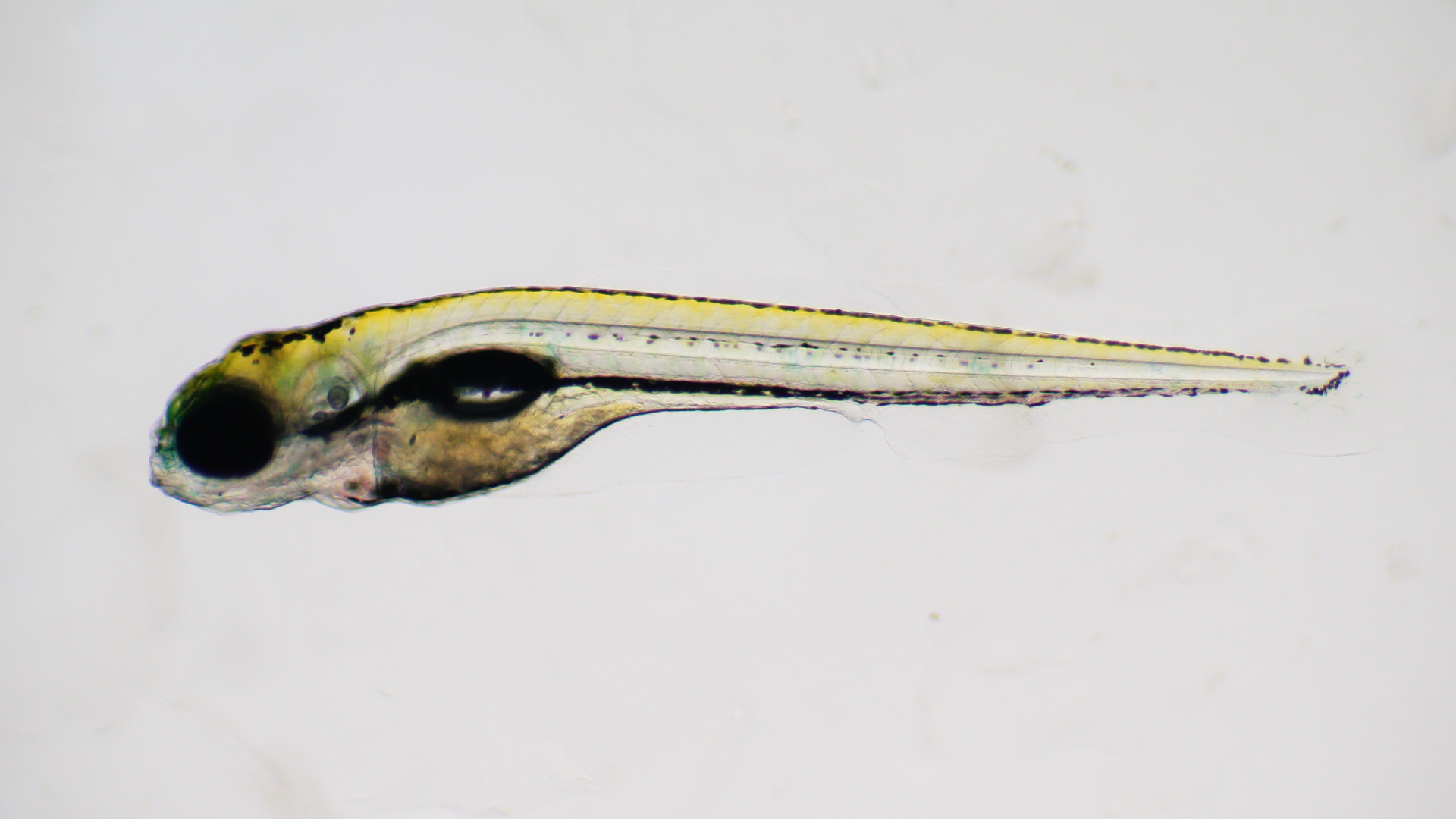

Supplement: Supplementary file 3 — Source data Fig. 3 [file 44321_2025_355_MOESM3_ESM.zip › Figure 3/3A/WT_5dpf.tif]

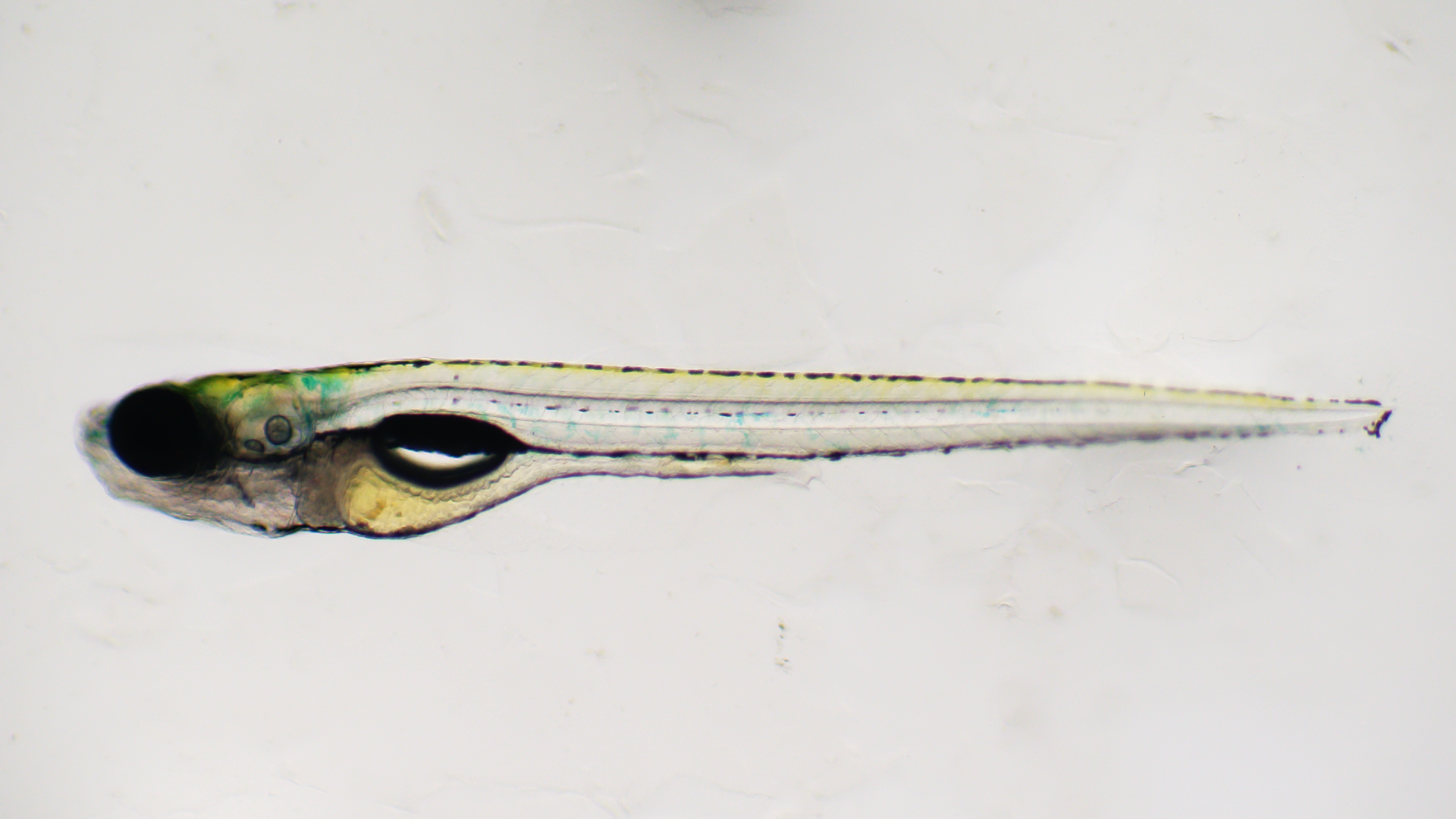

Supplement: Supplementary file 3 — Source data Fig. 3 [file 44321_2025_355_MOESM3_ESM.zip › Figure 3/3A/WT_9dpf.tif]

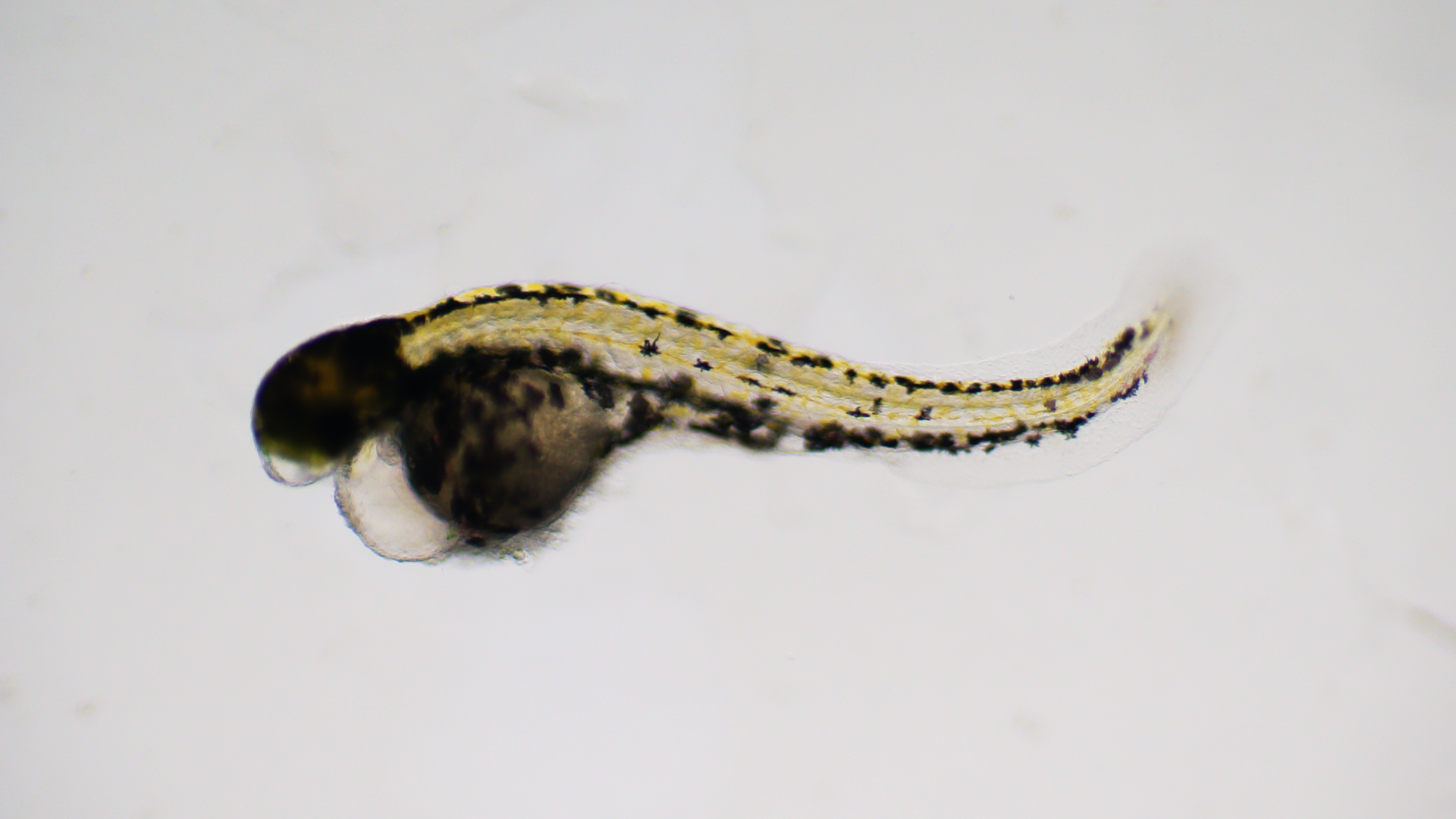

Supplement: Supplementary file 3 — Source data Fig. 3 [file 44321_2025_355_MOESM3_ESM.zip › Figure 3/3A/smn_null_5dpf.tif]

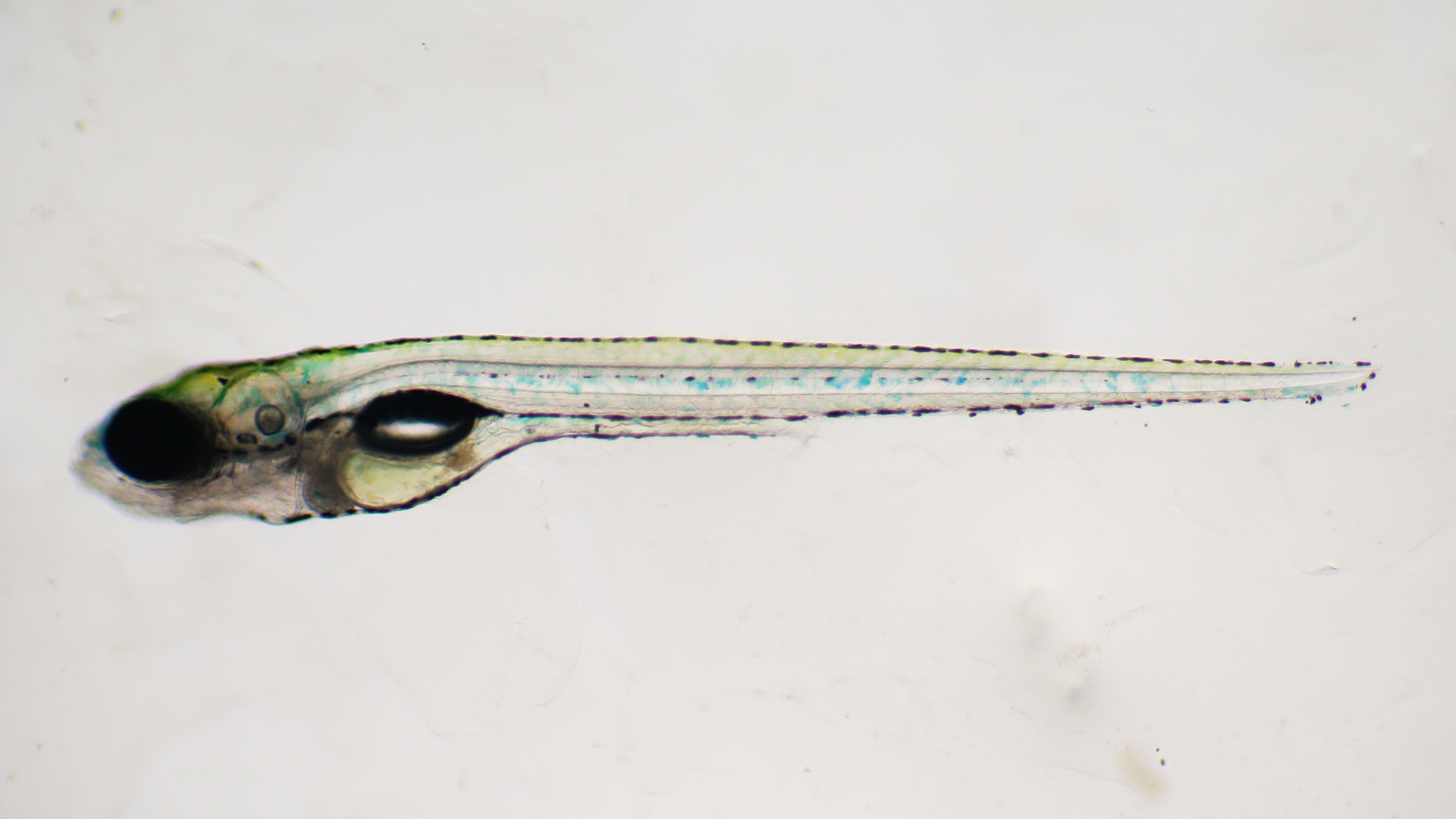

Supplement: Supplementary file 3 — Source data Fig. 3 [file 44321_2025_355_MOESM3_ESM.zip › Figure 3/3A/855VUS_10dpf.tif]

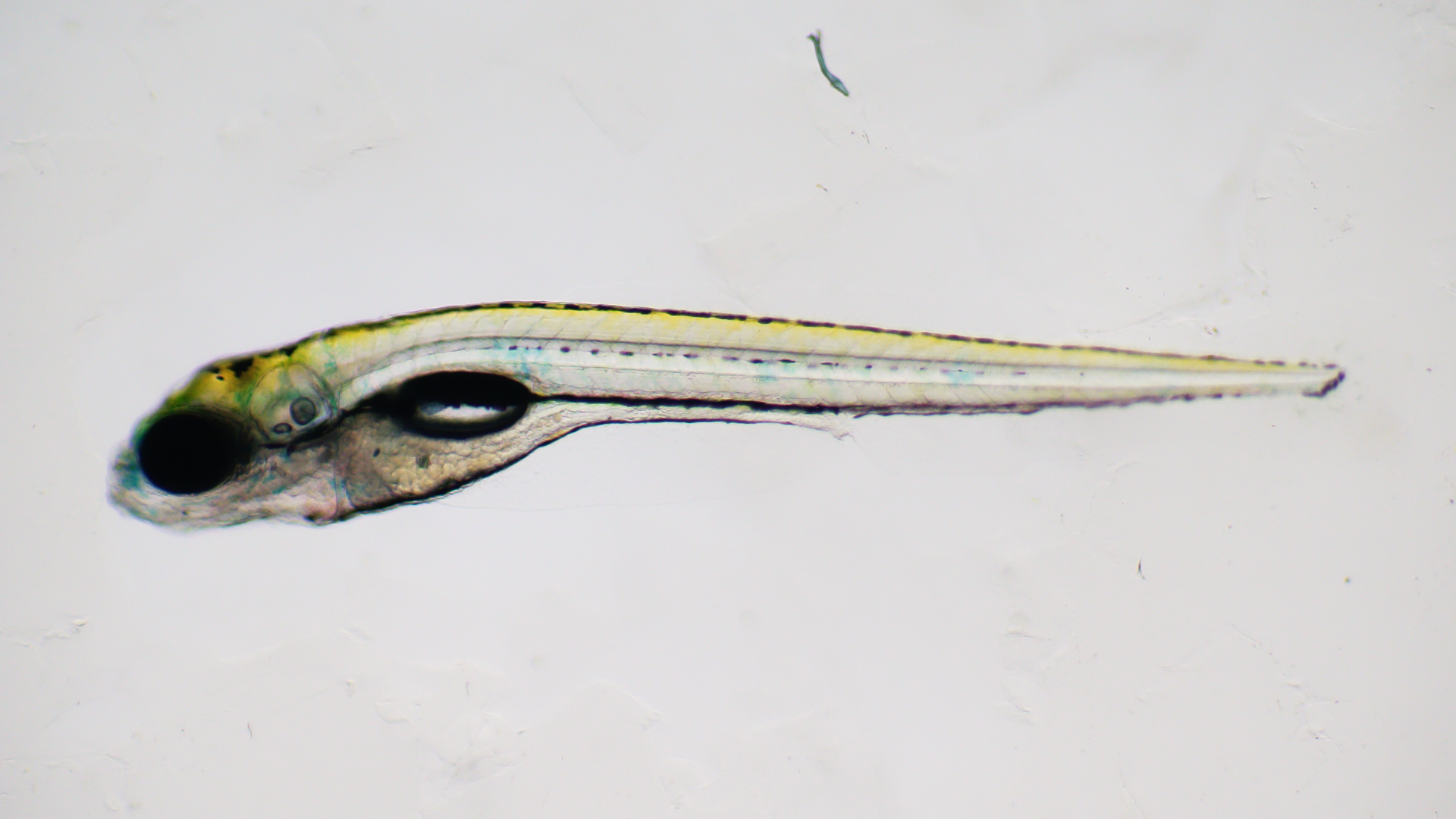

Supplement: Supplementary file 3 — Source data Fig. 3 [file 44321_2025_355_MOESM3_ESM.zip › Figure 3/3A/WT_7dpf.tif]

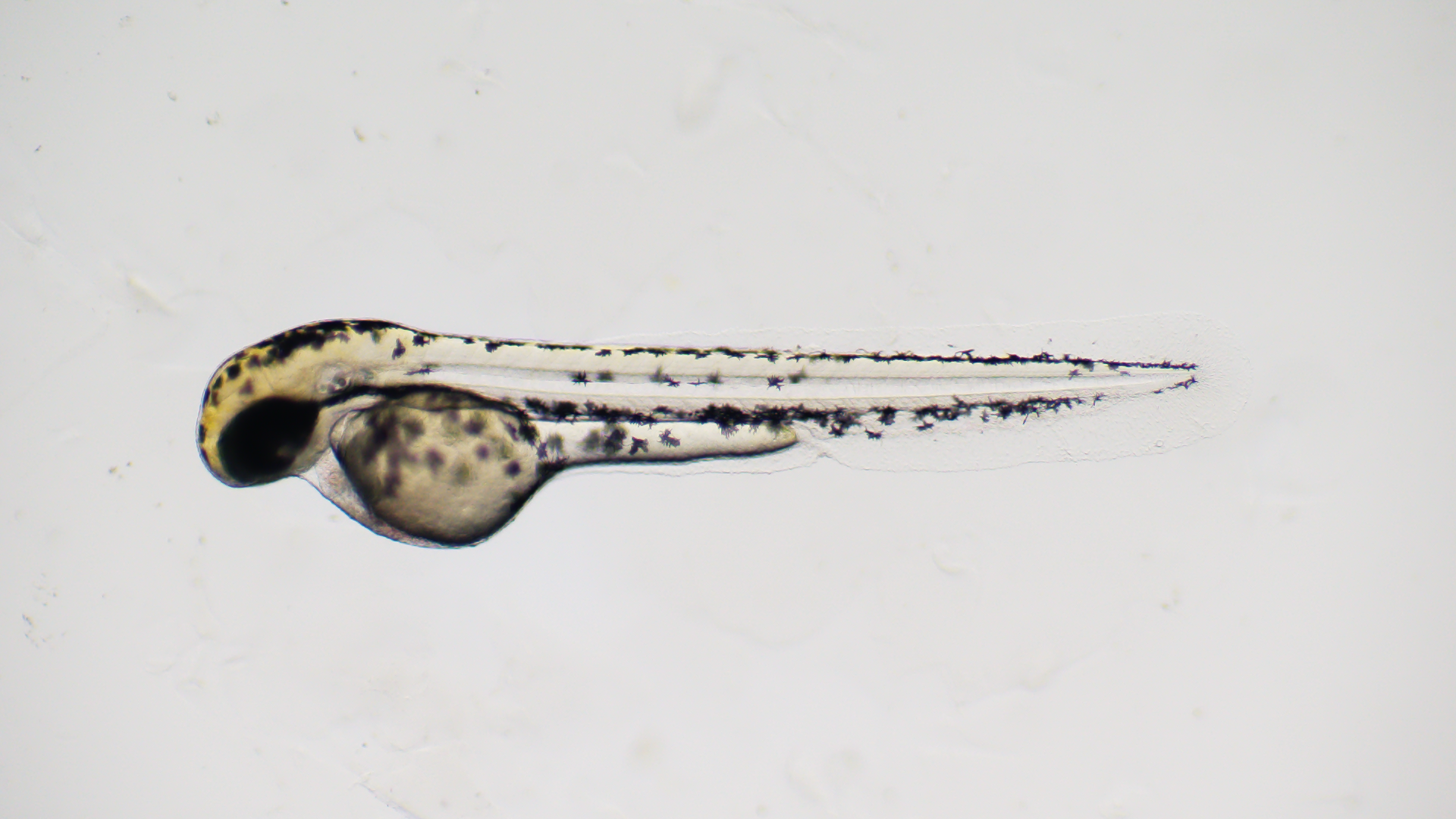

Supplement: Supplementary file 3 — Source data Fig. 3 [file 44321_2025_355_MOESM3_ESM.zip › Figure 3/3A/861VUS_2dpf.tif]

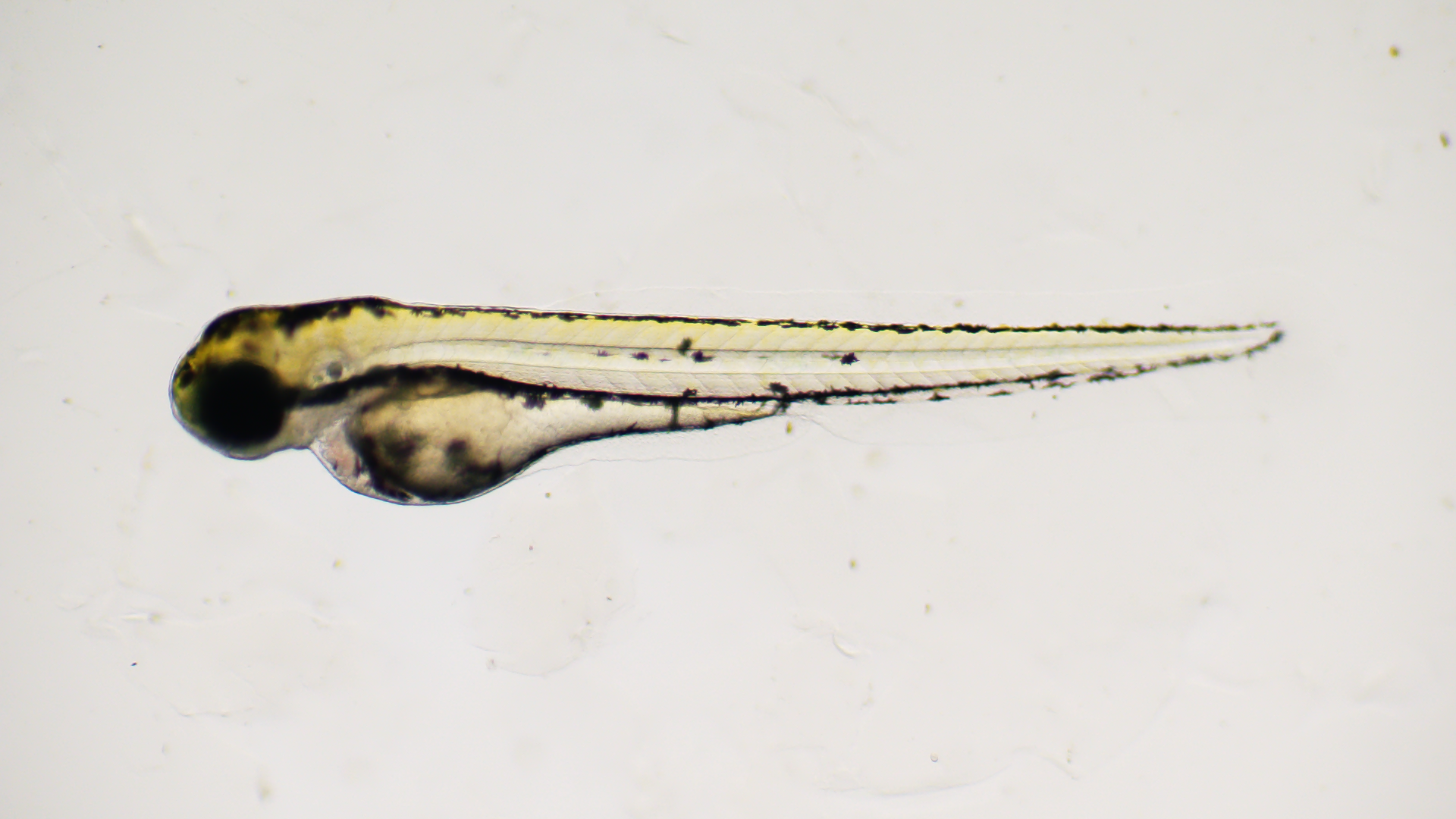

Supplement: Supplementary file 3 — Source data Fig. 3 [file 44321_2025_355_MOESM3_ESM.zip › Figure 3/3A/non-path_3dpf.tif]

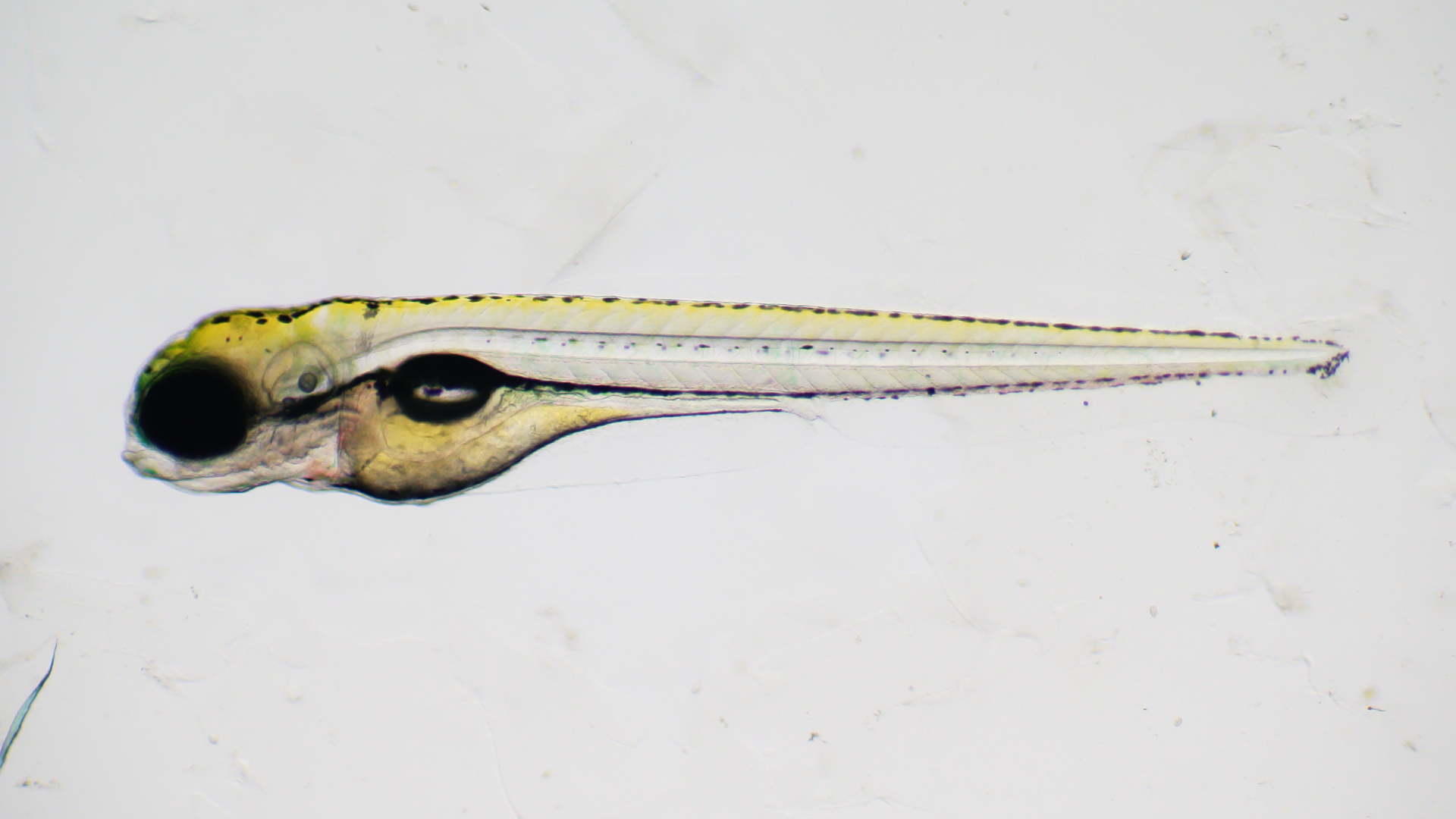

Supplement: Supplementary file 3 — Source data Fig. 3 [file 44321_2025_355_MOESM3_ESM.zip › Figure 3/3A/855VUS_4dpf.tif]

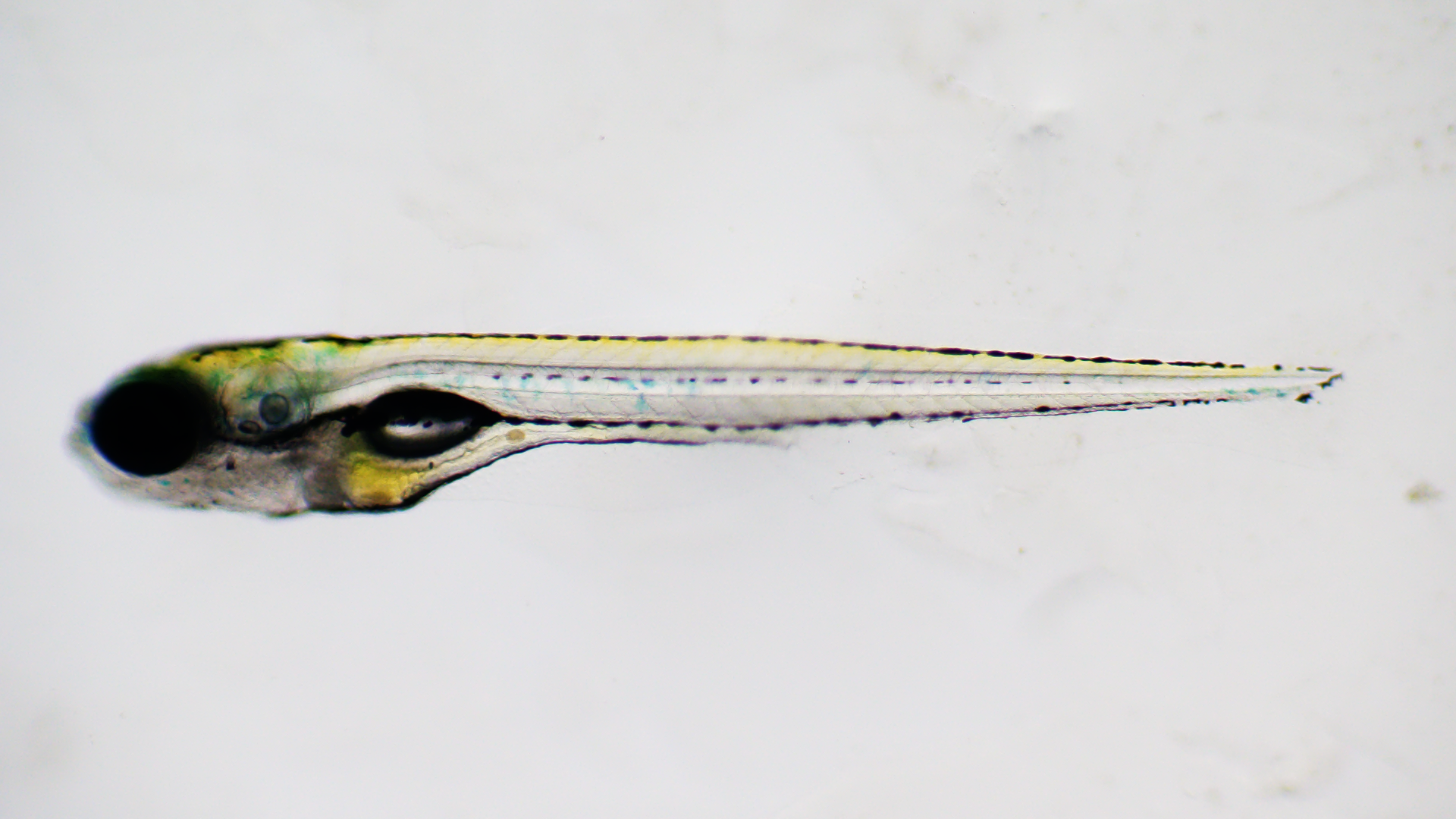

Supplement: Supplementary file 3 — Source data Fig. 3 [file 44321_2025_355_MOESM3_ESM.zip › Figure 3/3A/Tg(SMN1)_10dpf.tif]

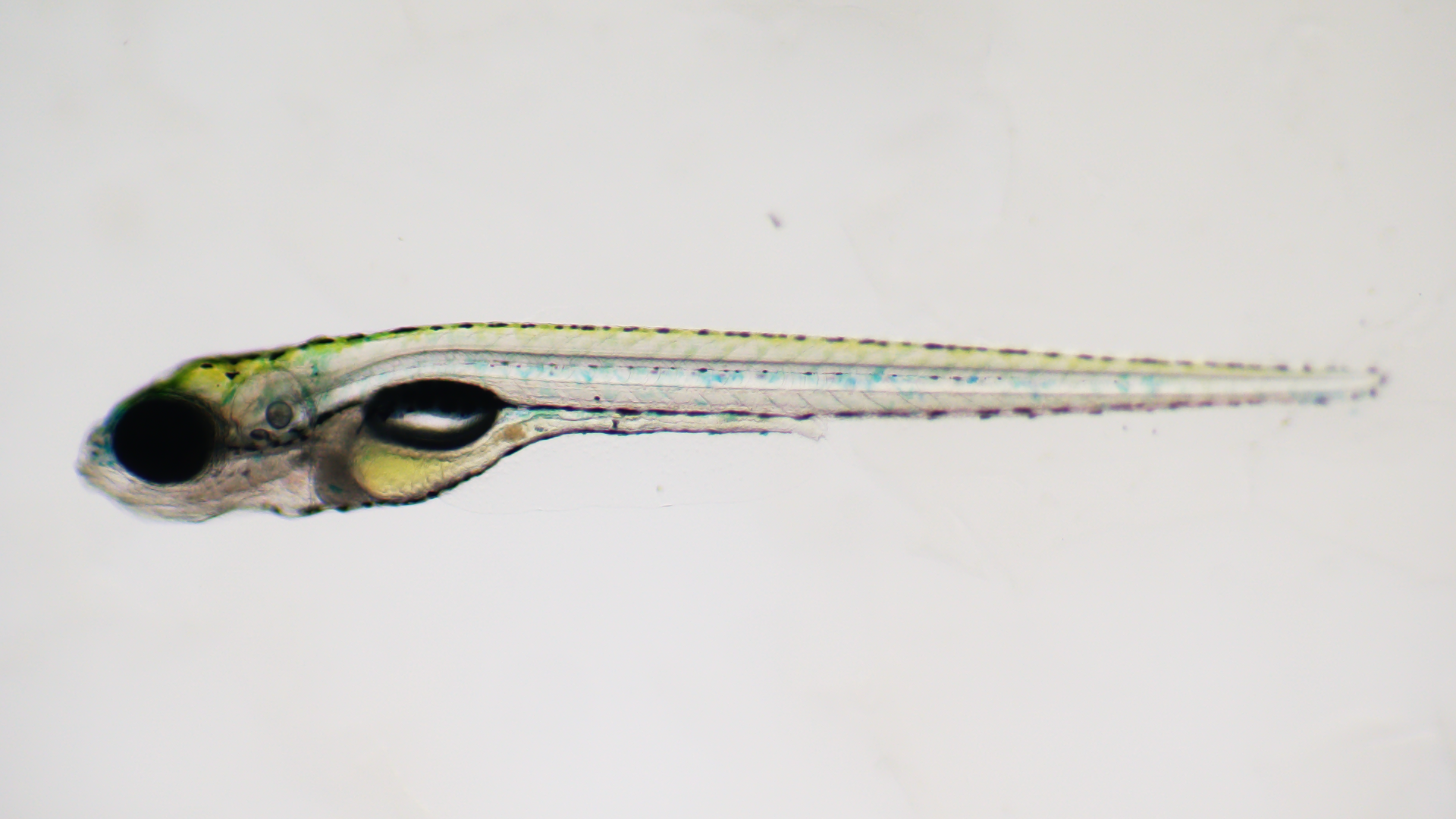

Supplement: Supplementary file 3 — Source data Fig. 3 [file 44321_2025_355_MOESM3_ESM.zip › Figure 3/3A/855VUS_8dpf.tif]

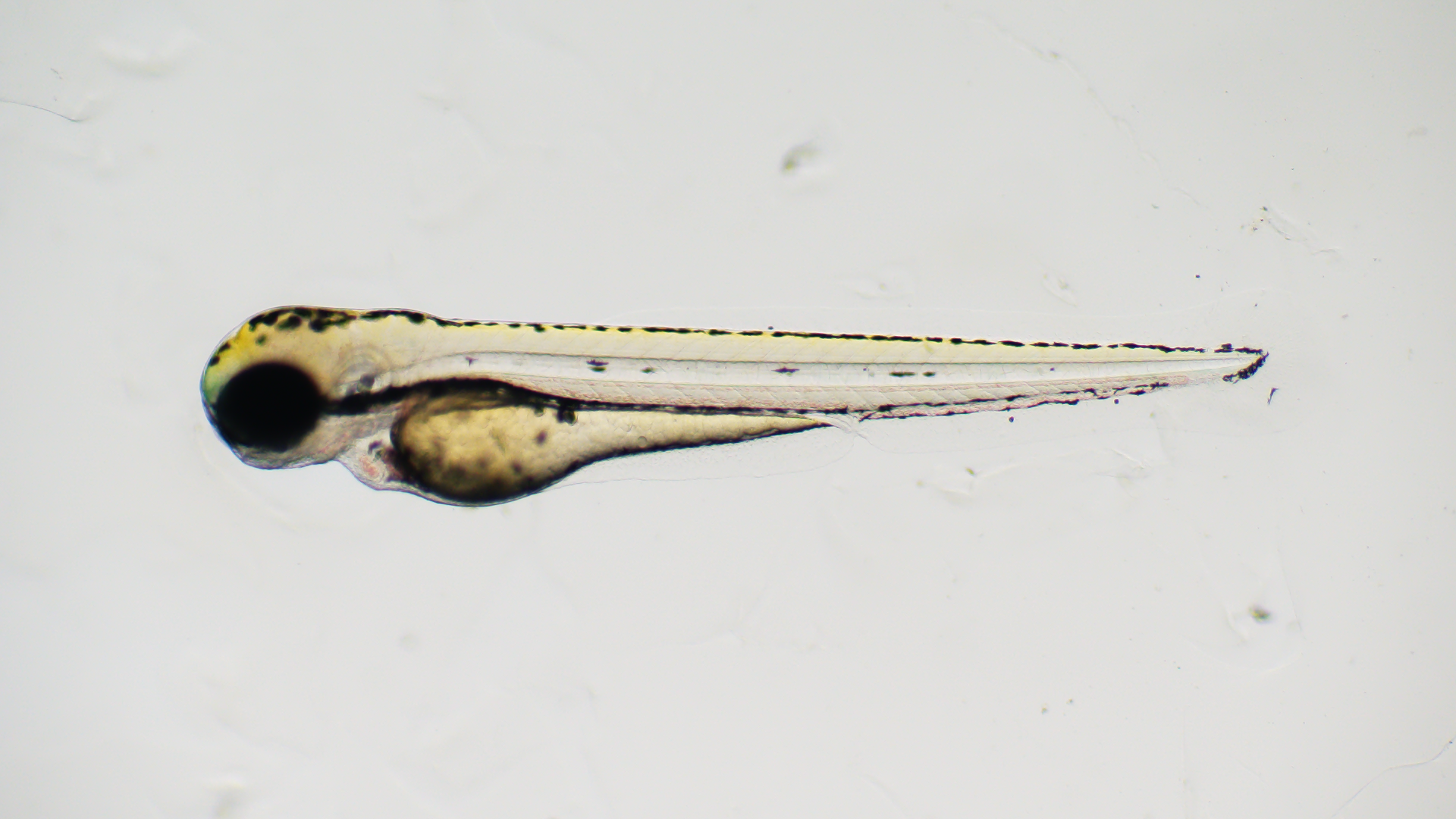

Supplement: Supplementary file 3 — Source data Fig. 3 [file 44321_2025_355_MOESM3_ESM.zip › Figure 3/3A/control_3dpf.tif]

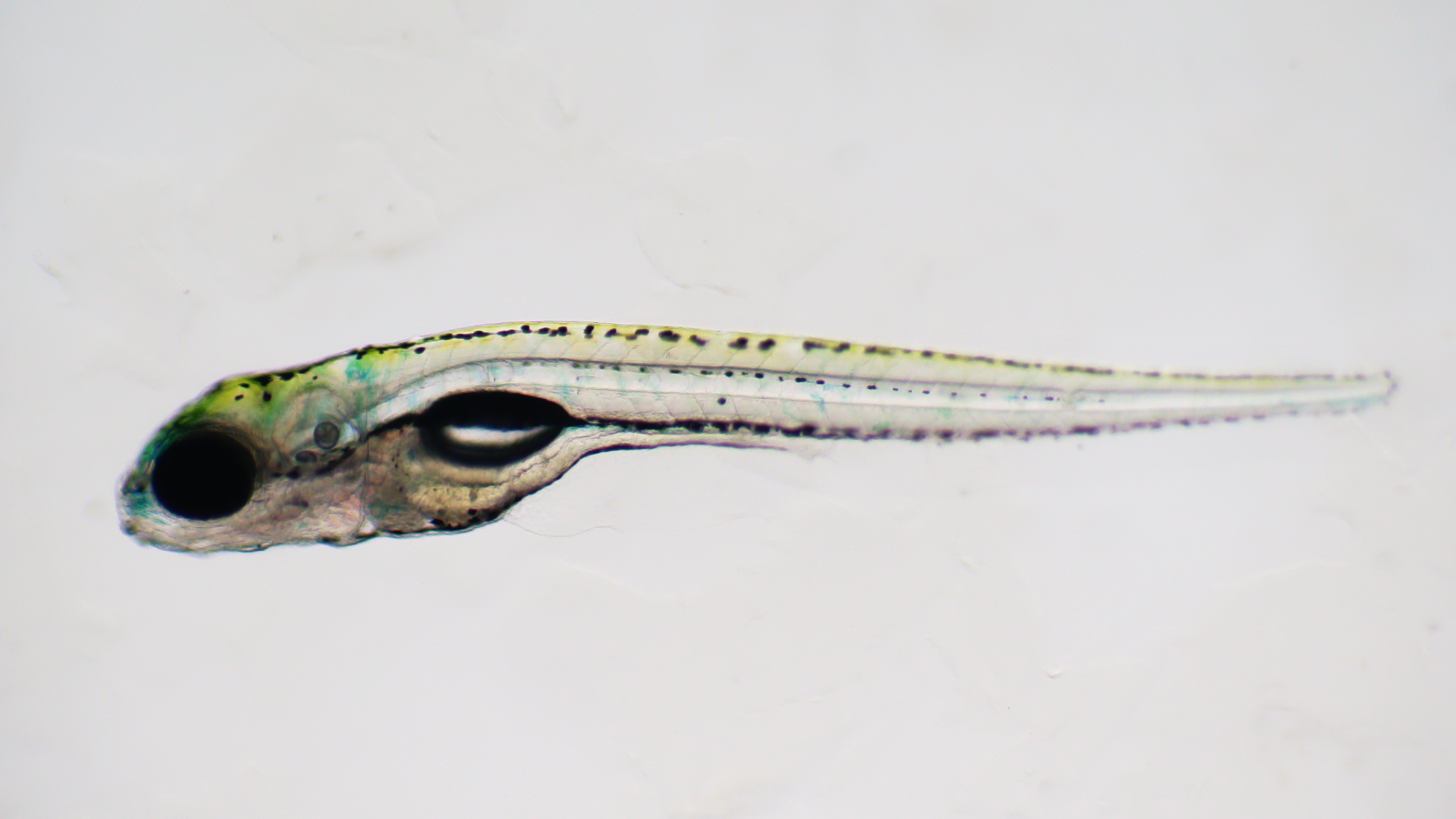

Supplement: Supplementary file 3 — Source data Fig. 3 [file 44321_2025_355_MOESM3_ESM.zip › Figure 3/3A/non-path_6dpf.tif]

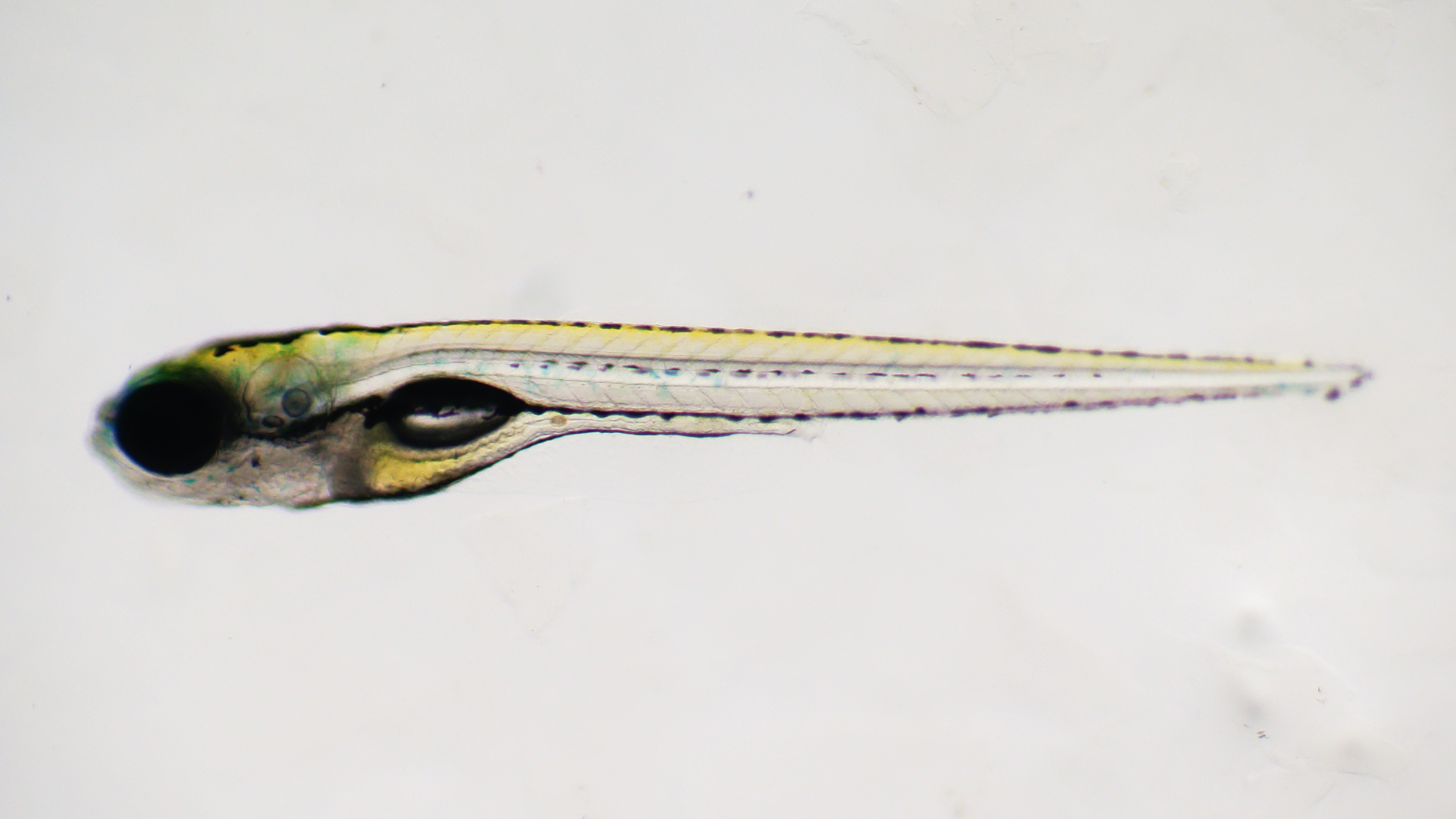

Supplement: Supplementary file 3 — Source data Fig. 3 [file 44321_2025_355_MOESM3_ESM.zip › Figure 3/3A/Tg(SMN1)_9dpf.tif]

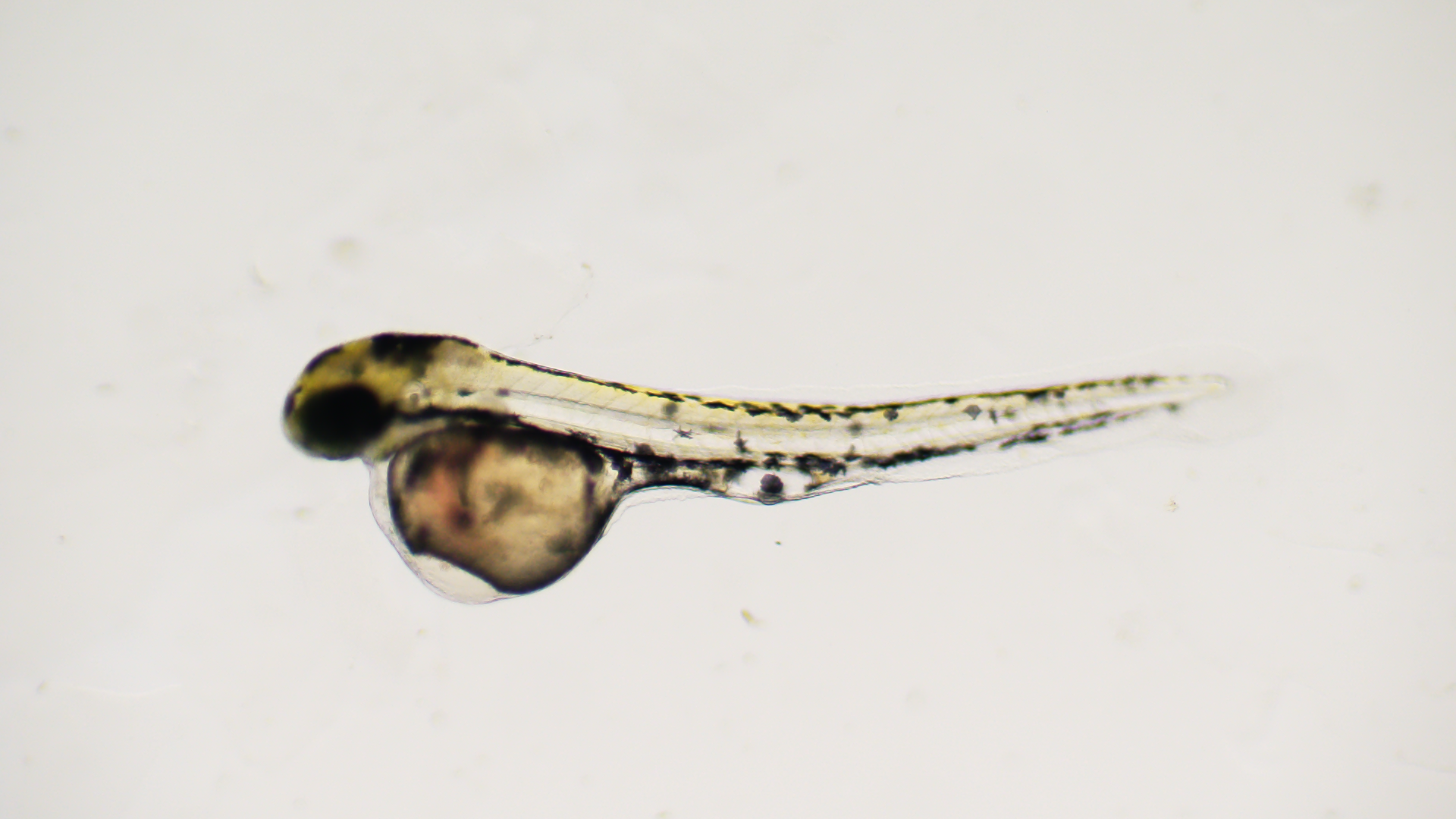

Supplement: Supplementary file 3 — Source data Fig. 3 [file 44321_2025_355_MOESM3_ESM.zip › Figure 3/3A/path_3dpf.tif]

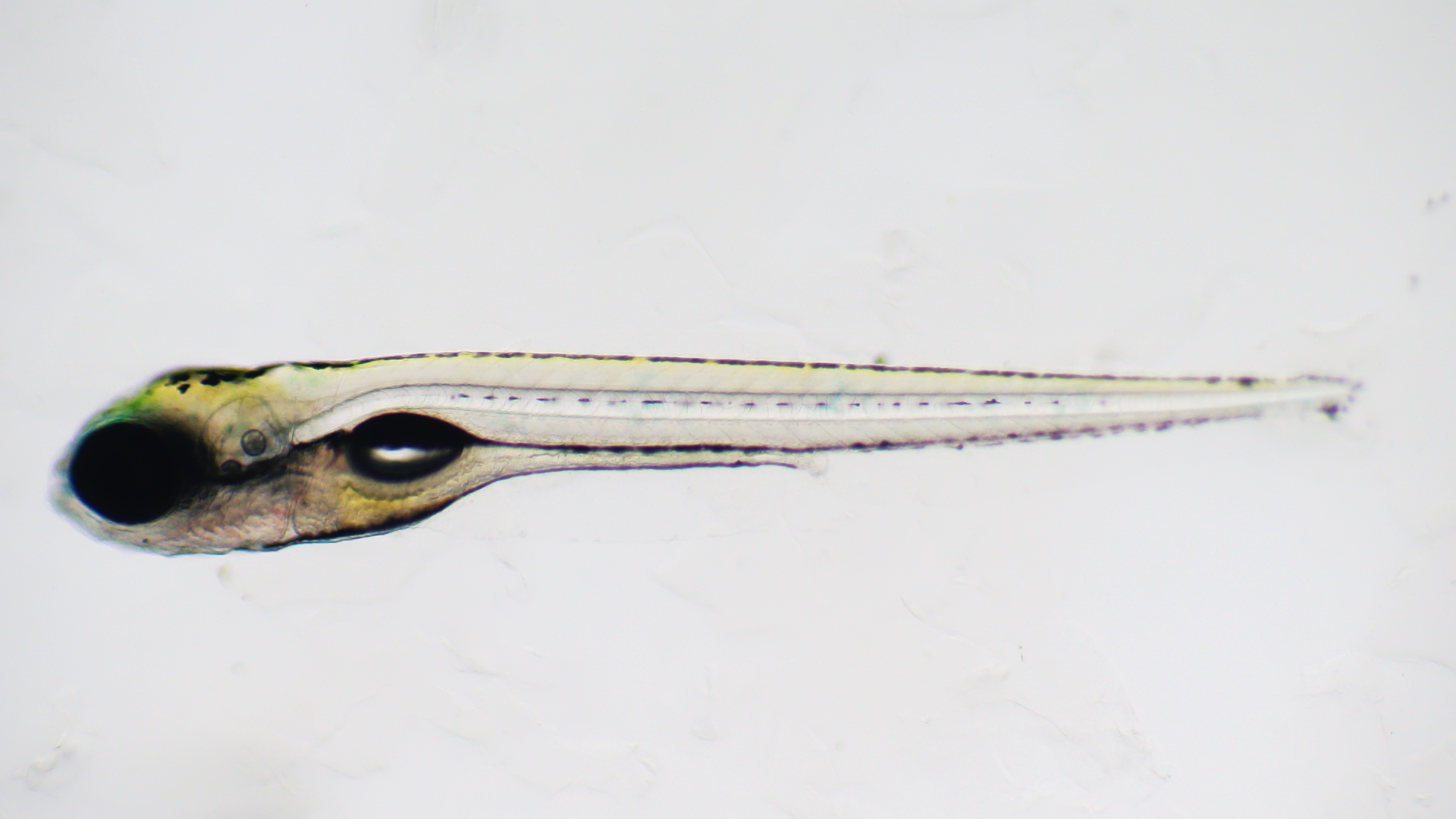

Supplement: Supplementary file 3 — Source data Fig. 3 [file 44321_2025_355_MOESM3_ESM.zip › Figure 3/3A/control_6dpf.tif]

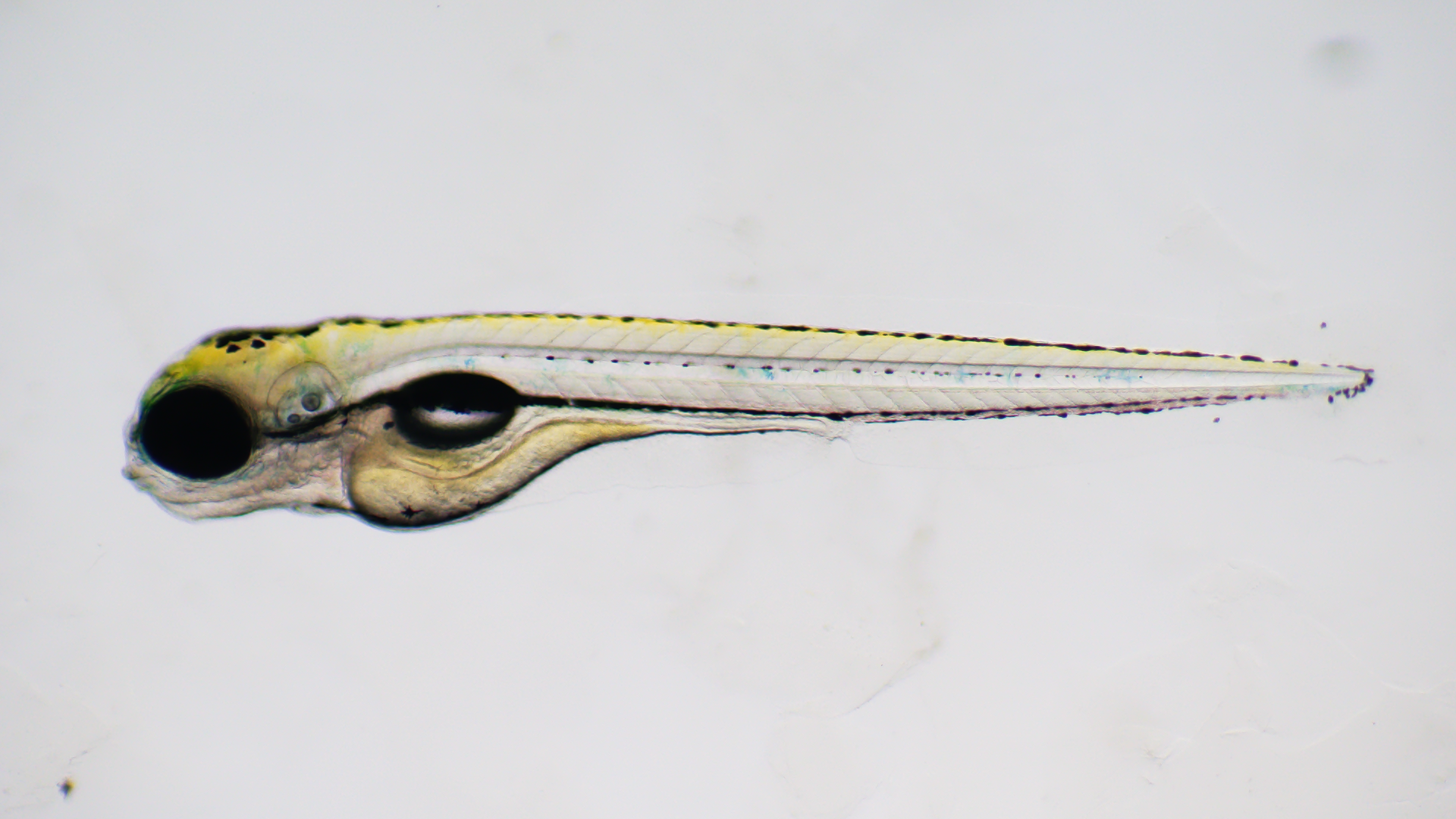

Supplement: Supplementary file 3 — Source data Fig. 3 [file 44321_2025_355_MOESM3_ESM.zip › Figure 3/3A/Tg(SMN1)_5dpf.tif]

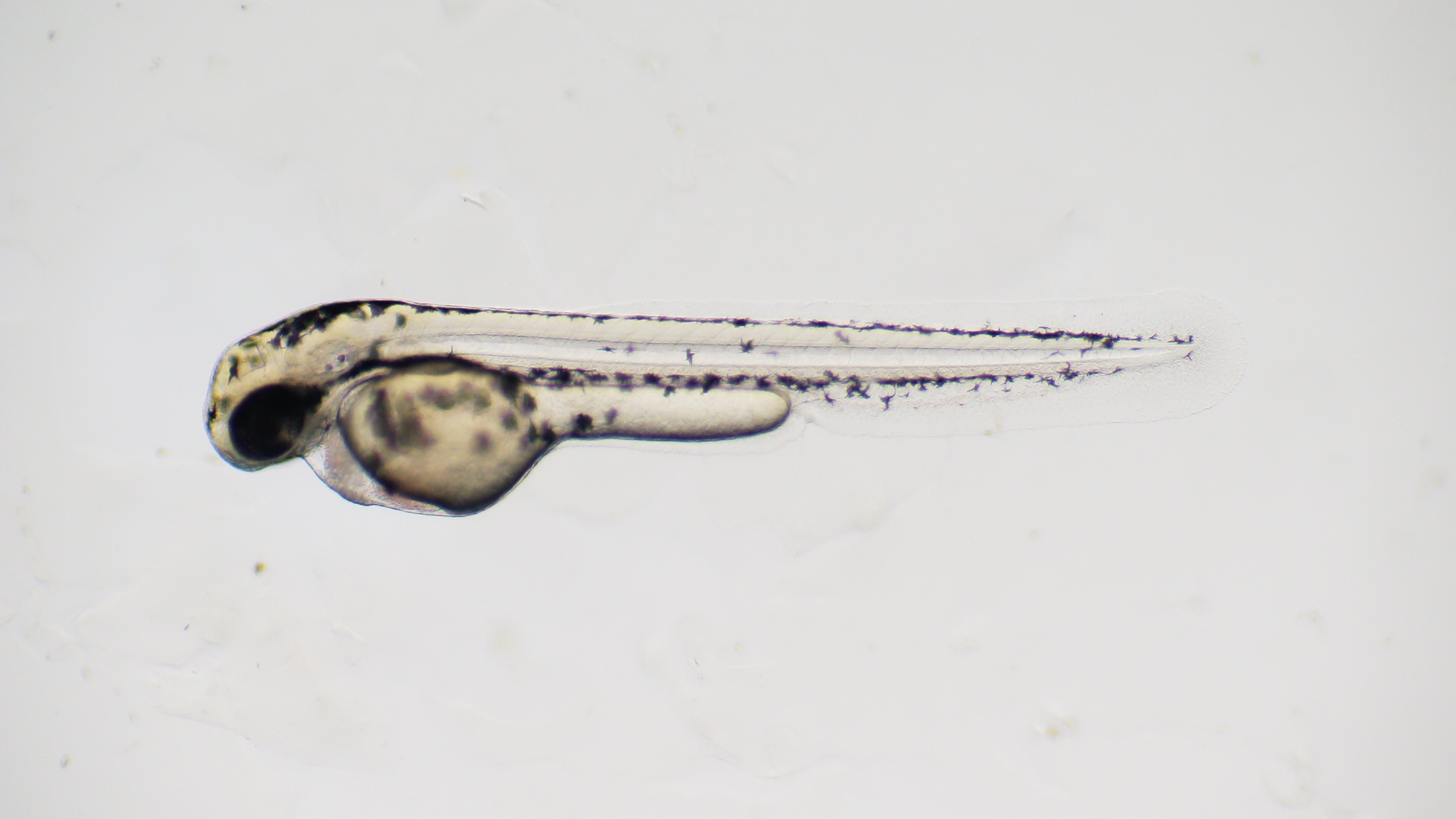

Supplement: Supplementary file 3 — Source data Fig. 3 [file 44321_2025_355_MOESM3_ESM.zip › Figure 3/3A/WT_2dpf.tif]

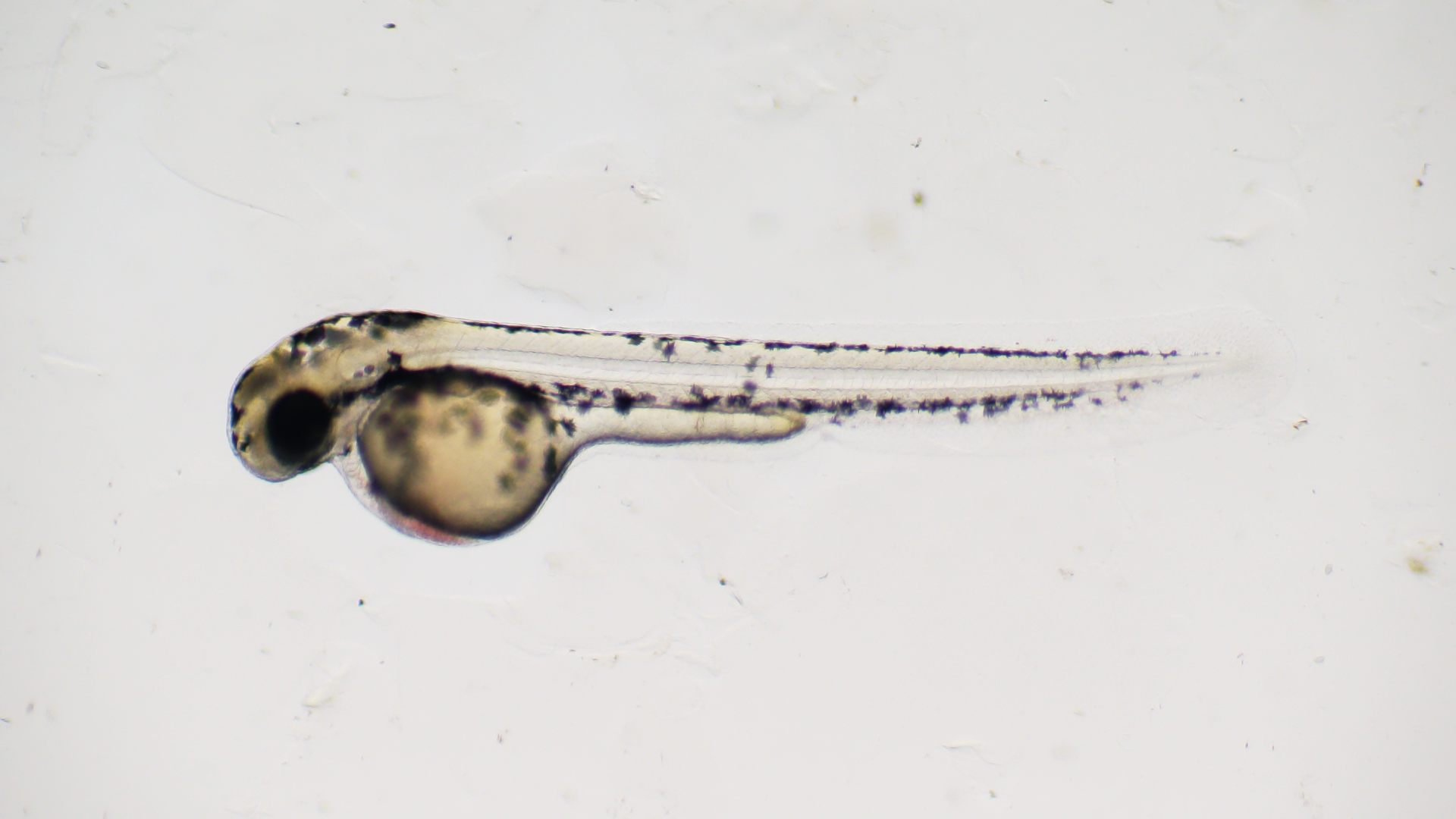

Supplement: Supplementary file 3 — Source data Fig. 3 [file 44321_2025_355_MOESM3_ESM.zip › Figure 3/3A/smn_null_2dpf.tif]

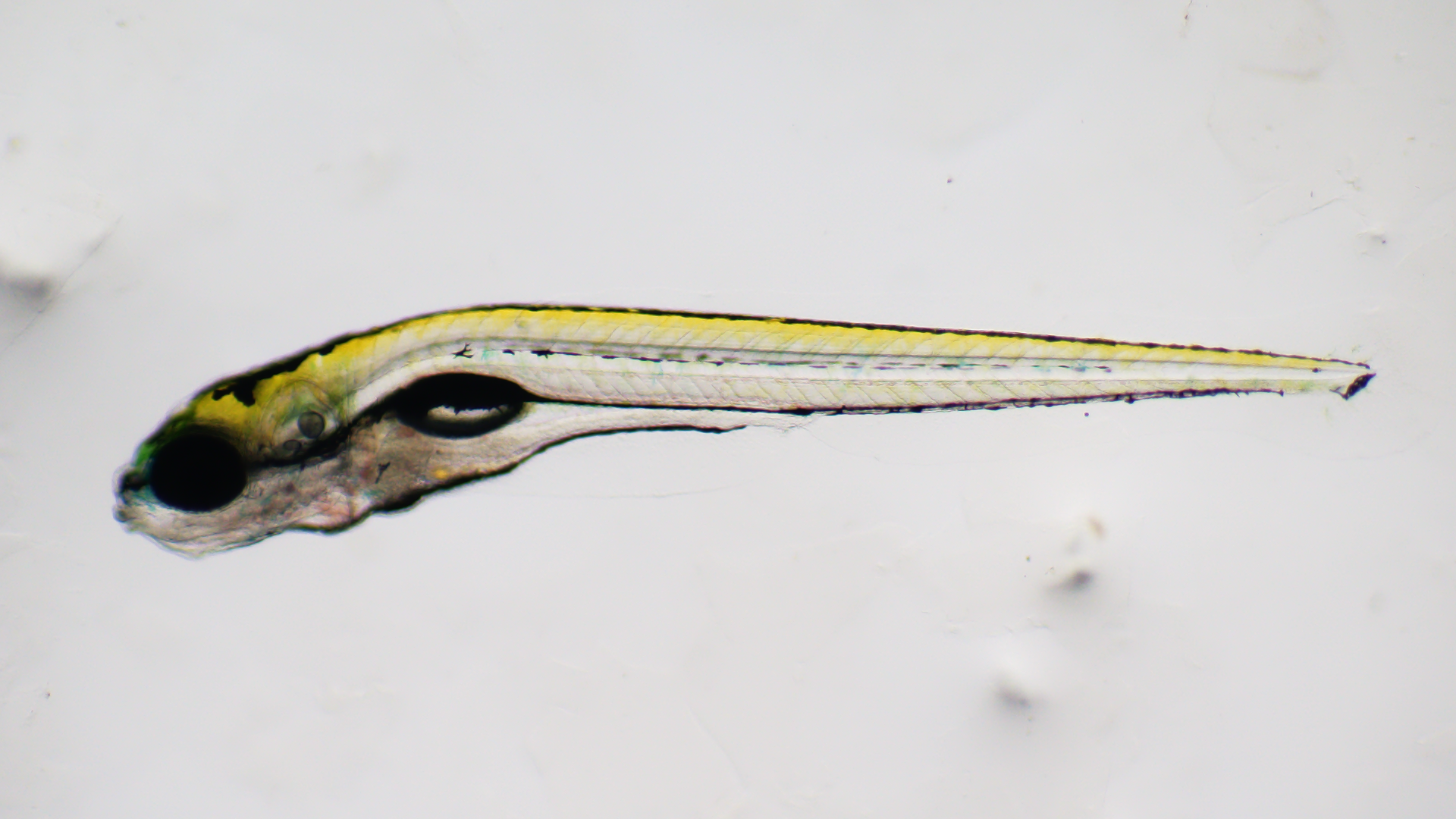

Supplement: Supplementary file 3 — Source data Fig. 3 [file 44321_2025_355_MOESM3_ESM.zip › Figure 3/3A/861VUS_7dpf.tif]

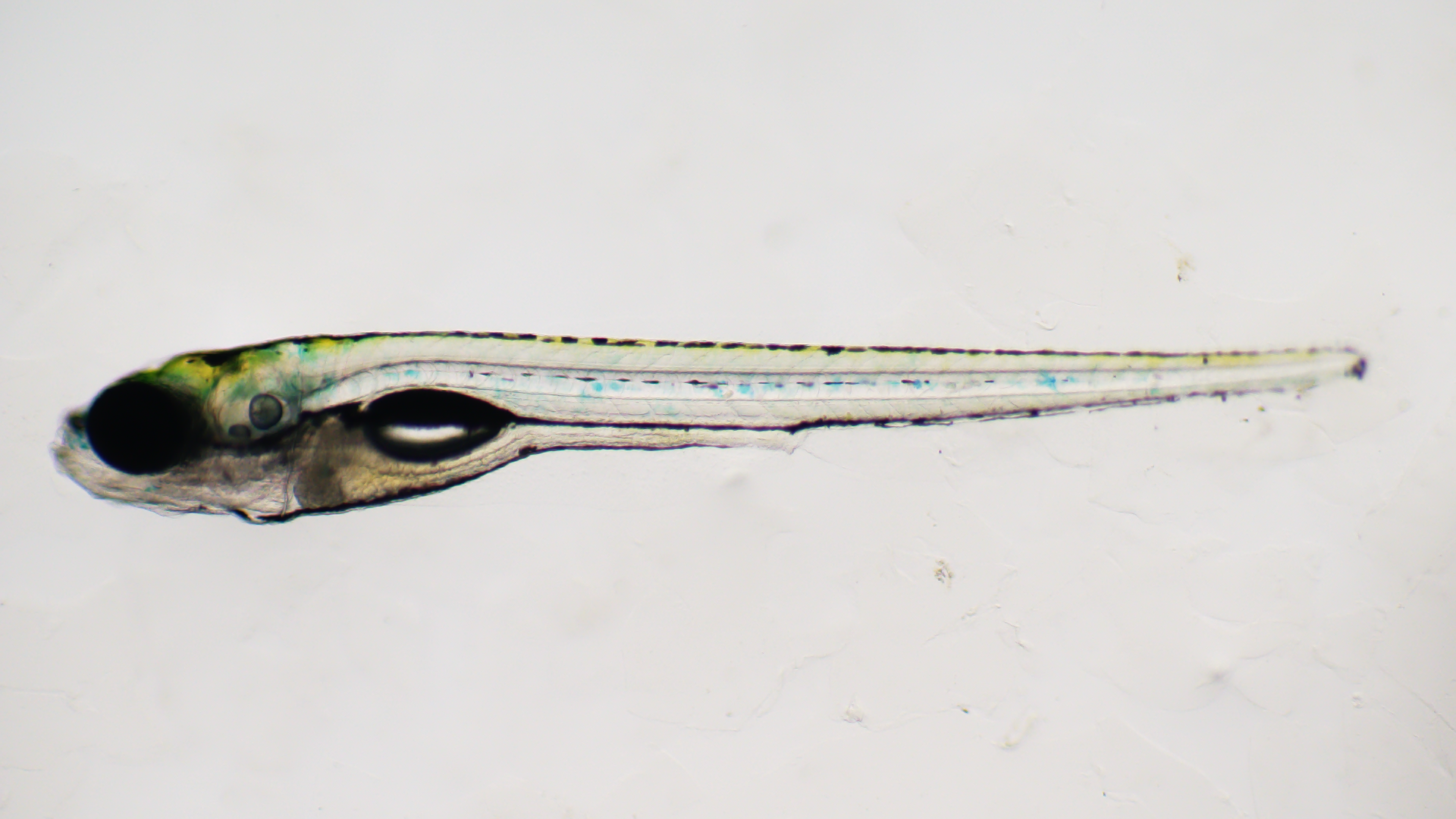

Supplement: Supplementary file 3 — Source data Fig. 3 [file 44321_2025_355_MOESM3_ESM.zip › Figure 3/3A/861VUS_10dpf.tif]

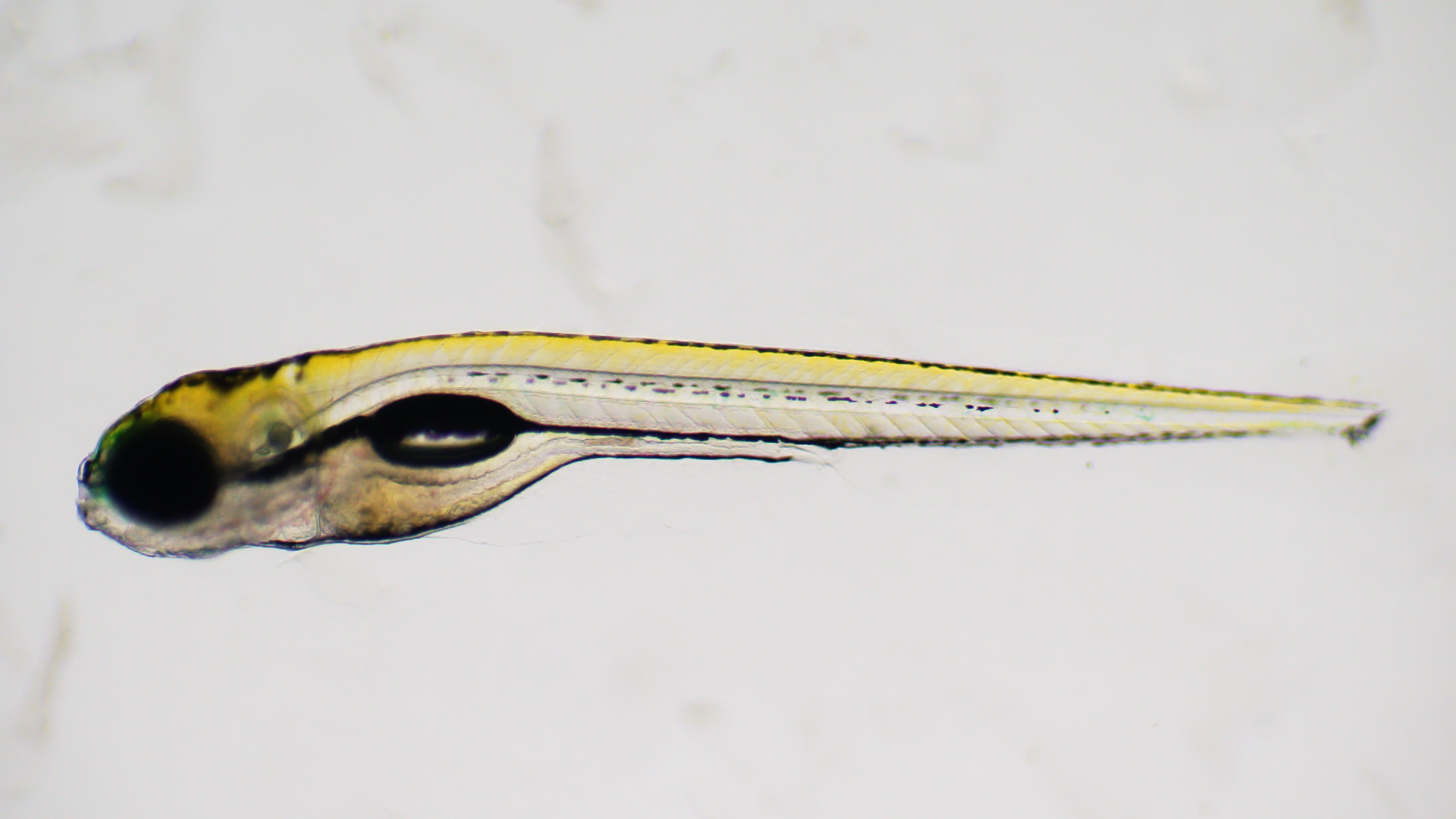

Supplement: Supplementary file 3 — Source data Fig. 3 [file 44321_2025_355_MOESM3_ESM.zip › Figure 3/3A/861VUS_5dpf.tif]

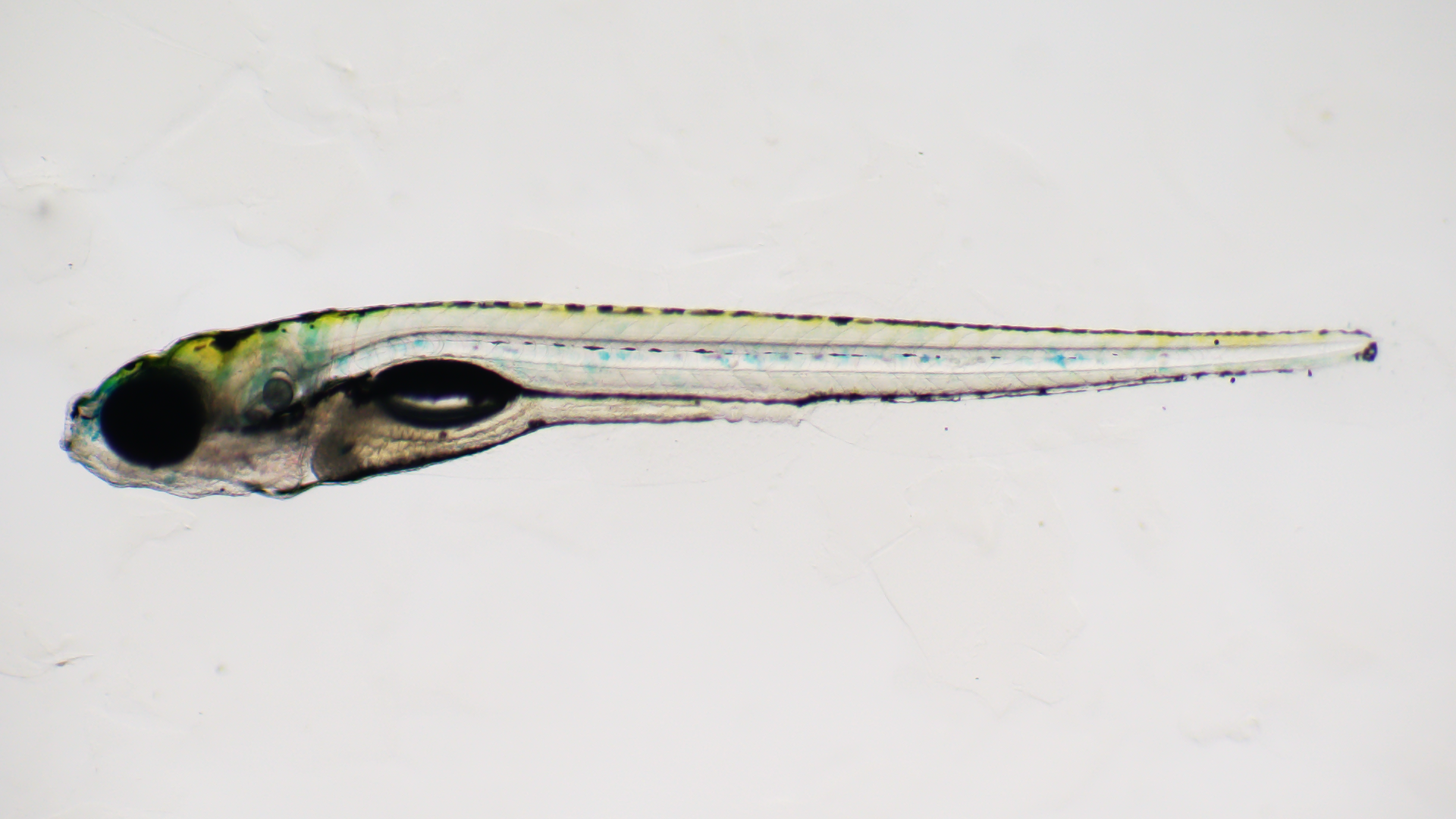

Supplement: Supplementary file 3 — Source data Fig. 3 [file 44321_2025_355_MOESM3_ESM.zip › Figure 3/3A/861VUS_9dpf.tif]

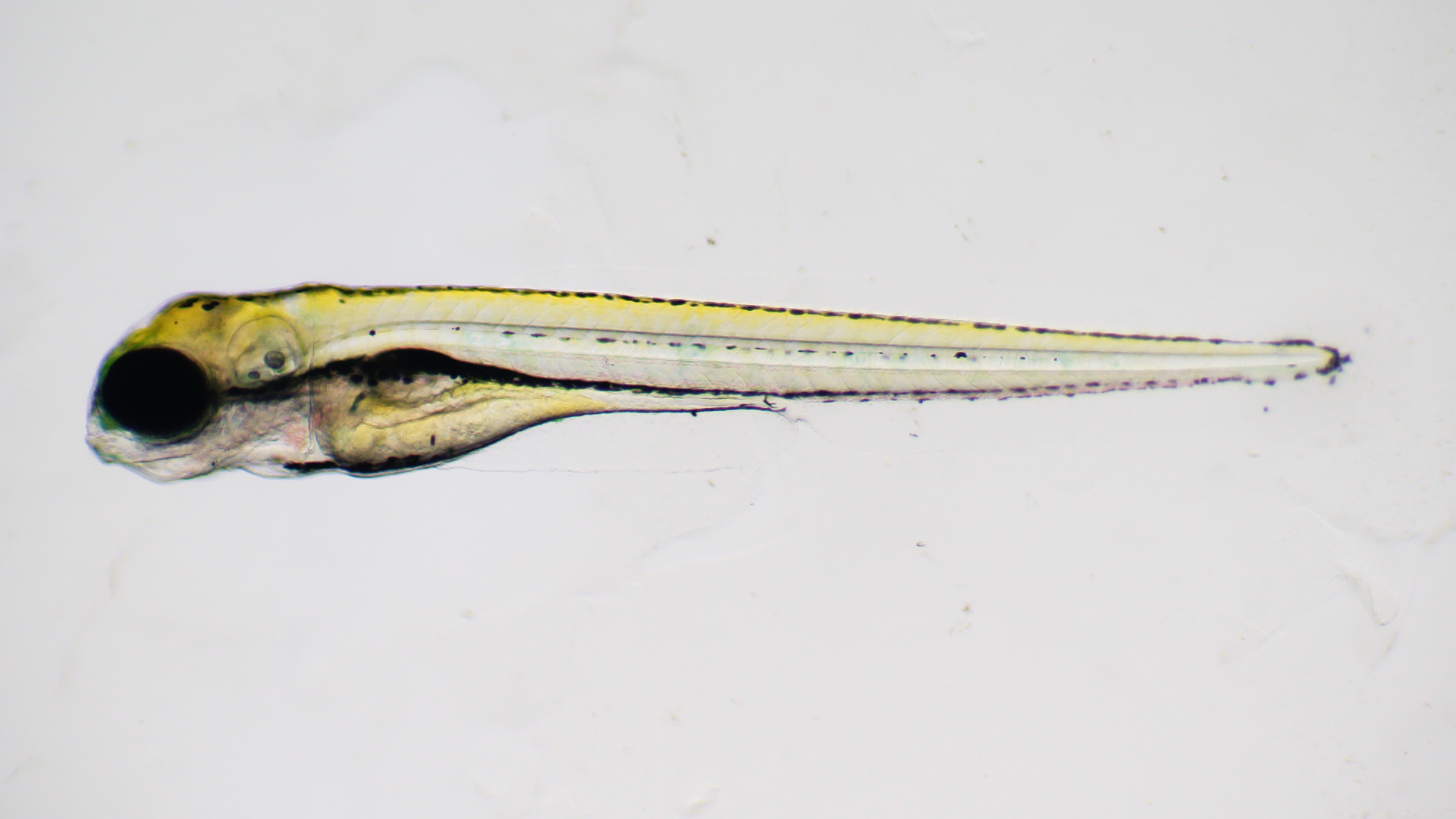

Supplement: Supplementary file 3 — Source data Fig. 3 [file 44321_2025_355_MOESM3_ESM.zip › Figure 3/3A/non-path_4dpf.tif]

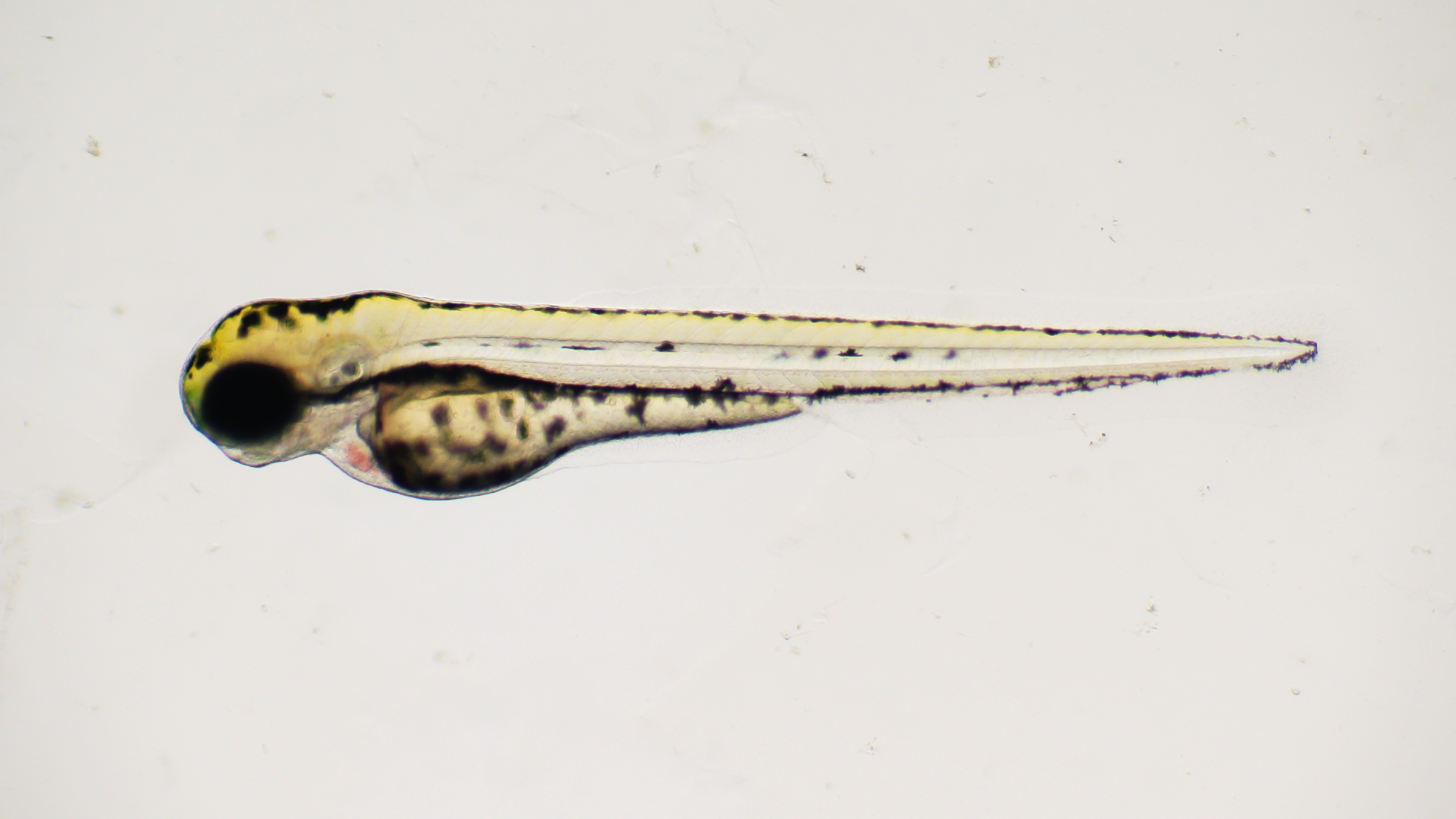

Supplement: Supplementary file 3 — Source data Fig. 3 [file 44321_2025_355_MOESM3_ESM.zip › Figure 3/3A/855VUS_3dpf.tif]

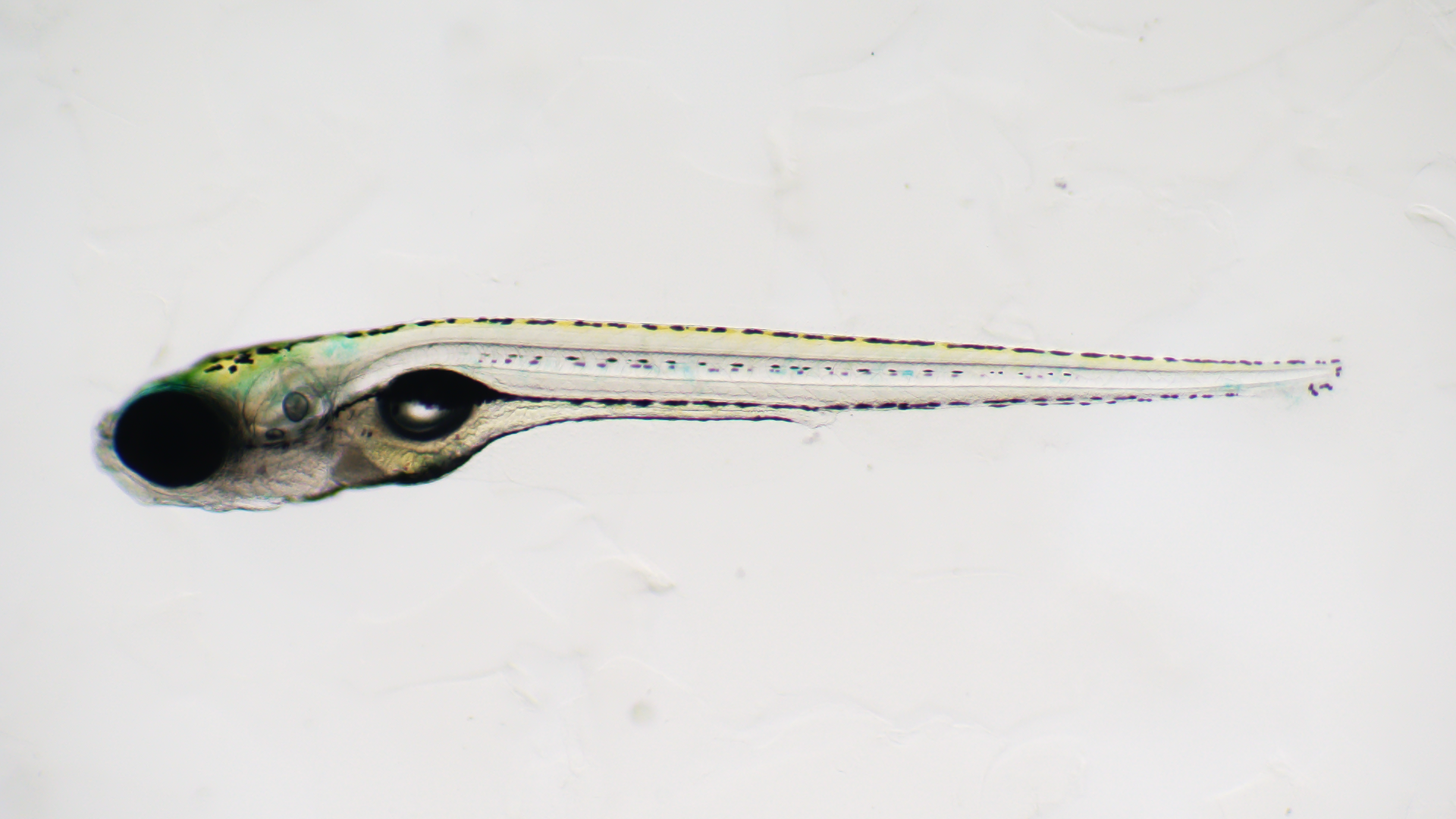

Supplement: Supplementary file 3 — Source data Fig. 3 [file 44321_2025_355_MOESM3_ESM.zip › Figure 3/3A/control_10dpf.tif]

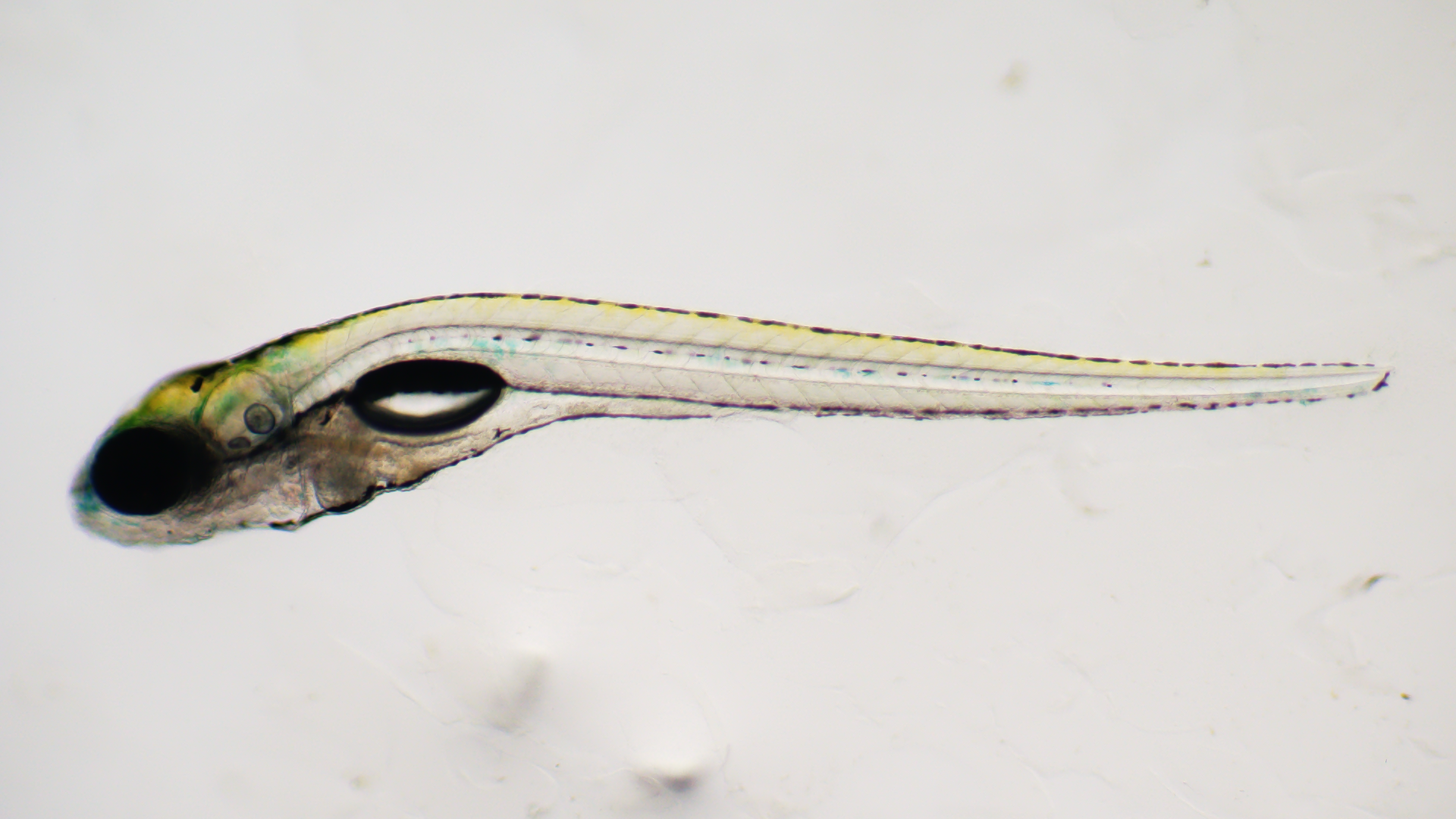

Supplement: Supplementary file 3 — Source data Fig. 3 [file 44321_2025_355_MOESM3_ESM.zip › Figure 3/3A/non-path_8dpf.tif]

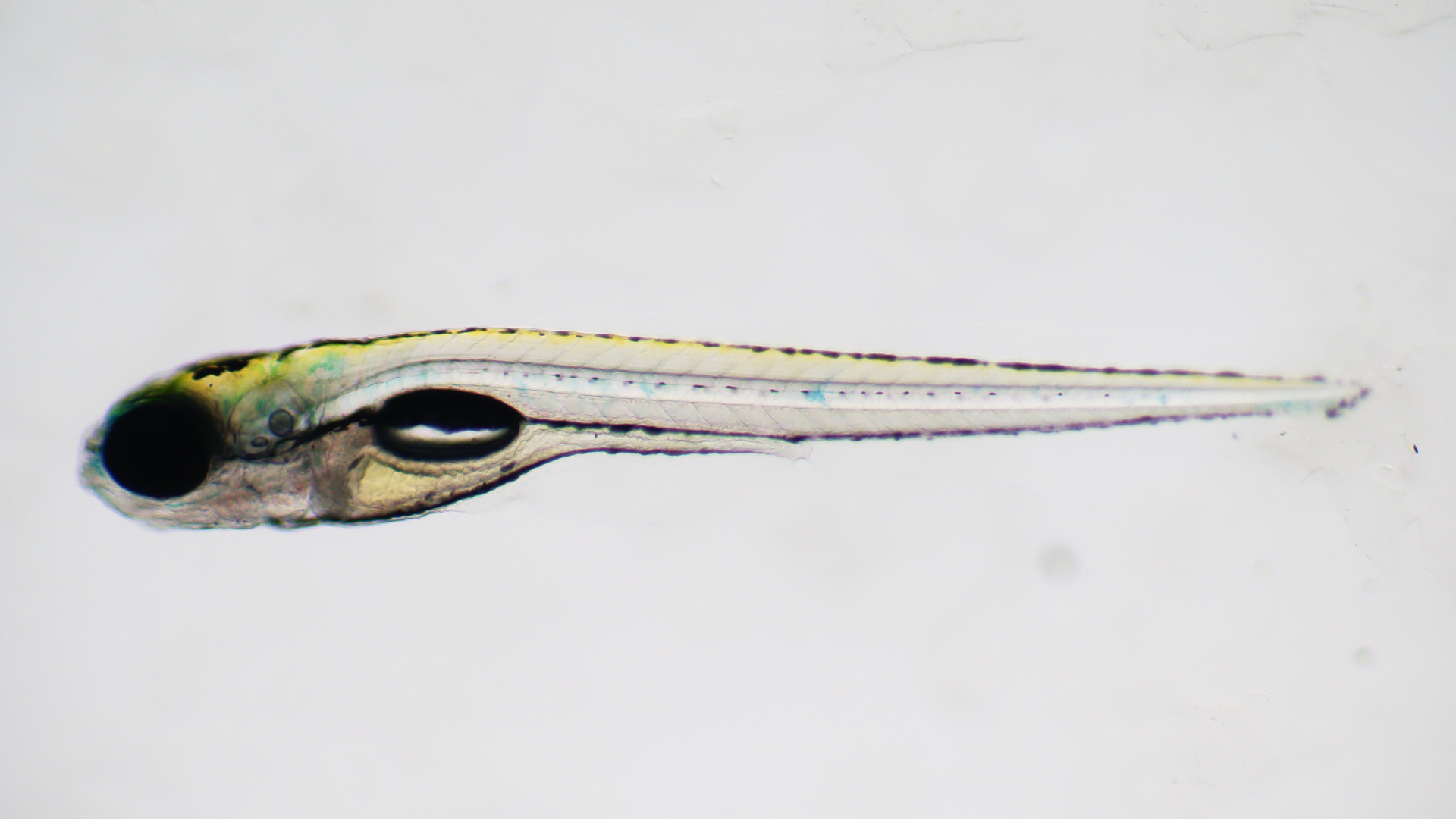

Supplement: Supplementary file 3 — Source data Fig. 3 [file 44321_2025_355_MOESM3_ESM.zip › Figure 3/3A/Tg(SMN1)_7dpf.tif]

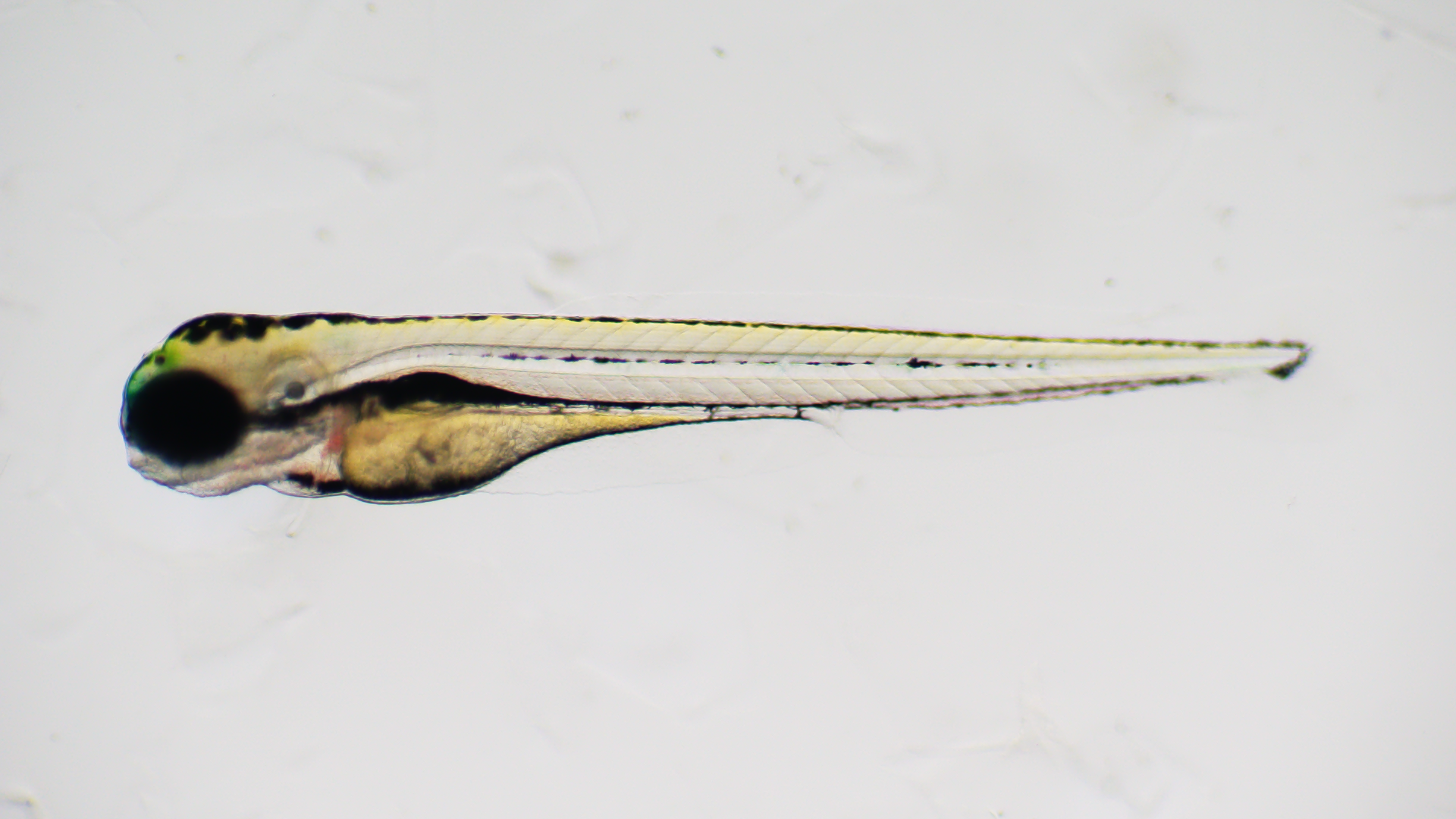

Supplement: Supplementary file 3 — Source data Fig. 3 [file 44321_2025_355_MOESM3_ESM.zip › Figure 3/3A/control_4dpf.tif]

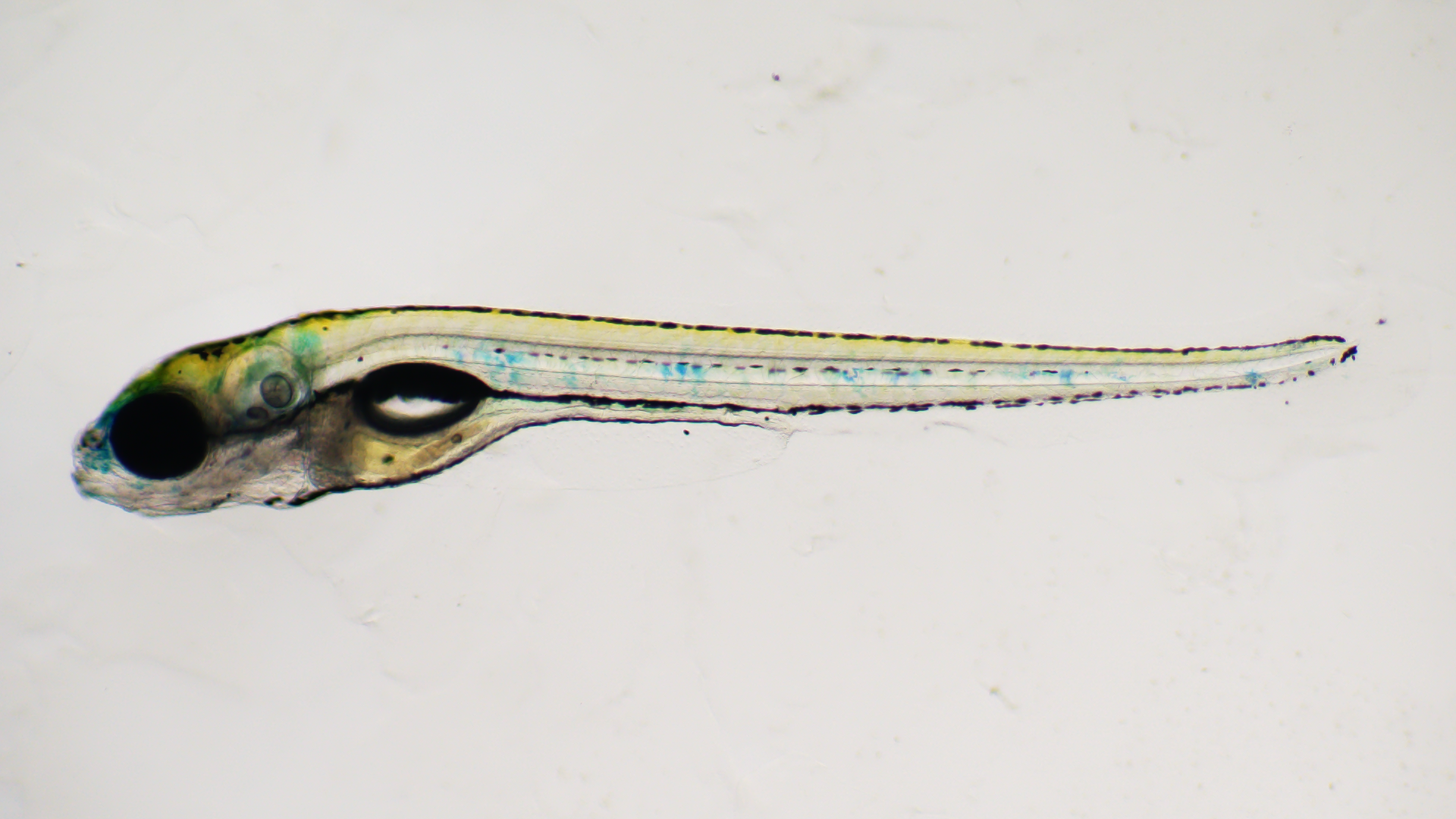

Supplement: Supplementary file 3 — Source data Fig. 3 [file 44321_2025_355_MOESM3_ESM.zip › Figure 3/3A/non-path_10dpf.tif]

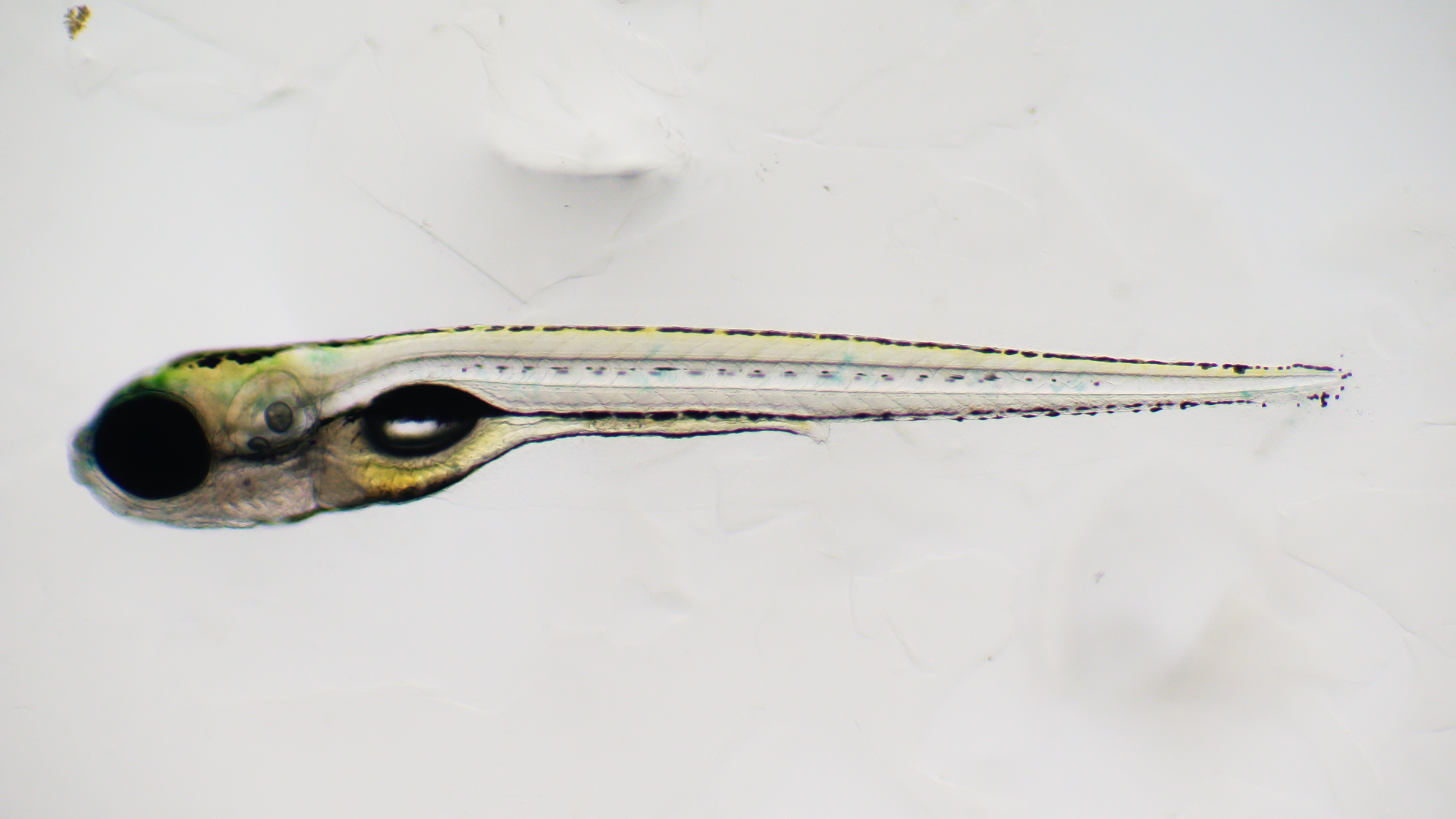

Supplement: Supplementary file 3 — Source data Fig. 3 [file 44321_2025_355_MOESM3_ESM.zip › Figure 3/3A/control_8dpf.tif]

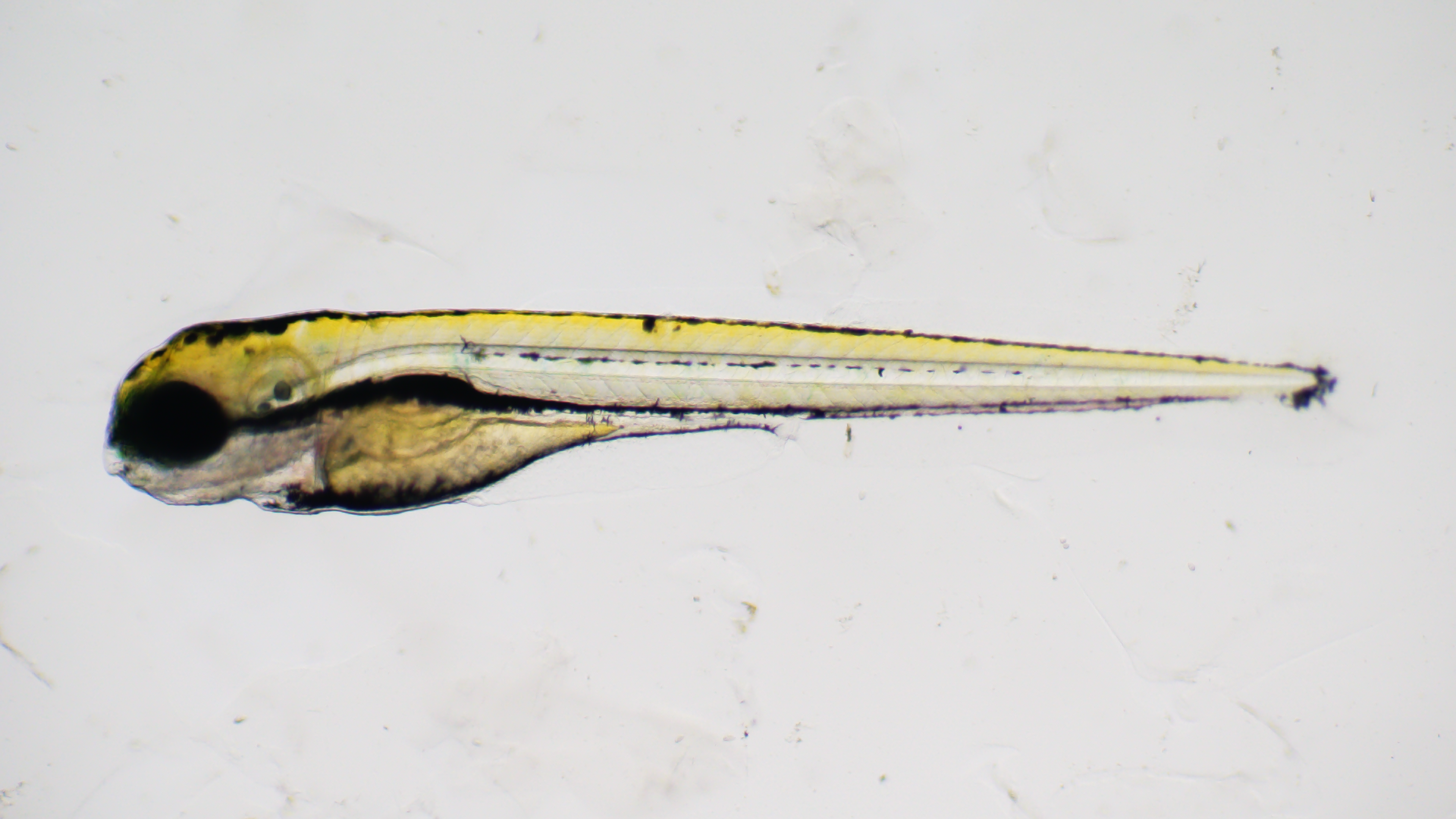

Supplement: Supplementary file 3 — Source data Fig. 3 [file 44321_2025_355_MOESM3_ESM.zip › Figure 3/3A/WT_4dpf.tif]

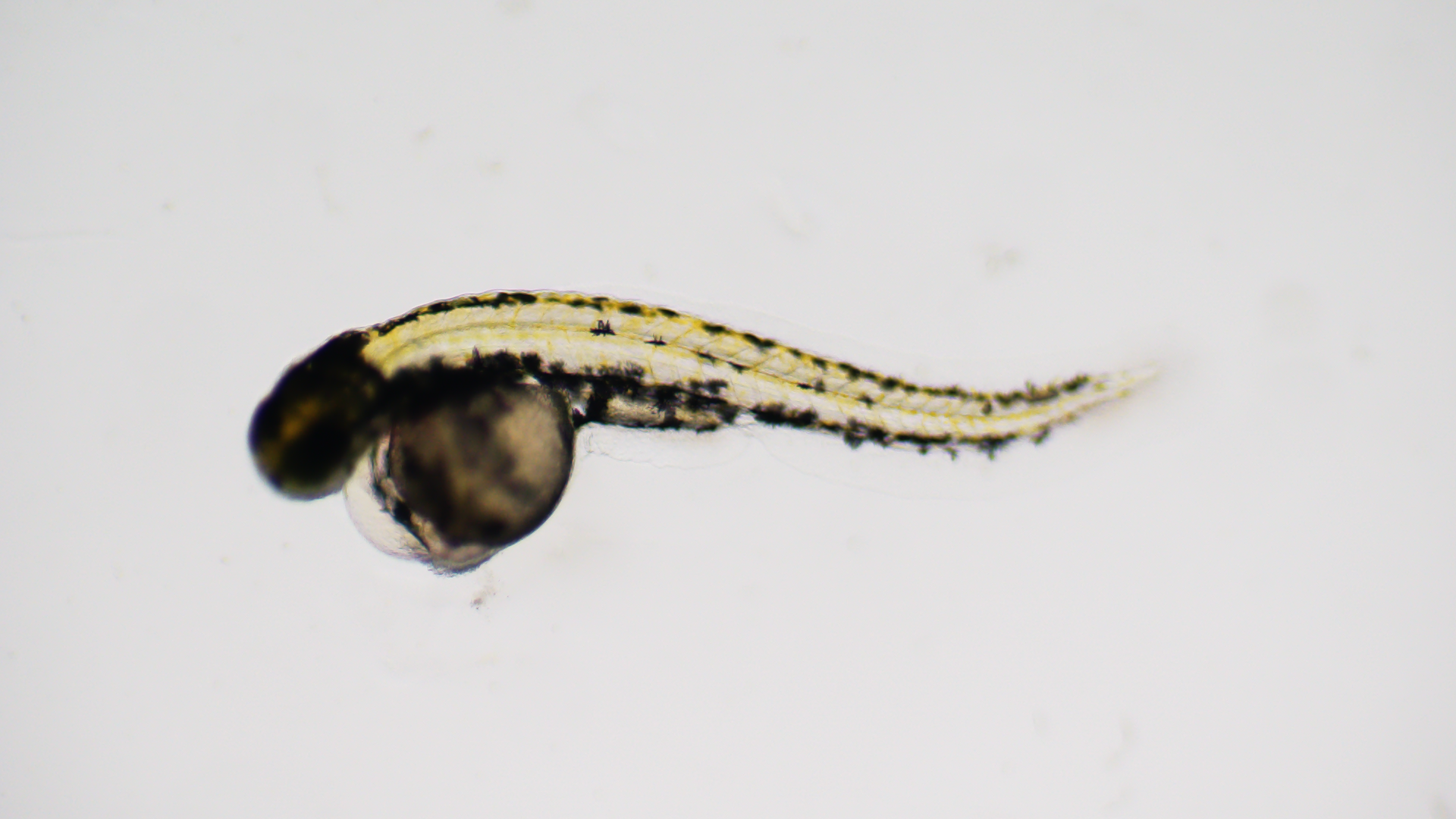

Supplement: Supplementary file 3 — Source data Fig. 3 [file 44321_2025_355_MOESM3_ESM.zip › Figure 3/3A/smn_null_4dpf.tif]

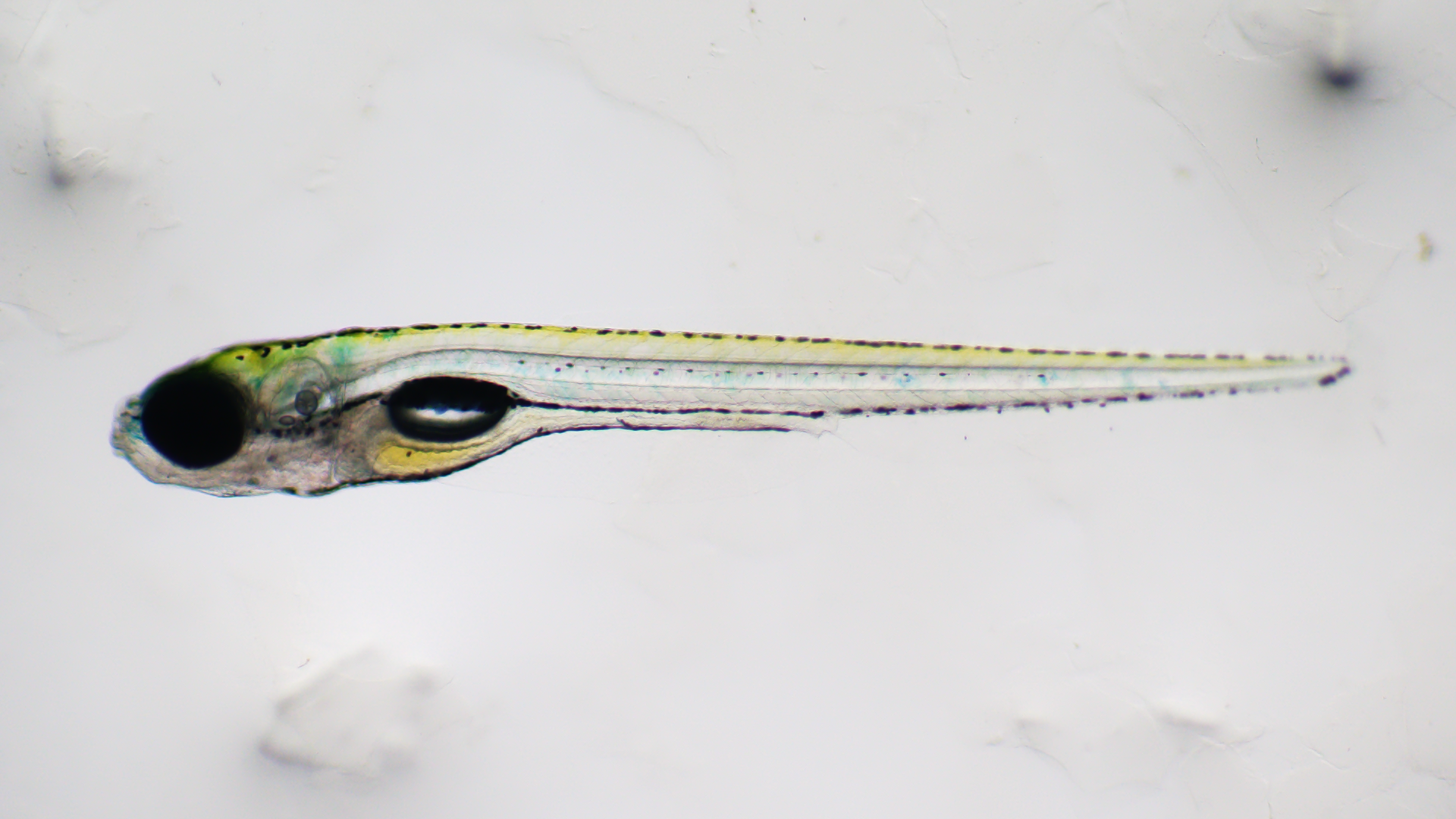

Supplement: Supplementary file 3 — Source data Fig. 3 [file 44321_2025_355_MOESM3_ESM.zip › Figure 3/3A/WT_8dpf.tif]

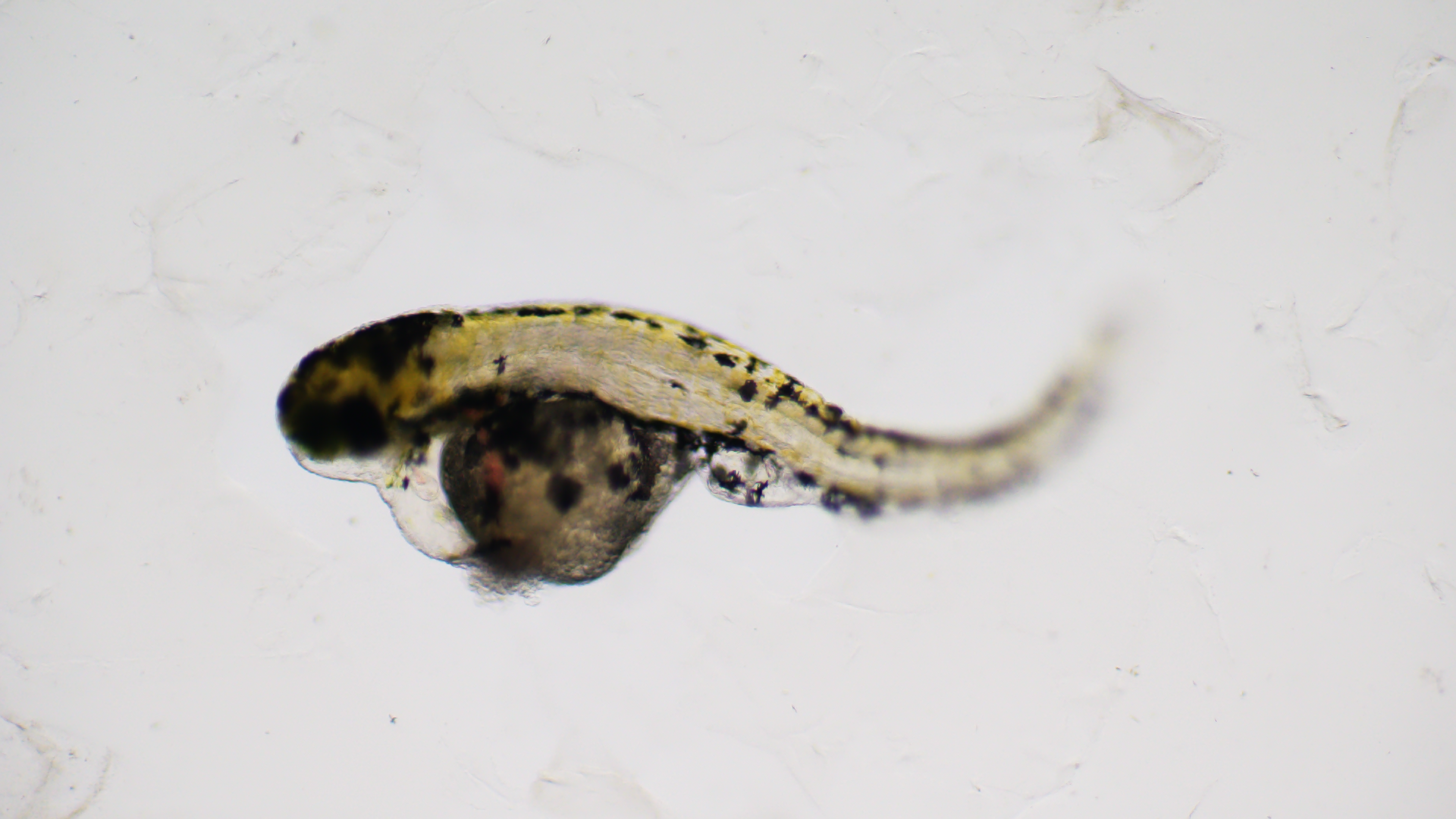

Supplement: Supplementary file 3 — Source data Fig. 3 [file 44321_2025_355_MOESM3_ESM.zip › Figure 3/3A/path_5dpf.tif]

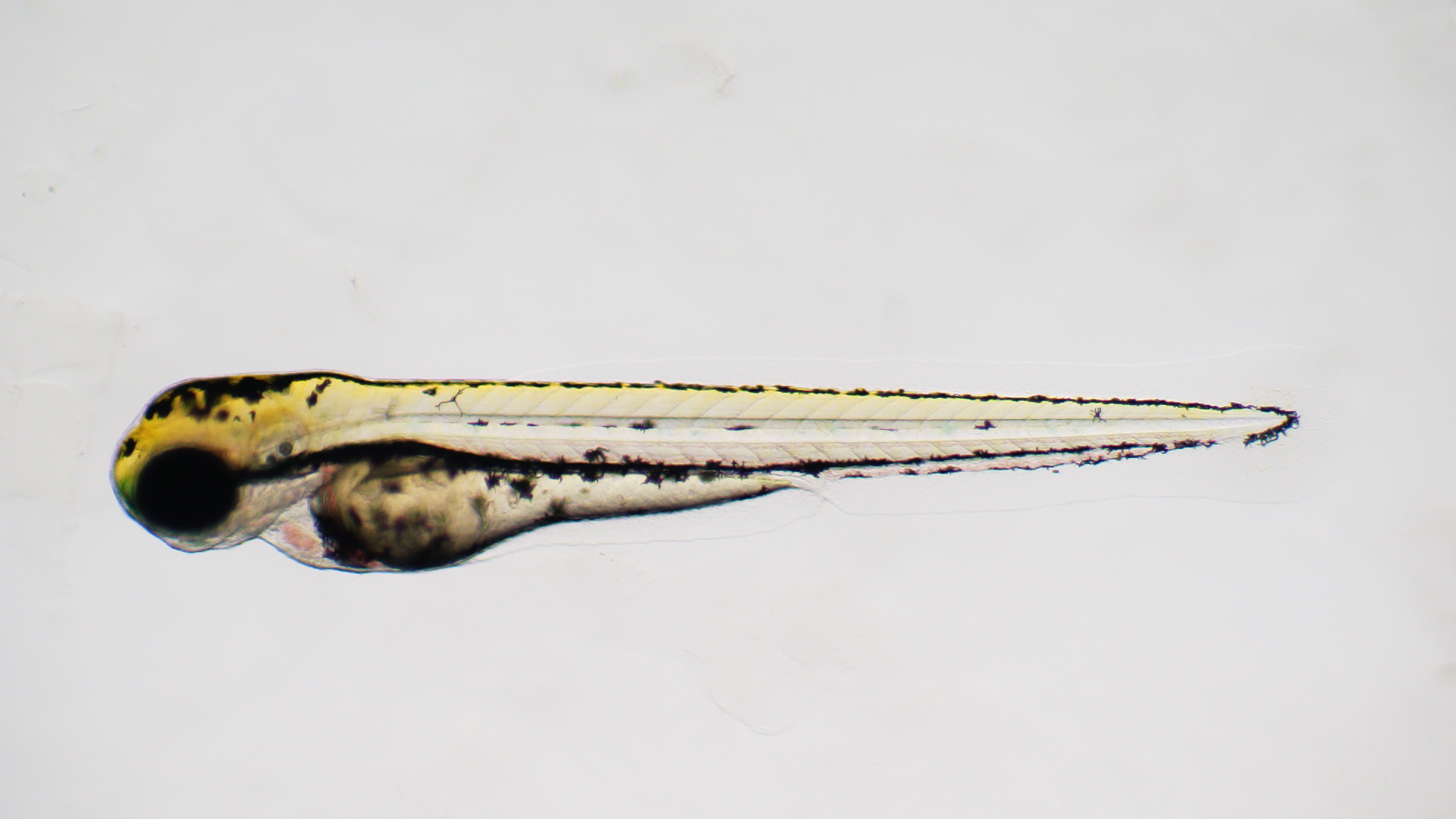

Supplement: Supplementary file 3 — Source data Fig. 3 [file 44321_2025_355_MOESM3_ESM.zip › Figure 3/3A/Tg(SMN1)_3dpf.tif]

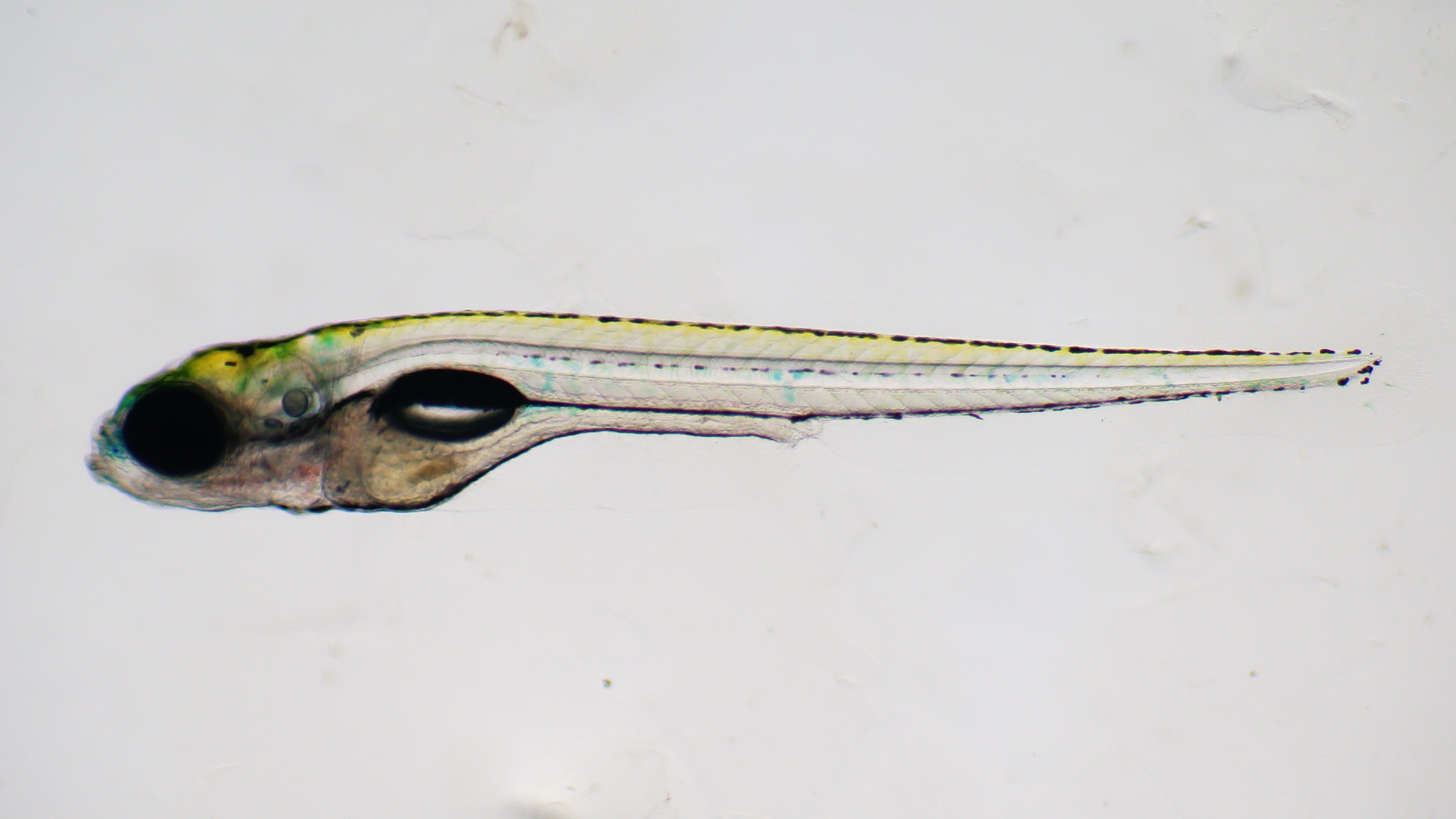

Supplement: Supplementary file 3 — Source data Fig. 3 [file 44321_2025_355_MOESM3_ESM.zip › Figure 3/3A/855VUS_7dpf.tif]

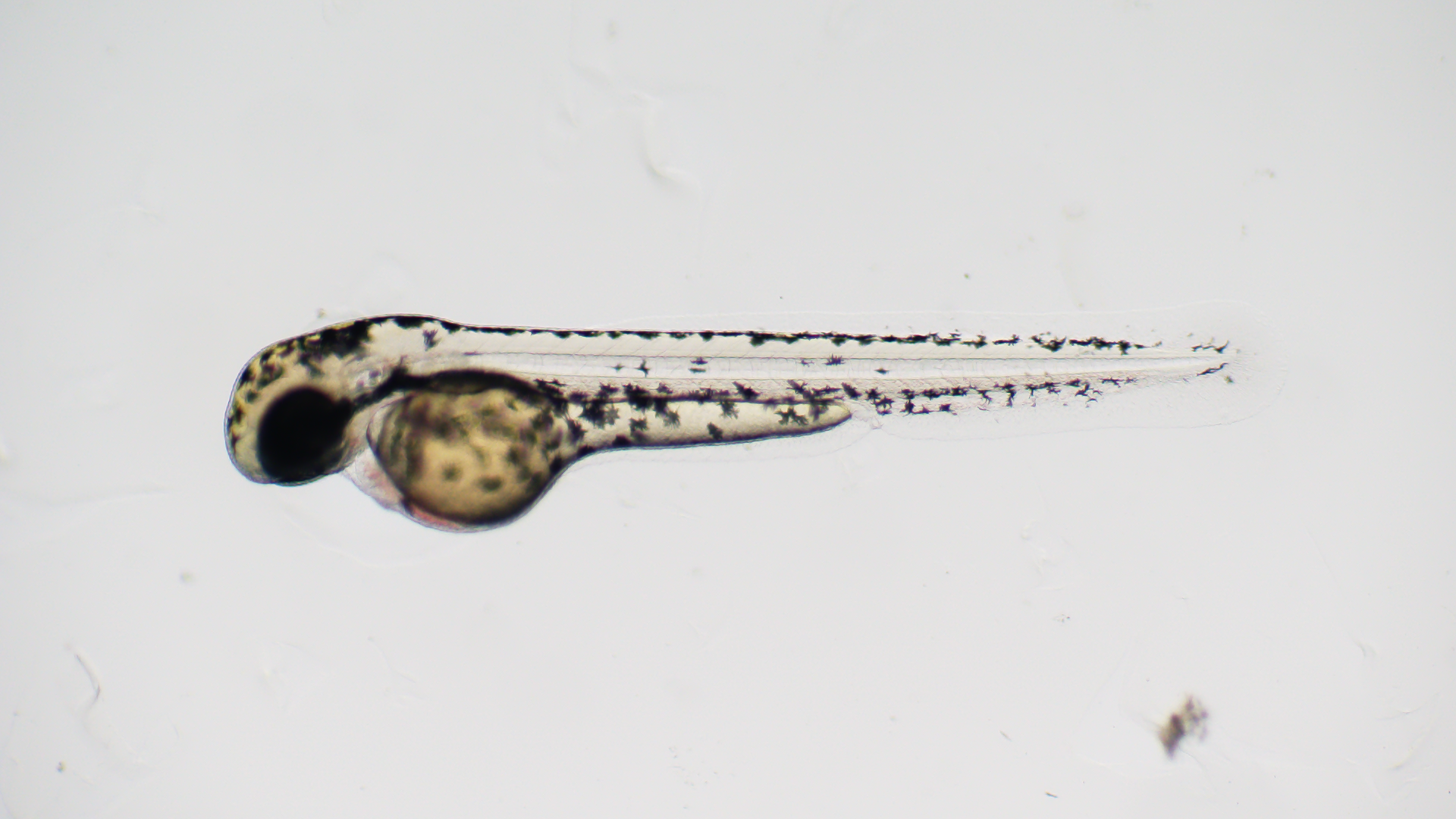

Supplement: Supplementary file 3 — Source data Fig. 3 [file 44321_2025_355_MOESM3_ESM.zip › Figure 3/3A/control_2dpf.tif]

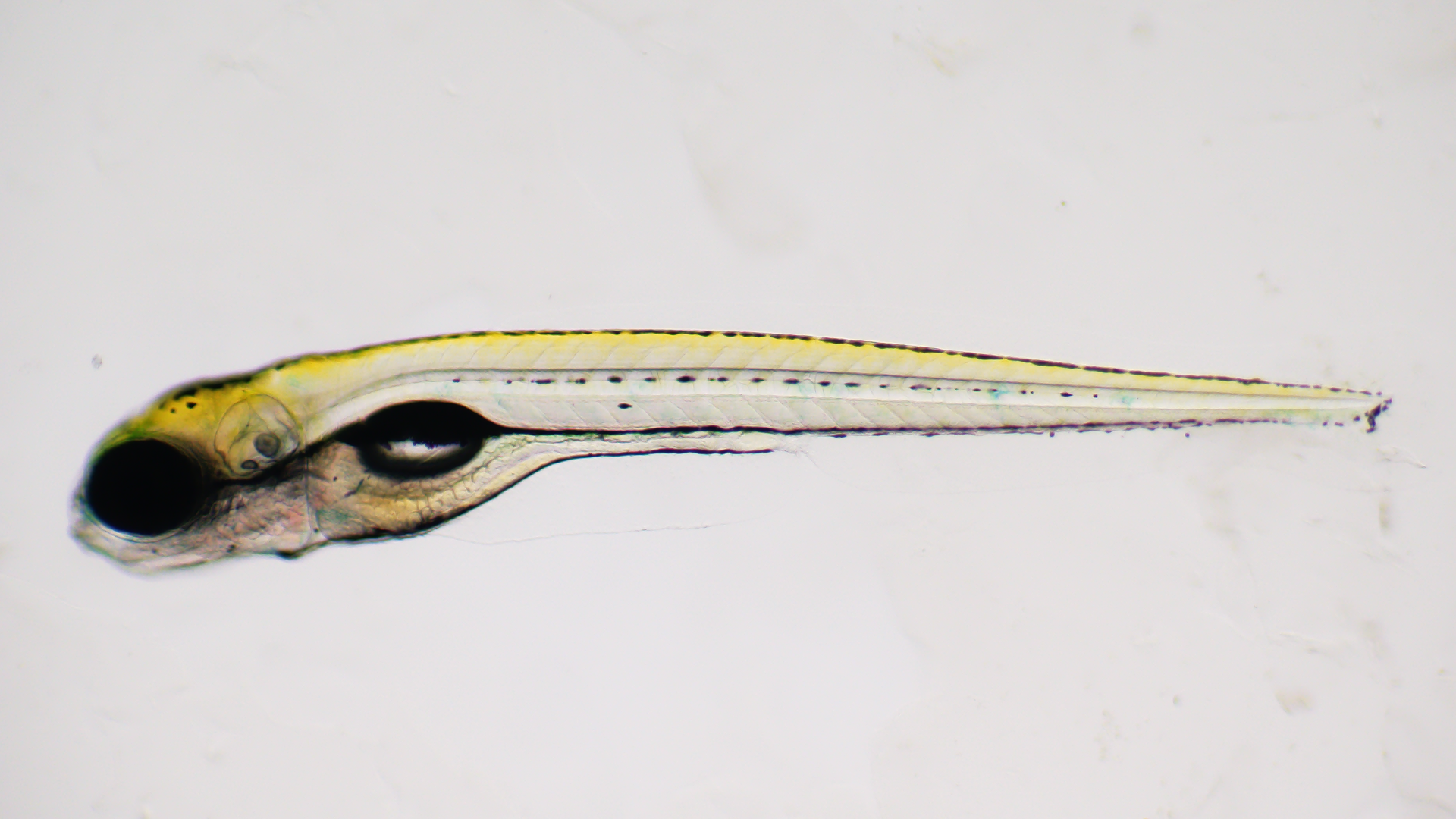

Supplement: Supplementary file 3 — Source data Fig. 3 [file 44321_2025_355_MOESM3_ESM.zip › Figure 3/3A/855VUS_5dpf.tif]

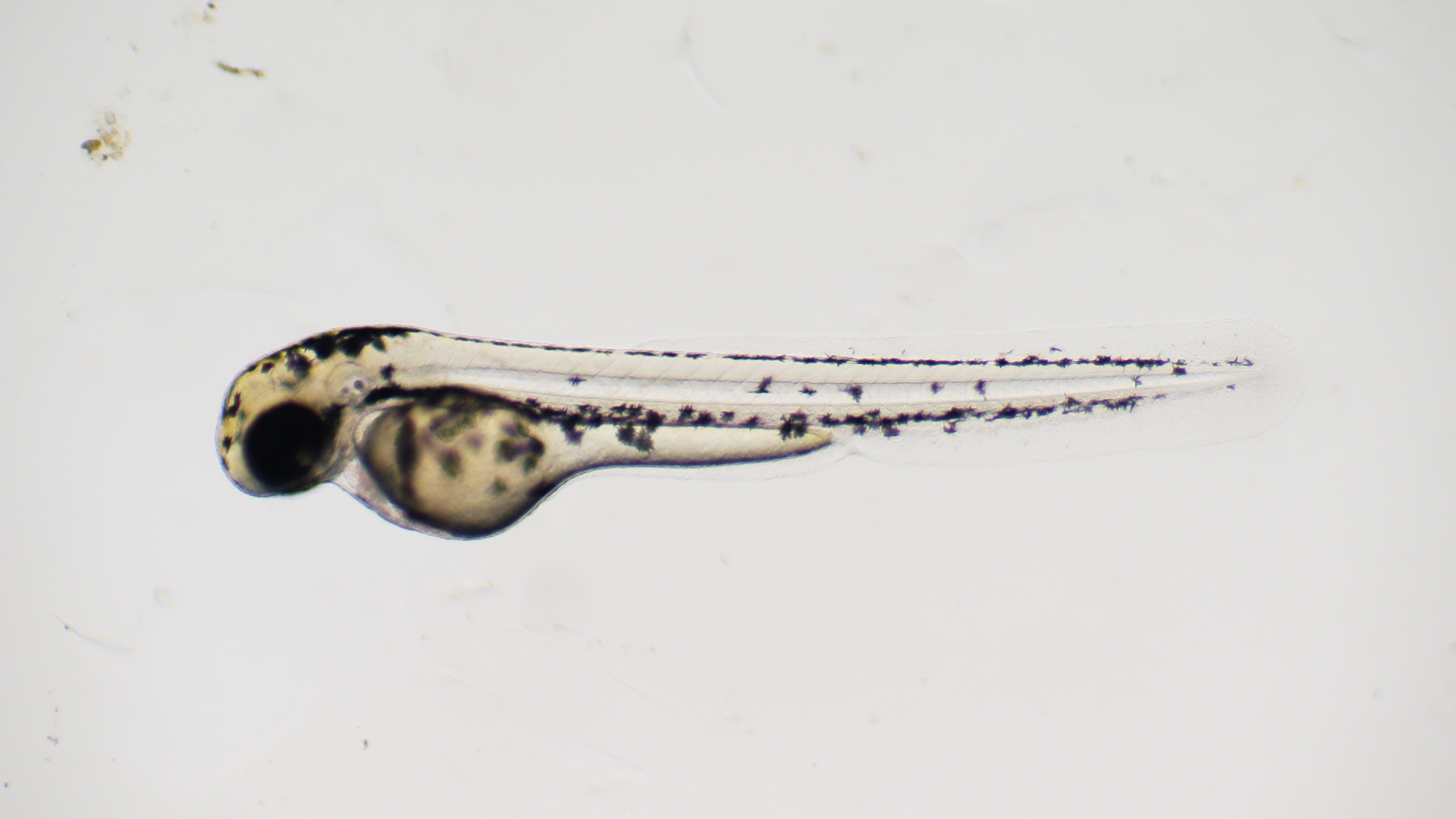

Supplement: Supplementary file 3 — Source data Fig. 3 [file 44321_2025_355_MOESM3_ESM.zip › Figure 3/3A/non-path_2dpf.tif]

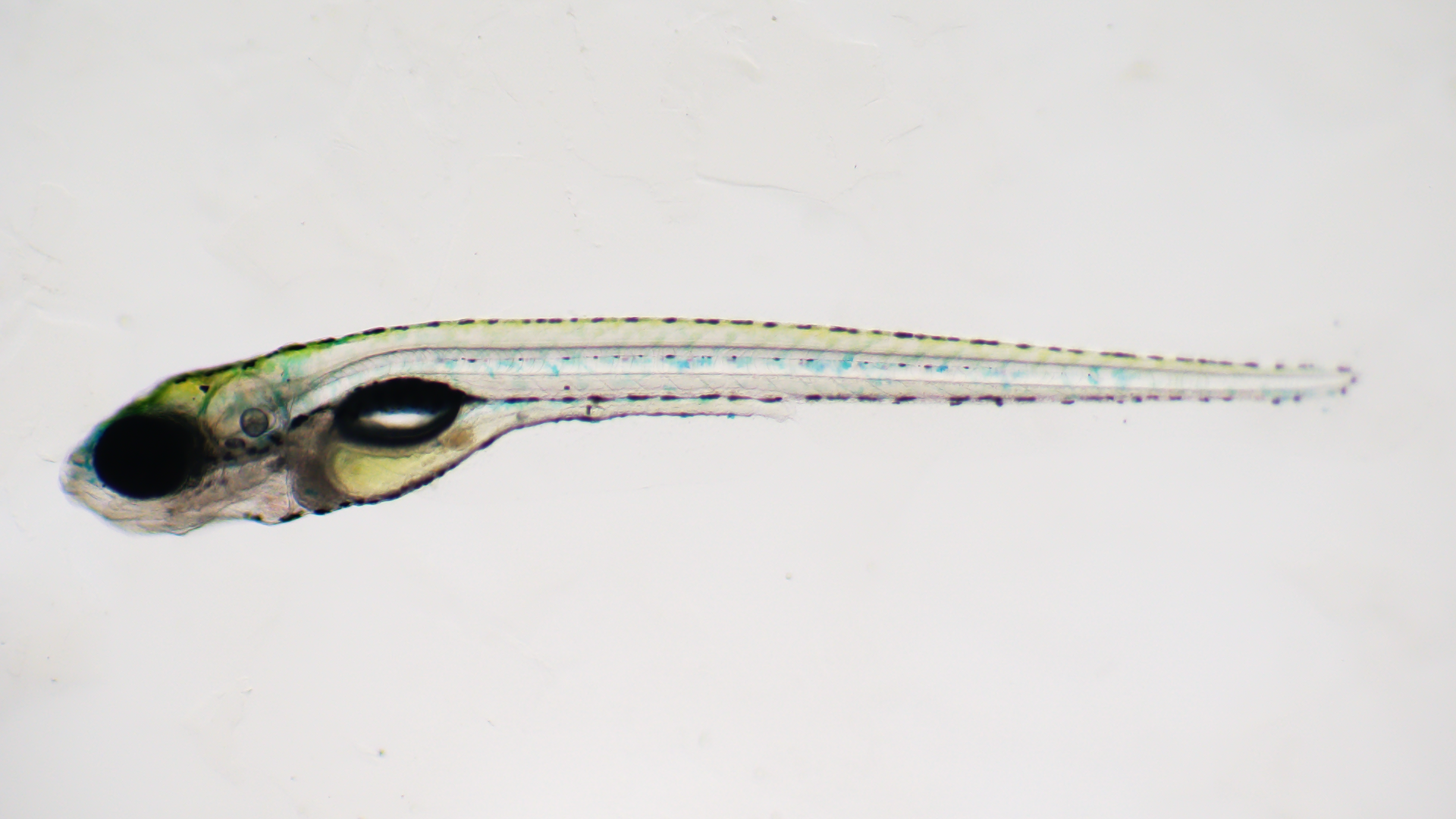

Supplement: Supplementary file 3 — Source data Fig. 3 [file 44321_2025_355_MOESM3_ESM.zip › Figure 3/3A/855VUS_9dpf.tif]

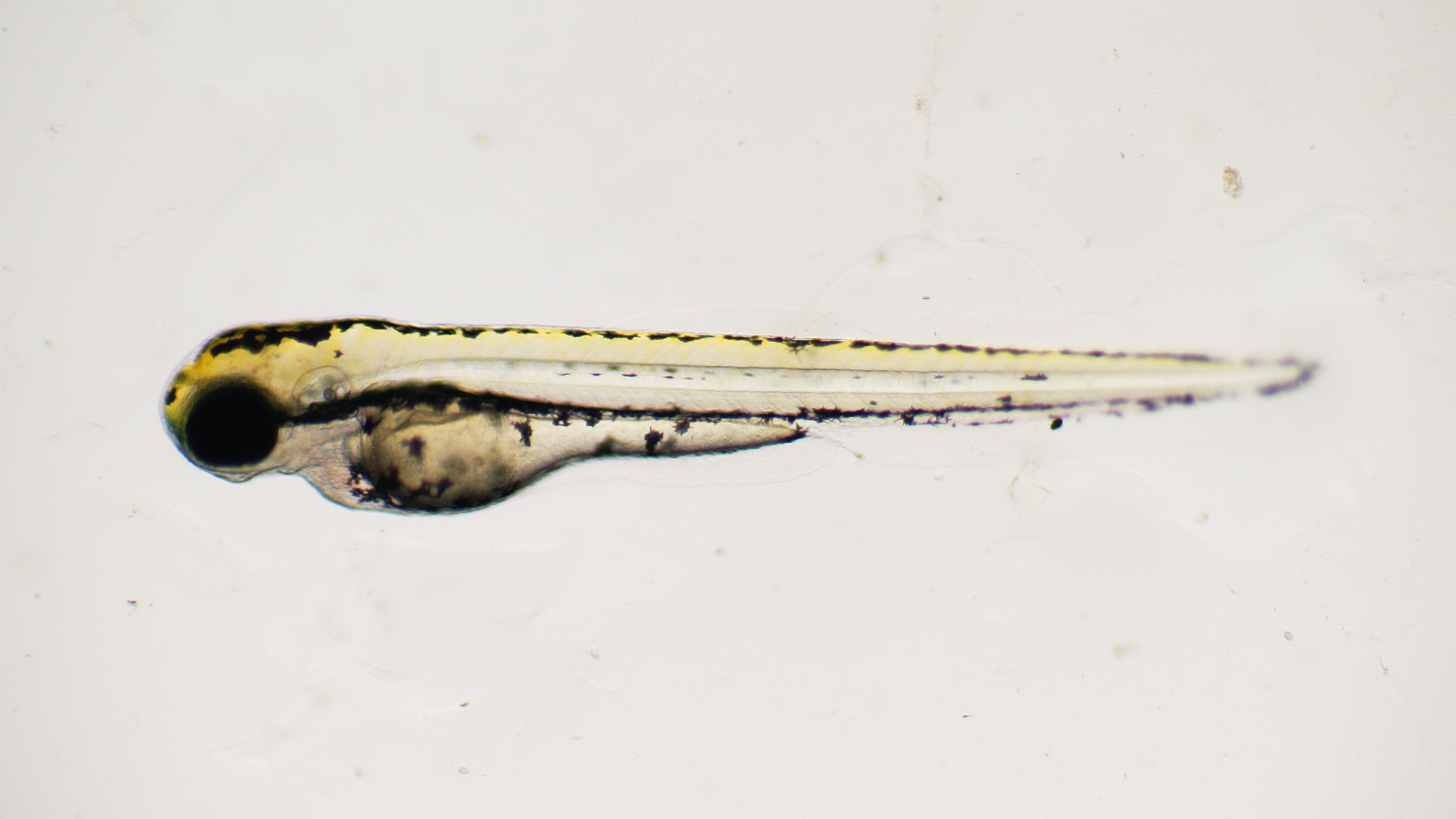

Supplement: Supplementary file 3 — Source data Fig. 3 [file 44321_2025_355_MOESM3_ESM.zip › Figure 3/3A/861VUS_3dpf.tif]

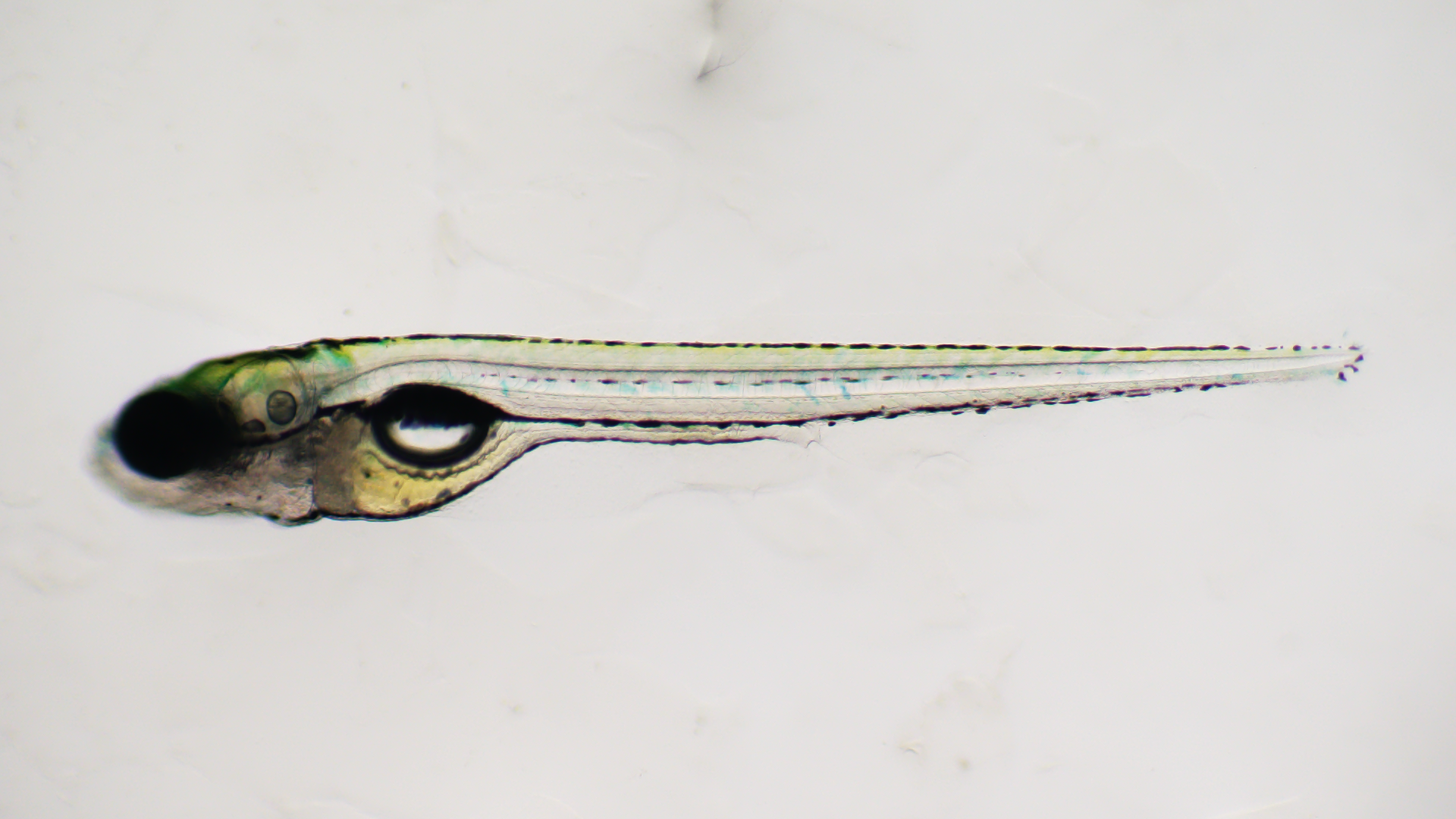

Supplement: Supplementary file 3 — Source data Fig. 3 [file 44321_2025_355_MOESM3_ESM.zip › Figure 3/3A/WT_10dpf.tif]

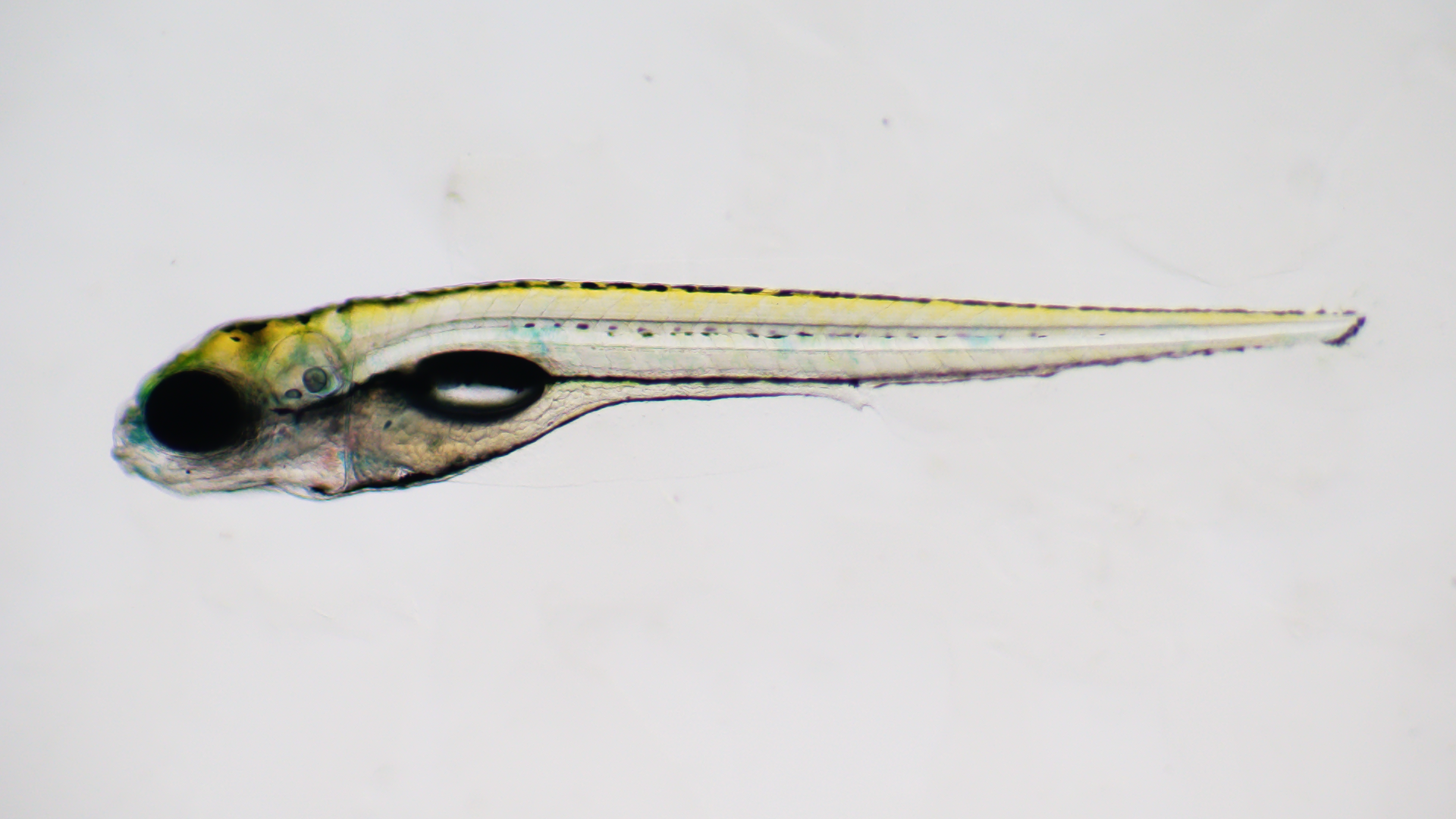

Supplement: Supplementary file 3 — Source data Fig. 3 [file 44321_2025_355_MOESM3_ESM.zip › Figure 3/3A/WT_6dpf.tif]

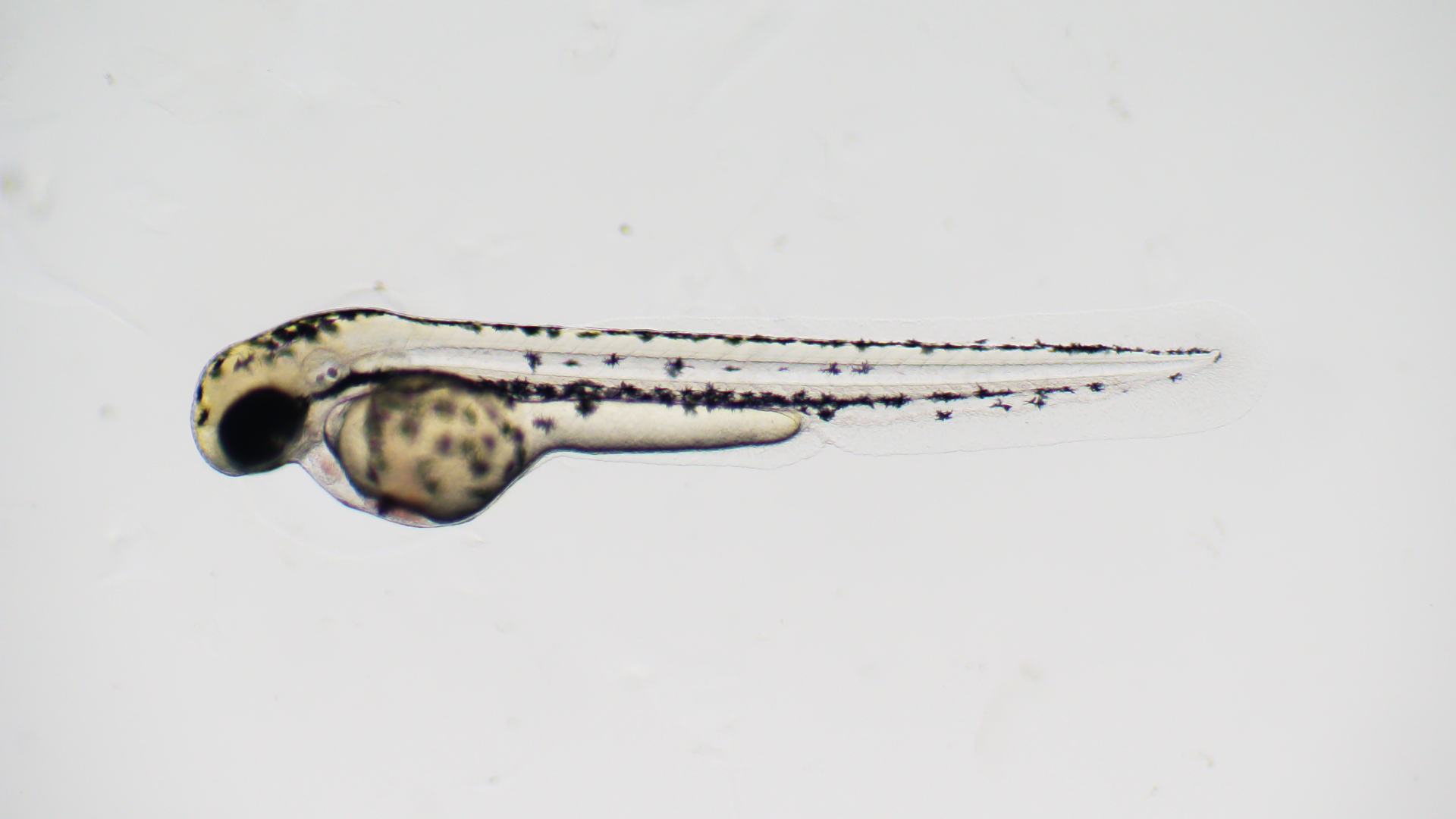

Supplement: Supplementary file 4 — Source data Fig. 4.1 [file 44321_2025_355_MOESM4_ESM.zip › 855VUS_2dpf.tif]

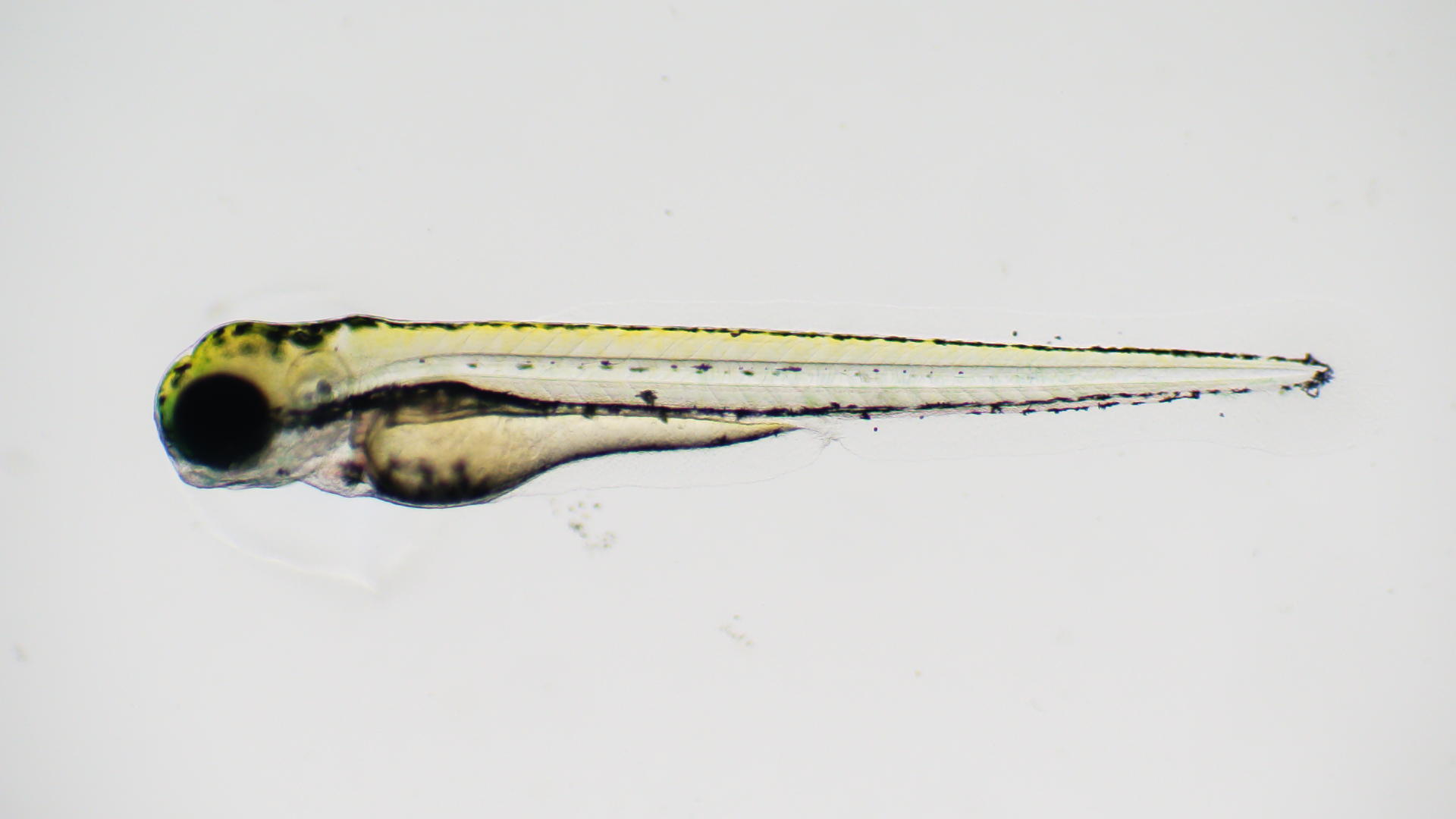

Supplement: Supplementary file 4 — Source data Fig. 4.1 [file 44321_2025_355_MOESM4_ESM.zip › 855VUS_3dpf.tif]

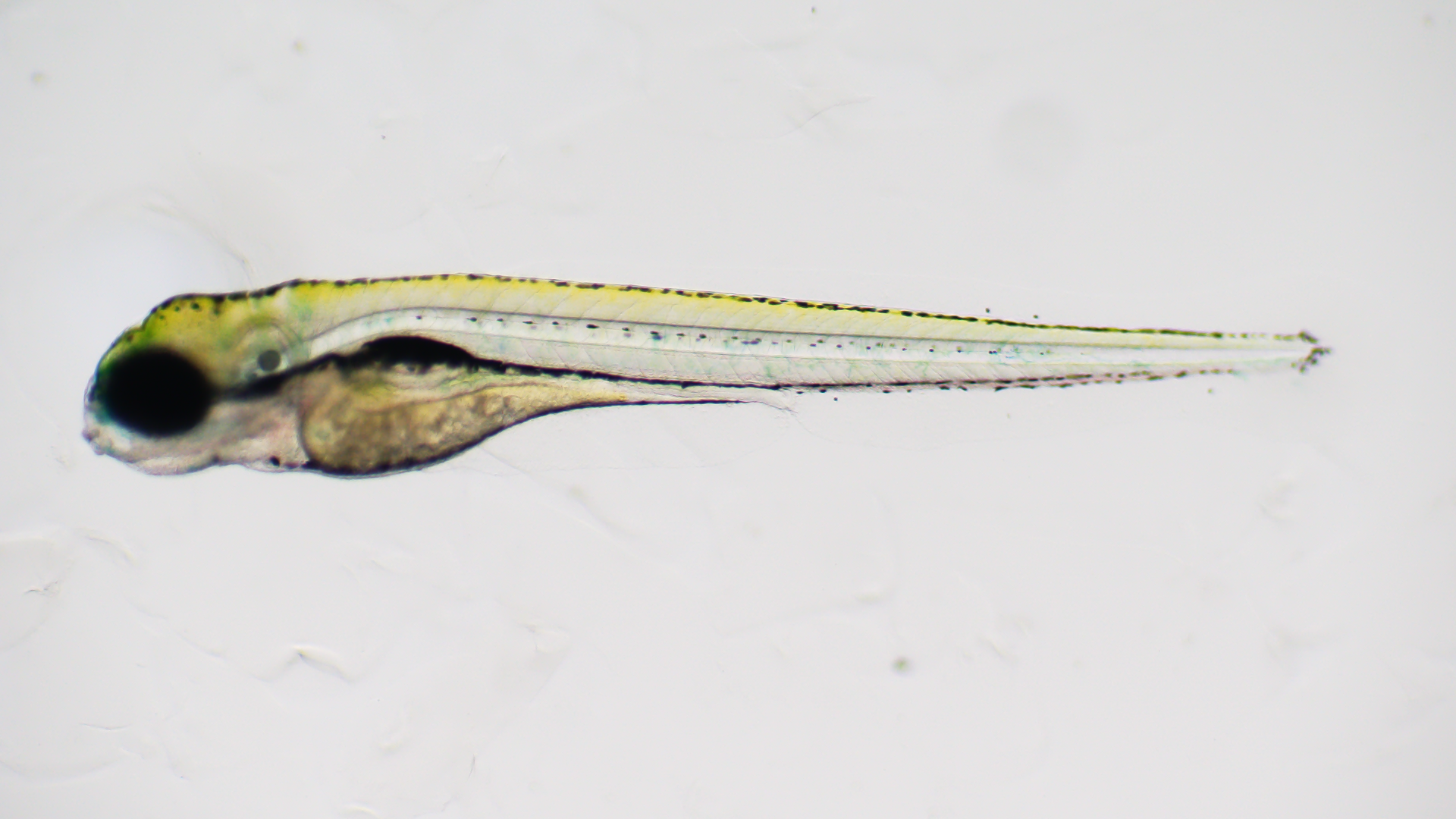

Supplement: Supplementary file 4 — Source data Fig. 4.1 [file 44321_2025_355_MOESM4_ESM.zip › 855VUS_4dpf.tif]

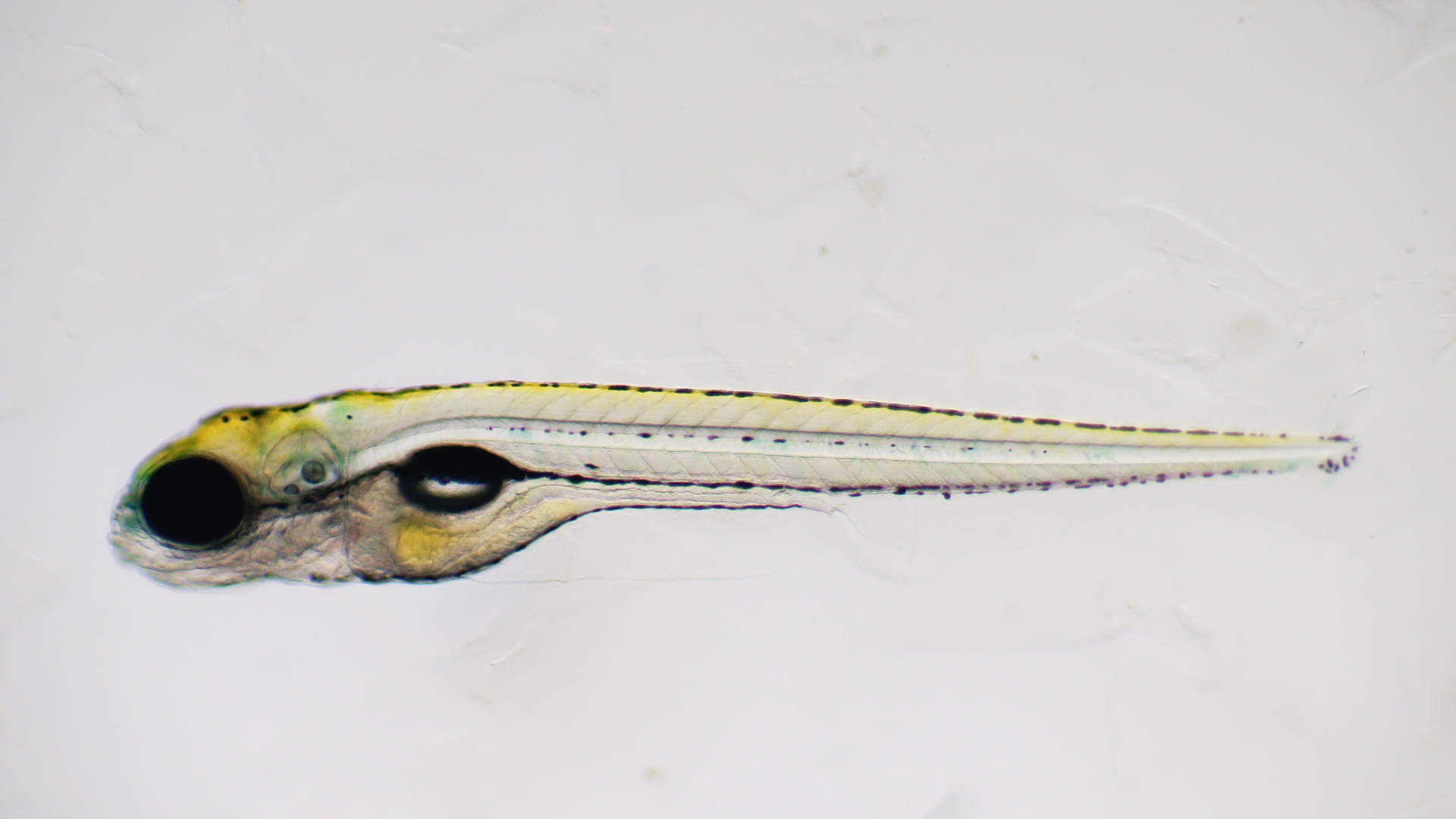

Supplement: Supplementary file 4 — Source data Fig. 4.1 [file 44321_2025_355_MOESM4_ESM.zip › 855VUS_5dpf.tif]

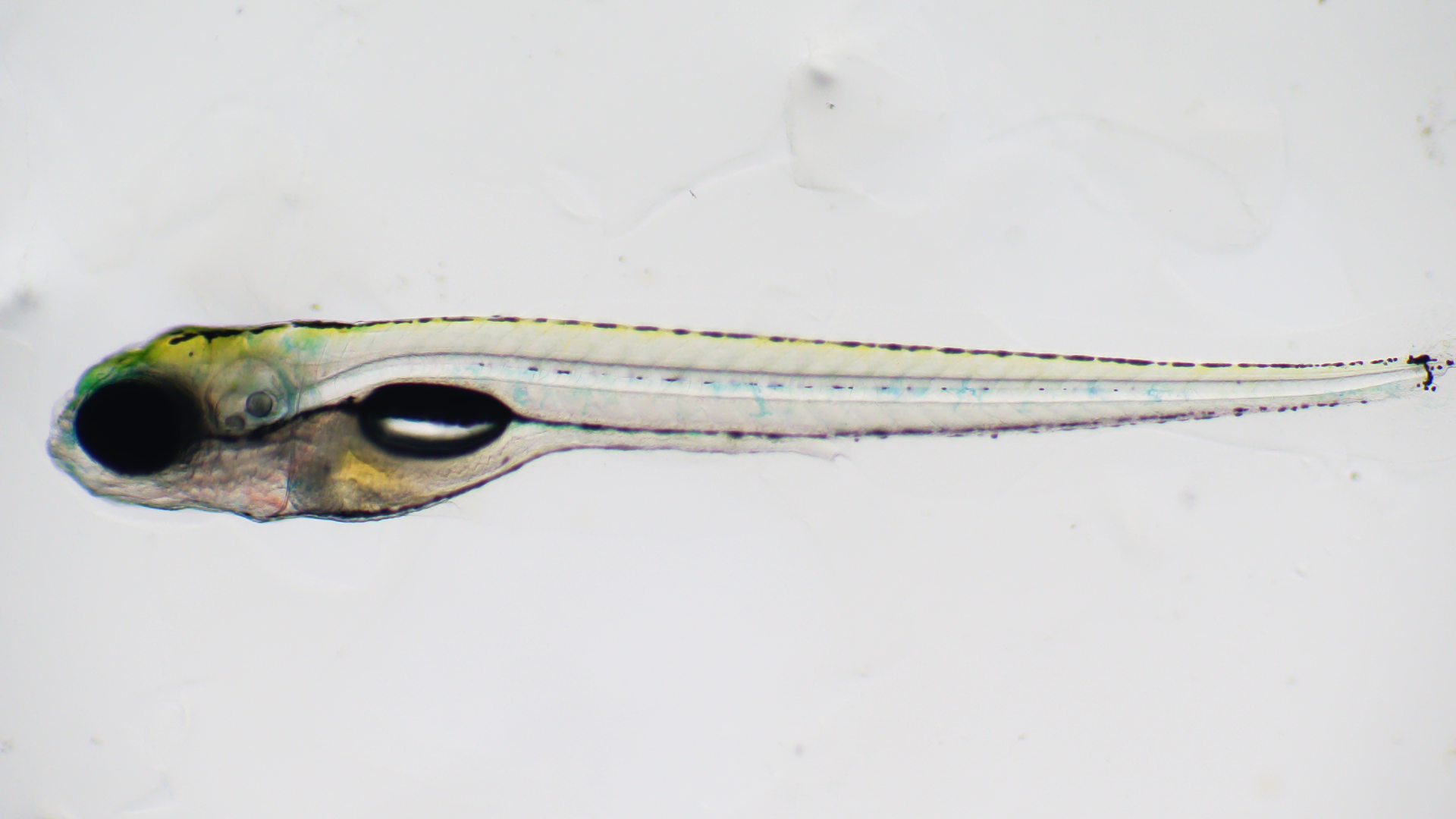

Supplement: Supplementary file 4 — Source data Fig. 4.1 [file 44321_2025_355_MOESM4_ESM.zip › 855VUS_6dpf.tif]

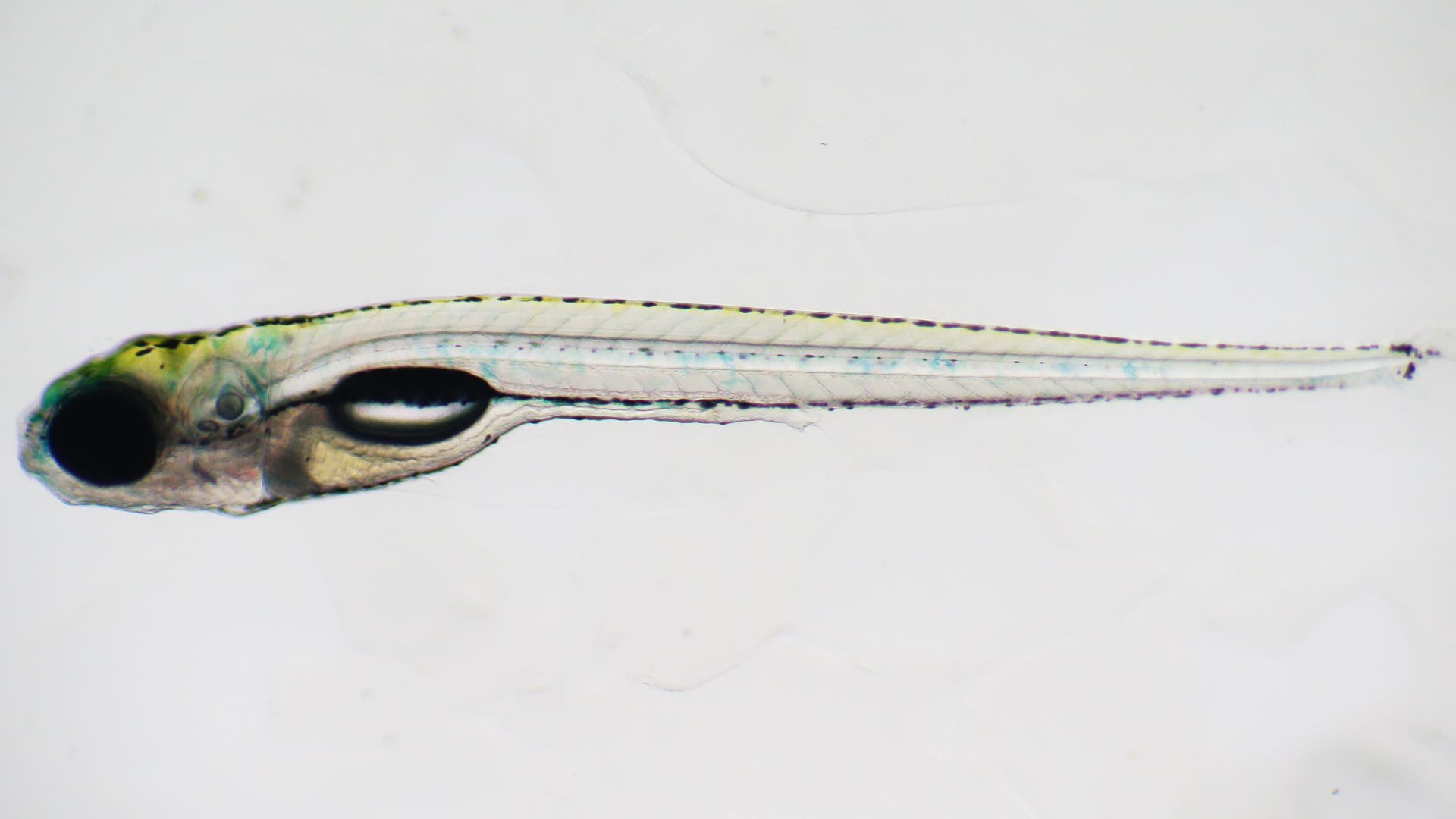

Supplement: Supplementary file 4 — Source data Fig. 4.1 [file 44321_2025_355_MOESM4_ESM.zip › 855VUS_7dpf.tif]

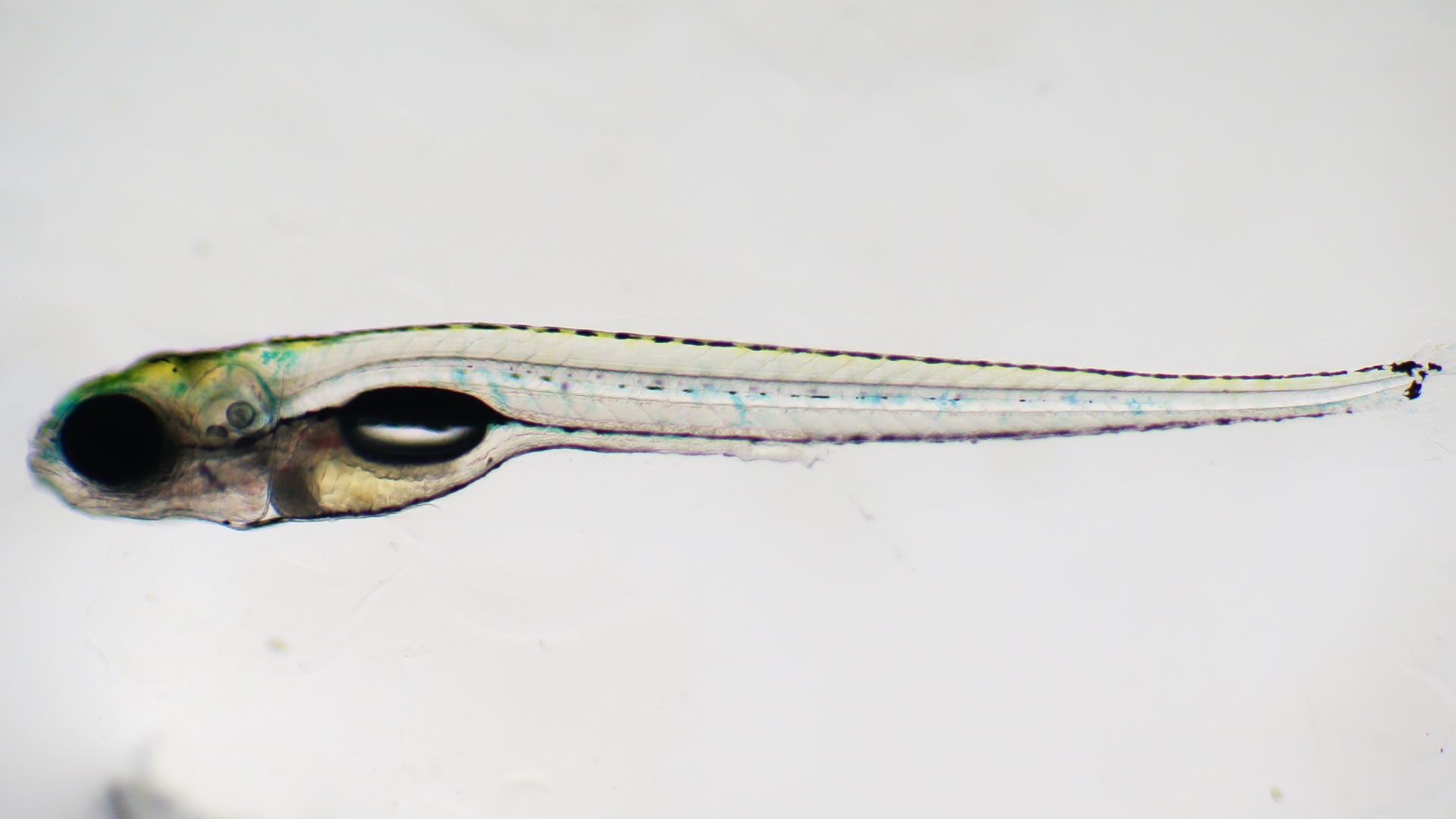

Supplement: Supplementary file 4 — Source data Fig. 4.1 [file 44321_2025_355_MOESM4_ESM.zip › 855VUS_8dpf.tif]

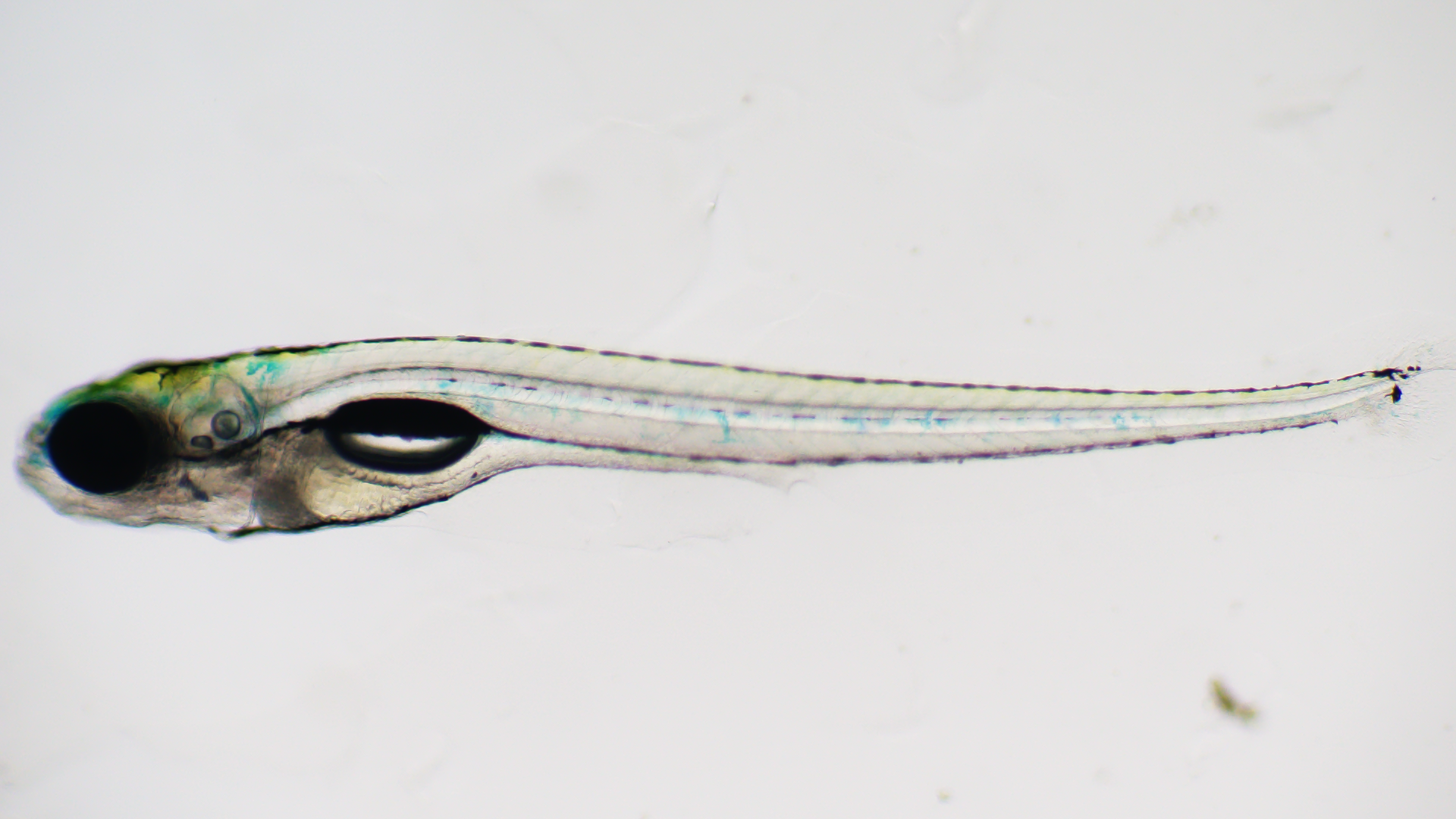

Supplement: Supplementary file 4 — Source data Fig. 4.1 [file 44321_2025_355_MOESM4_ESM.zip › 855VUS_9dpf.tif]

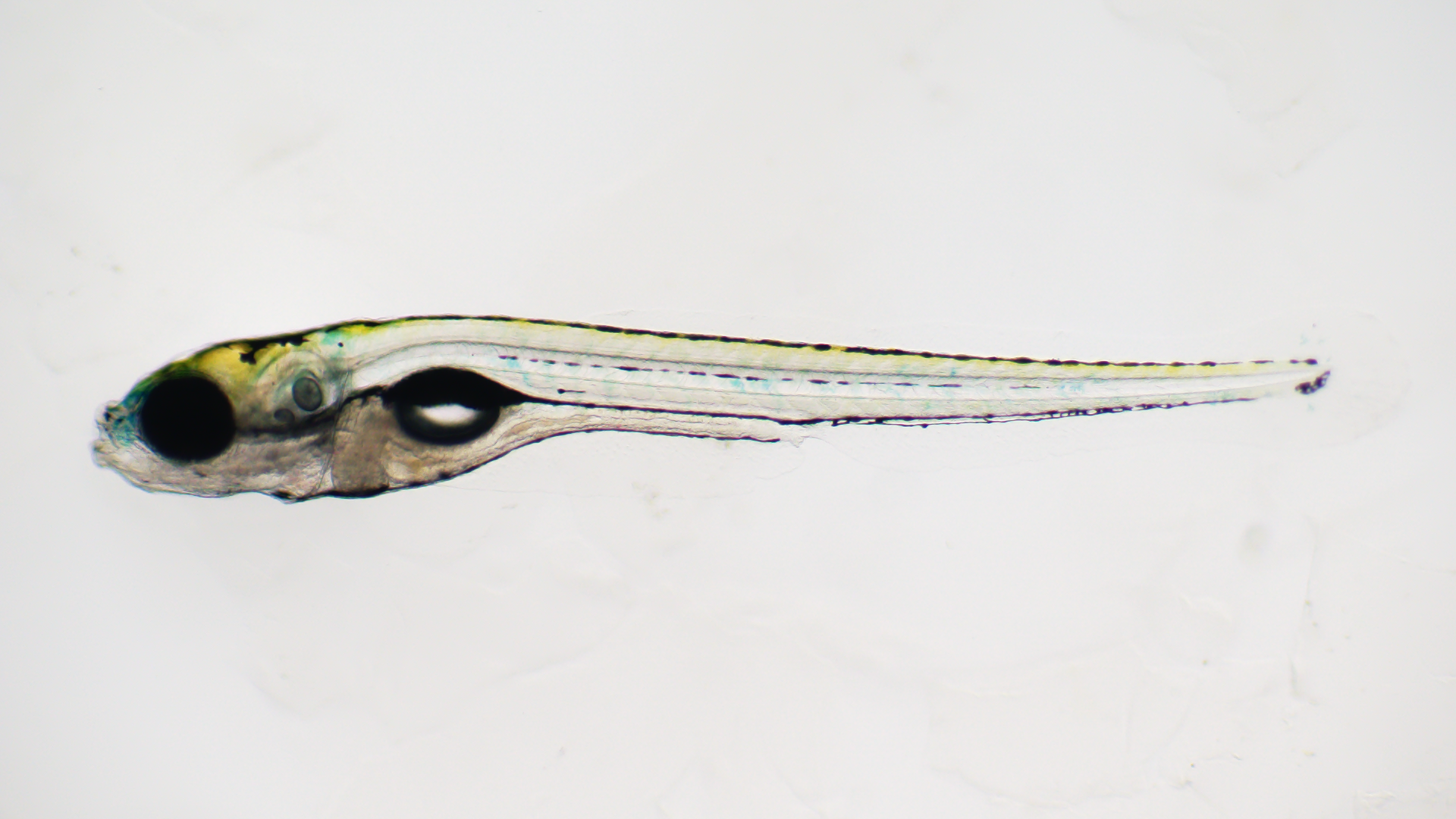

Supplement: Supplementary file 4 — Source data Fig. 4.1 [file 44321_2025_355_MOESM4_ESM.zip › 855VUS_10dpf.tif]

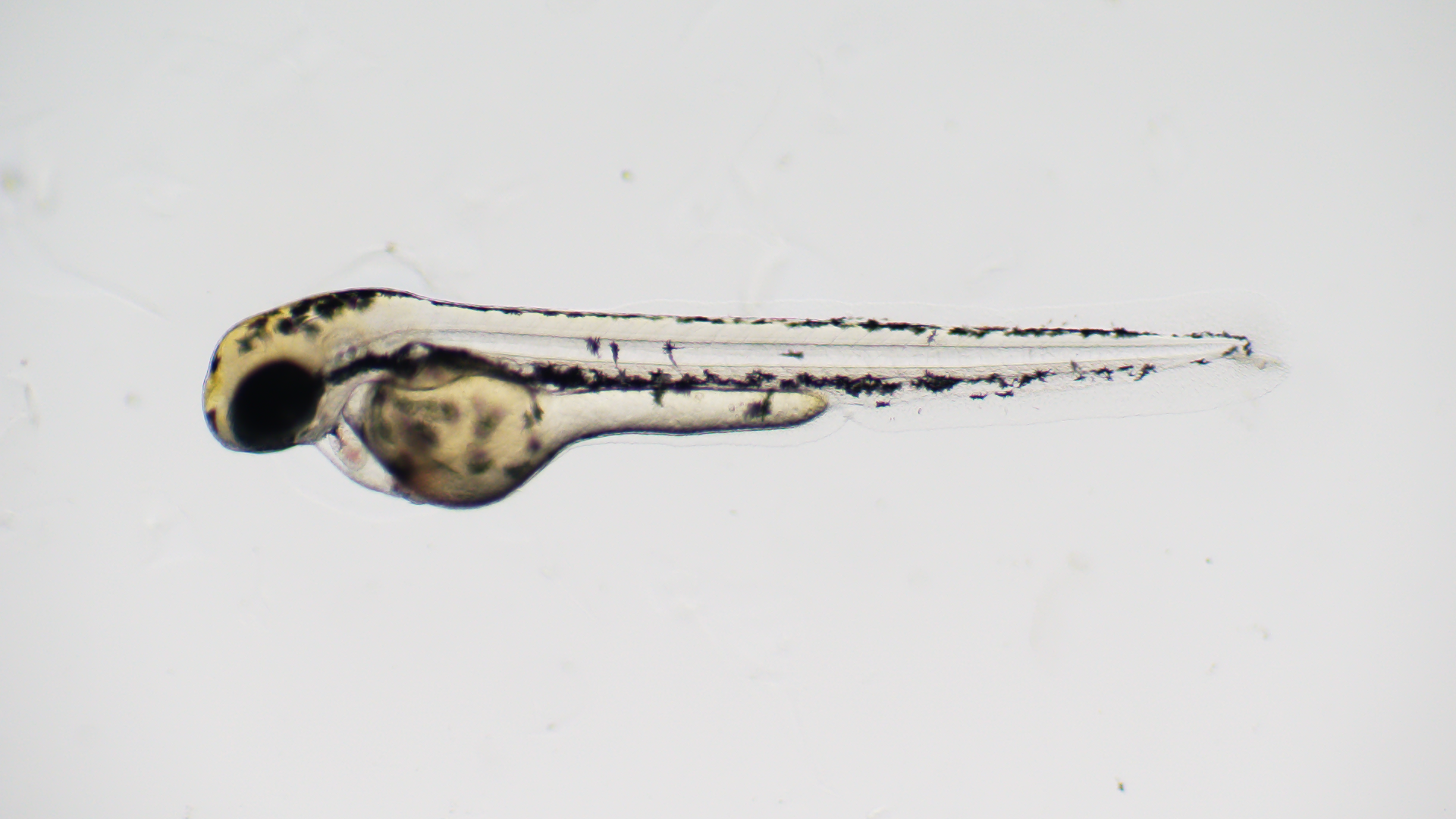

Supplement: Supplementary file 4 — Source data Fig. 4.1 [file 44321_2025_355_MOESM4_ESM.zip › 861VUS_2dpf.tif]

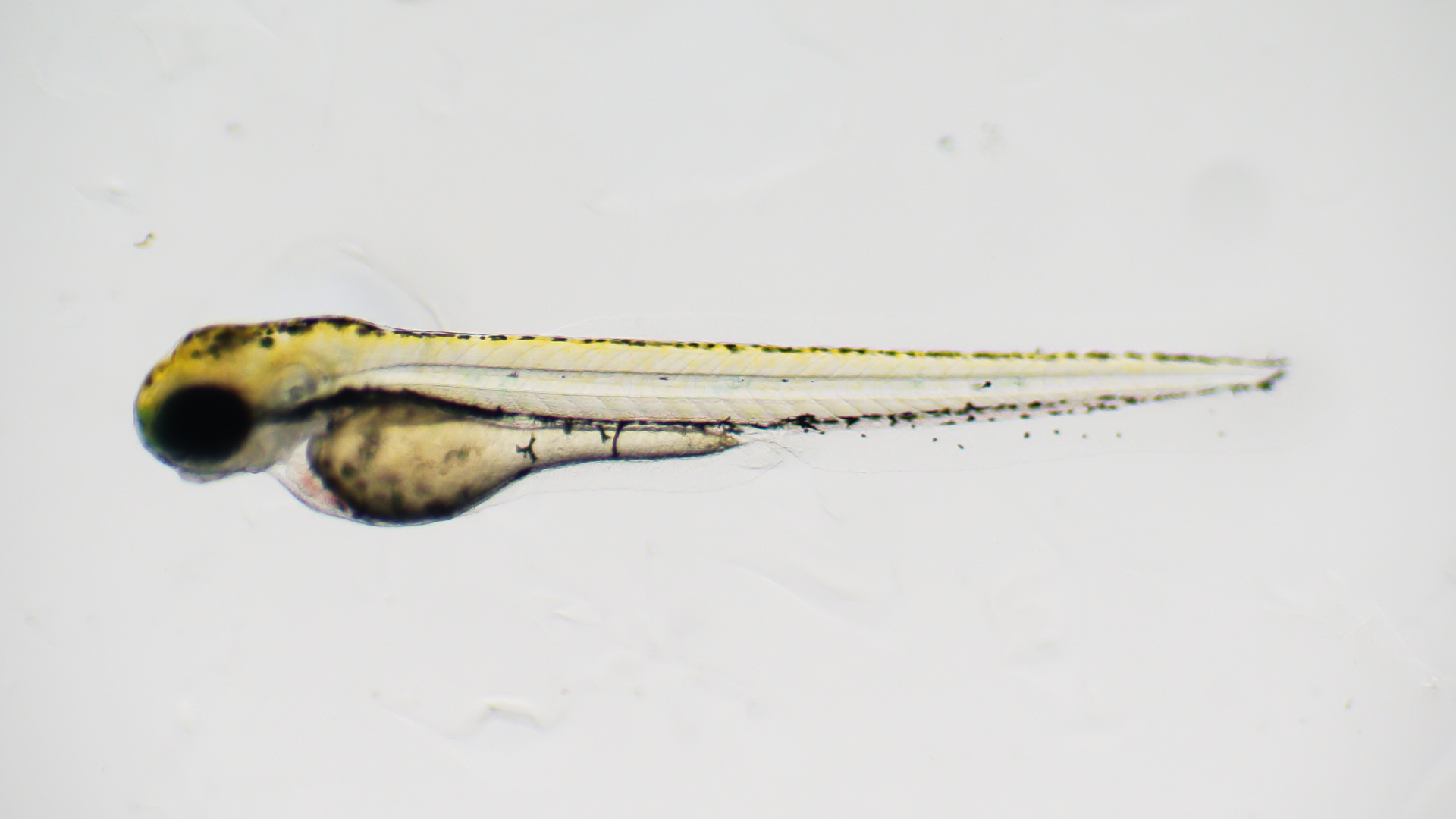

Supplement: Supplementary file 4 — Source data Fig. 4.1 [file 44321_2025_355_MOESM4_ESM.zip › 861VUS_3dpf.tif]

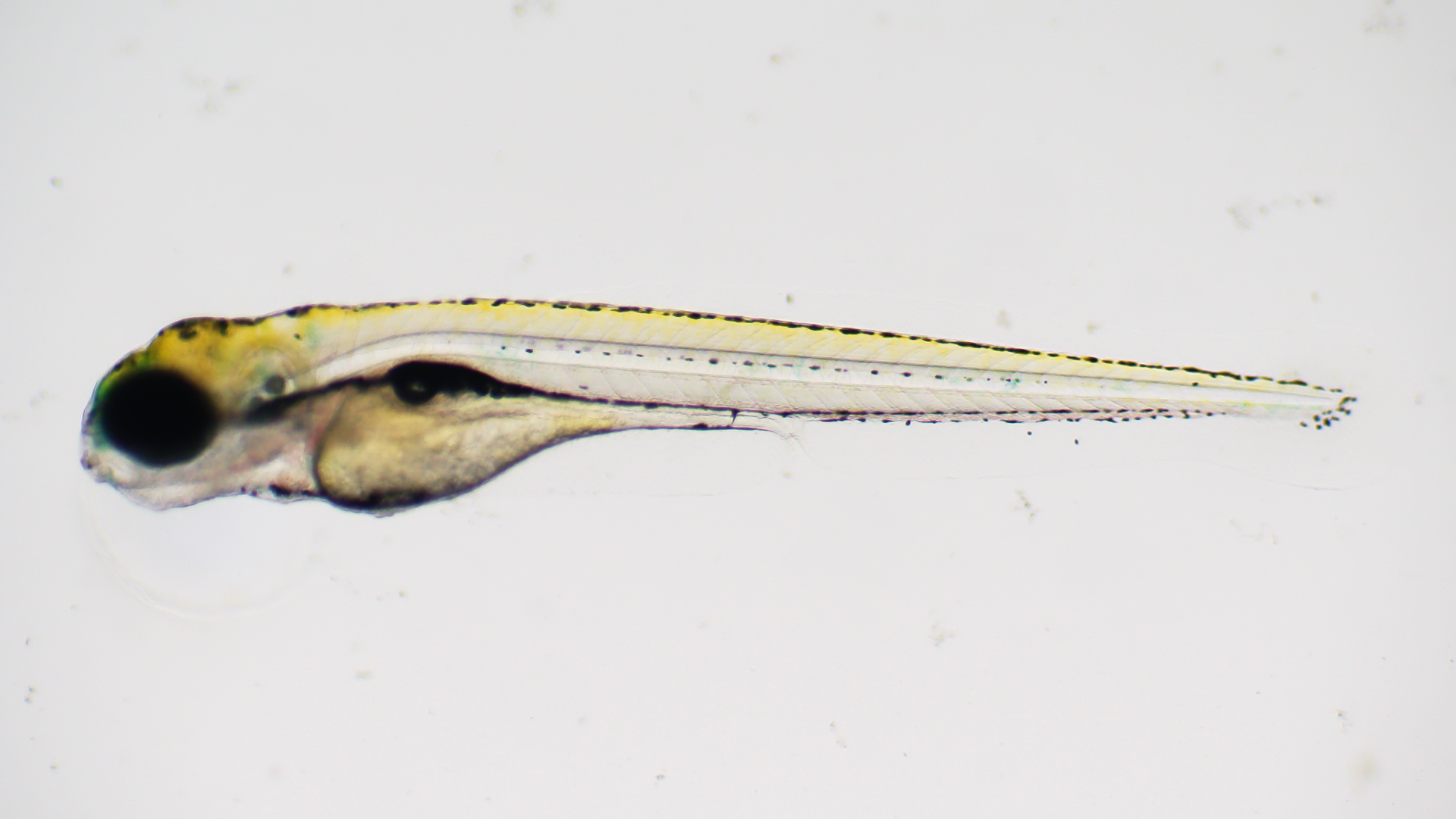

Supplement: Supplementary file 4 — Source data Fig. 4.1 [file 44321_2025_355_MOESM4_ESM.zip › 861VUS_4dpf.tif]

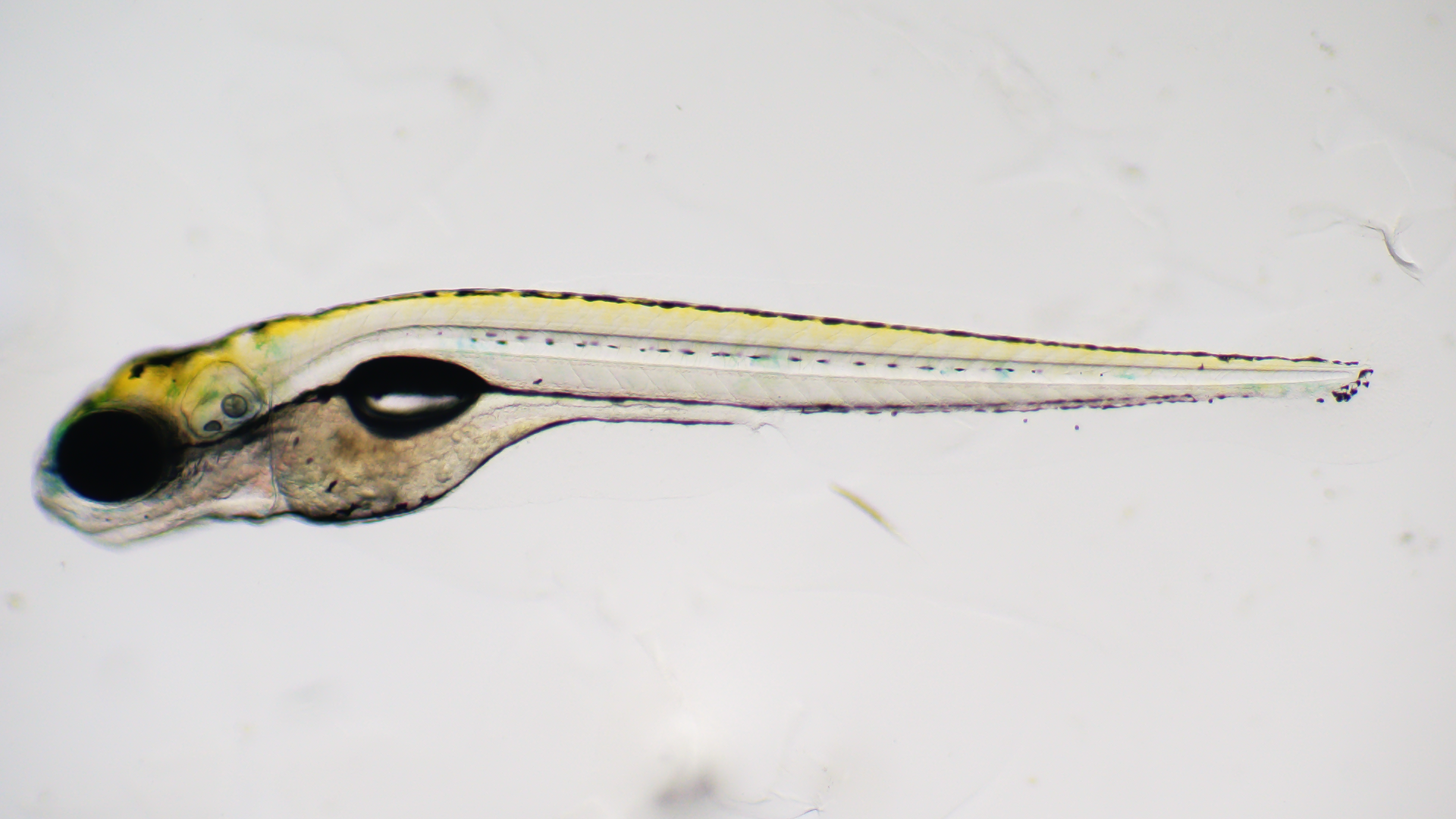

Supplement: Supplementary file 4 — Source data Fig. 4.1 [file 44321_2025_355_MOESM4_ESM.zip › 861VUS_5dpf.tif]

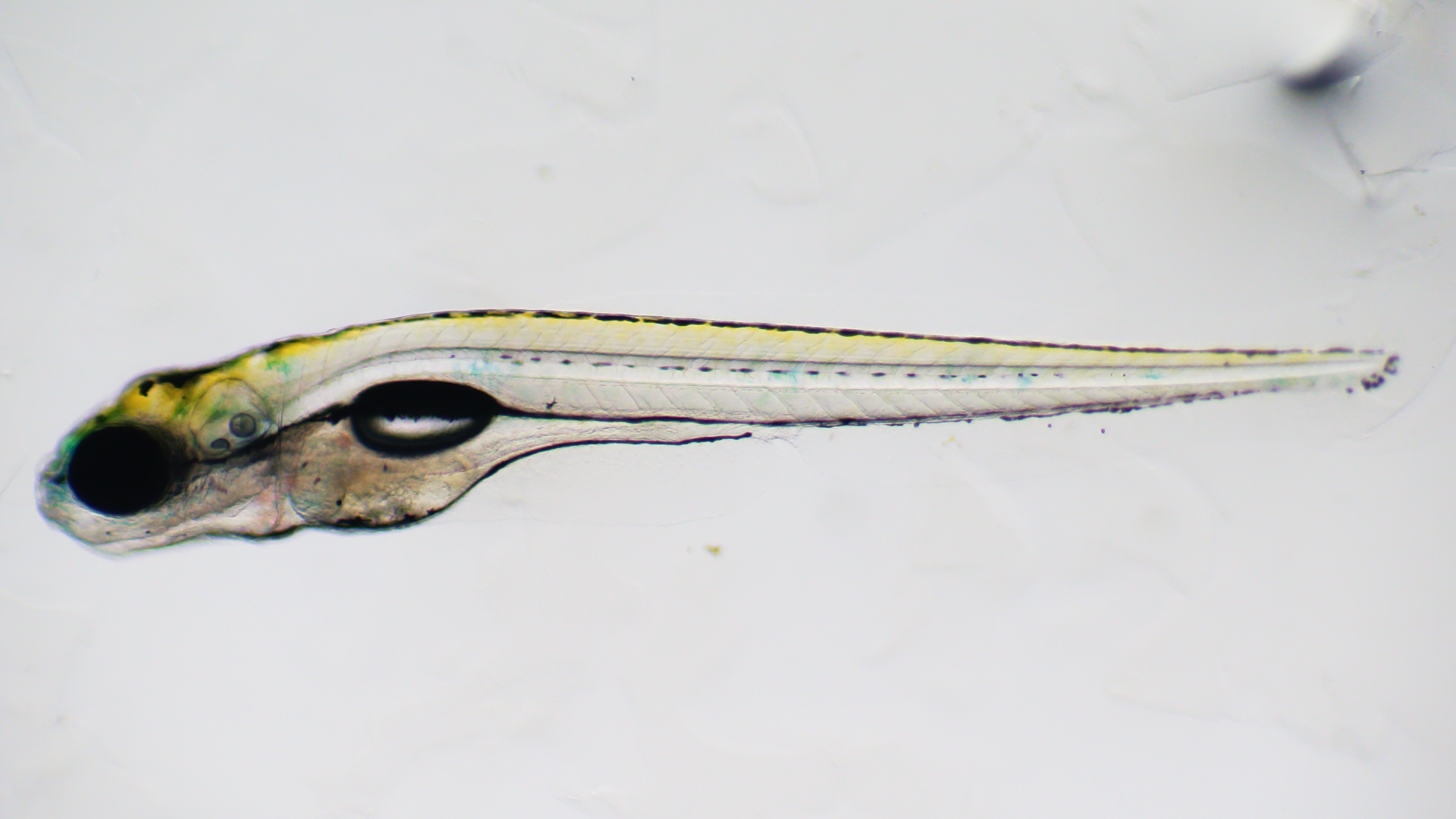

Supplement: Supplementary file 4 — Source data Fig. 4.1 [file 44321_2025_355_MOESM4_ESM.zip › 861VUS_6dpf.tif]

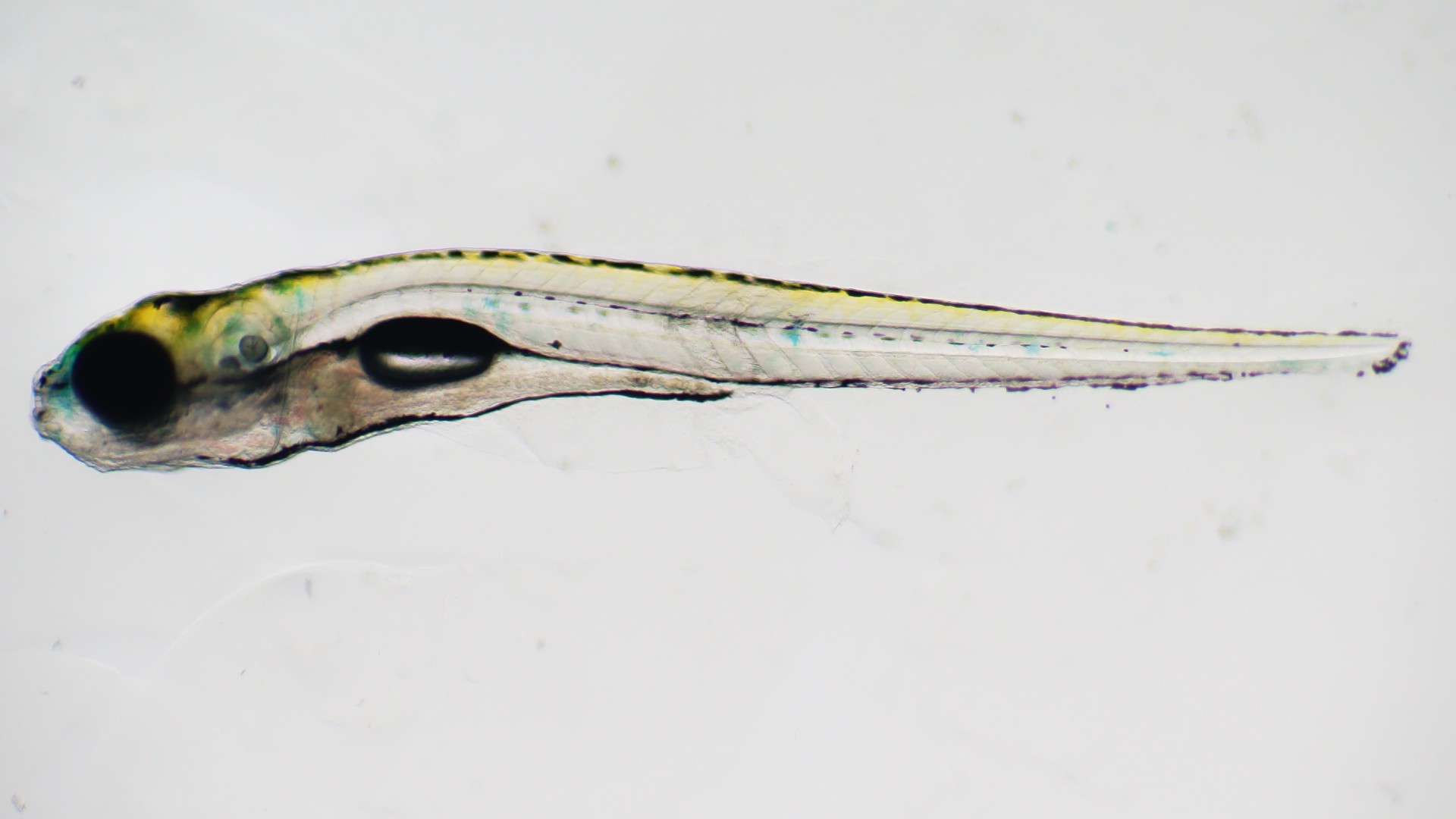

Supplement: Supplementary file 4 — Source data Fig. 4.1 [file 44321_2025_355_MOESM4_ESM.zip › 861VUS_7dpf.tif]

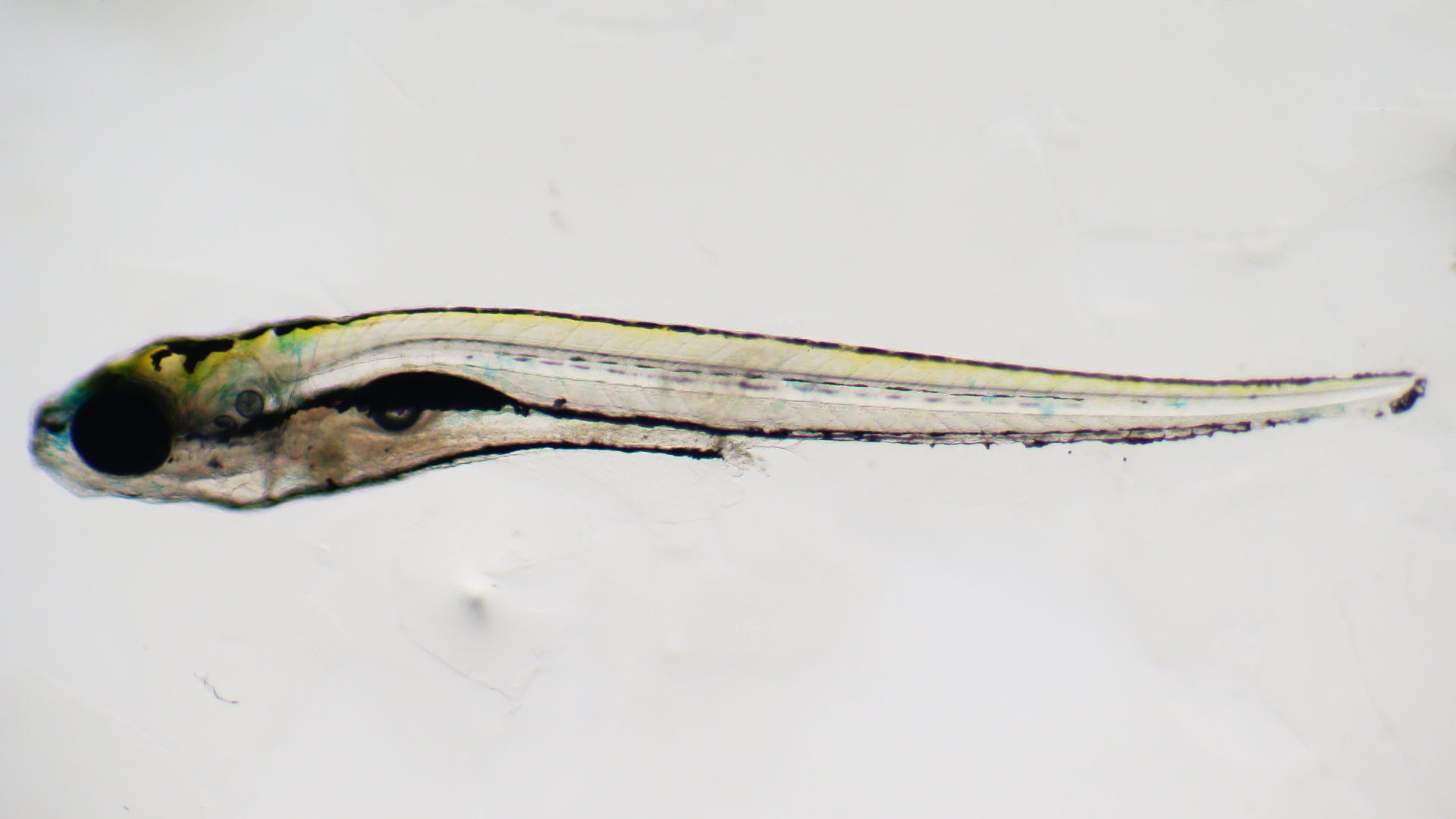

Supplement: Supplementary file 4 — Source data Fig. 4.1 [file 44321_2025_355_MOESM4_ESM.zip › 861VUS_8dpf.tif]

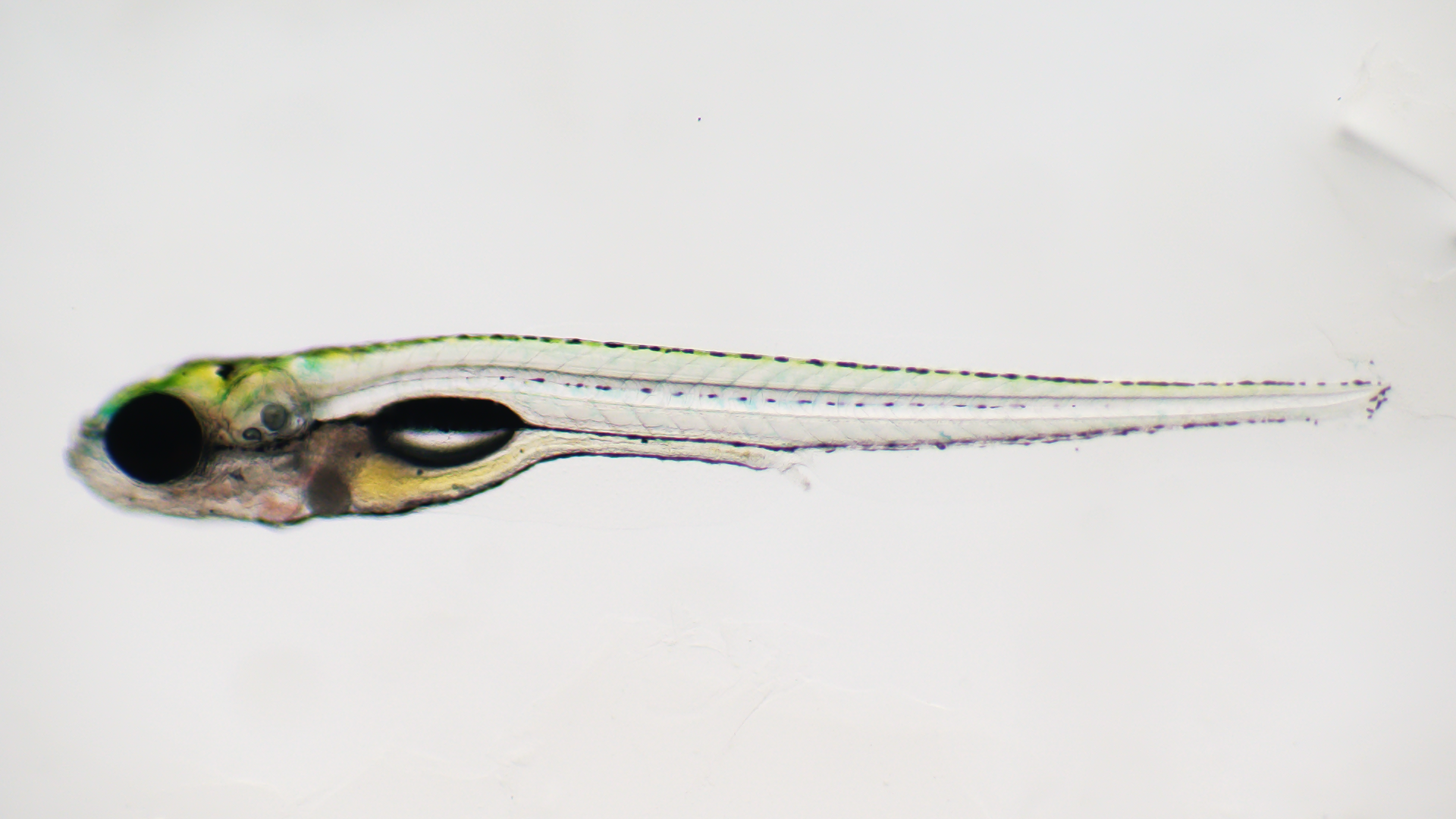

Supplement: Supplementary file 4 — Source data Fig. 4.1 [file 44321_2025_355_MOESM4_ESM.zip › 861VUS_9dpf.tif]

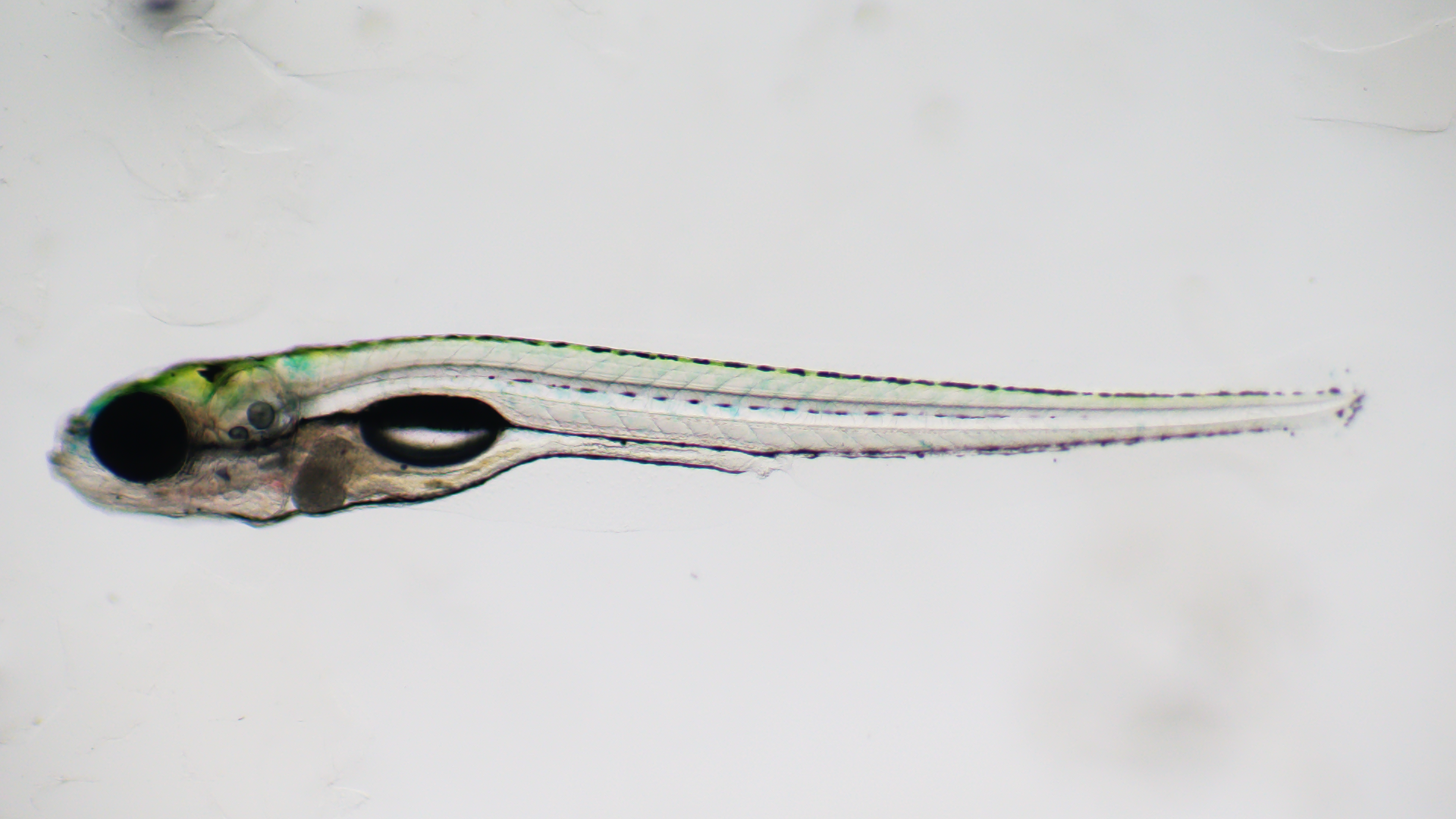

Supplement: Supplementary file 4 — Source data Fig. 4.1 [file 44321_2025_355_MOESM4_ESM.zip › 861VUS_10dpf.tif]

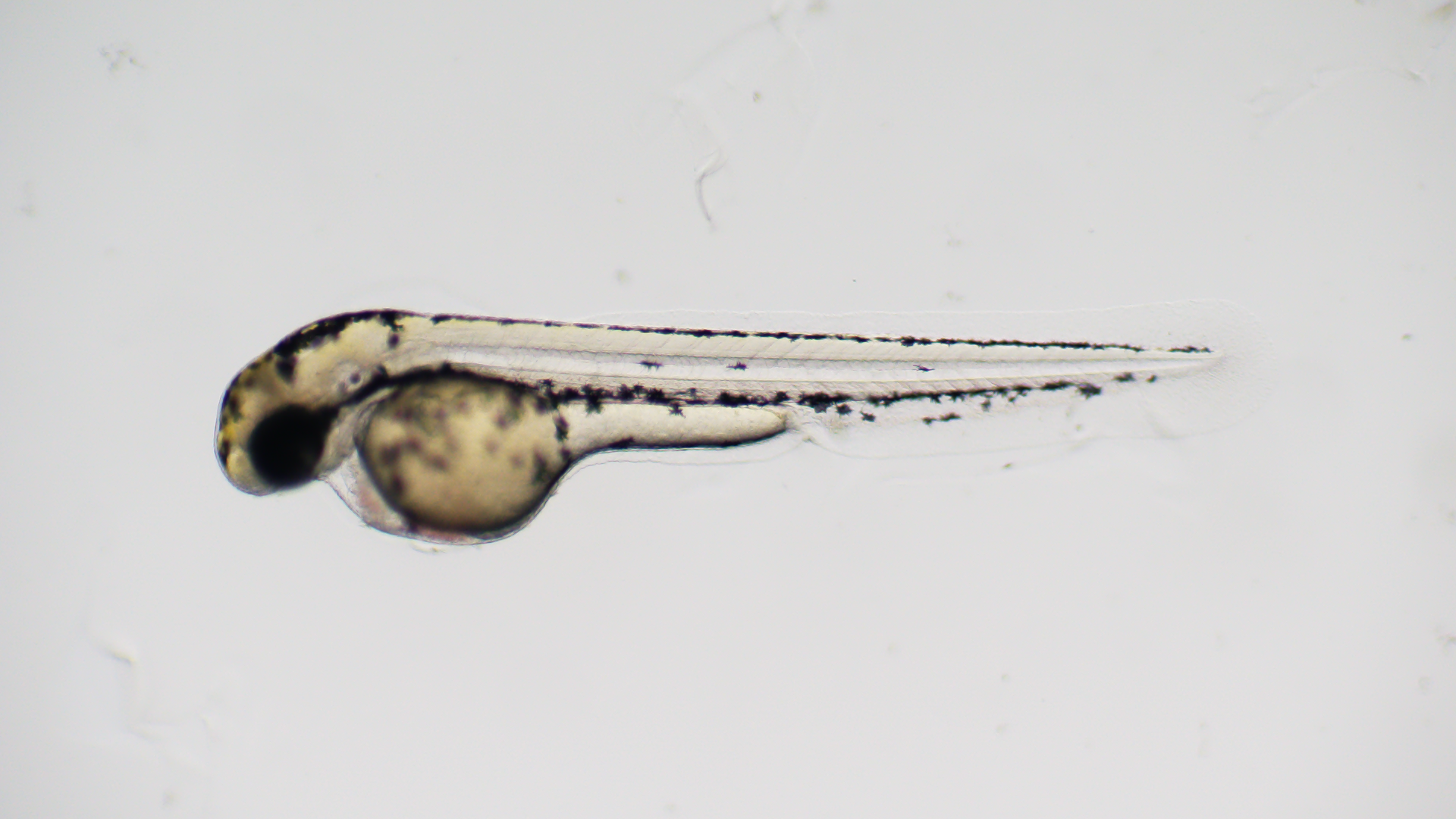

Supplement: Supplementary file 4 — Source data Fig. 4.1 [file 44321_2025_355_MOESM4_ESM.zip › A2G_2dpf.tif]

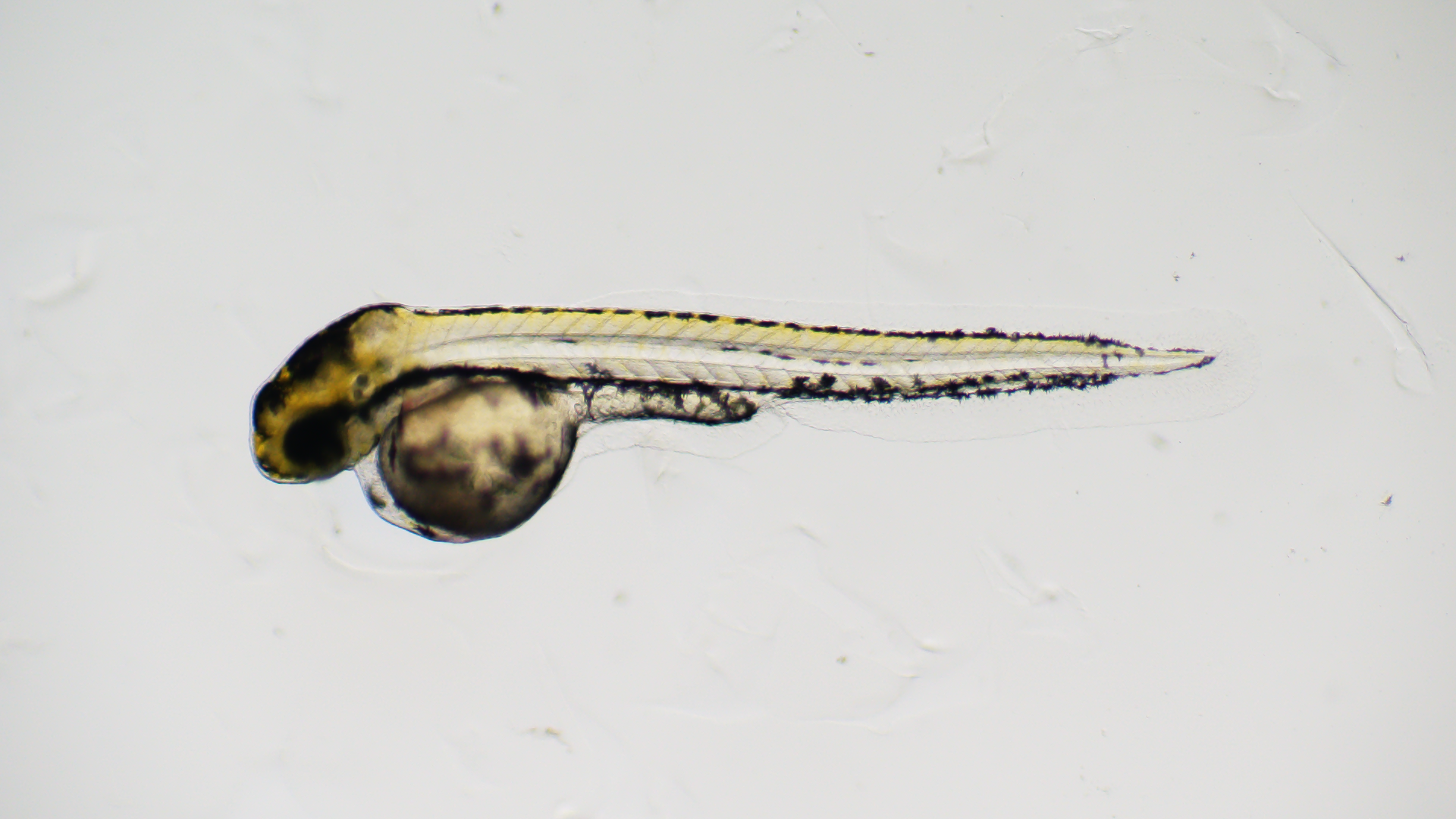

Supplement: Supplementary file 4 — Source data Fig. 4.1 [file 44321_2025_355_MOESM4_ESM.zip › A2G_3dpf.tif]

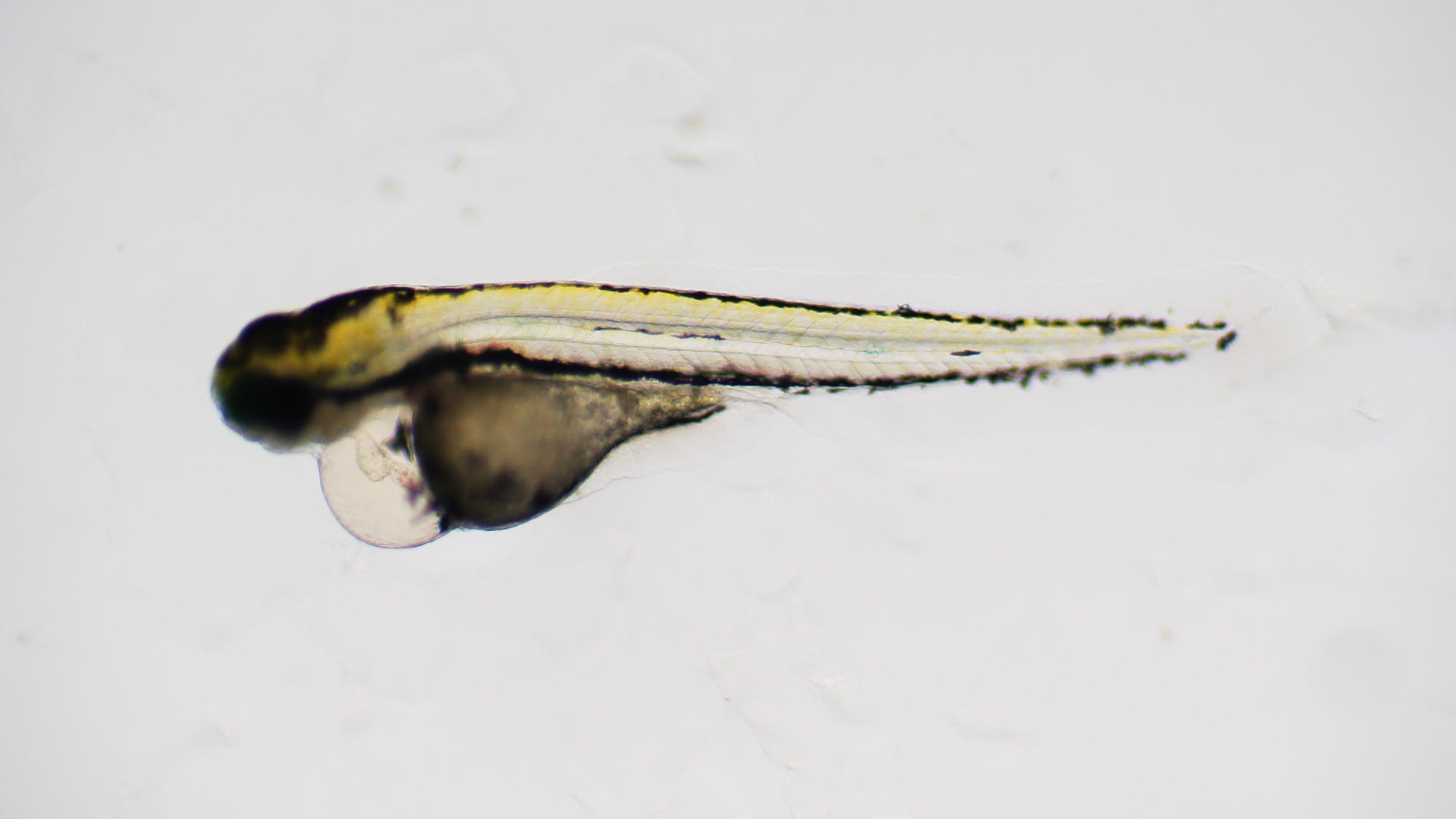

Supplement: Supplementary file 4 — Source data Fig. 4.1 [file 44321_2025_355_MOESM4_ESM.zip › A2G_4dpf.tif]

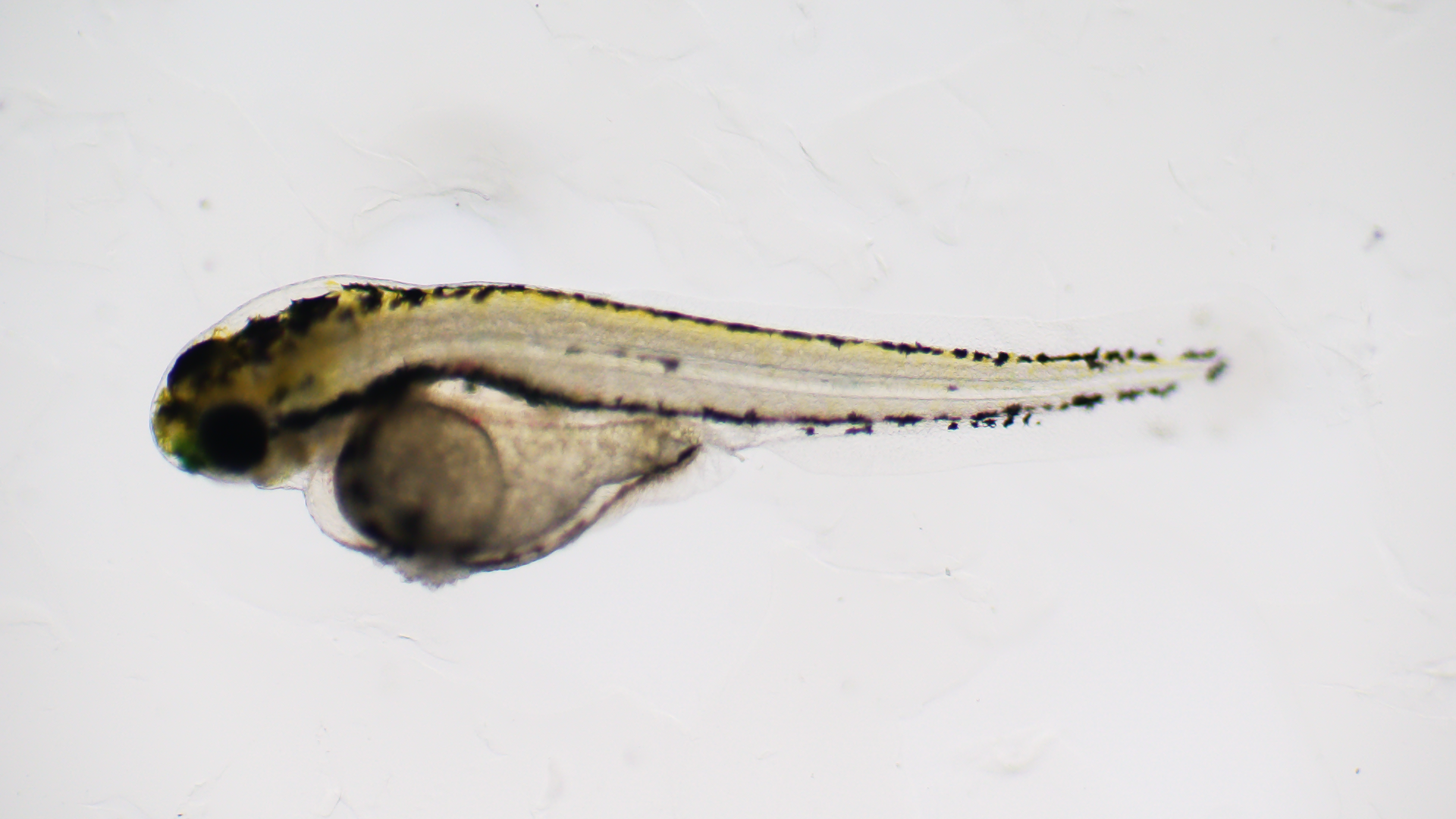

Supplement: Supplementary file 4 — Source data Fig. 4.1 [file 44321_2025_355_MOESM4_ESM.zip › A2G_5dpf.tif]

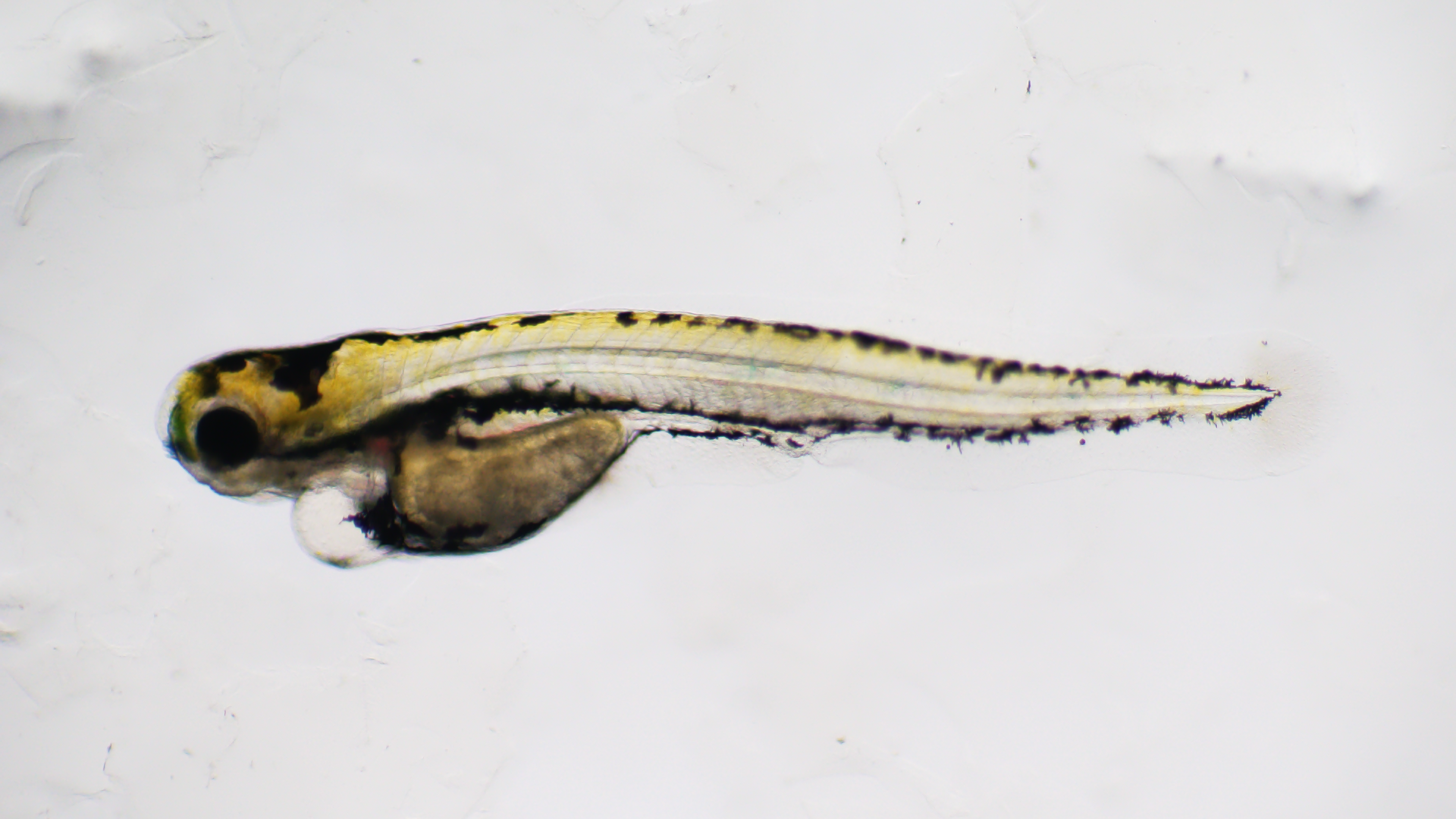

Supplement: Supplementary file 4 — Source data Fig. 4.1 [file 44321_2025_355_MOESM4_ESM.zip › A2G_6dpf.tif]

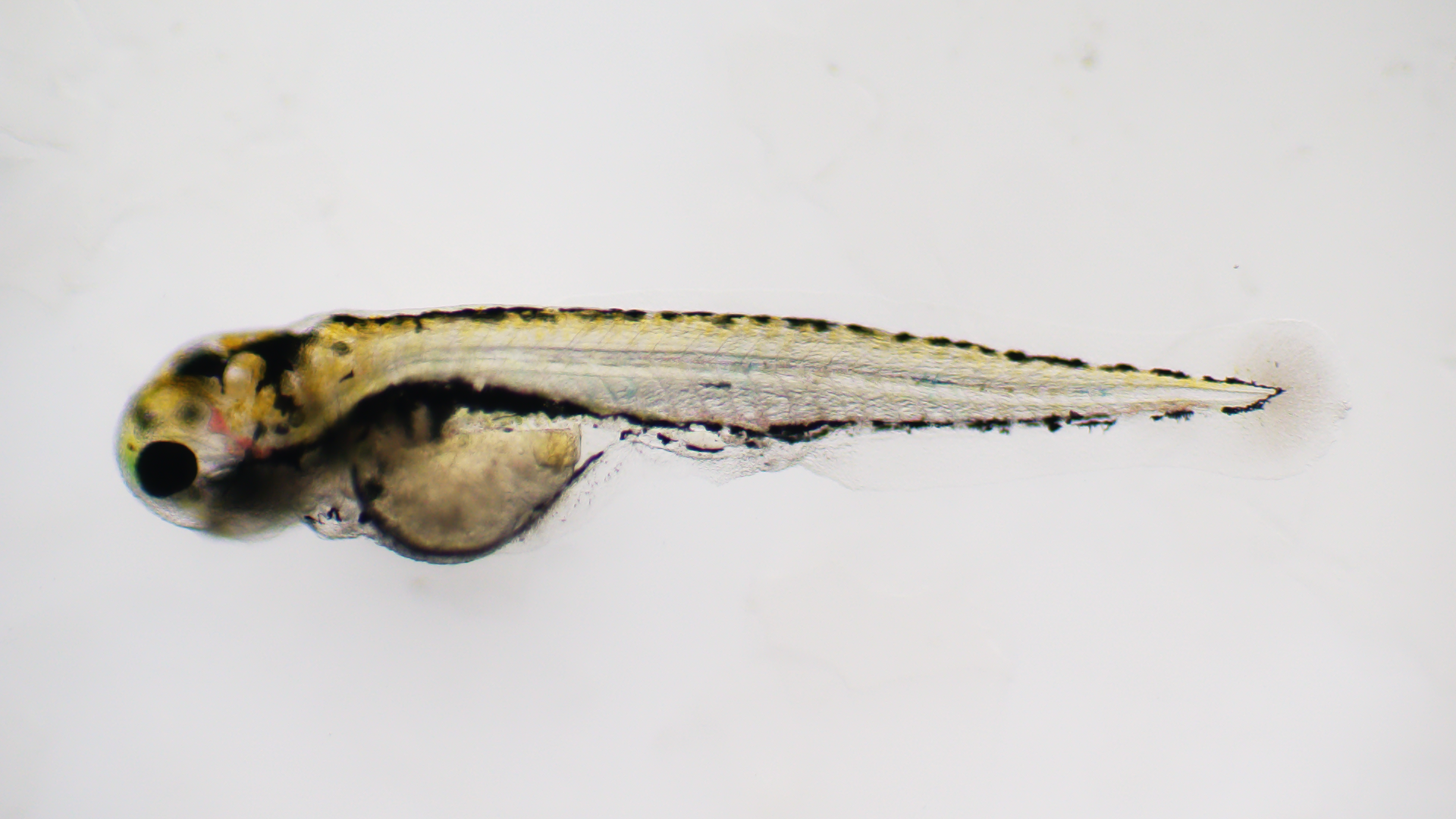

Supplement: Supplementary file 4 — Source data Fig. 4.1 [file 44321_2025_355_MOESM4_ESM.zip › A2G_7dpf.tif]

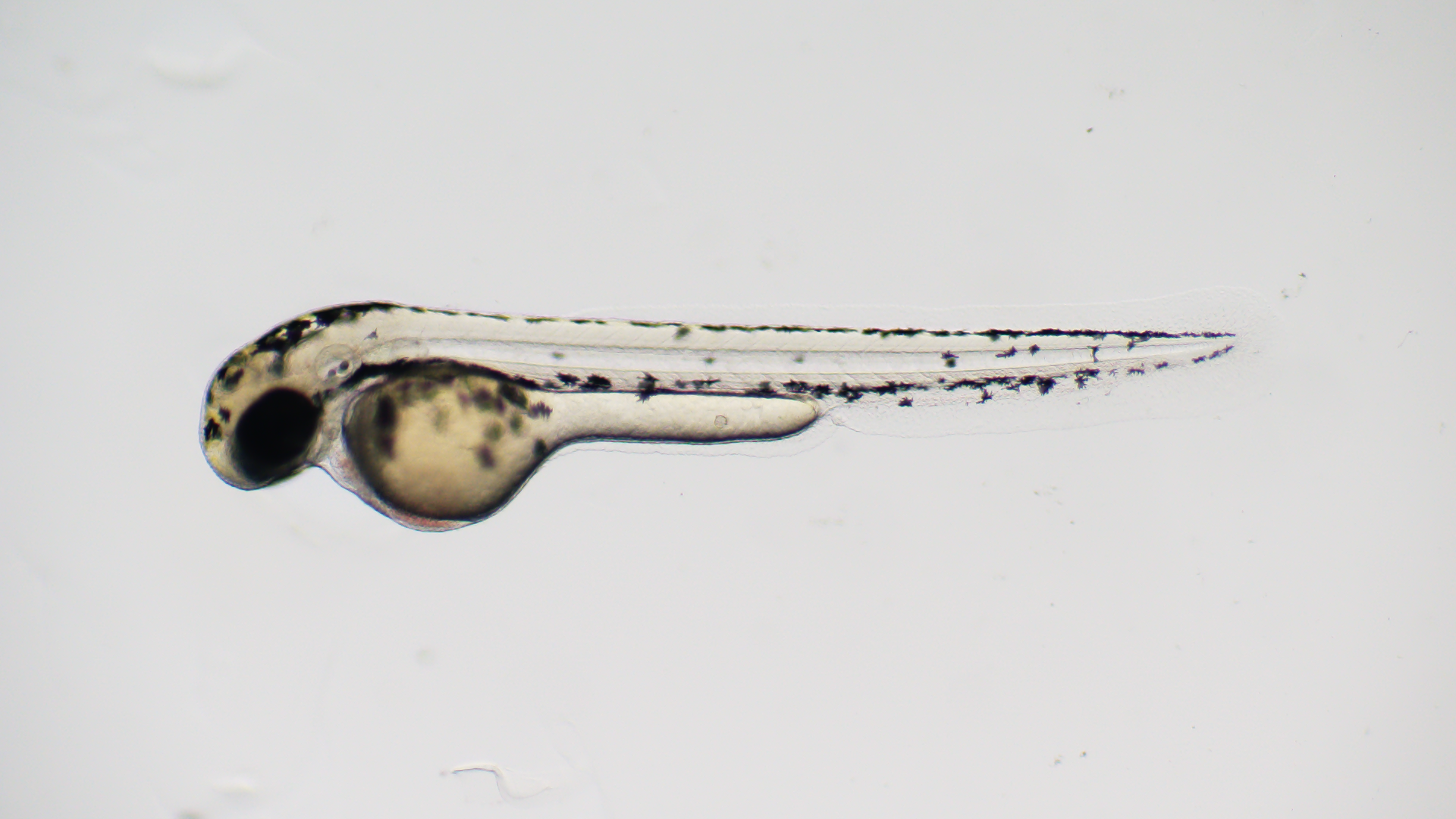

Supplement: Supplementary file 4 — Source data Fig. 4.1 [file 44321_2025_355_MOESM4_ESM.zip › A2V_2dpf.tif]

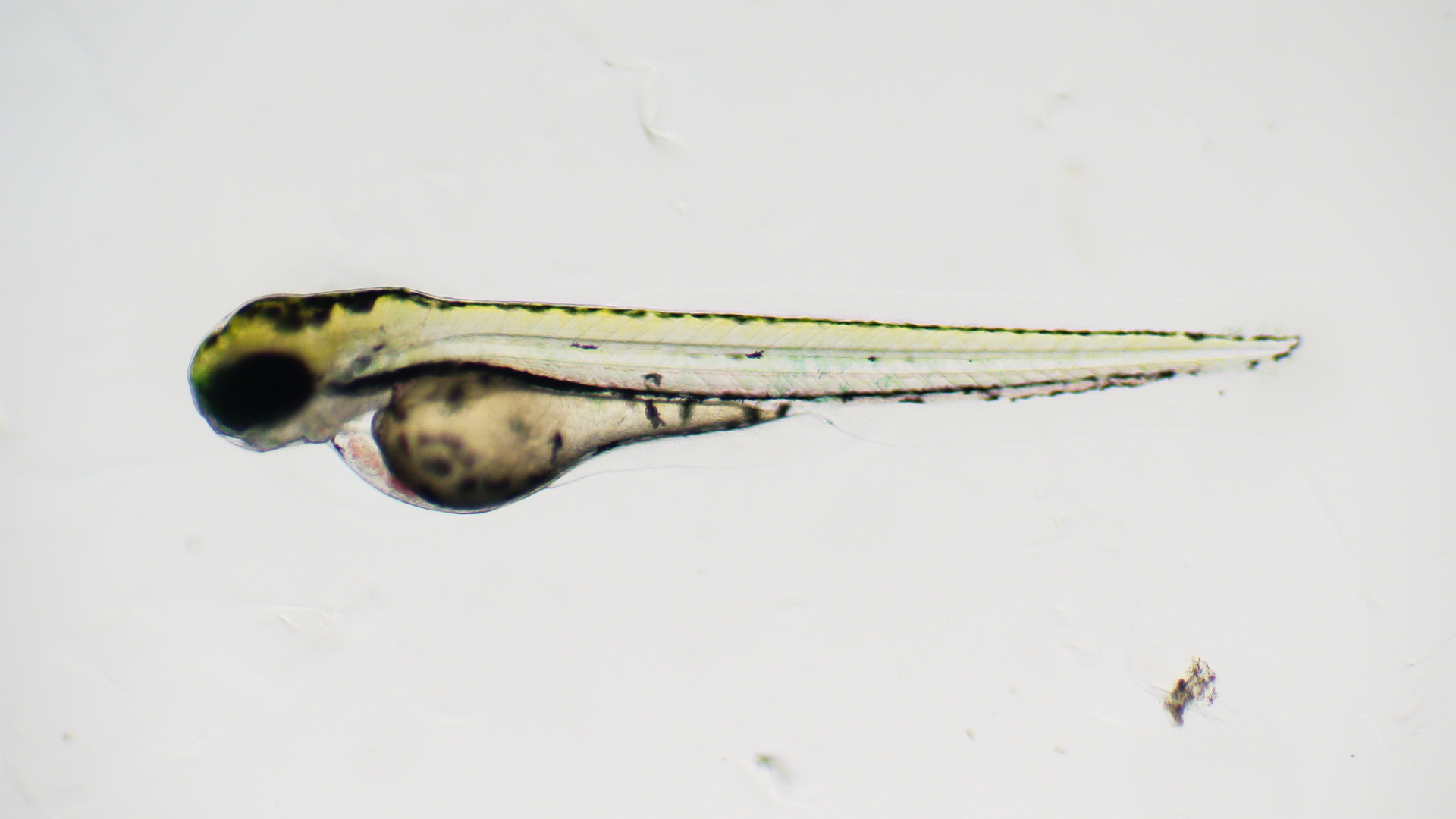

Supplement: Supplementary file 4 — Source data Fig. 4.1 [file 44321_2025_355_MOESM4_ESM.zip › A2V_3dpf.tif]

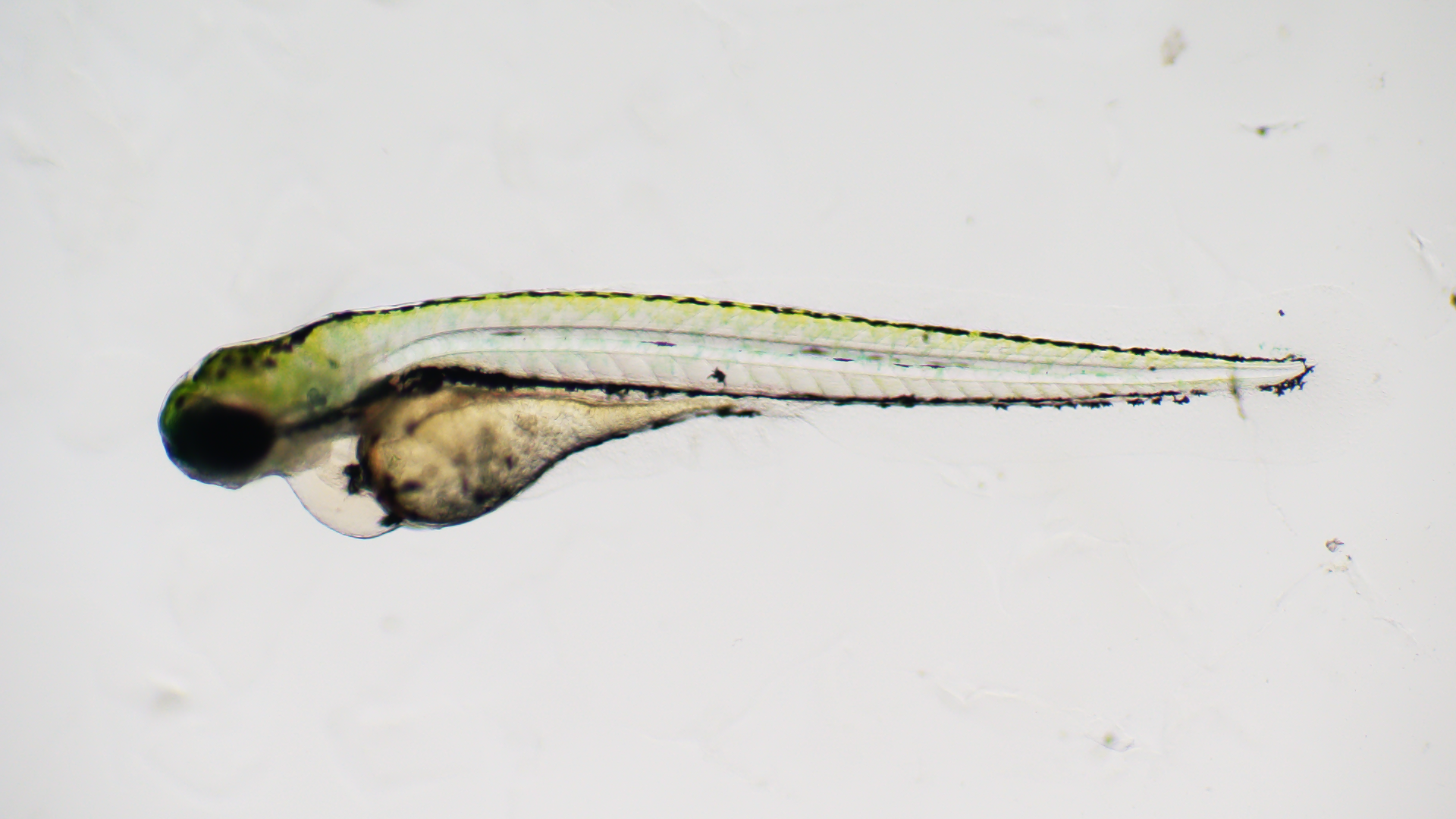

Supplement: Supplementary file 4 — Source data Fig. 4.1 [file 44321_2025_355_MOESM4_ESM.zip › A2V_4dpf.tif]

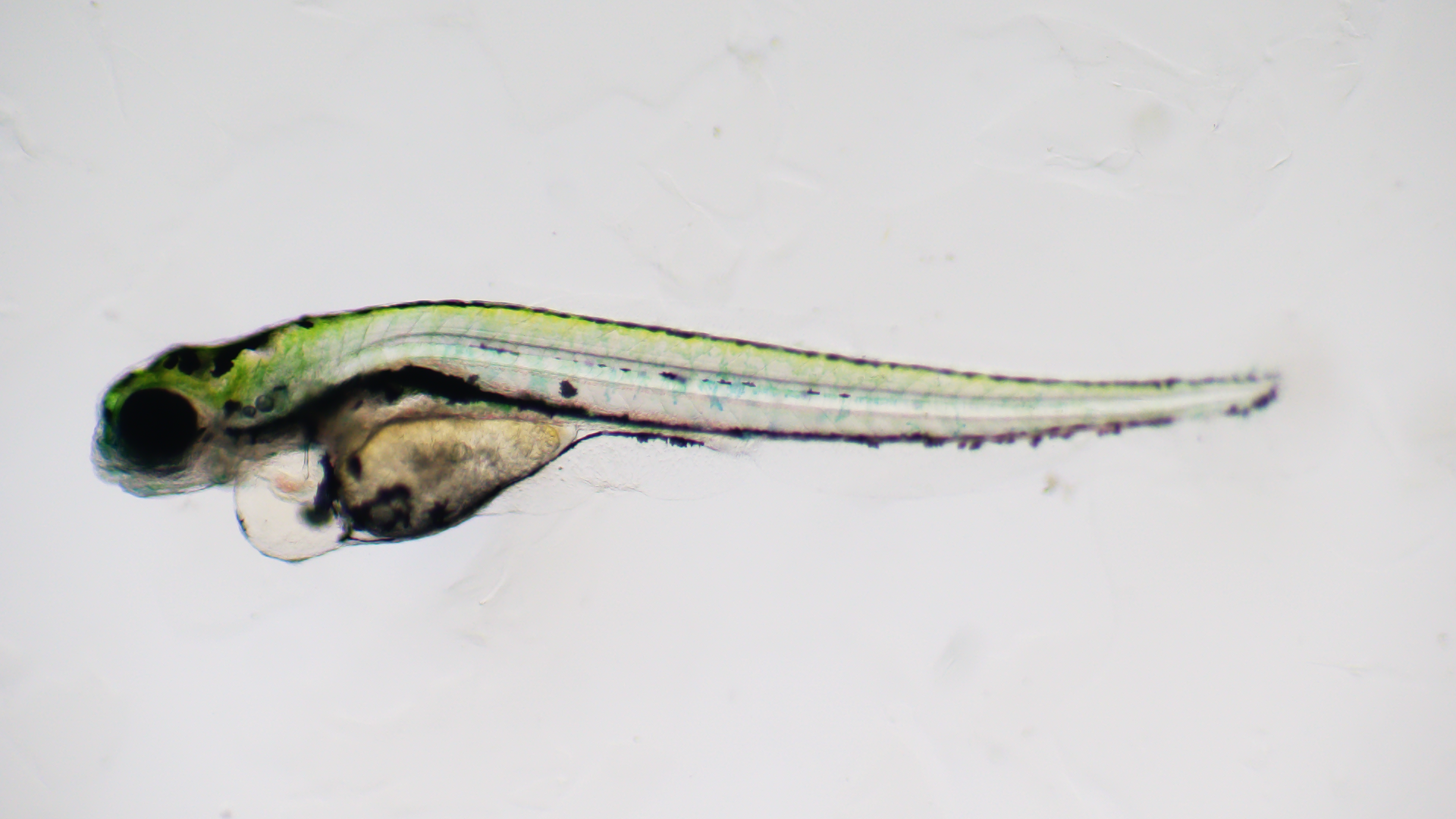

Supplement: Supplementary file 4 — Source data Fig. 4.1 [file 44321_2025_355_MOESM4_ESM.zip › A2V_5dpf.tif]

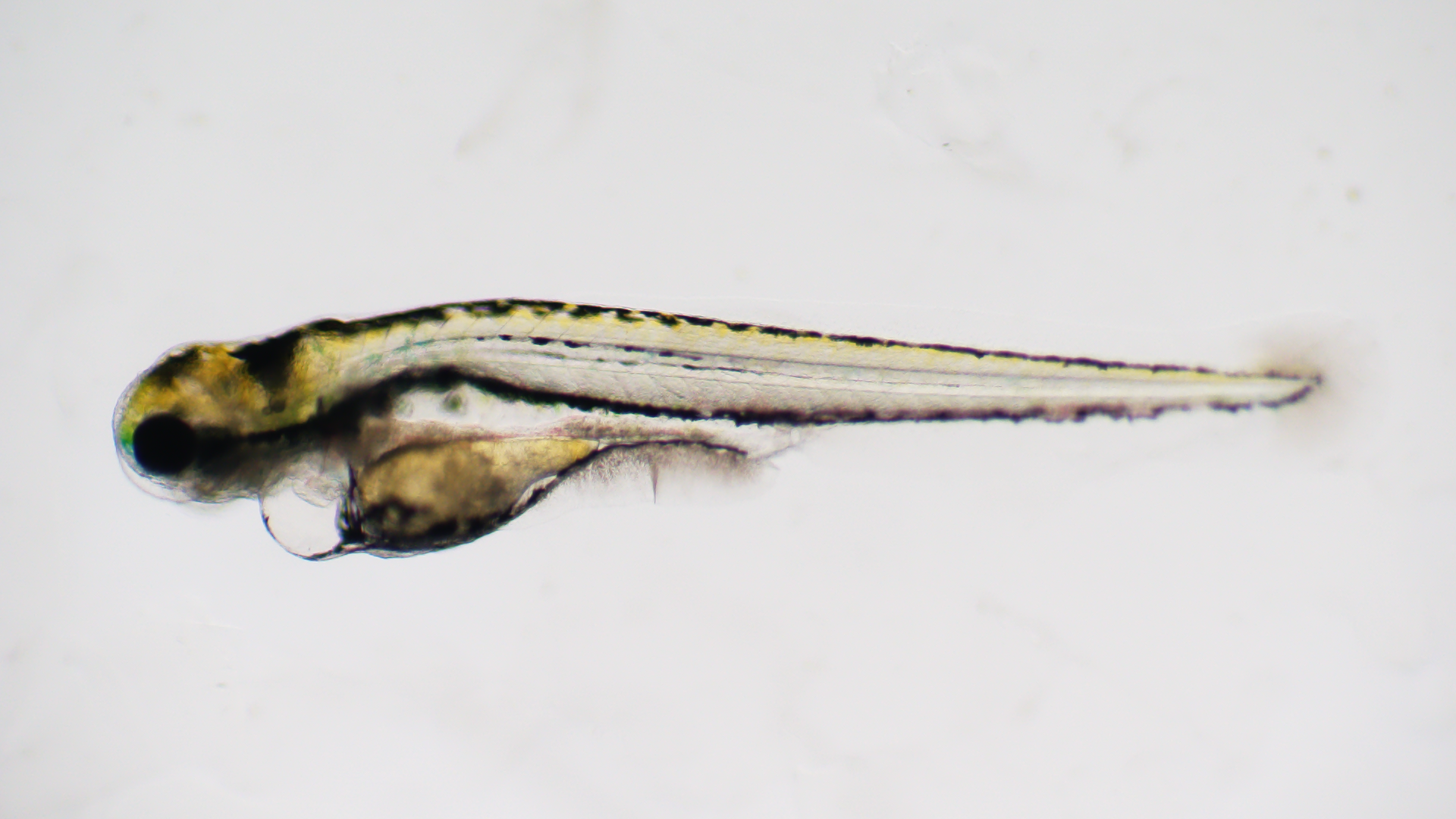

Supplement: Supplementary file 4 — Source data Fig. 4.1 [file 44321_2025_355_MOESM4_ESM.zip › A2V_6dpf.tif]

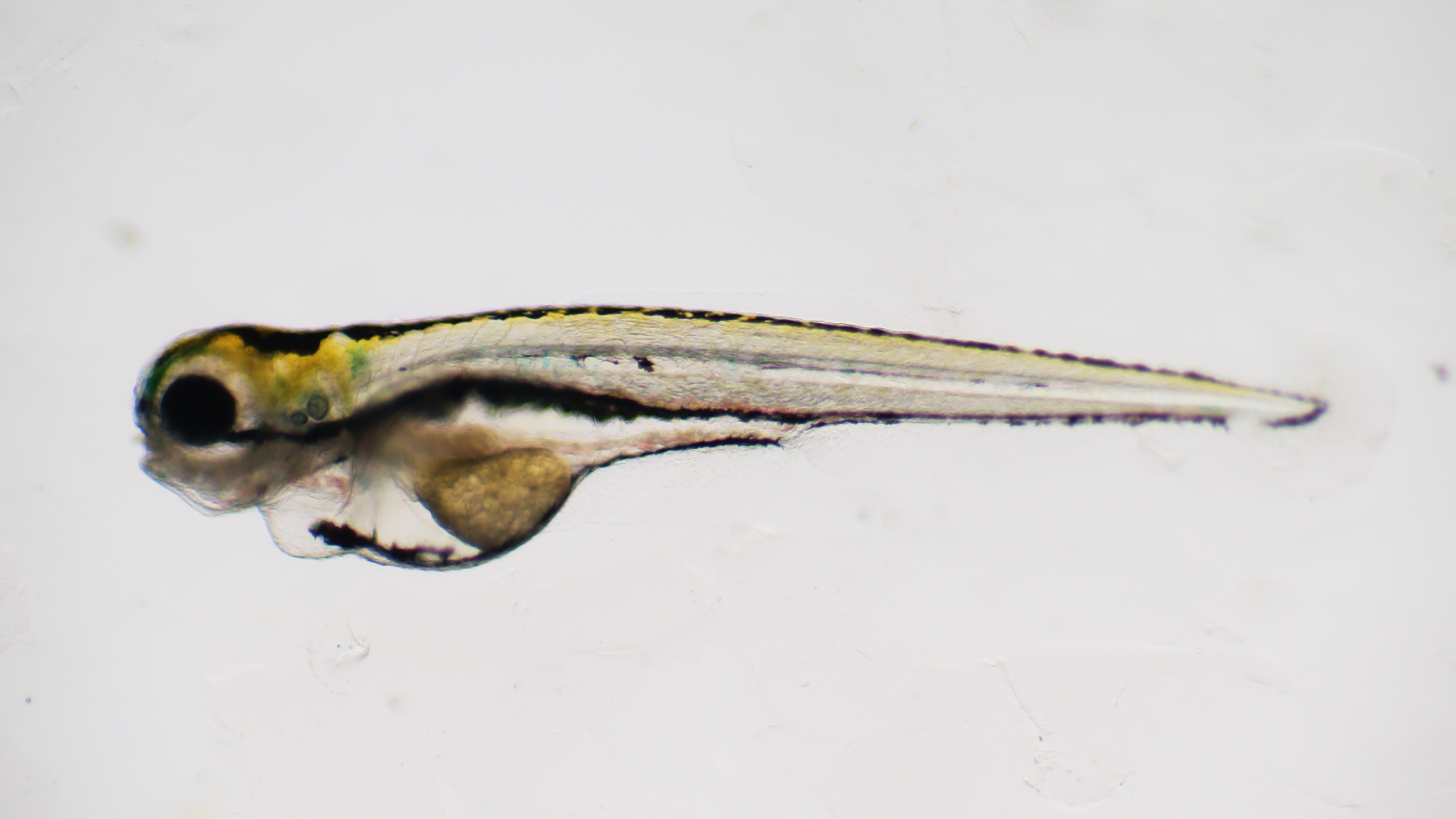

Supplement: Supplementary file 4 — Source data Fig. 4.1 [file 44321_2025_355_MOESM4_ESM.zip › A2V_7dpf.tif]

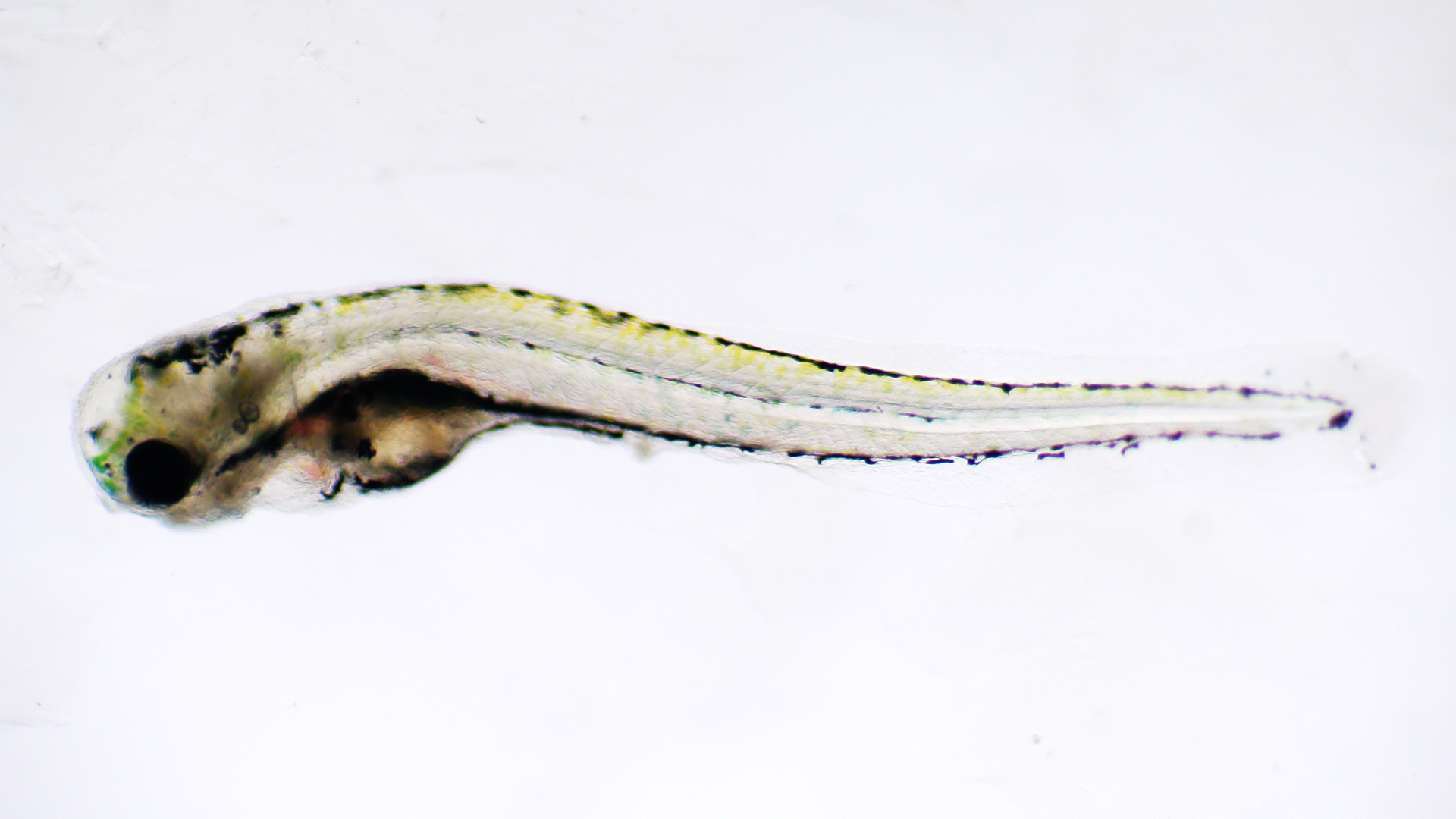

Supplement: Supplementary file 4 — Source data Fig. 4.1 [file 44321_2025_355_MOESM4_ESM.zip › A2V_8dpf.tif]

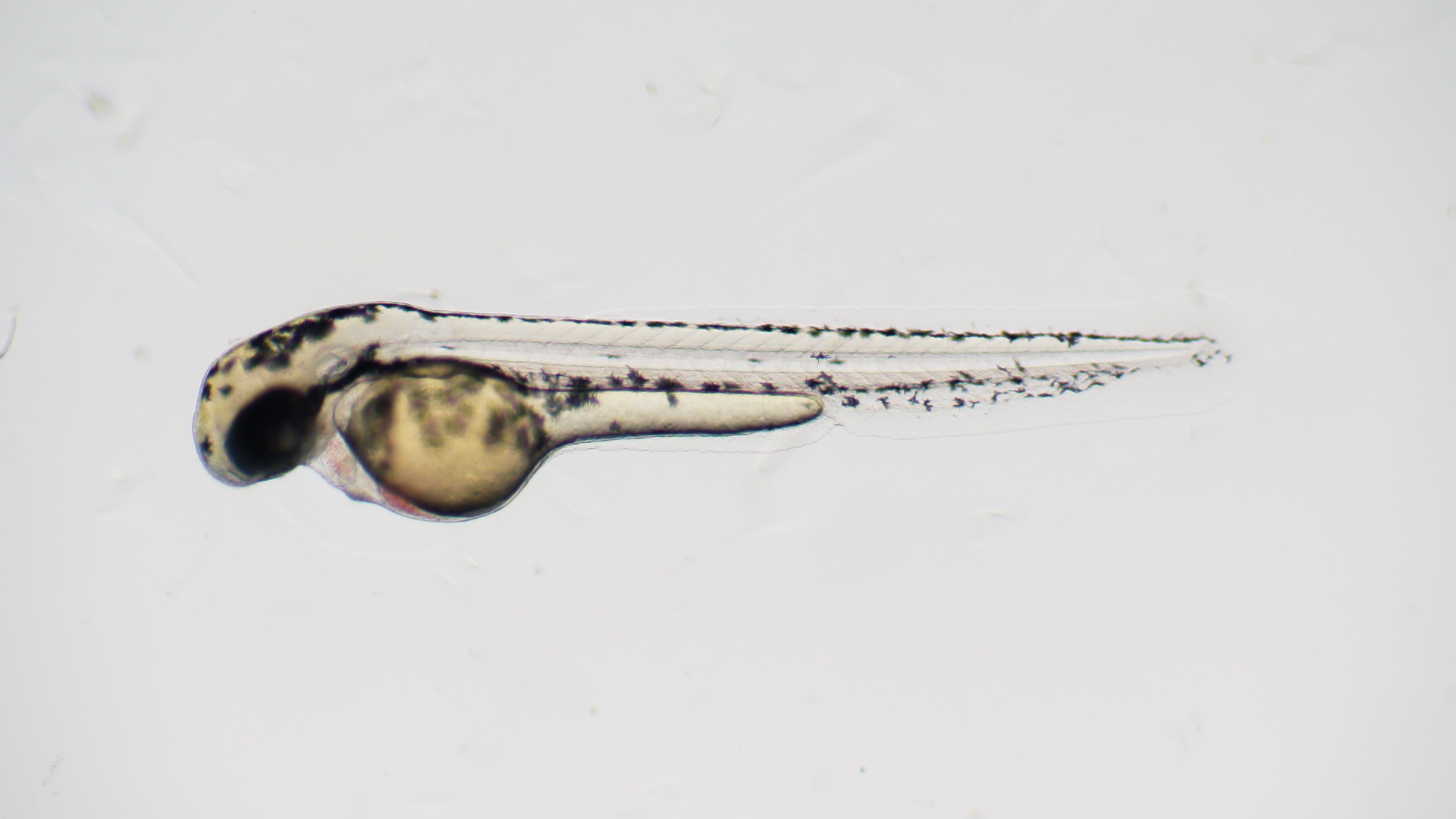

Supplement: Supplementary file 4 — Source data Fig. 4.1 [file 44321_2025_355_MOESM4_ESM.zip › control_2dpf.tif]

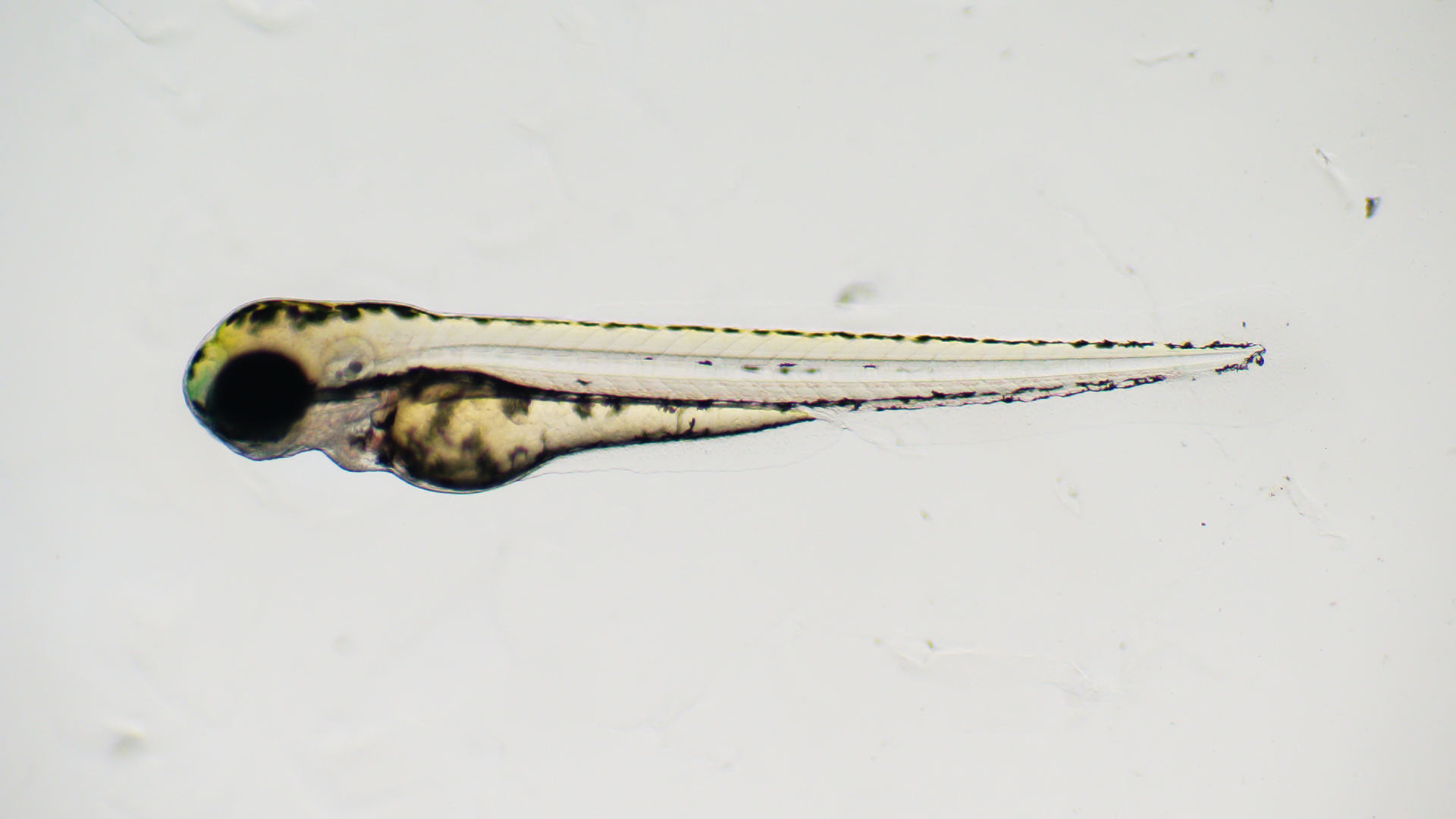

Supplement: Supplementary file 4 — Source data Fig. 4.1 [file 44321_2025_355_MOESM4_ESM.zip › control_3dpf.tif]

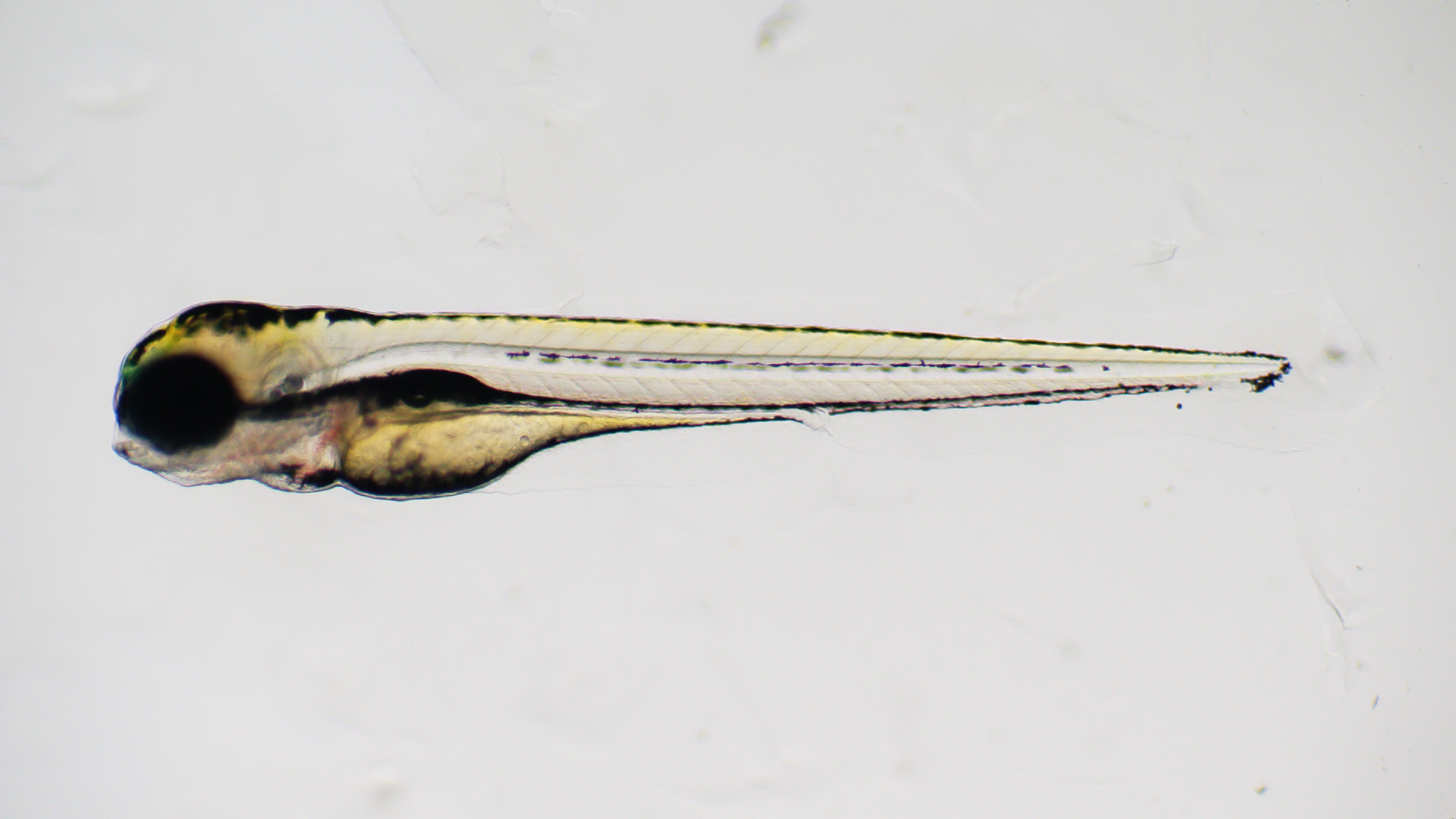

Supplement: Supplementary file 4 — Source data Fig. 4.1 [file 44321_2025_355_MOESM4_ESM.zip › control_4dpf.tif]

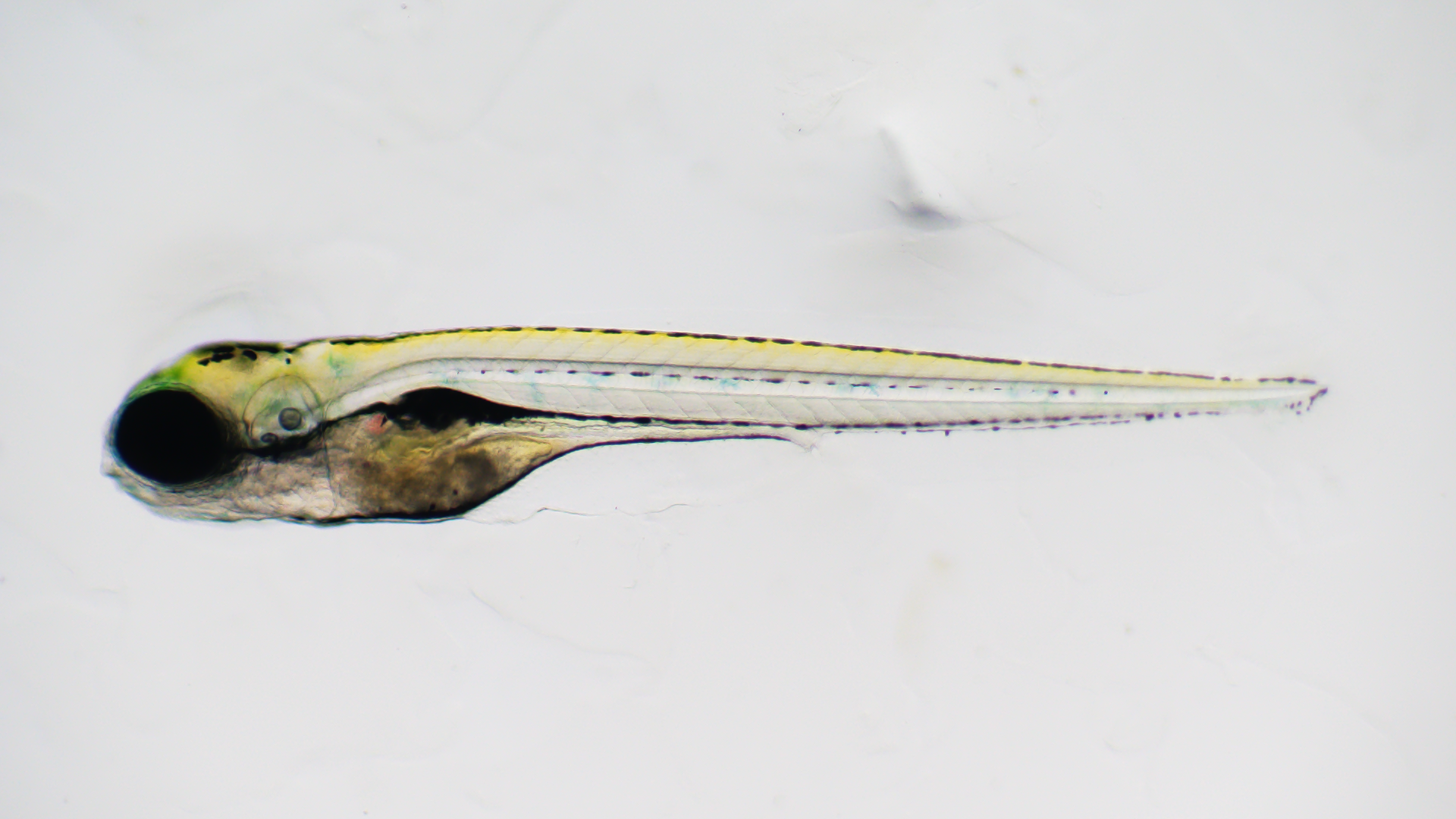

Supplement: Supplementary file 4 — Source data Fig. 4.1 [file 44321_2025_355_MOESM4_ESM.zip › control_5dpf.tif]

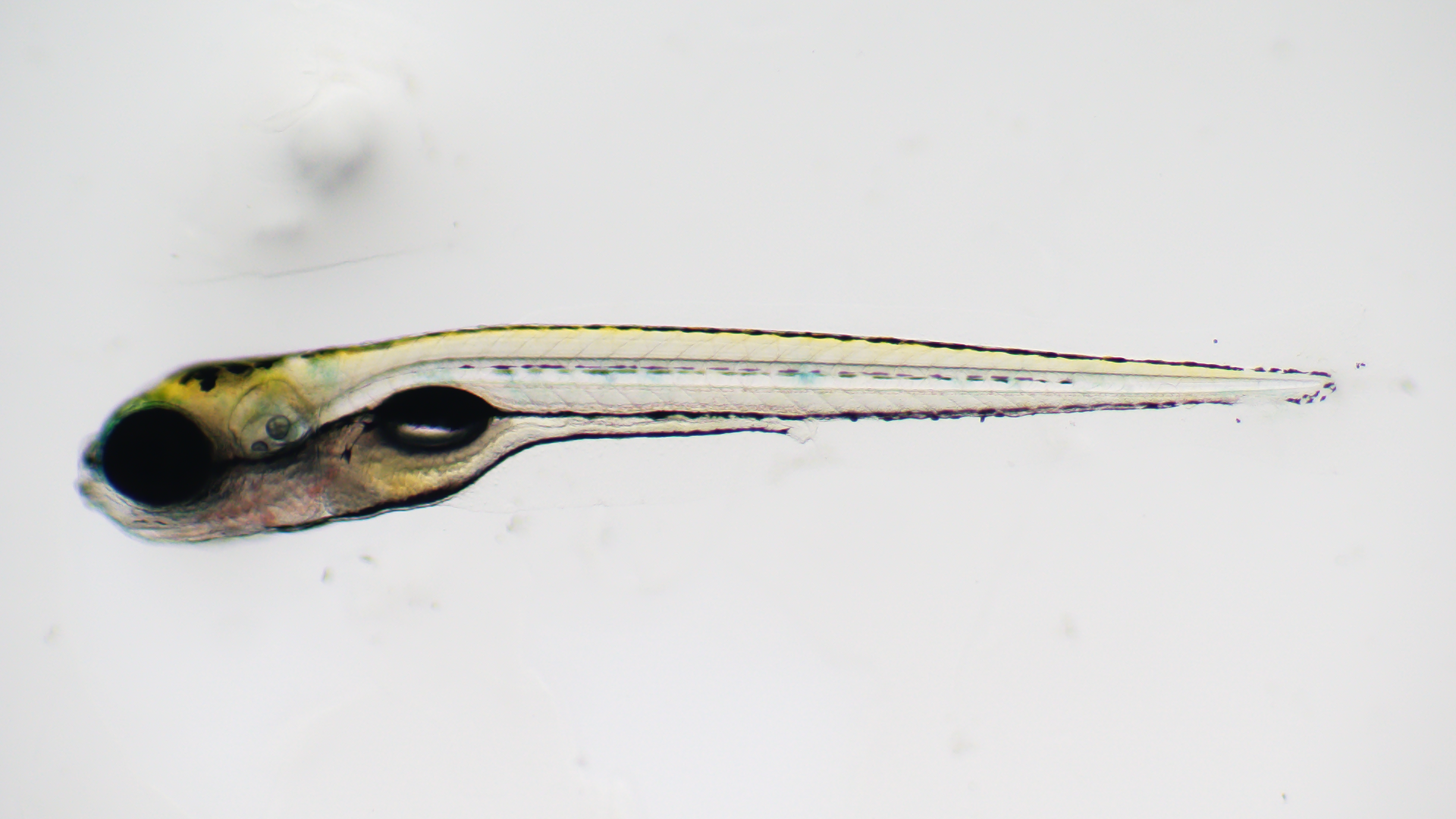

Supplement: Supplementary file 4 — Source data Fig. 4.1 [file 44321_2025_355_MOESM4_ESM.zip › control_6dpf.tif]

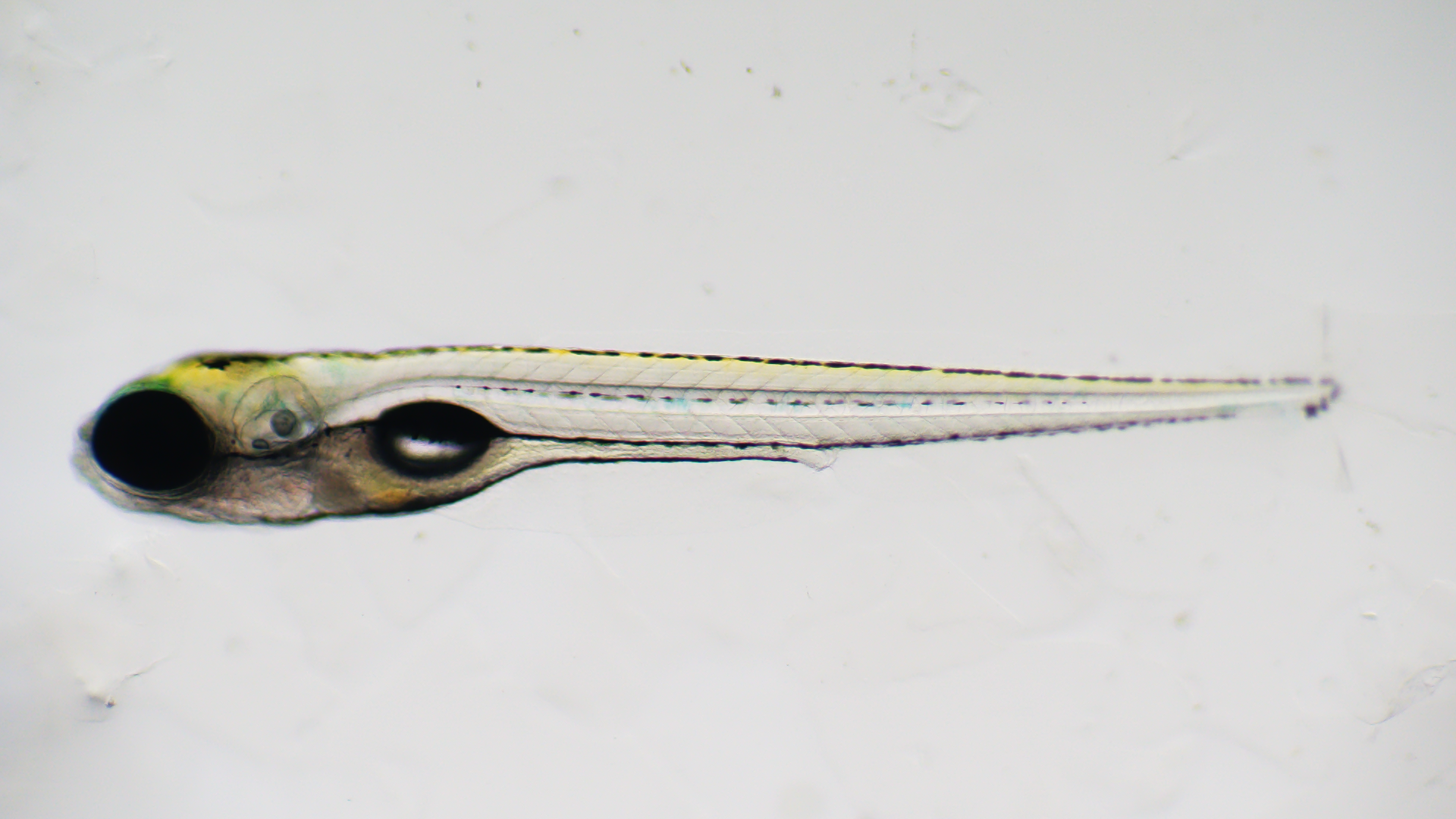

Supplement: Supplementary file 4 — Source data Fig. 4.1 [file 44321_2025_355_MOESM4_ESM.zip › control_7dpf.tif]

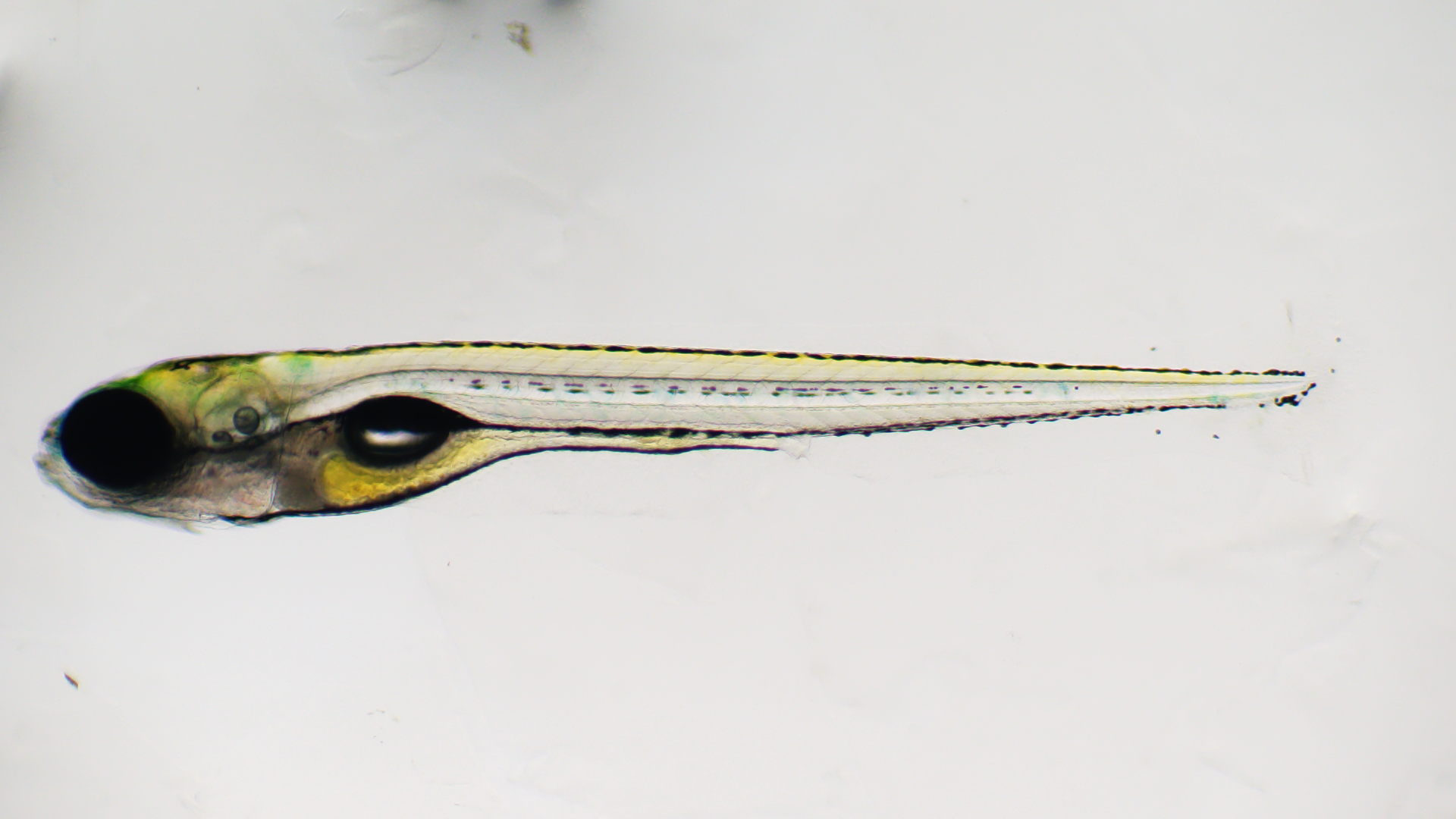

Supplement: Supplementary file 4 — Source data Fig. 4.1 [file 44321_2025_355_MOESM4_ESM.zip › control_8dpf.tif]
