# Supplementary figures and images for: Clinical relevance of zebrafish for gene variants testing. Proof-of-principle with SMN1/SMA (part 2 of 2)
Source: EMBO Mol Med. 2025 Dec 15;18(1):41–54. doi: 10.1038/s44321-025-00355-8 (PMC12808650; doi:10.1038/s44321-025-00355-8)

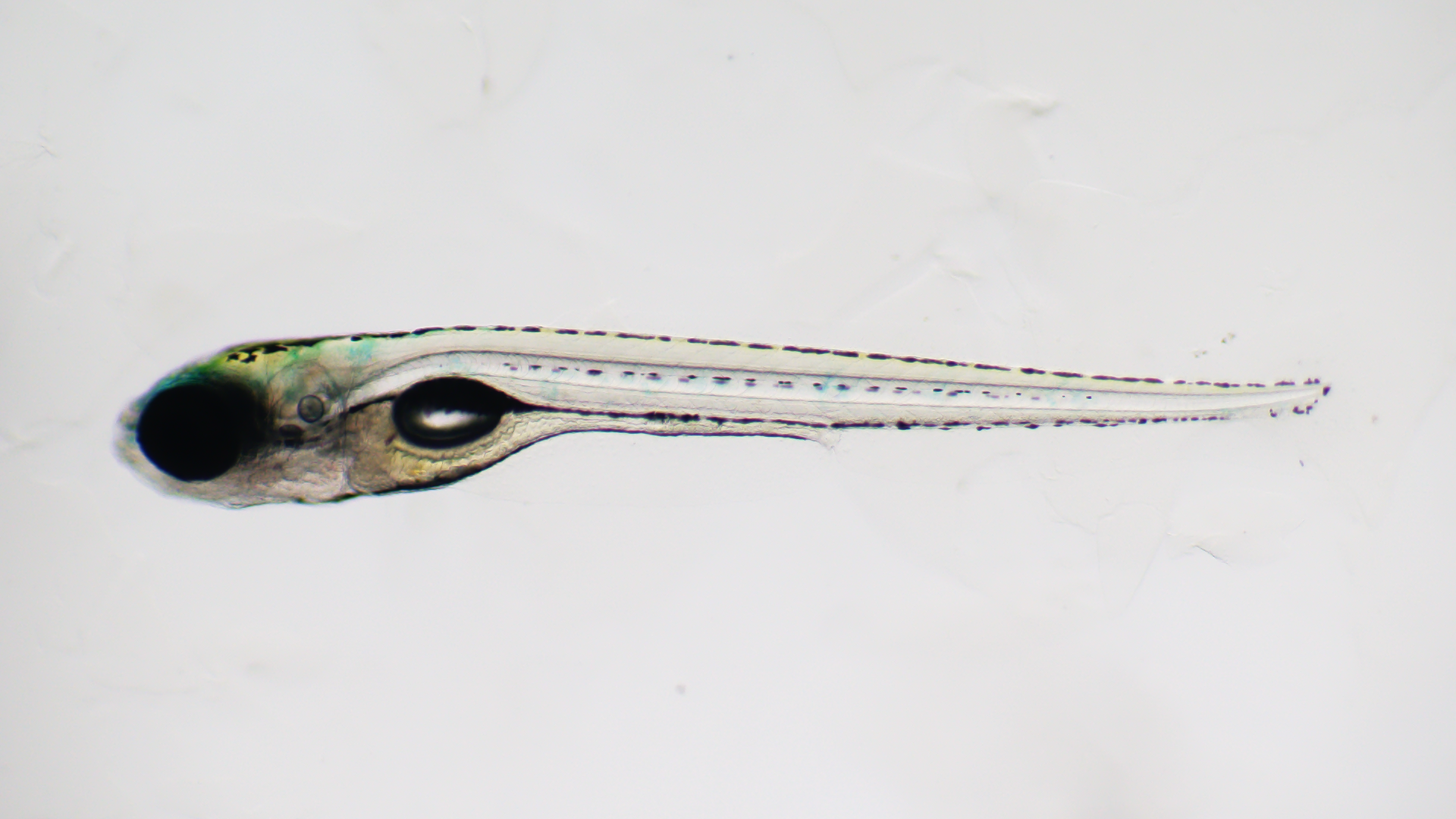

Supplement: Supplementary file 4 — Source data Fig. 4.1 [file 44321_2025_355_MOESM4_ESM.zip › control_9dpf.tif]

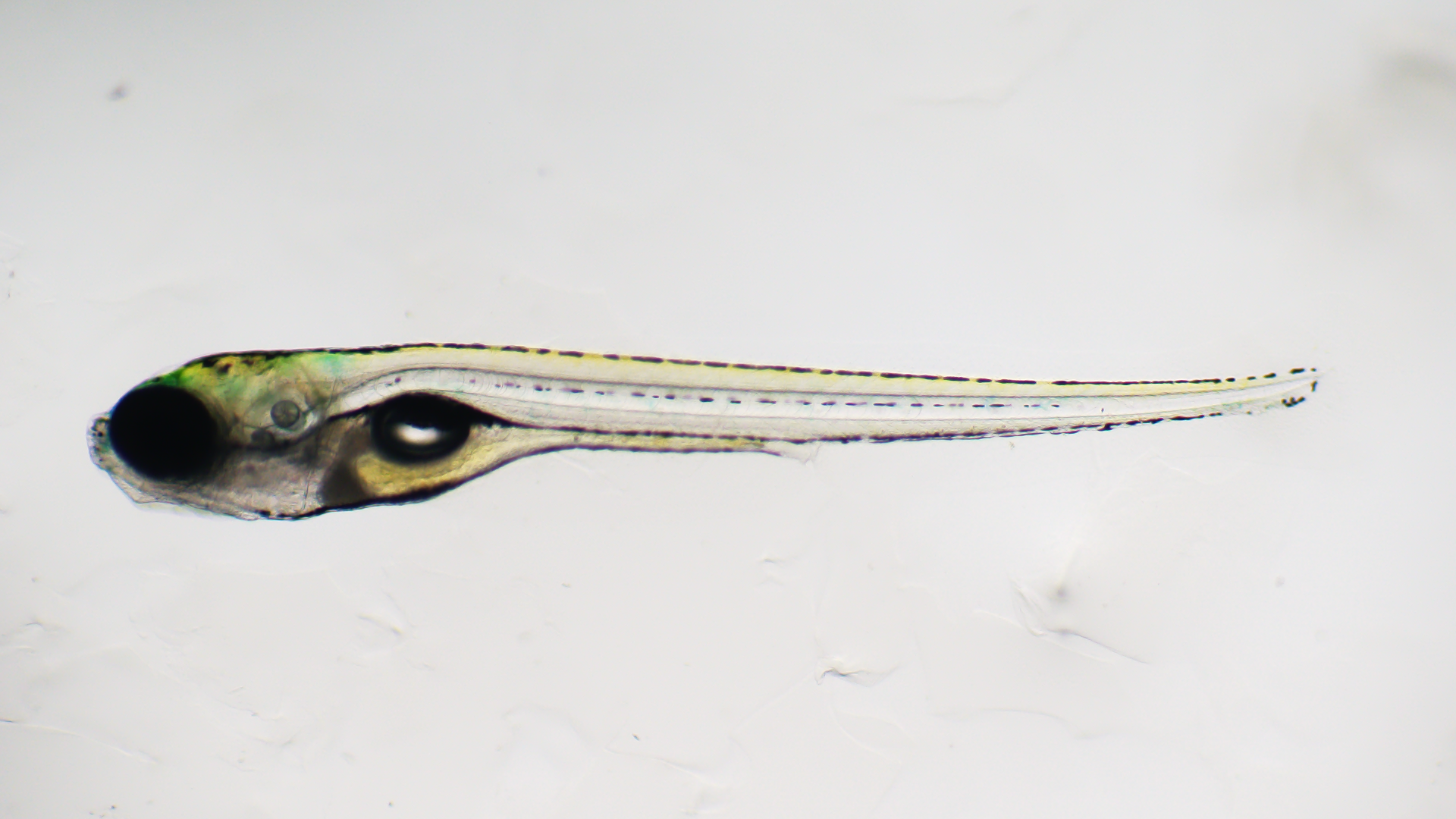

Supplement: Supplementary file 4 — Source data Fig. 4.1 [file 44321_2025_355_MOESM4_ESM.zip › control_10dpf.tif]

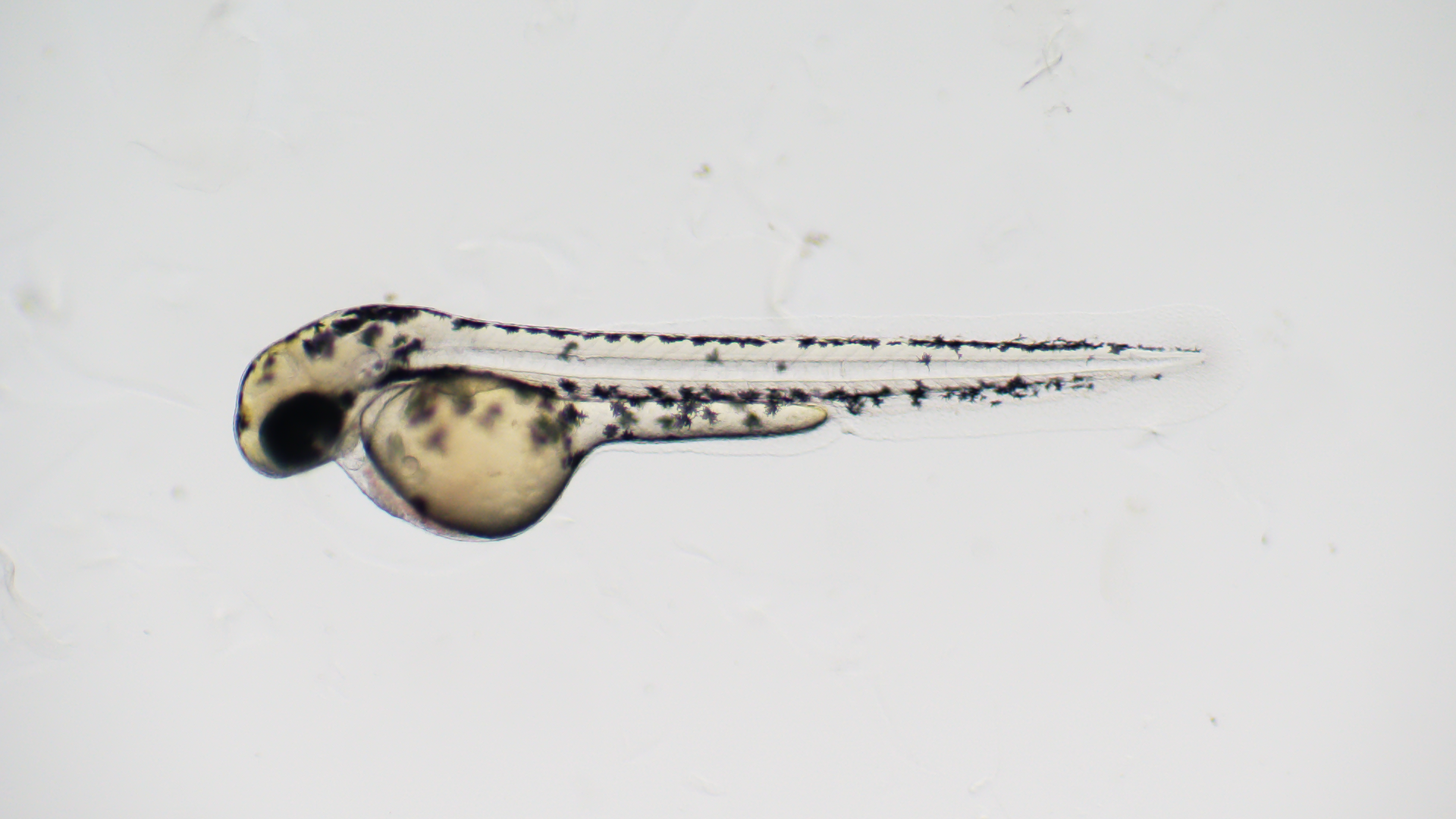

Supplement: Supplementary file 5 — Source data Fig. 4.2 [file 44321_2025_355_MOESM5_ESM.zip › D44V_2dpf.tif]

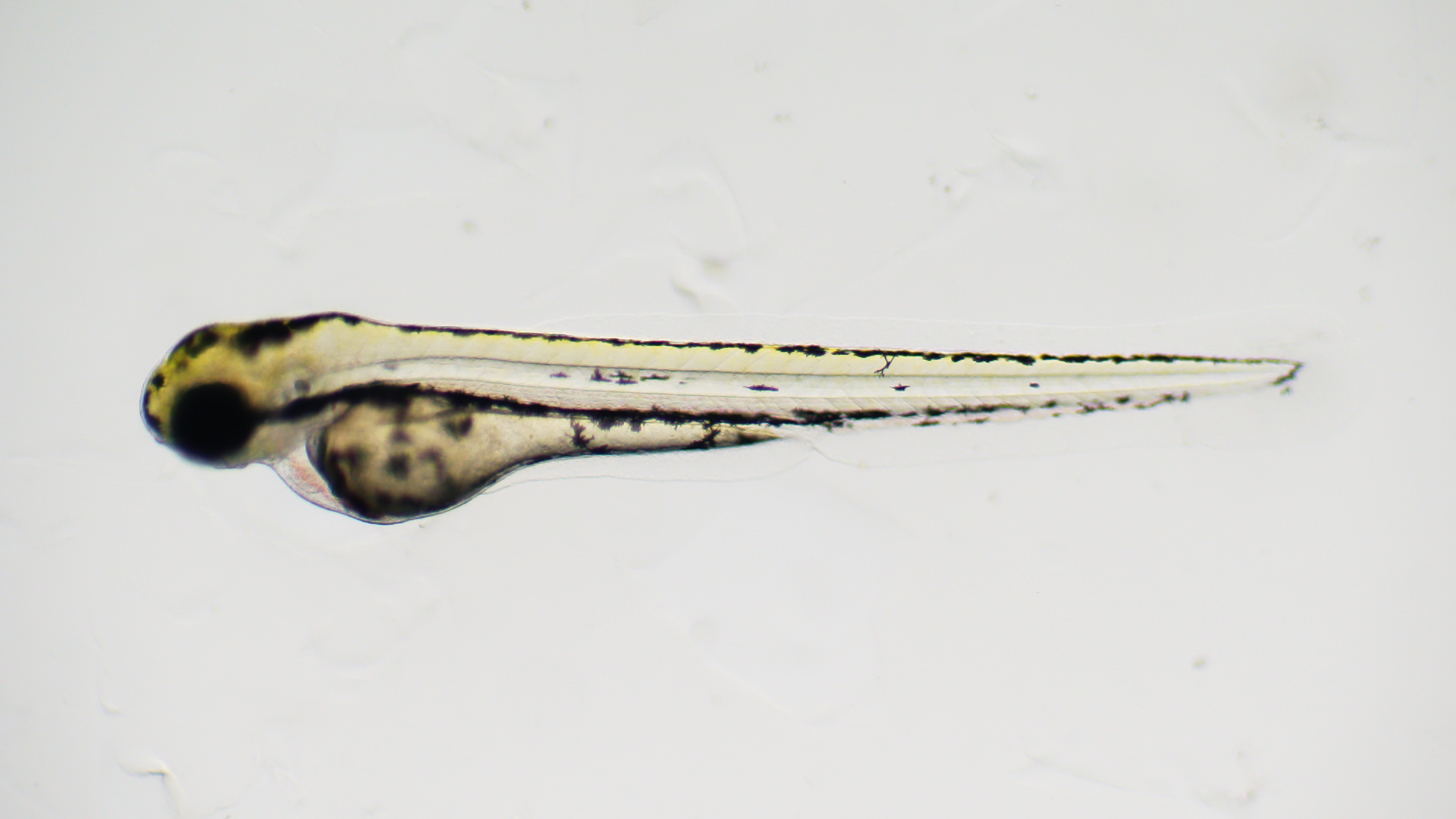

Supplement: Supplementary file 5 — Source data Fig. 4.2 [file 44321_2025_355_MOESM5_ESM.zip › D44V_3dpf.tif]

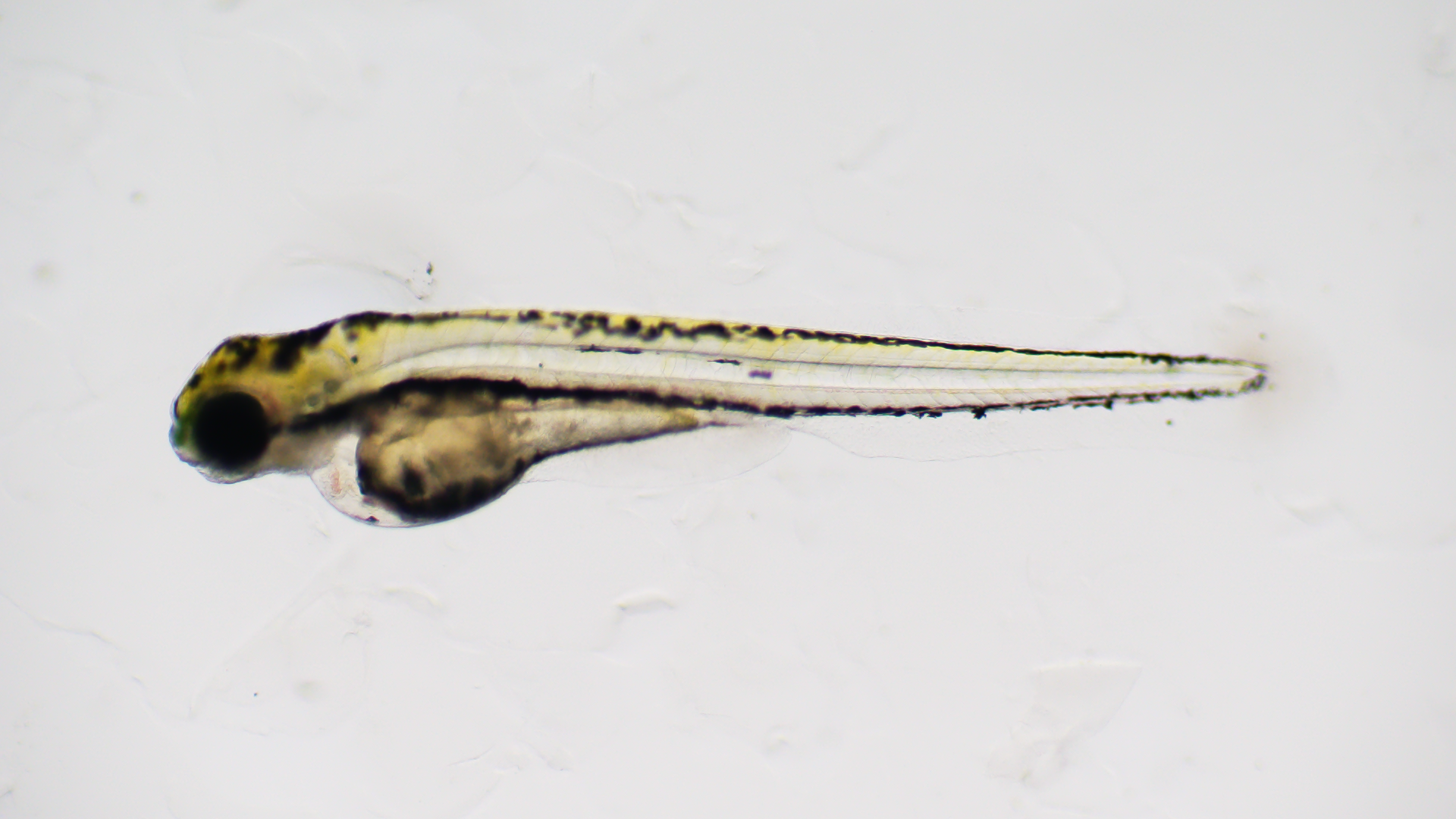

Supplement: Supplementary file 5 — Source data Fig. 4.2 [file 44321_2025_355_MOESM5_ESM.zip › D44V_4dpf.tif]

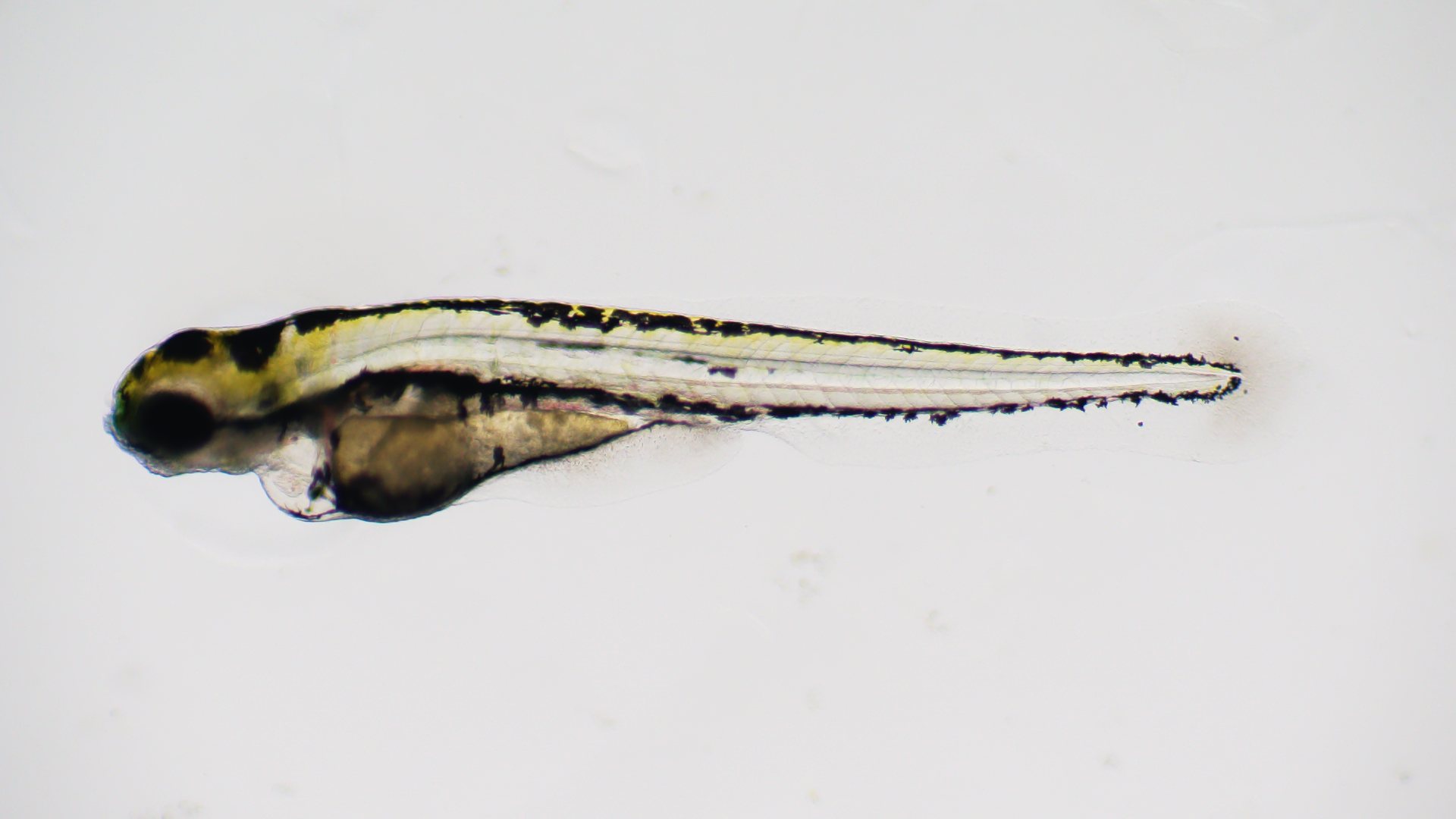

Supplement: Supplementary file 5 — Source data Fig. 4.2 [file 44321_2025_355_MOESM5_ESM.zip › D44V_5dpf.tif]

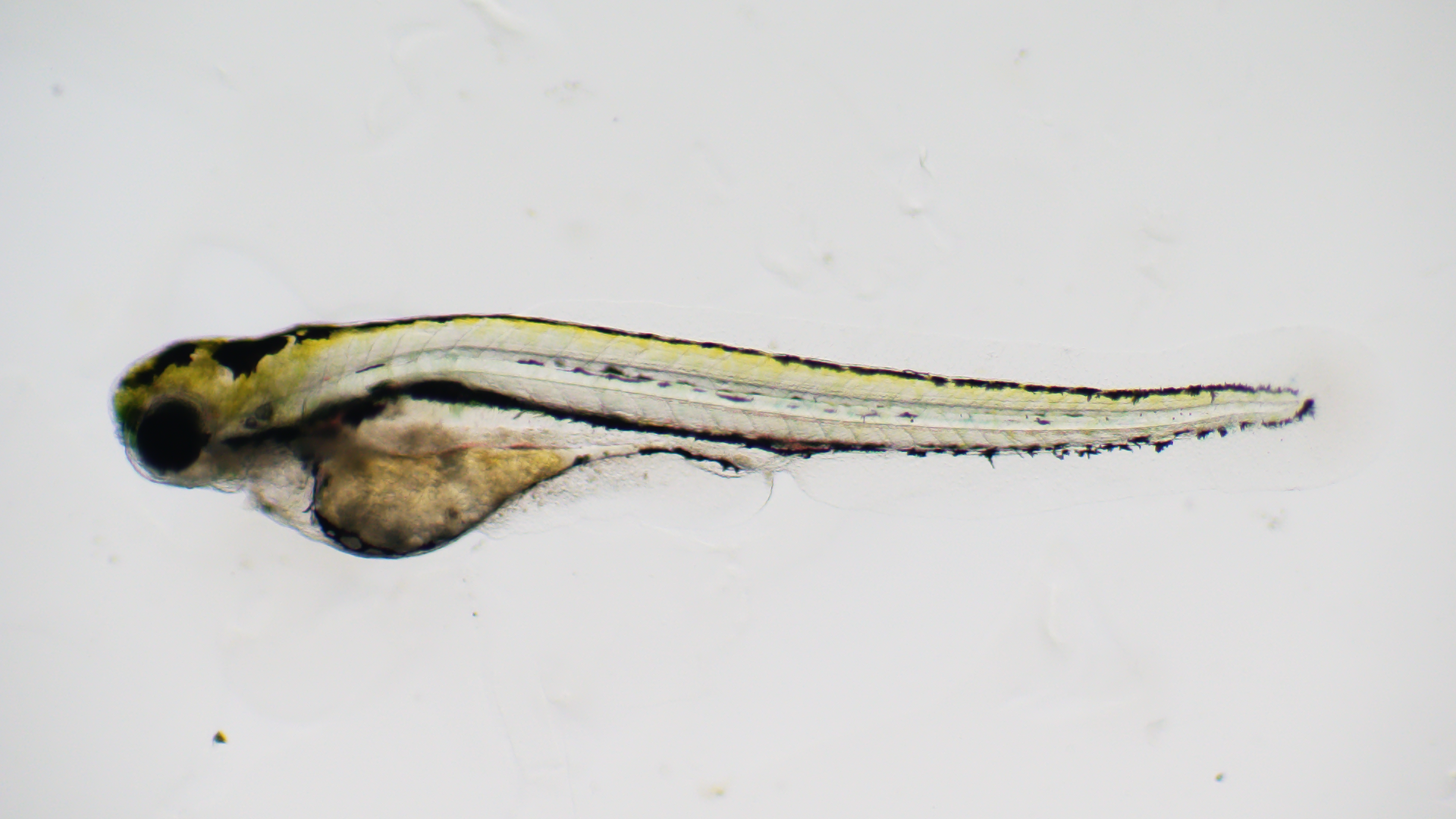

Supplement: Supplementary file 5 — Source data Fig. 4.2 [file 44321_2025_355_MOESM5_ESM.zip › D44V_6dpf.tif]

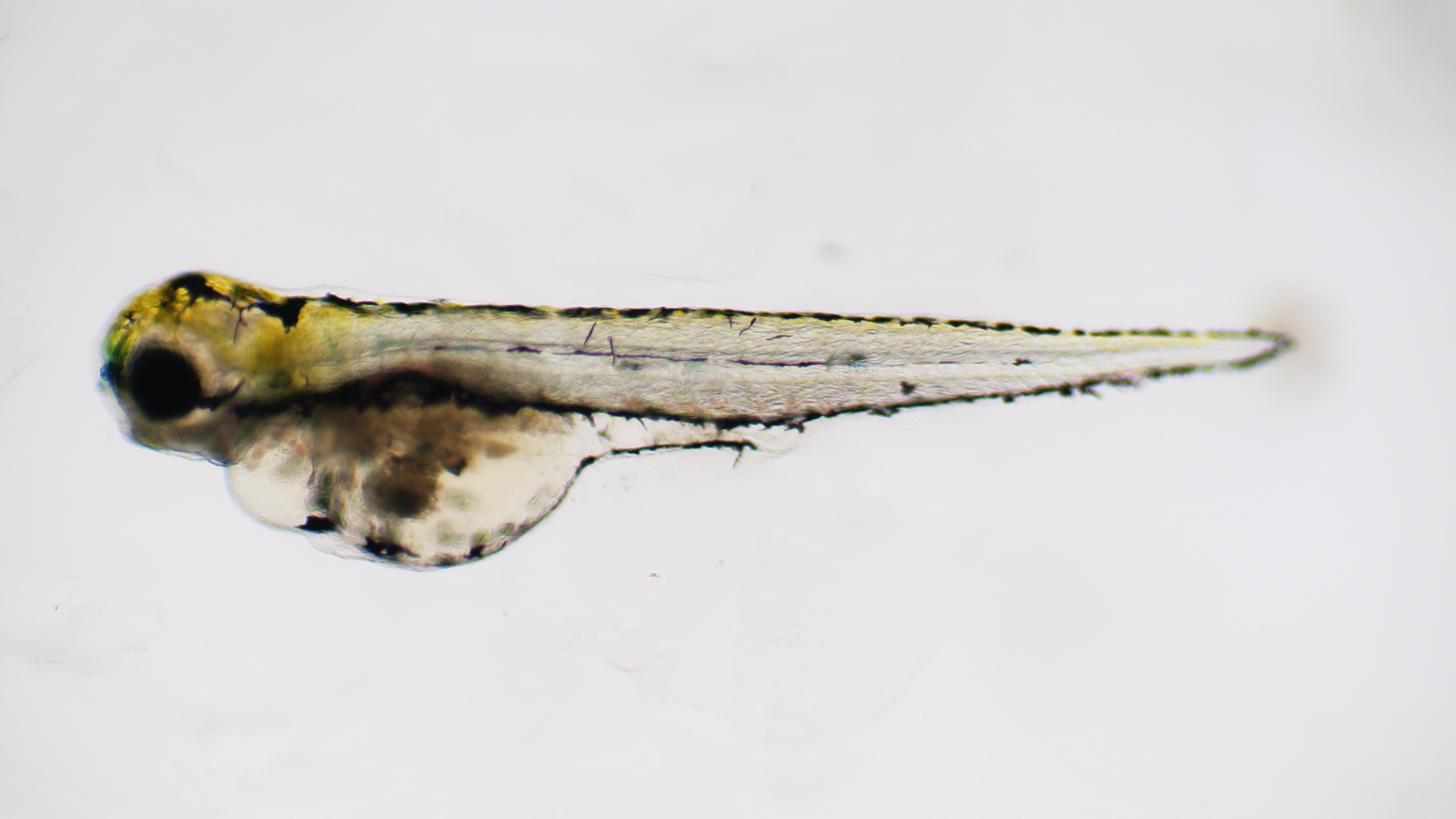

Supplement: Supplementary file 5 — Source data Fig. 4.2 [file 44321_2025_355_MOESM5_ESM.zip › D44V_7dpf.tif]

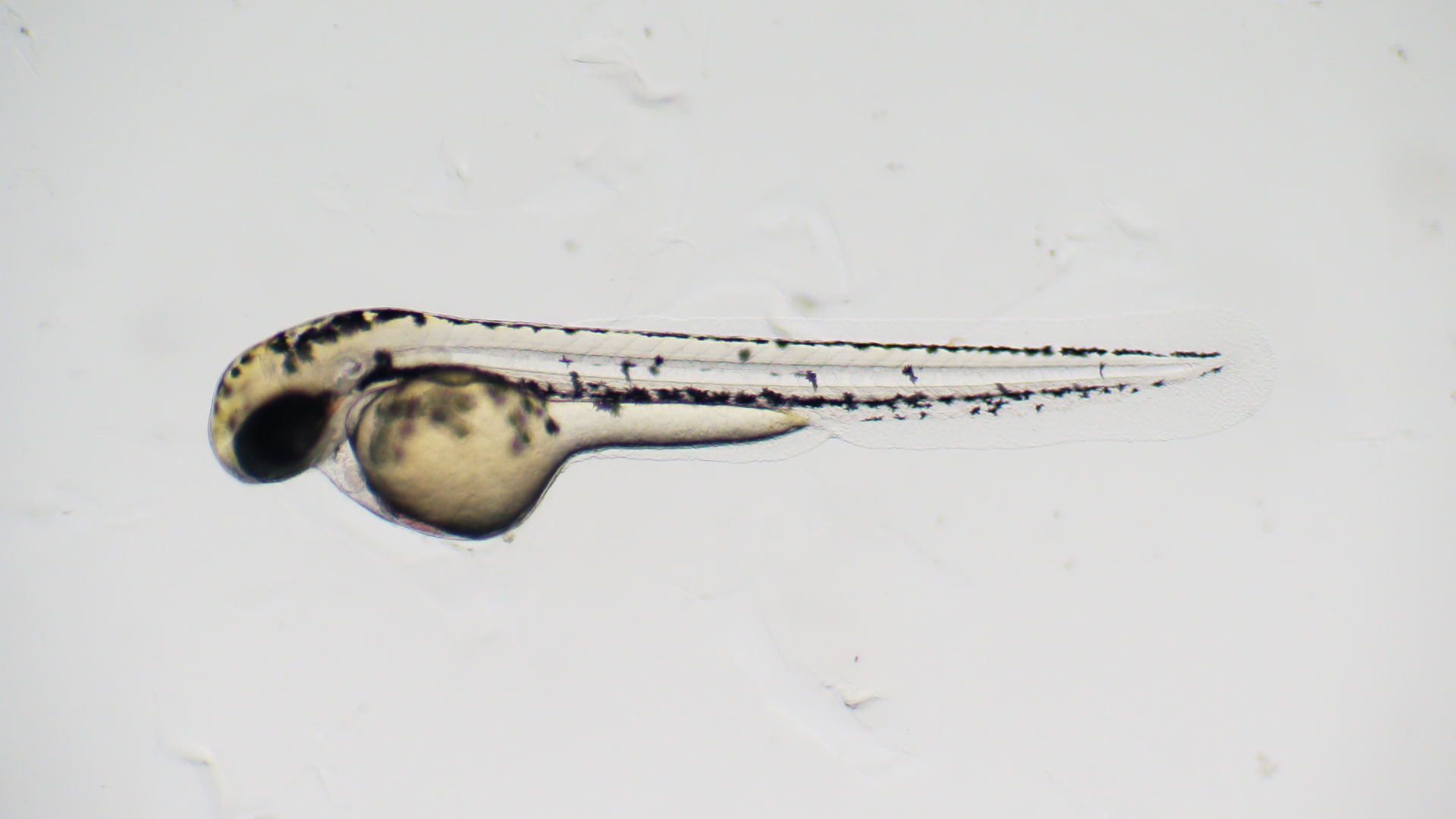

Supplement: Supplementary file 5 — Source data Fig. 4.2 [file 44321_2025_355_MOESM5_ESM.zip › non-path_1_2dpf.tif]

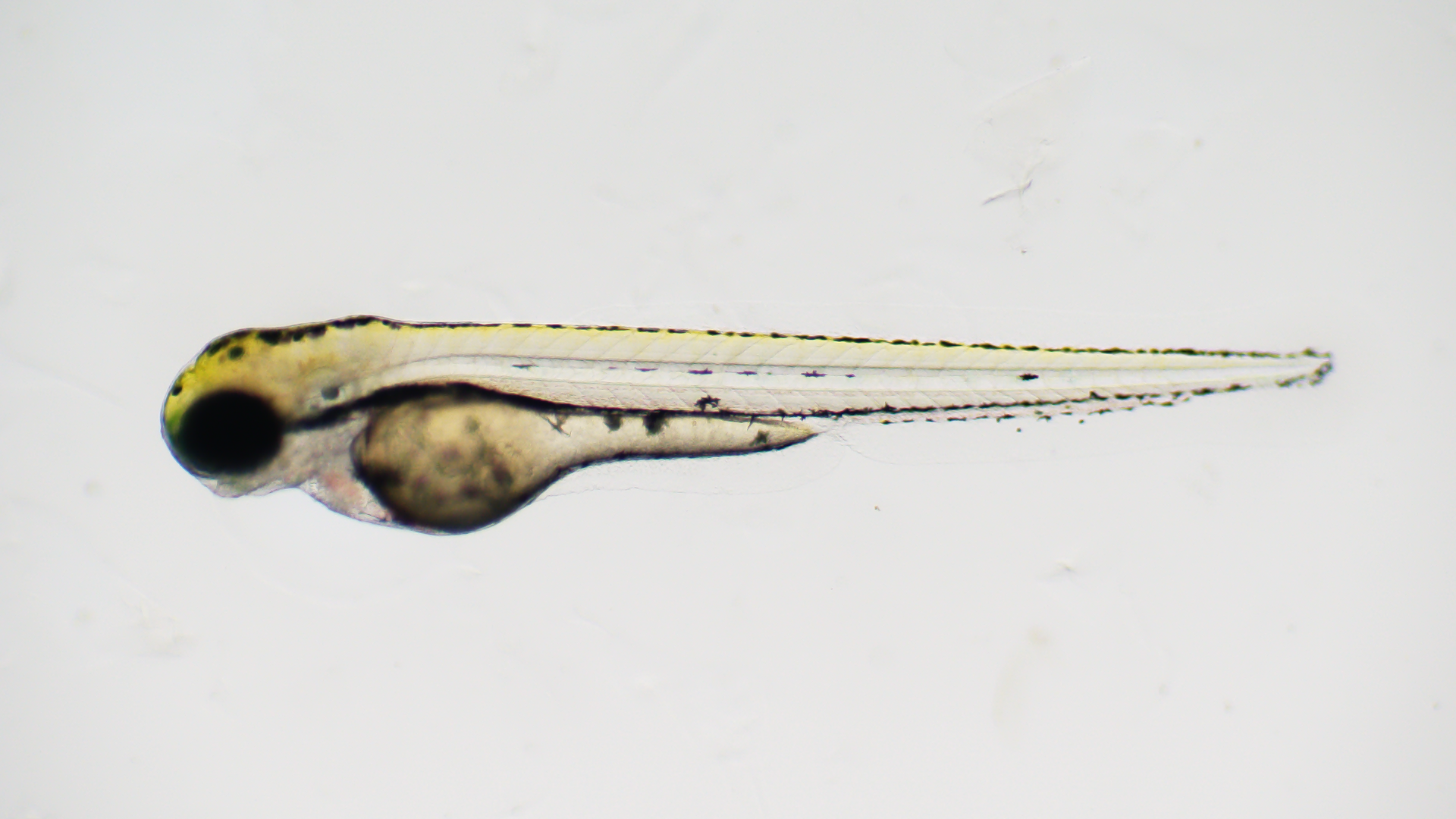

Supplement: Supplementary file 5 — Source data Fig. 4.2 [file 44321_2025_355_MOESM5_ESM.zip › non-path_1_3dpf.tif]

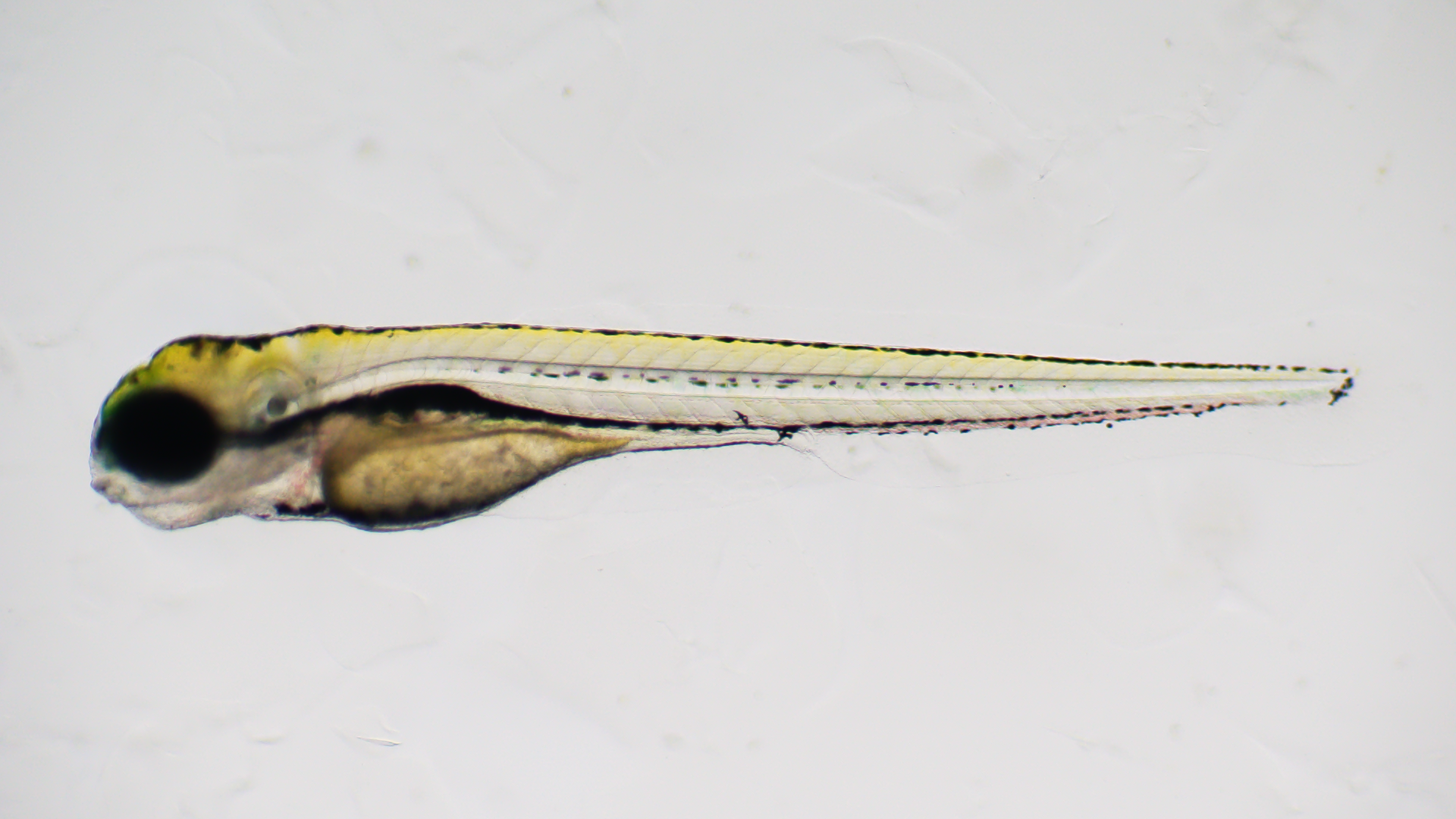

Supplement: Supplementary file 5 — Source data Fig. 4.2 [file 44321_2025_355_MOESM5_ESM.zip › non-path_1_4dpf.tif]

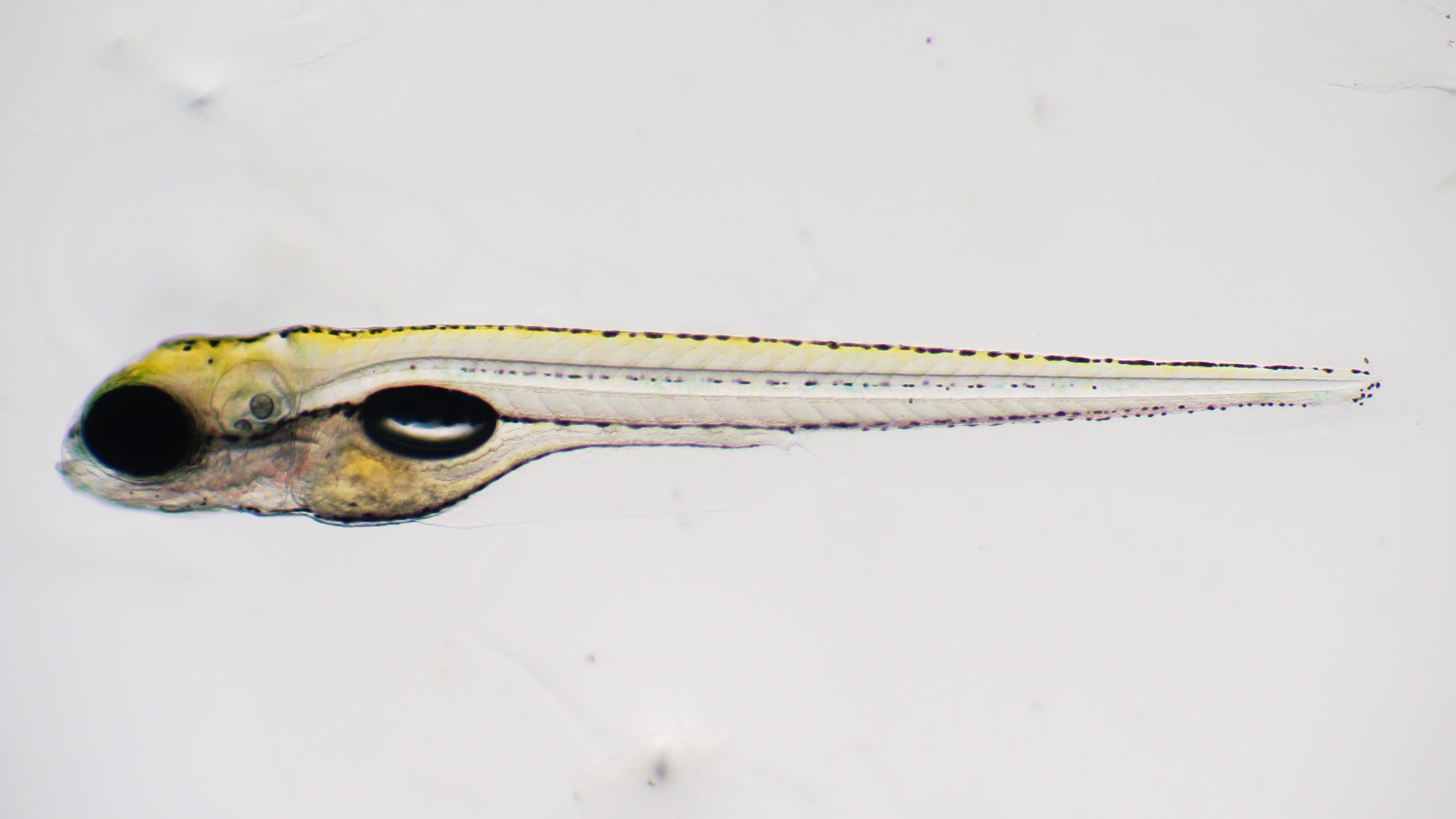

Supplement: Supplementary file 5 — Source data Fig. 4.2 [file 44321_2025_355_MOESM5_ESM.zip › non-path_1_5dpf.tif]

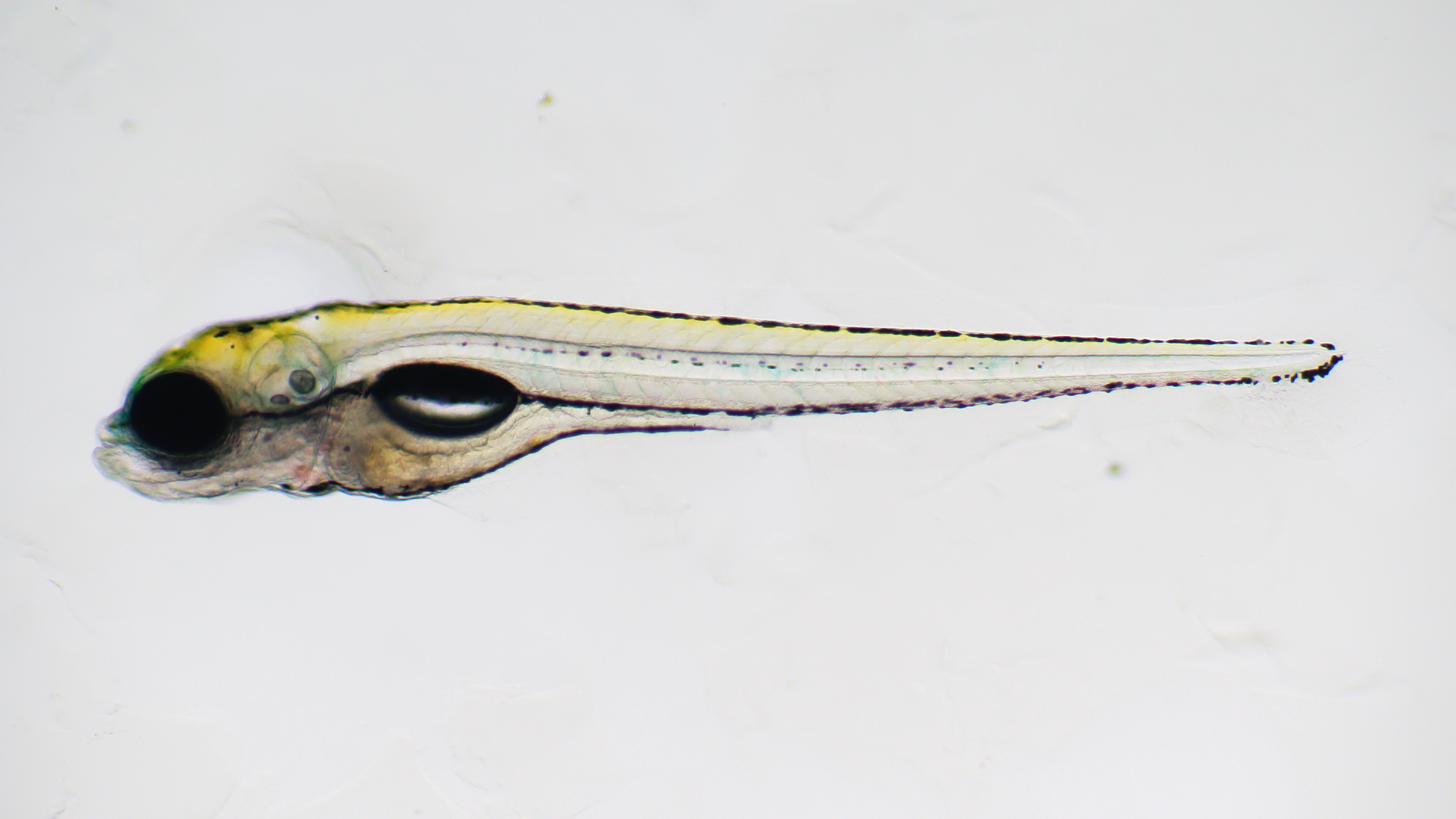

Supplement: Supplementary file 5 — Source data Fig. 4.2 [file 44321_2025_355_MOESM5_ESM.zip › non-path_1_6dpf.tif]

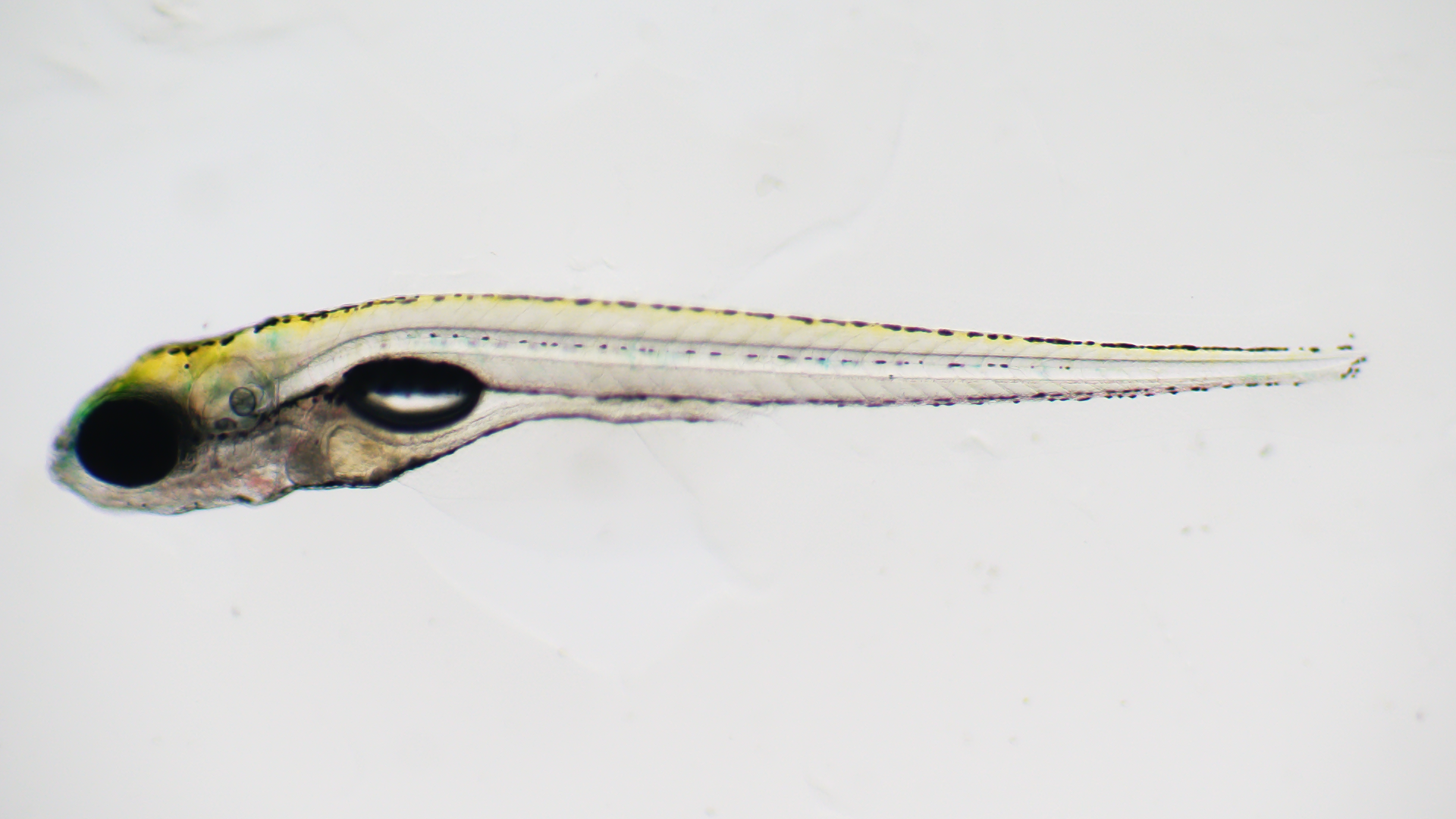

Supplement: Supplementary file 5 — Source data Fig. 4.2 [file 44321_2025_355_MOESM5_ESM.zip › non-path_1_7dpf.tif]

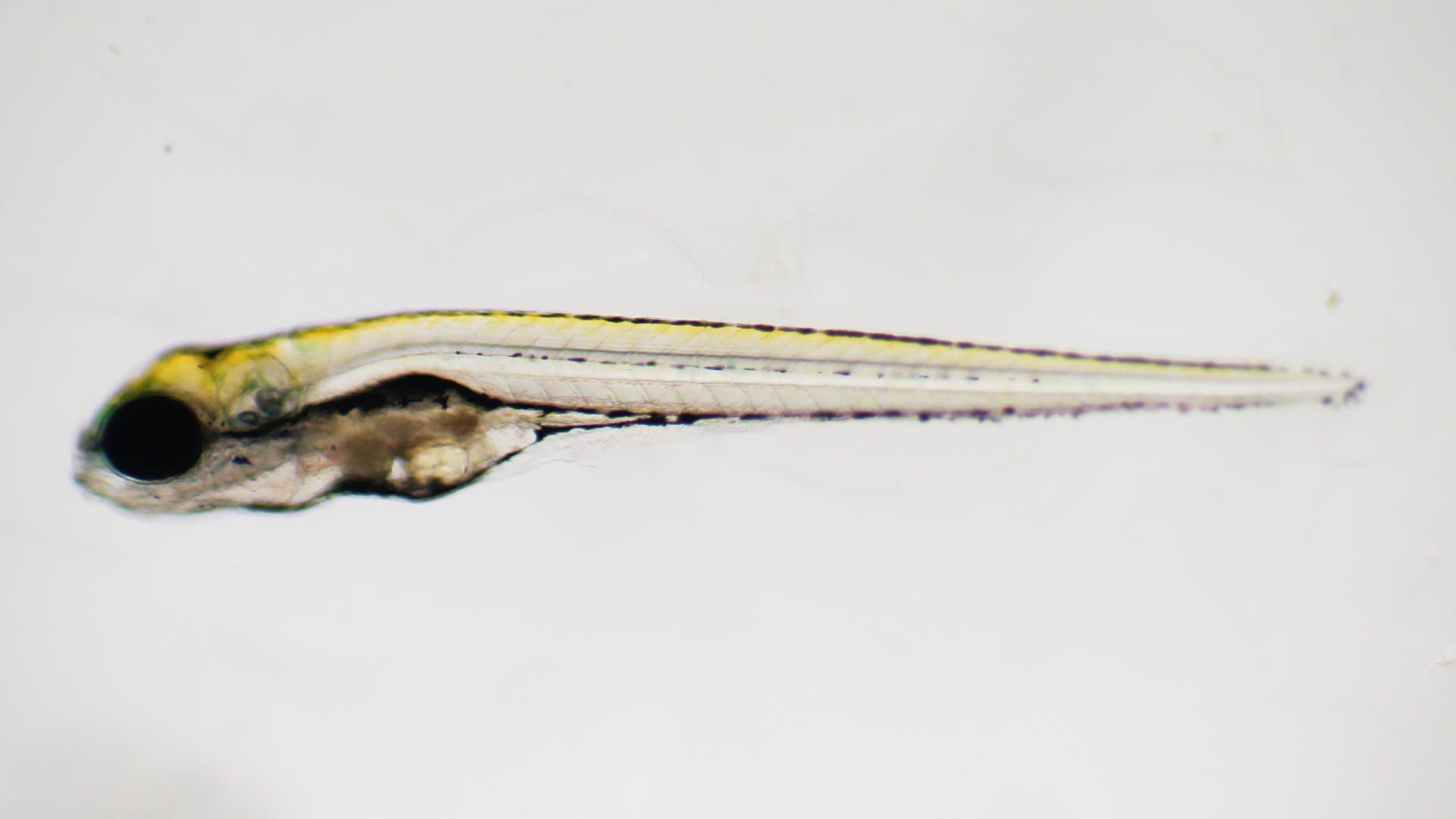

Supplement: Supplementary file 5 — Source data Fig. 4.2 [file 44321_2025_355_MOESM5_ESM.zip › non-path_1_8dpf.tif]

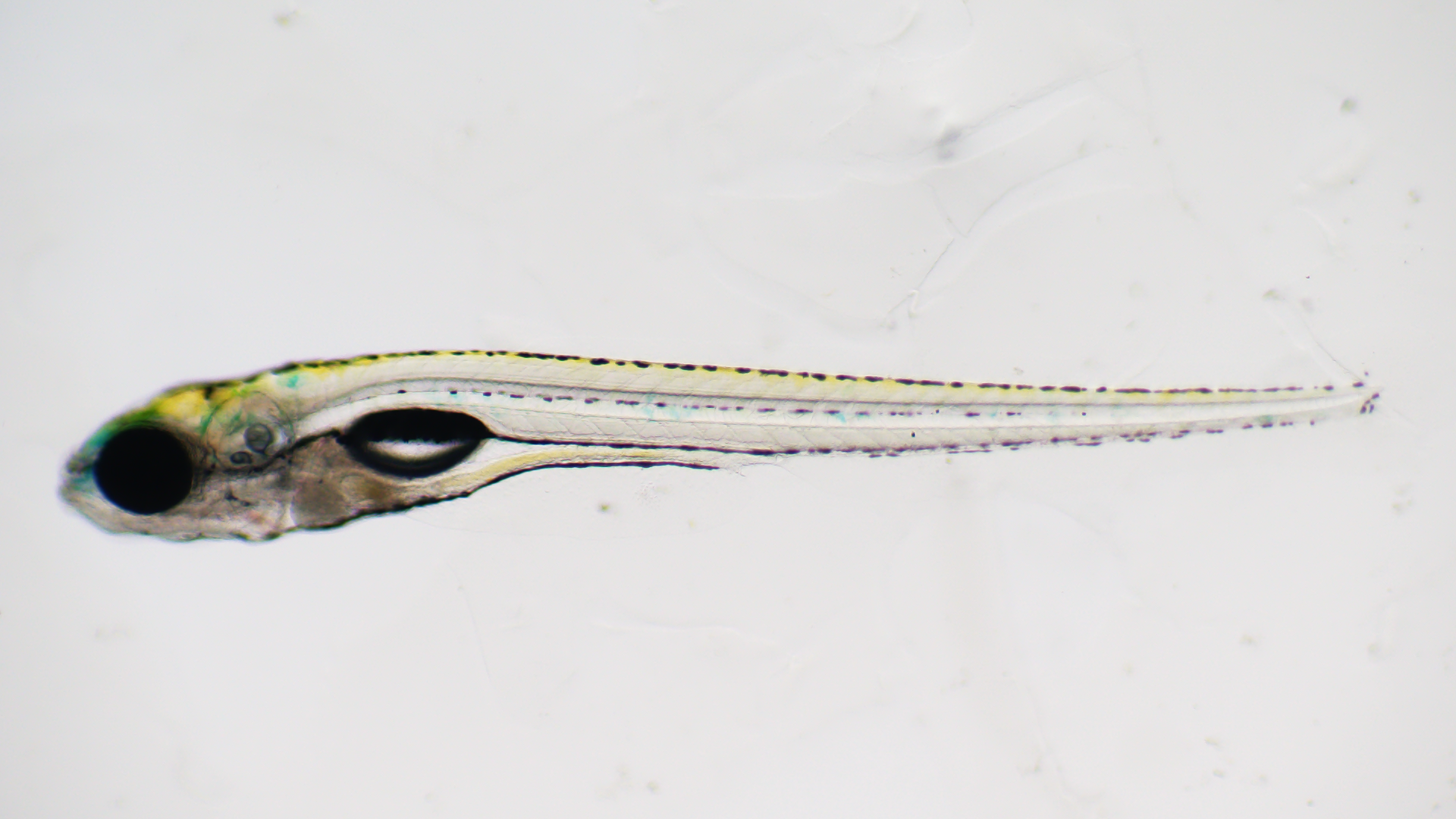

Supplement: Supplementary file 5 — Source data Fig. 4.2 [file 44321_2025_355_MOESM5_ESM.zip › non-path_1_9dpf.tif]

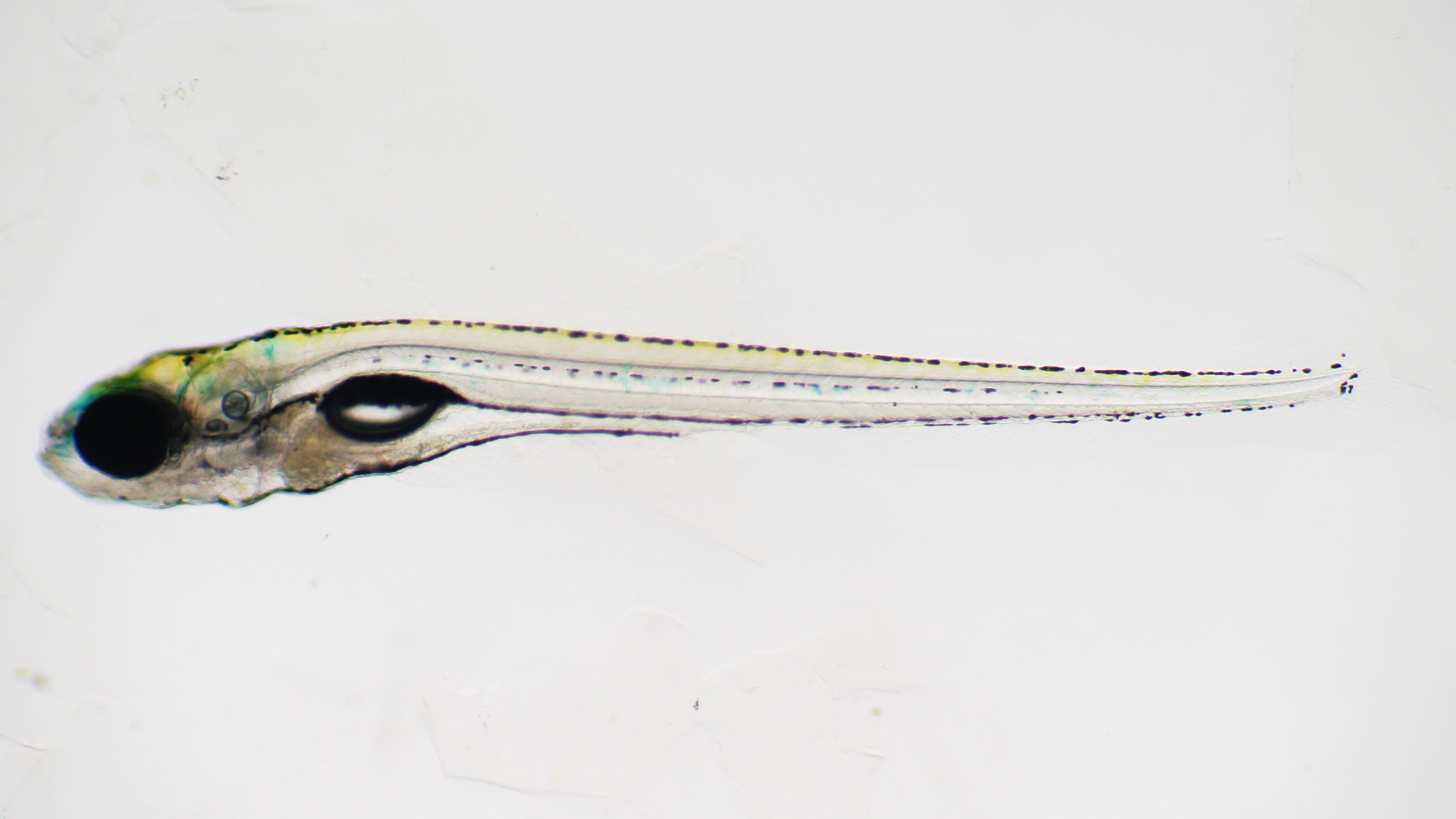

Supplement: Supplementary file 5 — Source data Fig. 4.2 [file 44321_2025_355_MOESM5_ESM.zip › non-path_1_10dpf.tif]

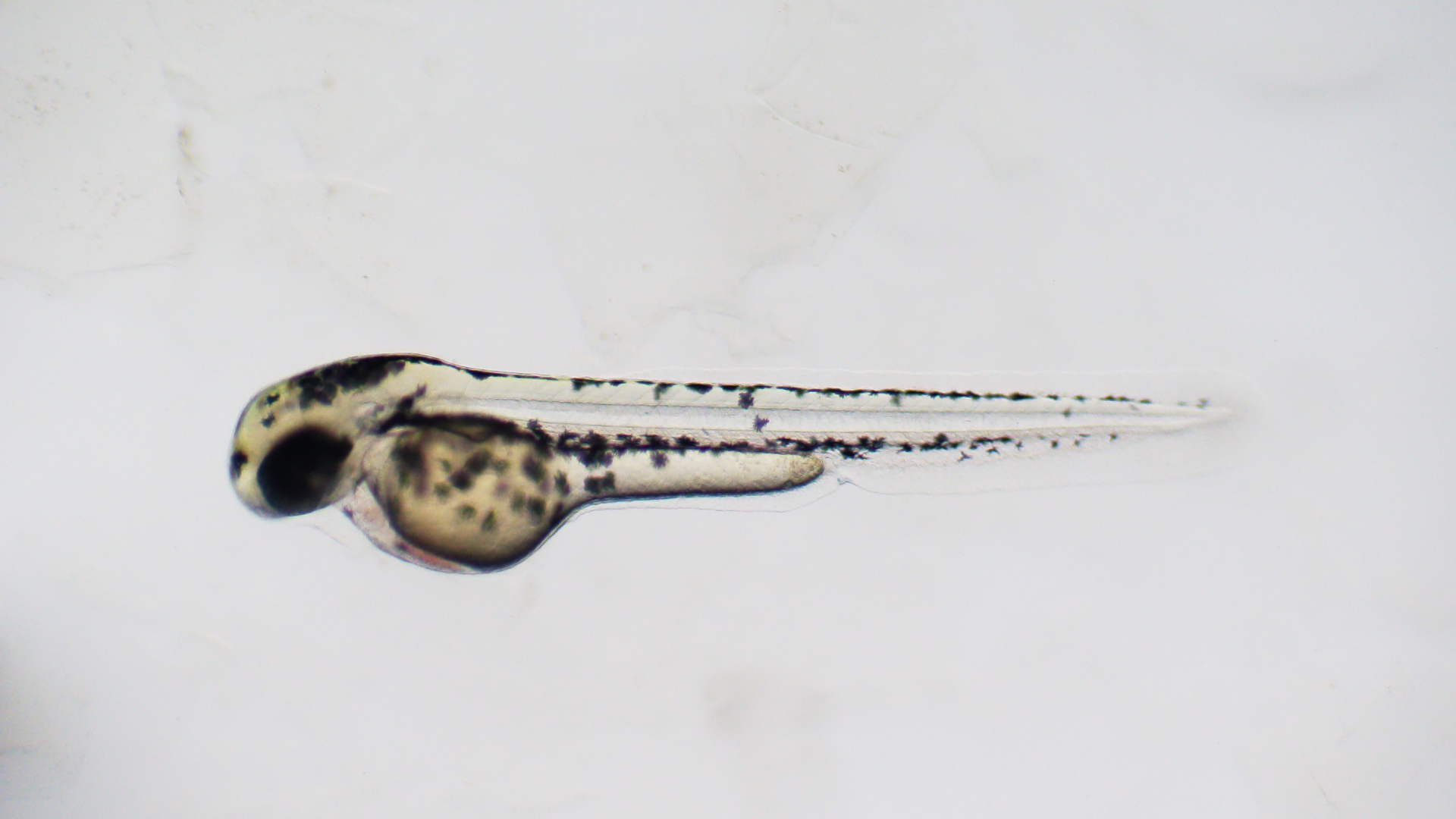

Supplement: Supplementary file 5 — Source data Fig. 4.2 [file 44321_2025_355_MOESM5_ESM.zip › non-path_2_2dpf.tif]

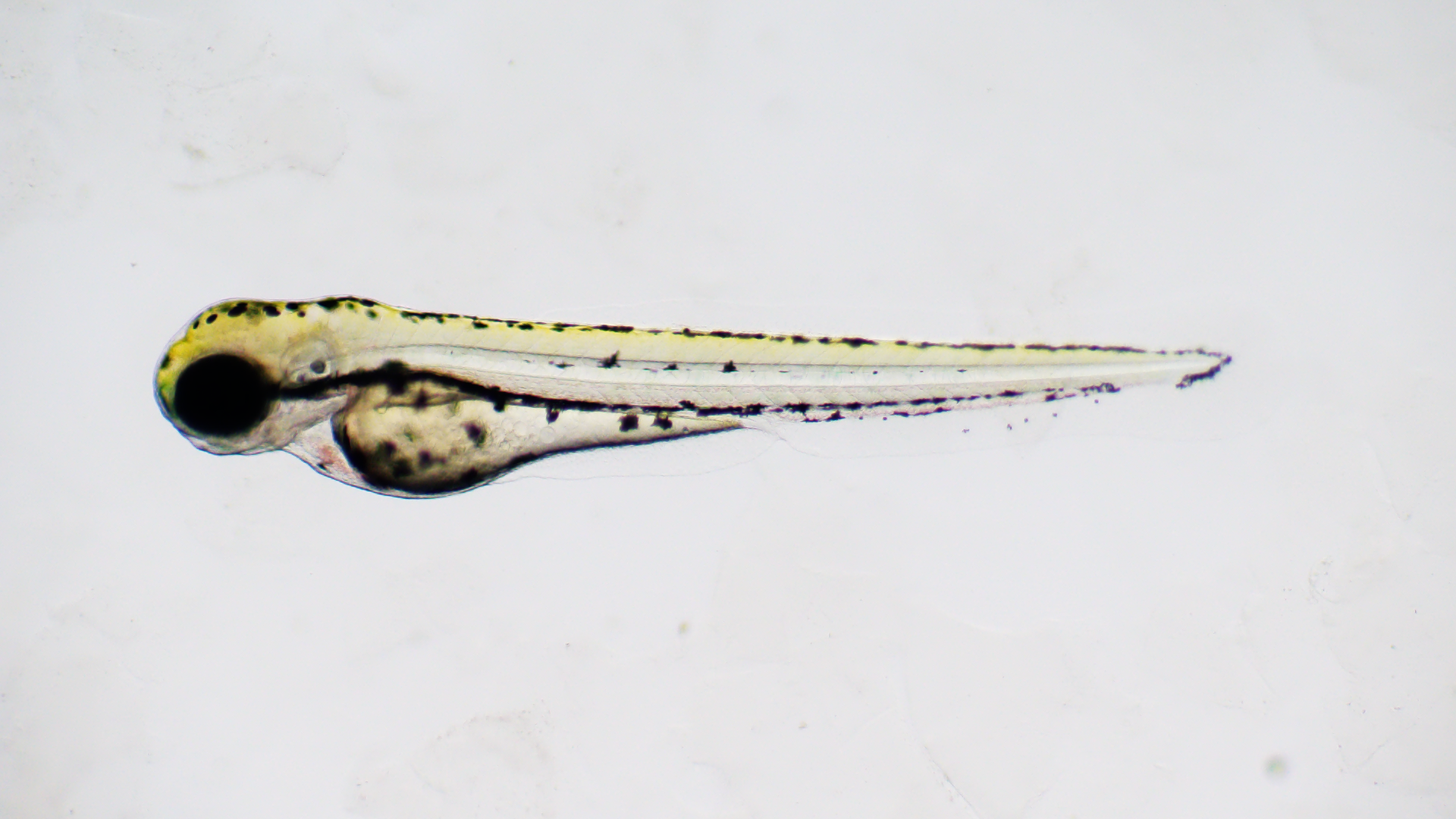

Supplement: Supplementary file 5 — Source data Fig. 4.2 [file 44321_2025_355_MOESM5_ESM.zip › non-path_2_3dpf.tif]

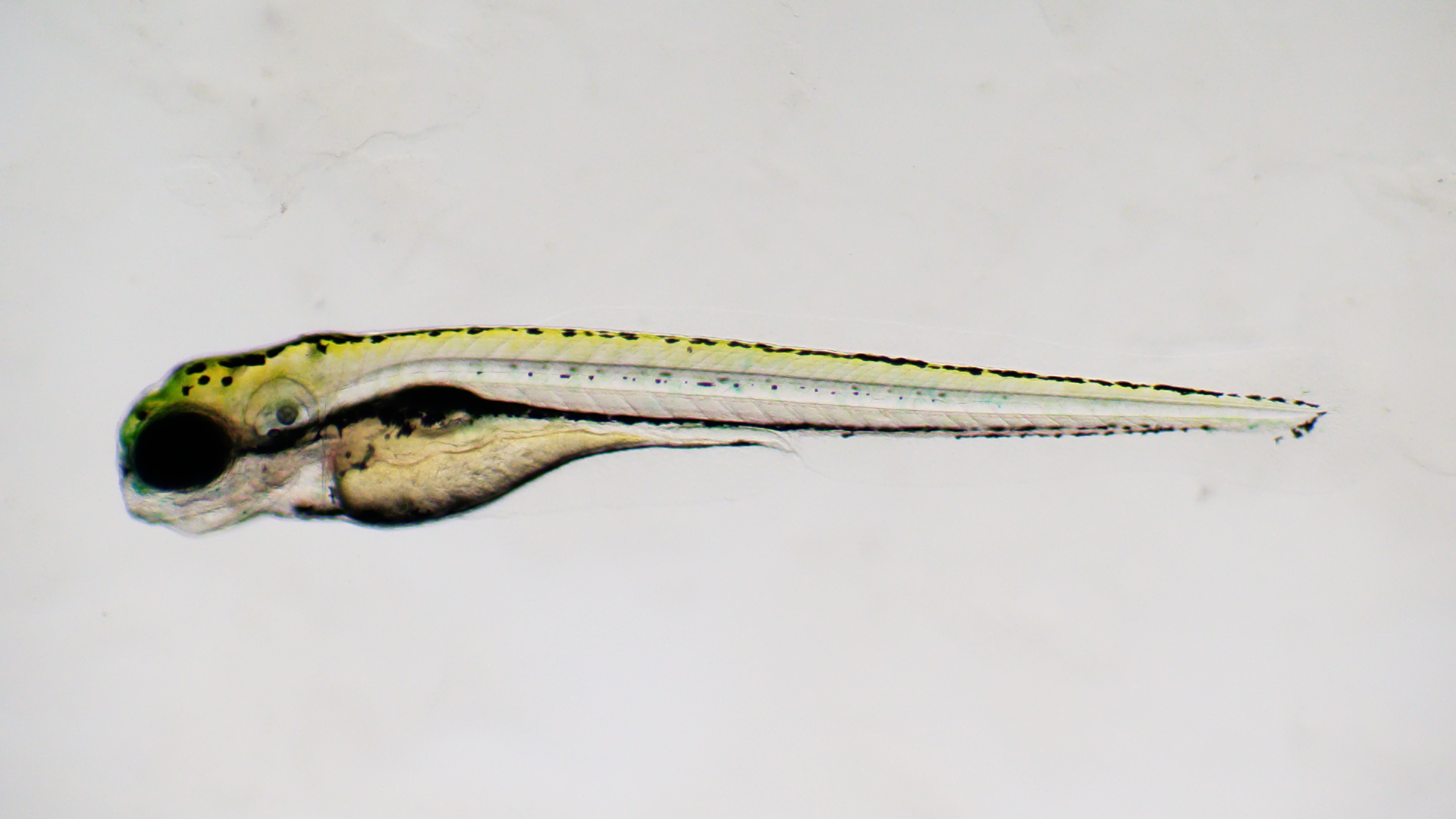

Supplement: Supplementary file 5 — Source data Fig. 4.2 [file 44321_2025_355_MOESM5_ESM.zip › non-path_2_4dpf.tif]

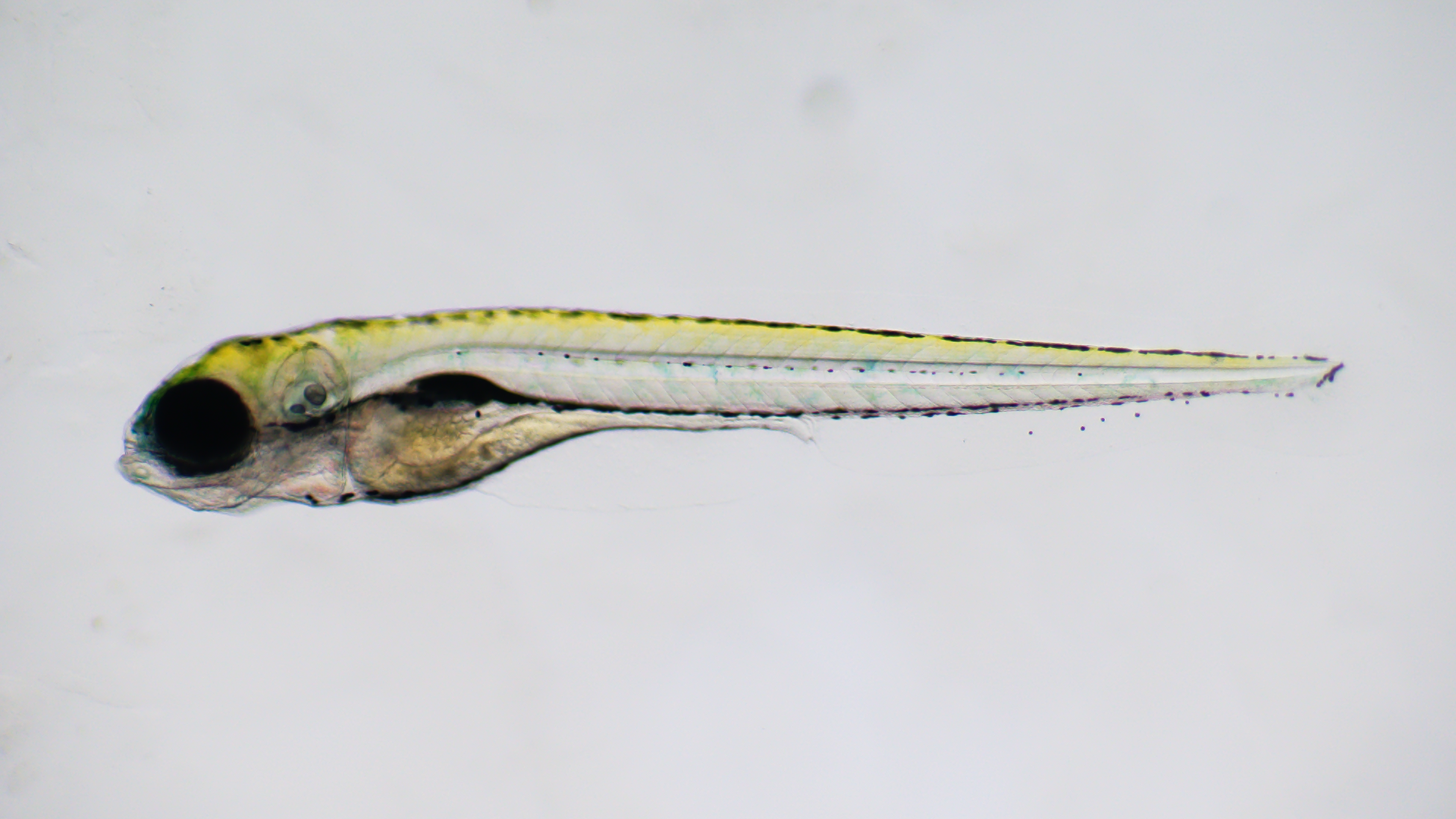

Supplement: Supplementary file 5 — Source data Fig. 4.2 [file 44321_2025_355_MOESM5_ESM.zip › non-path_2_5dpf.tif]

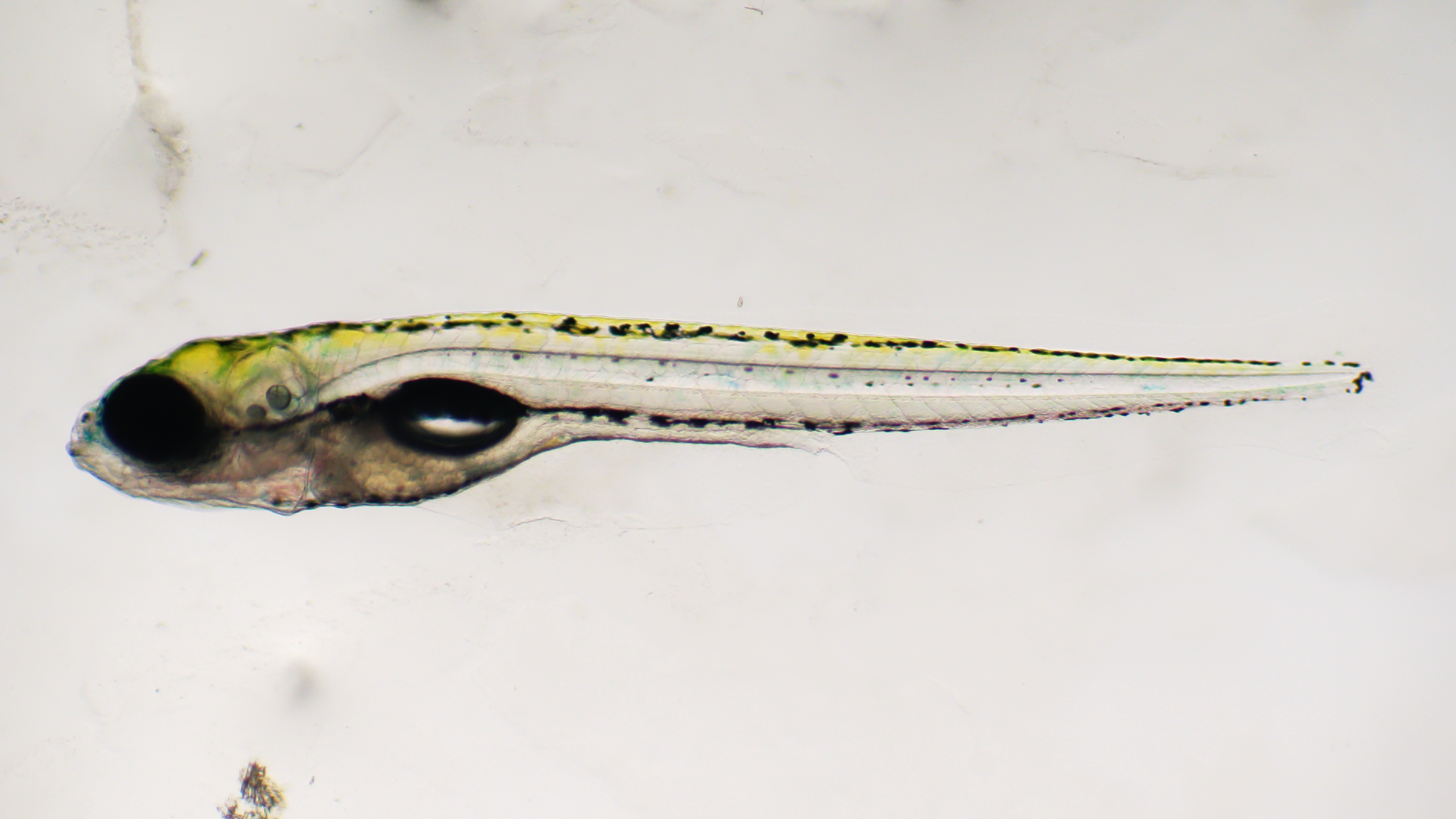

Supplement: Supplementary file 5 — Source data Fig. 4.2 [file 44321_2025_355_MOESM5_ESM.zip › non-path_2_6dpf.tif]

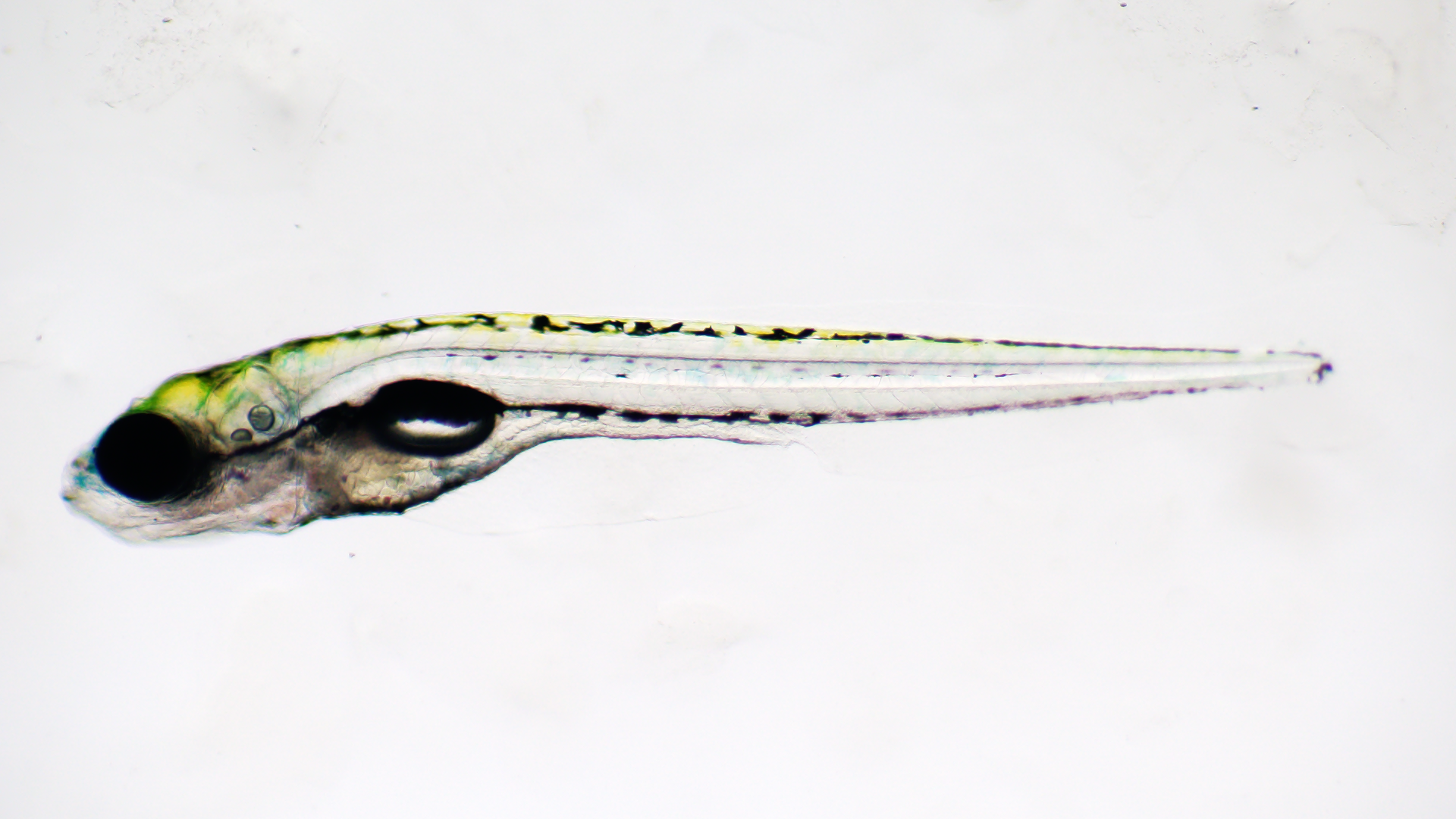

Supplement: Supplementary file 5 — Source data Fig. 4.2 [file 44321_2025_355_MOESM5_ESM.zip › non-path_2_7dpf.tif]

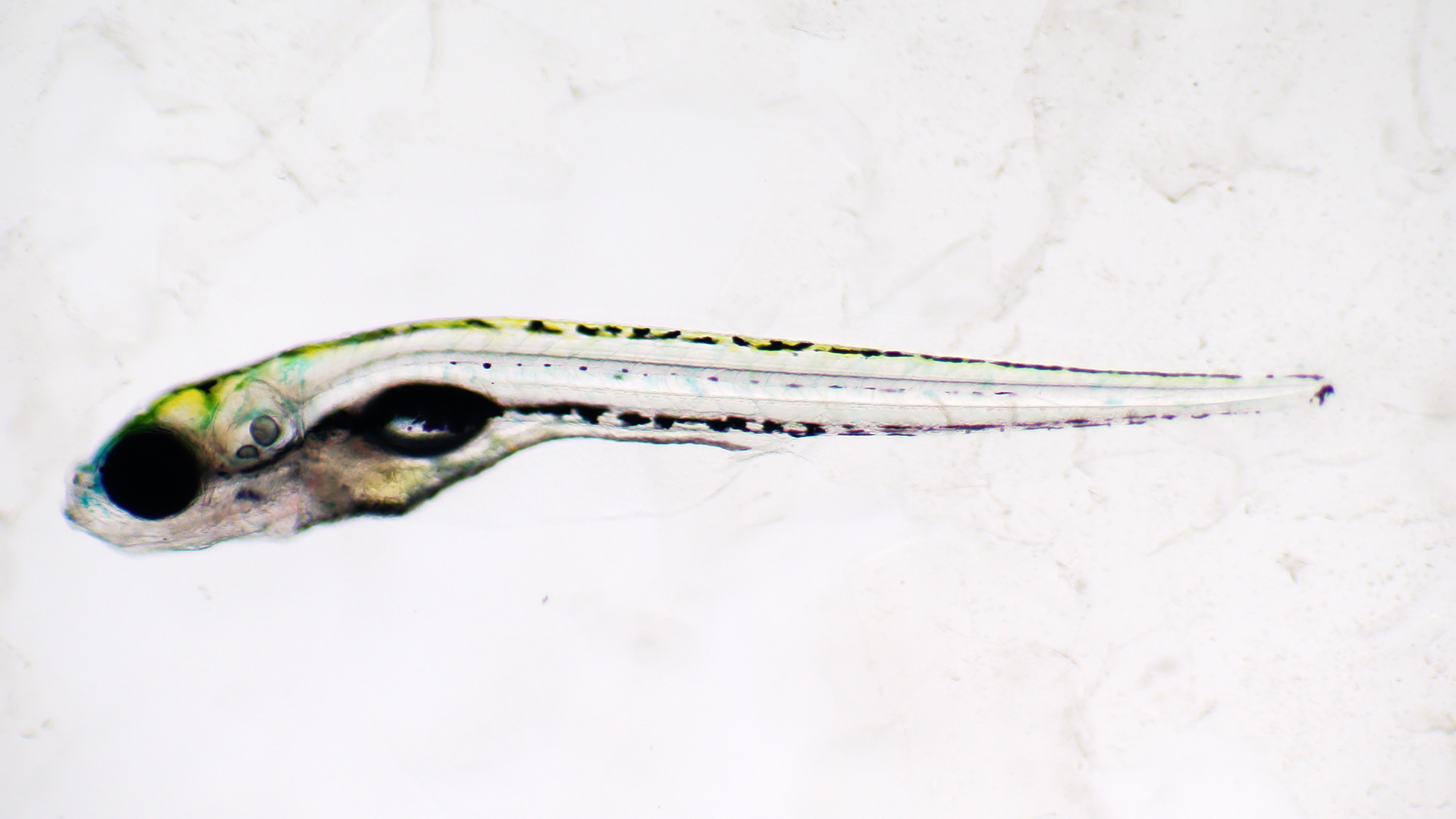

Supplement: Supplementary file 5 — Source data Fig. 4.2 [file 44321_2025_355_MOESM5_ESM.zip › non-path_2_8dpf.tif]

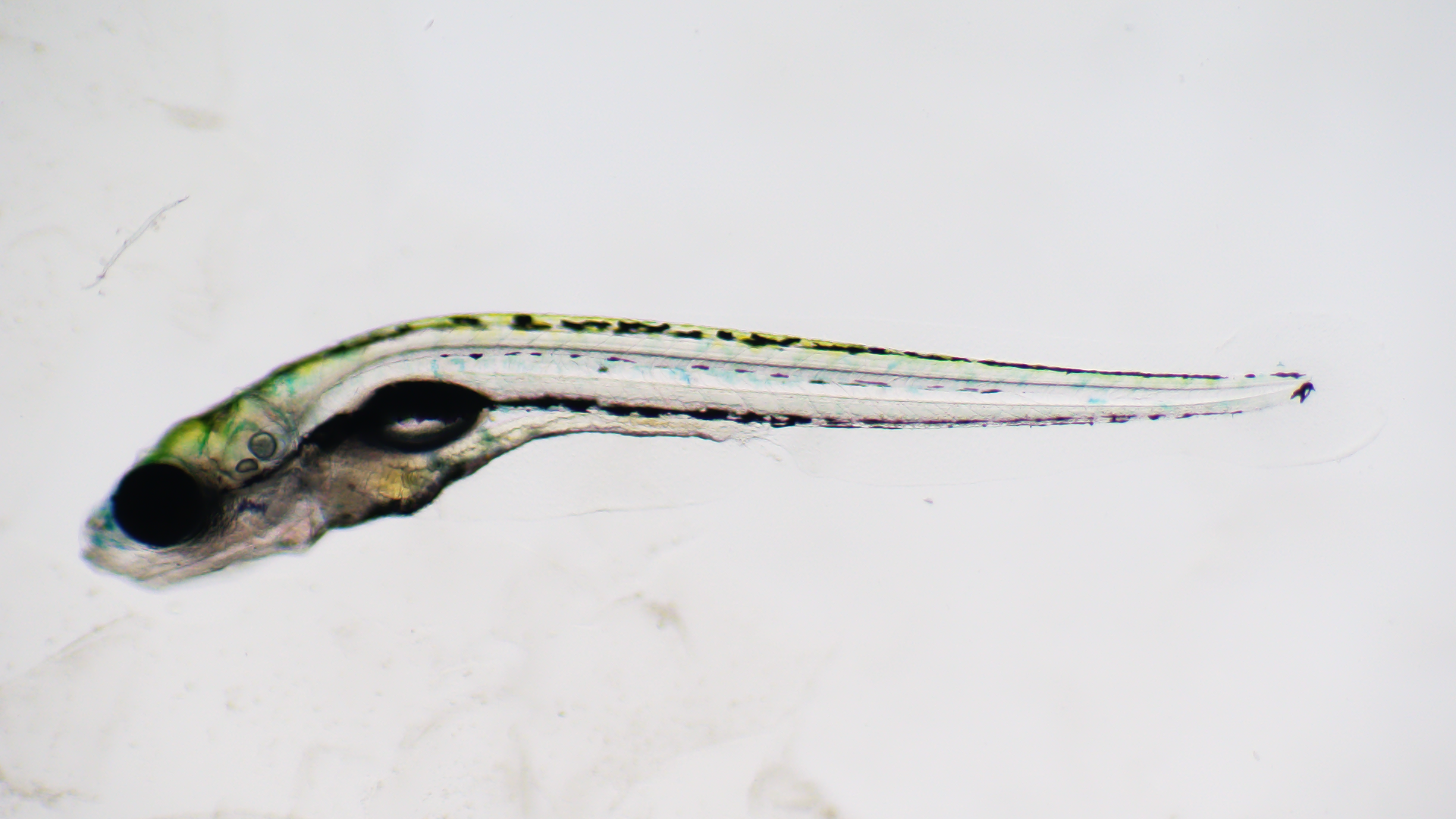

Supplement: Supplementary file 5 — Source data Fig. 4.2 [file 44321_2025_355_MOESM5_ESM.zip › non-path_2_9dpf.tif]

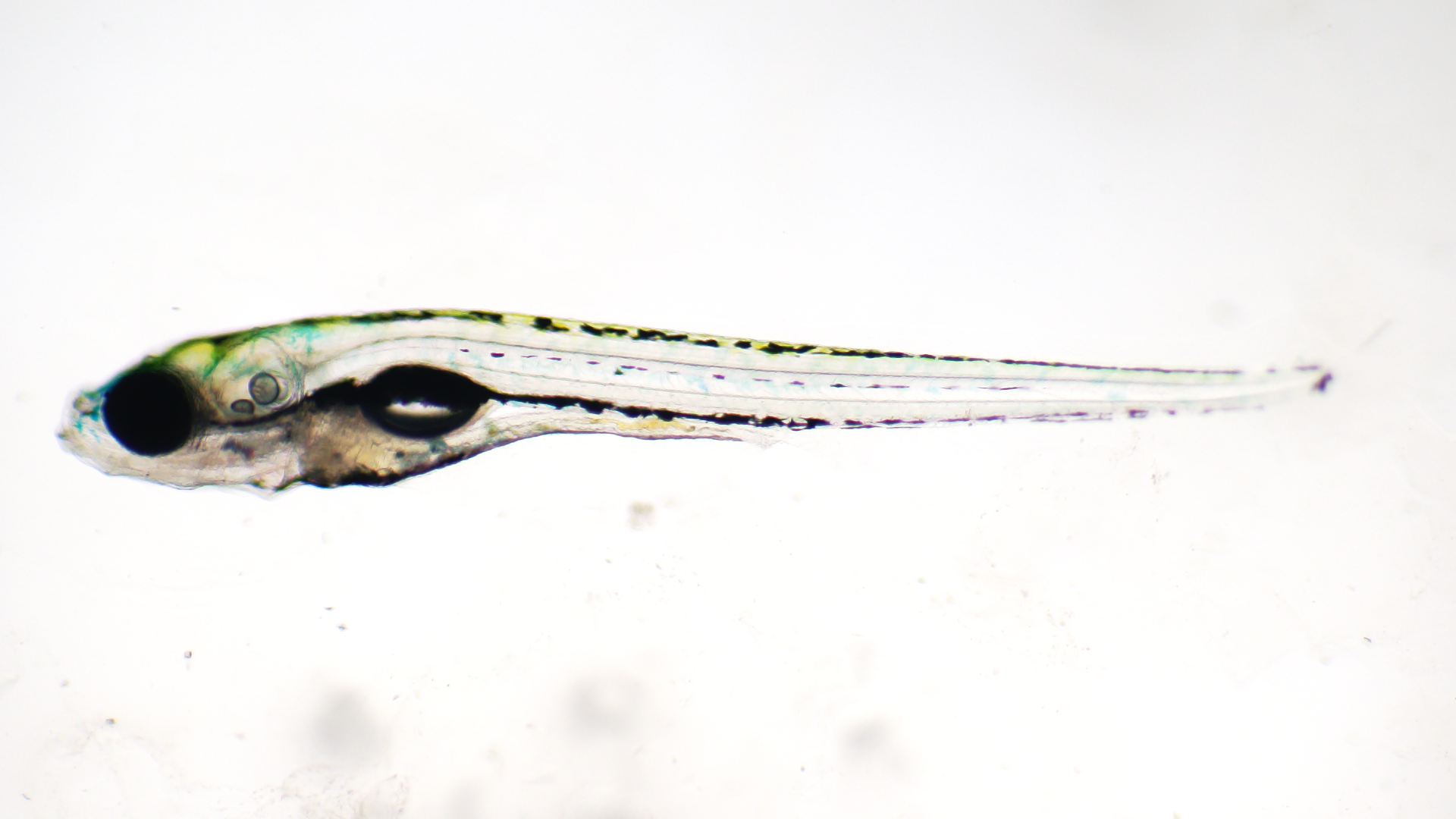

Supplement: Supplementary file 5 — Source data Fig. 4.2 [file 44321_2025_355_MOESM5_ESM.zip › non-path_2_10dpf.tif]

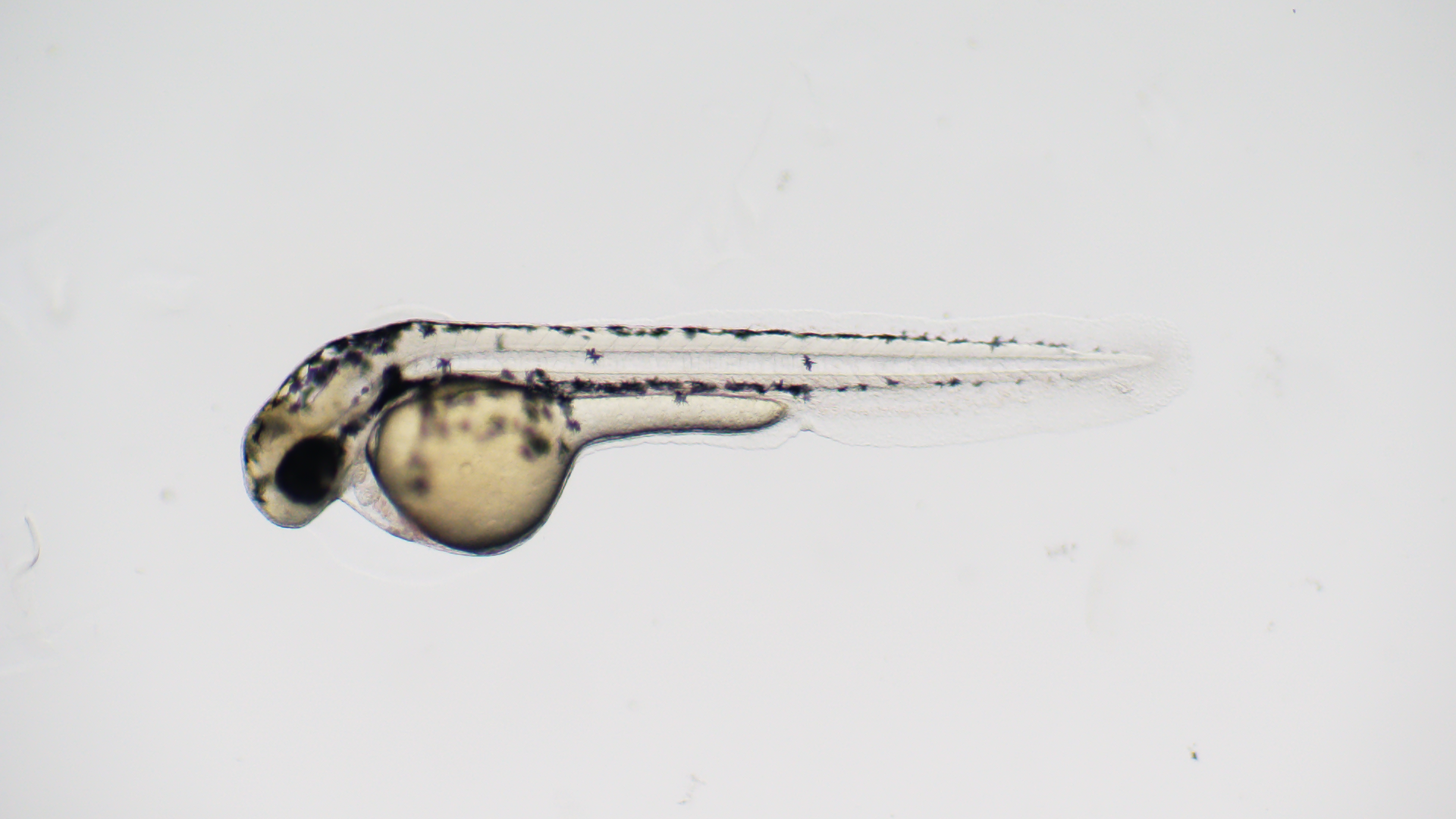

Supplement: Supplementary file 5 — Source data Fig. 4.2 [file 44321_2025_355_MOESM5_ESM.zip › P245L_2dpf.tif]

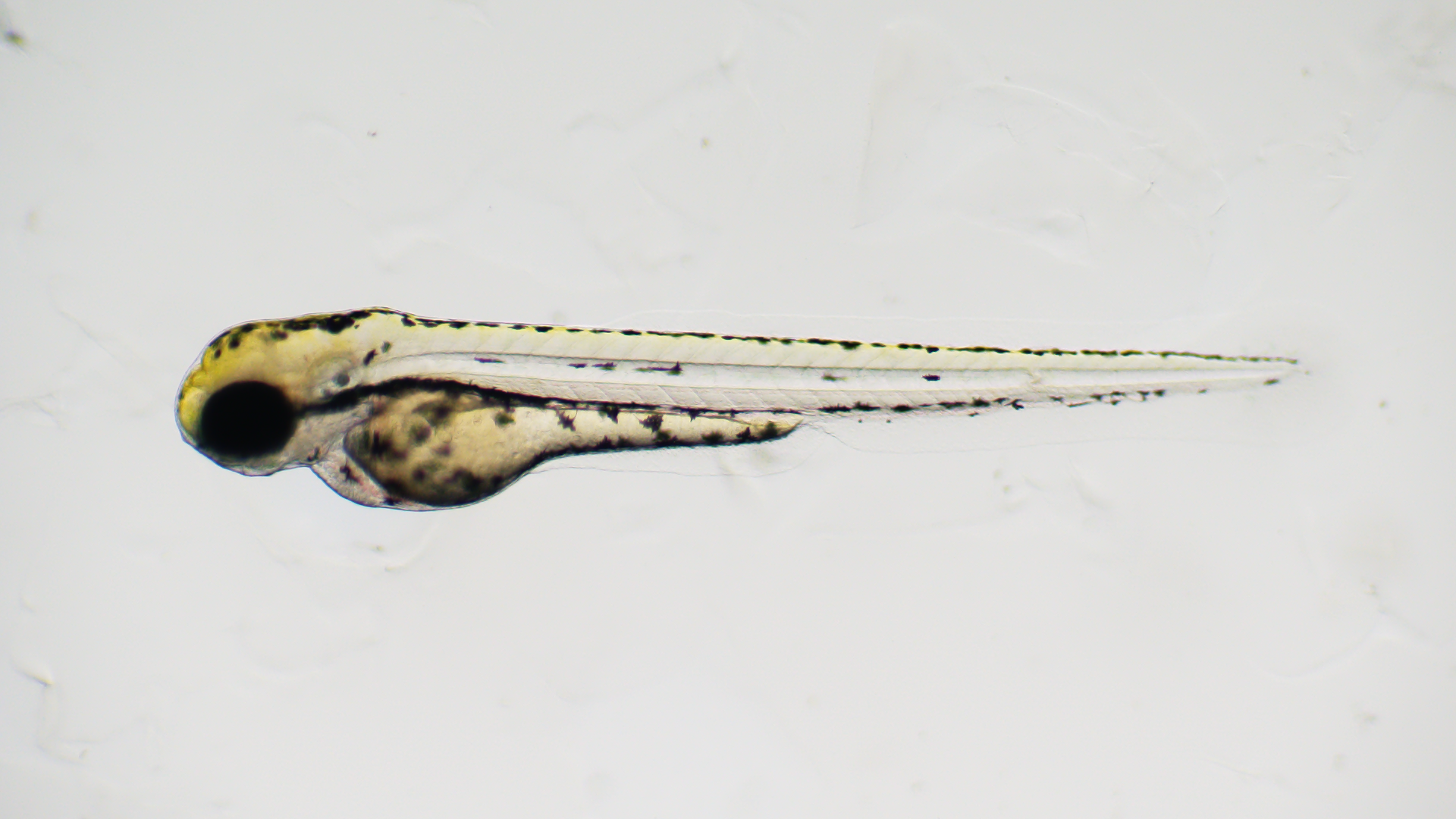

Supplement: Supplementary file 5 — Source data Fig. 4.2 [file 44321_2025_355_MOESM5_ESM.zip › P245L_3dpf.tif]

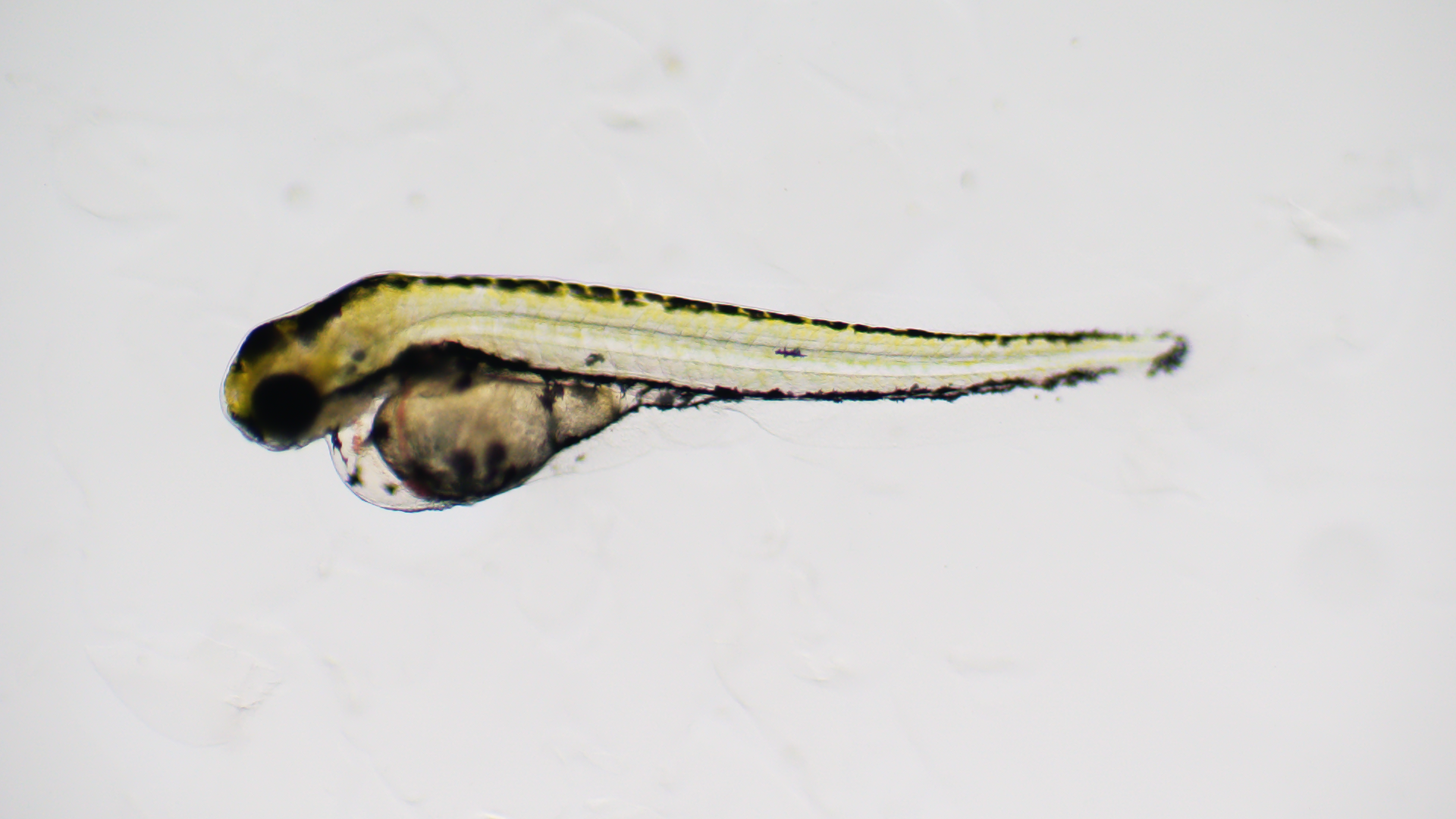

Supplement: Supplementary file 5 — Source data Fig. 4.2 [file 44321_2025_355_MOESM5_ESM.zip › P245L_4dpf.tif]

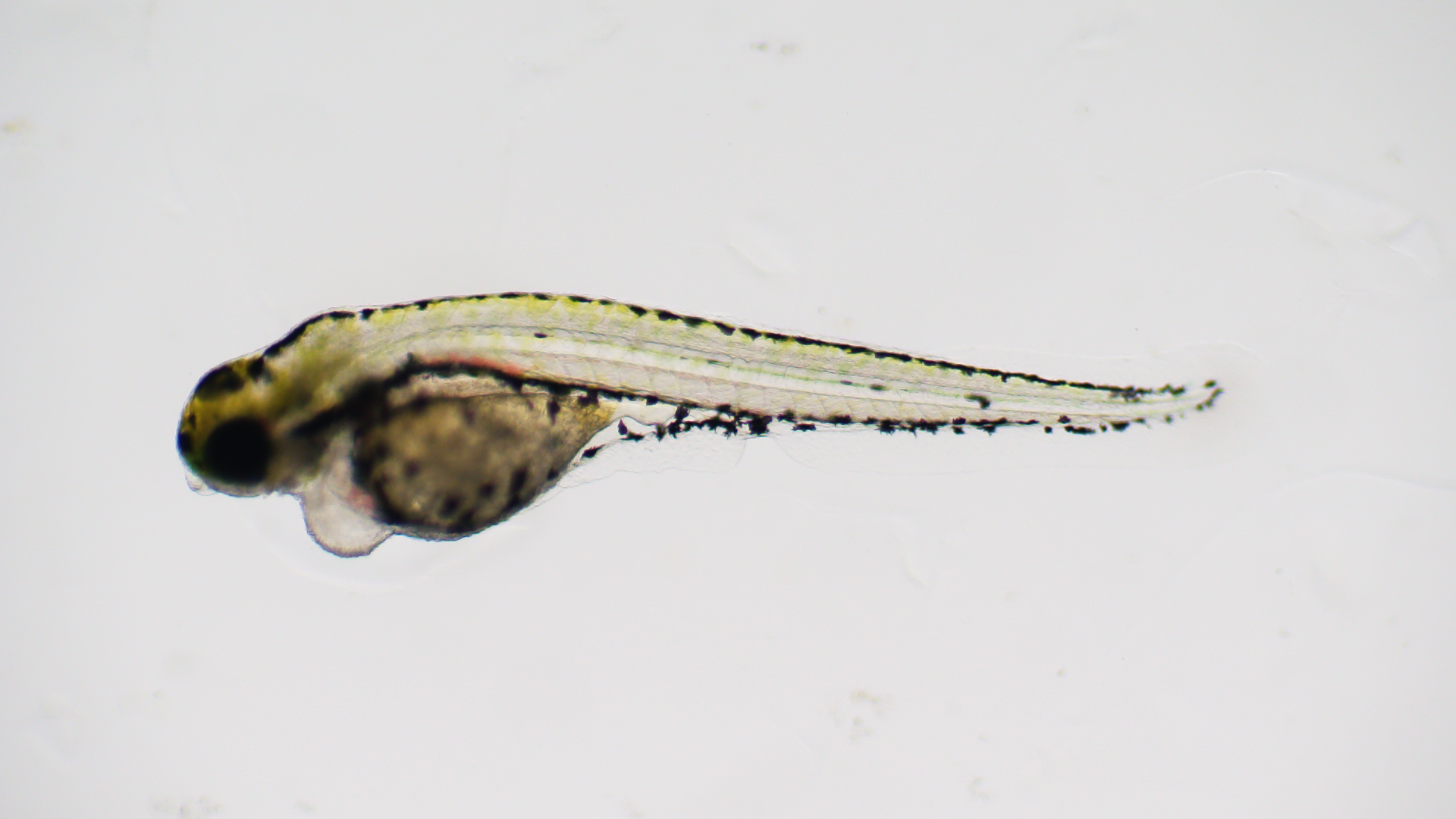

Supplement: Supplementary file 5 — Source data Fig. 4.2 [file 44321_2025_355_MOESM5_ESM.zip › P245L_5dpf.tif]

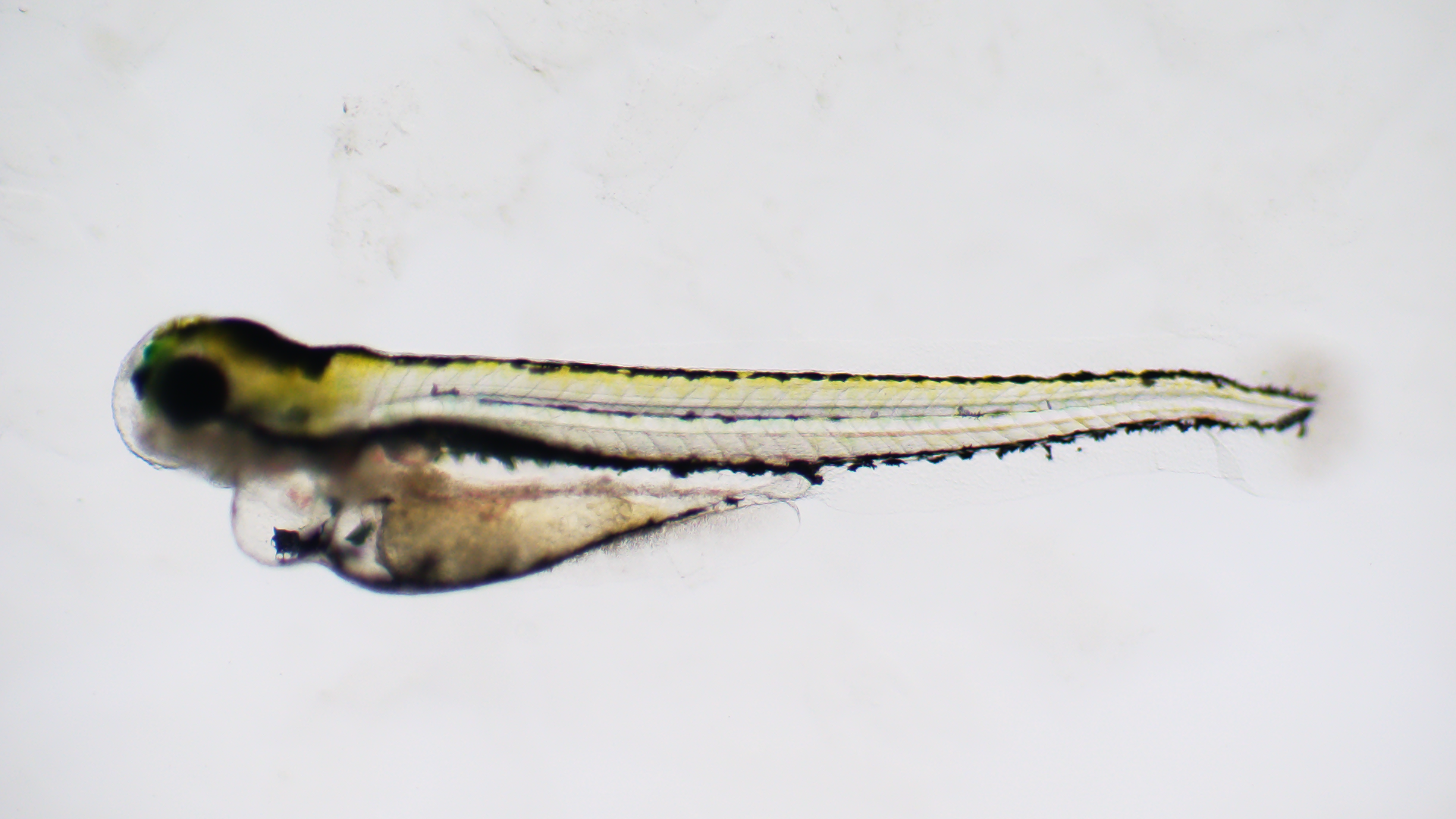

Supplement: Supplementary file 5 — Source data Fig. 4.2 [file 44321_2025_355_MOESM5_ESM.zip › P245L_6dpf.tif]

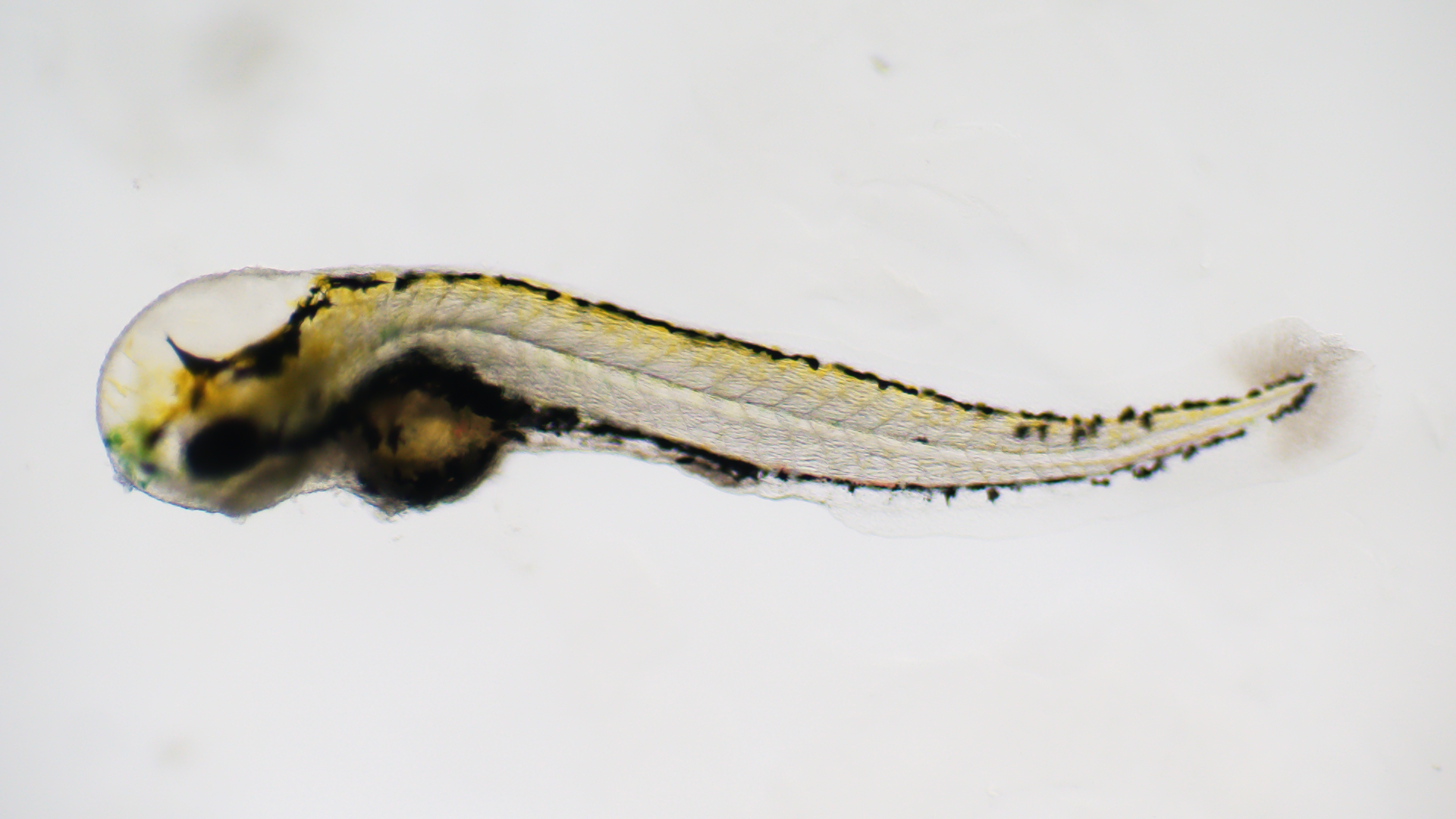

Supplement: Supplementary file 5 — Source data Fig. 4.2 [file 44321_2025_355_MOESM5_ESM.zip › P245L_7dpf.tif]

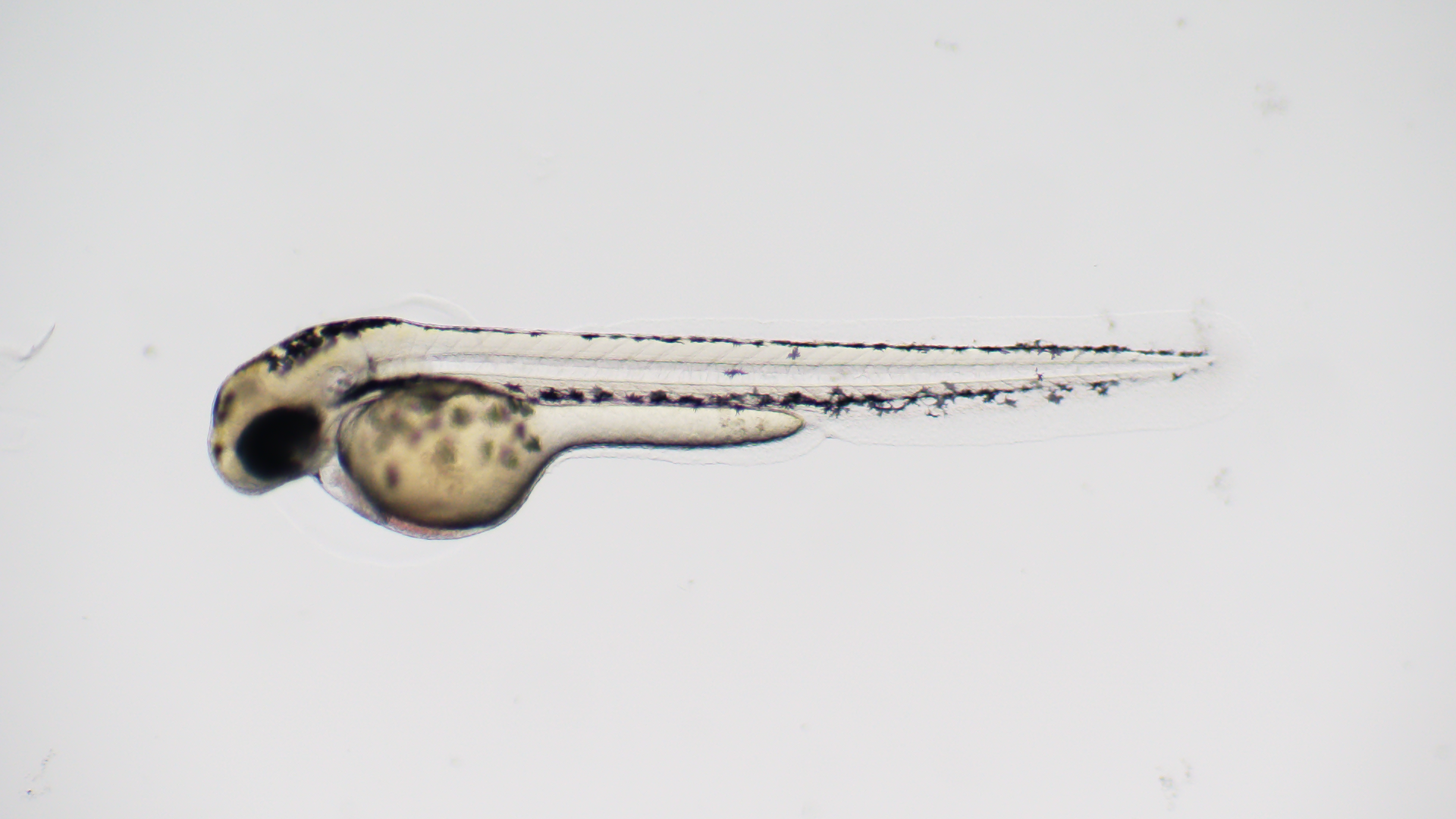

Supplement: Supplementary file 5 — Source data Fig. 4.2 [file 44321_2025_355_MOESM5_ESM.zip › path_1_2dpf.tif]

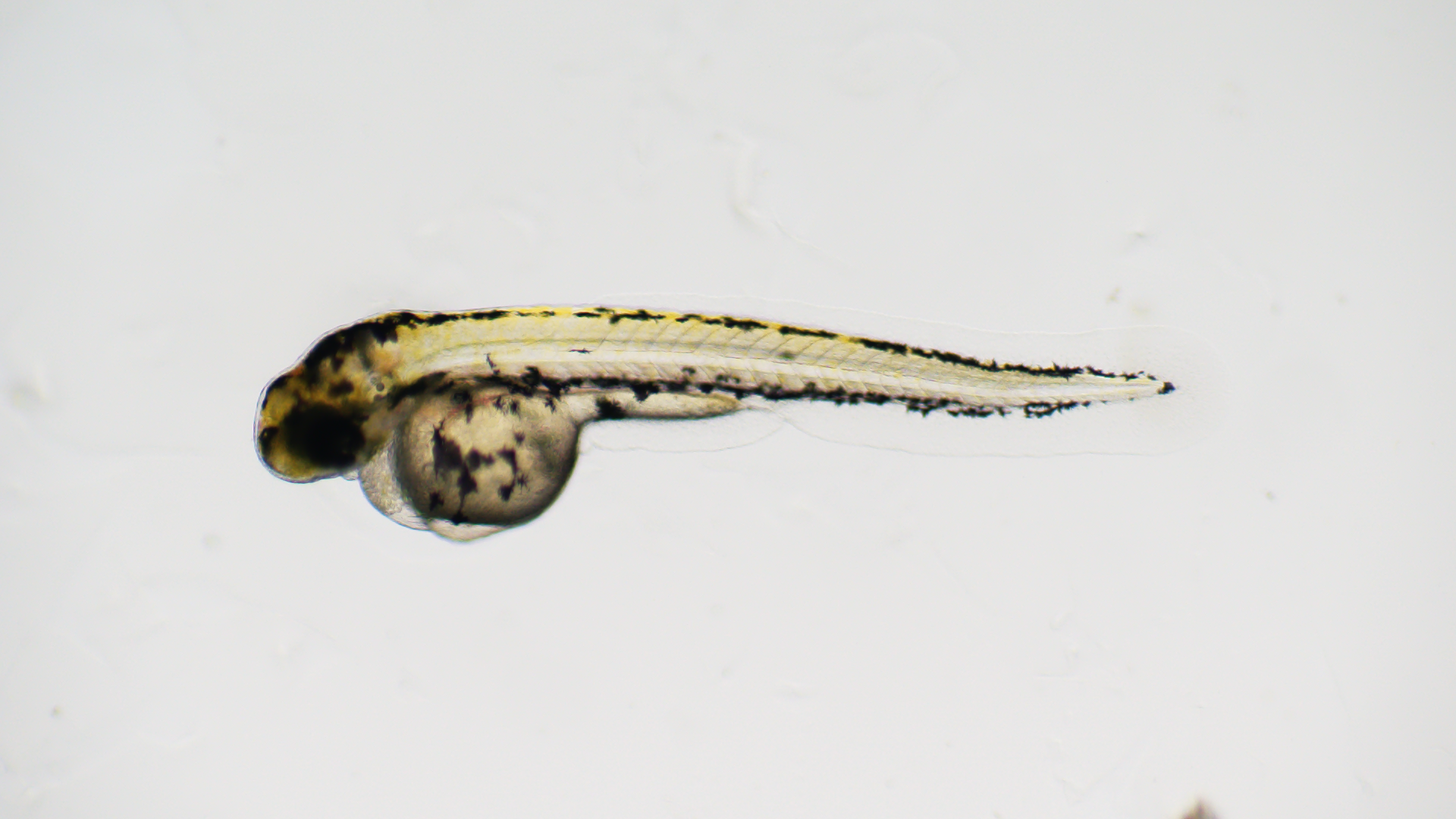

Supplement: Supplementary file 5 — Source data Fig. 4.2 [file 44321_2025_355_MOESM5_ESM.zip › path_1_3dpf.tif]

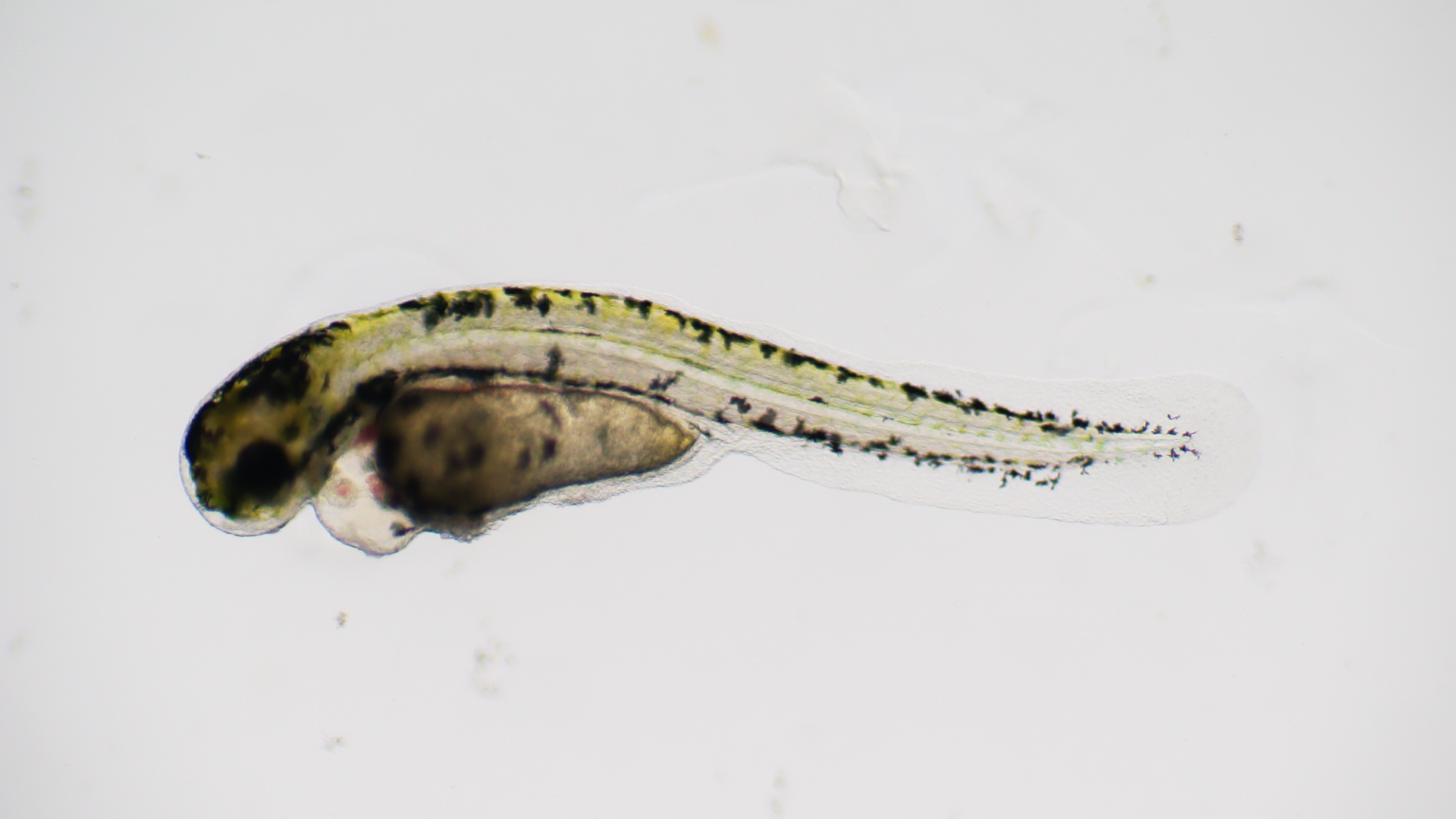

Supplement: Supplementary file 5 — Source data Fig. 4.2 [file 44321_2025_355_MOESM5_ESM.zip › path_1_4dpf.tif]

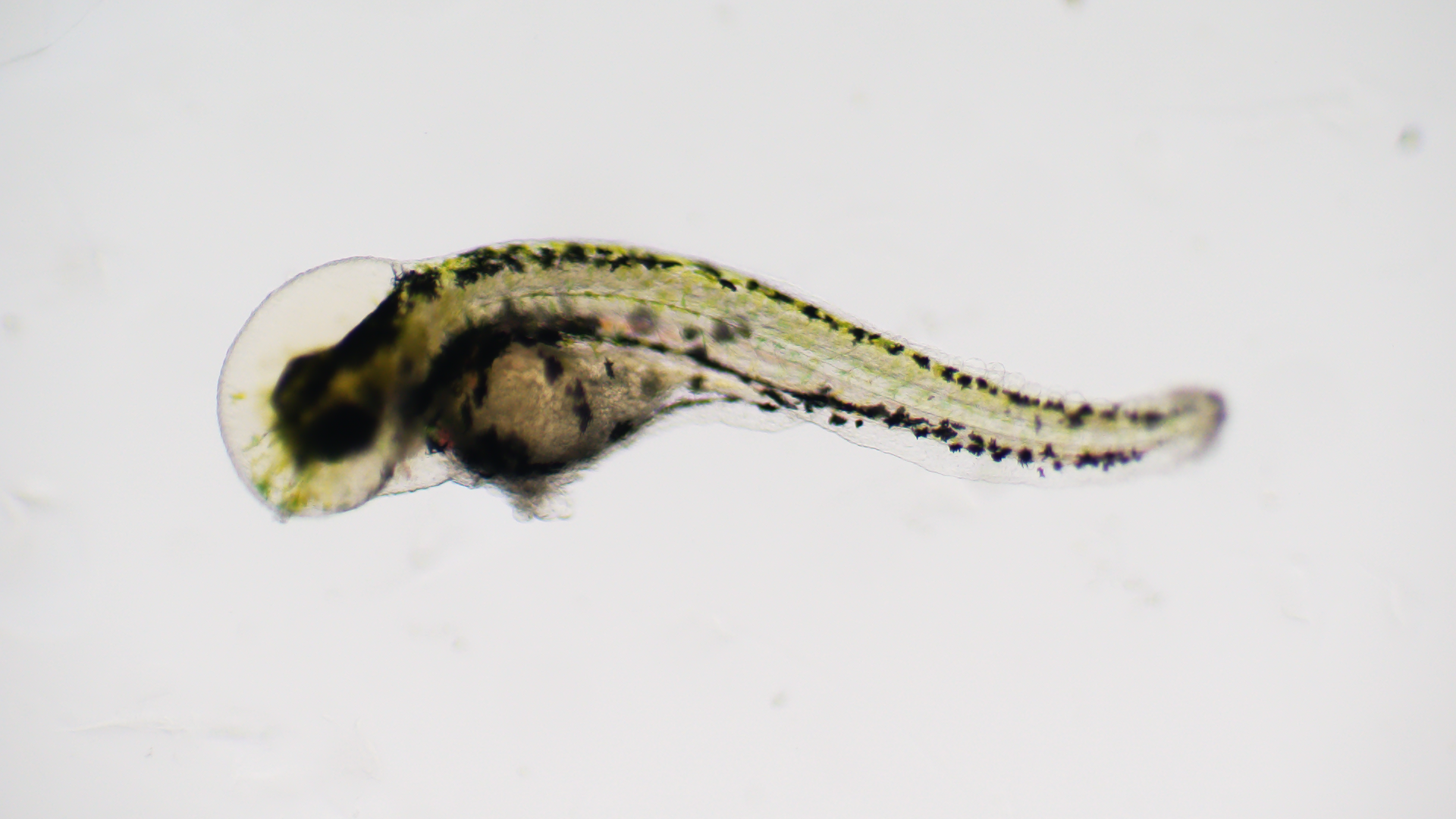

Supplement: Supplementary file 5 — Source data Fig. 4.2 [file 44321_2025_355_MOESM5_ESM.zip › path_1_5dpf.tif]

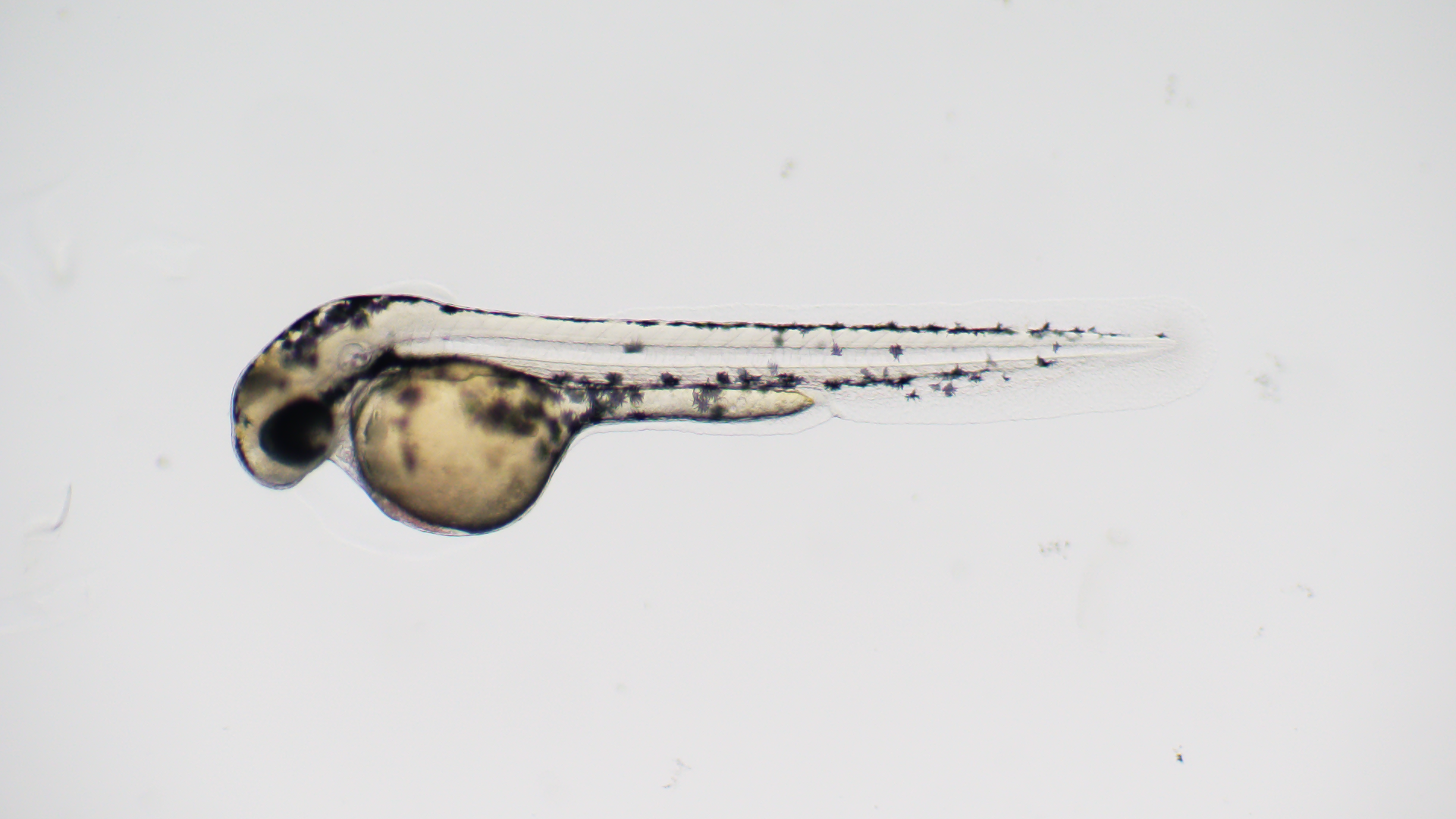

Supplement: Supplementary file 5 — Source data Fig. 4.2 [file 44321_2025_355_MOESM5_ESM.zip › path_2_2dpf.tif]

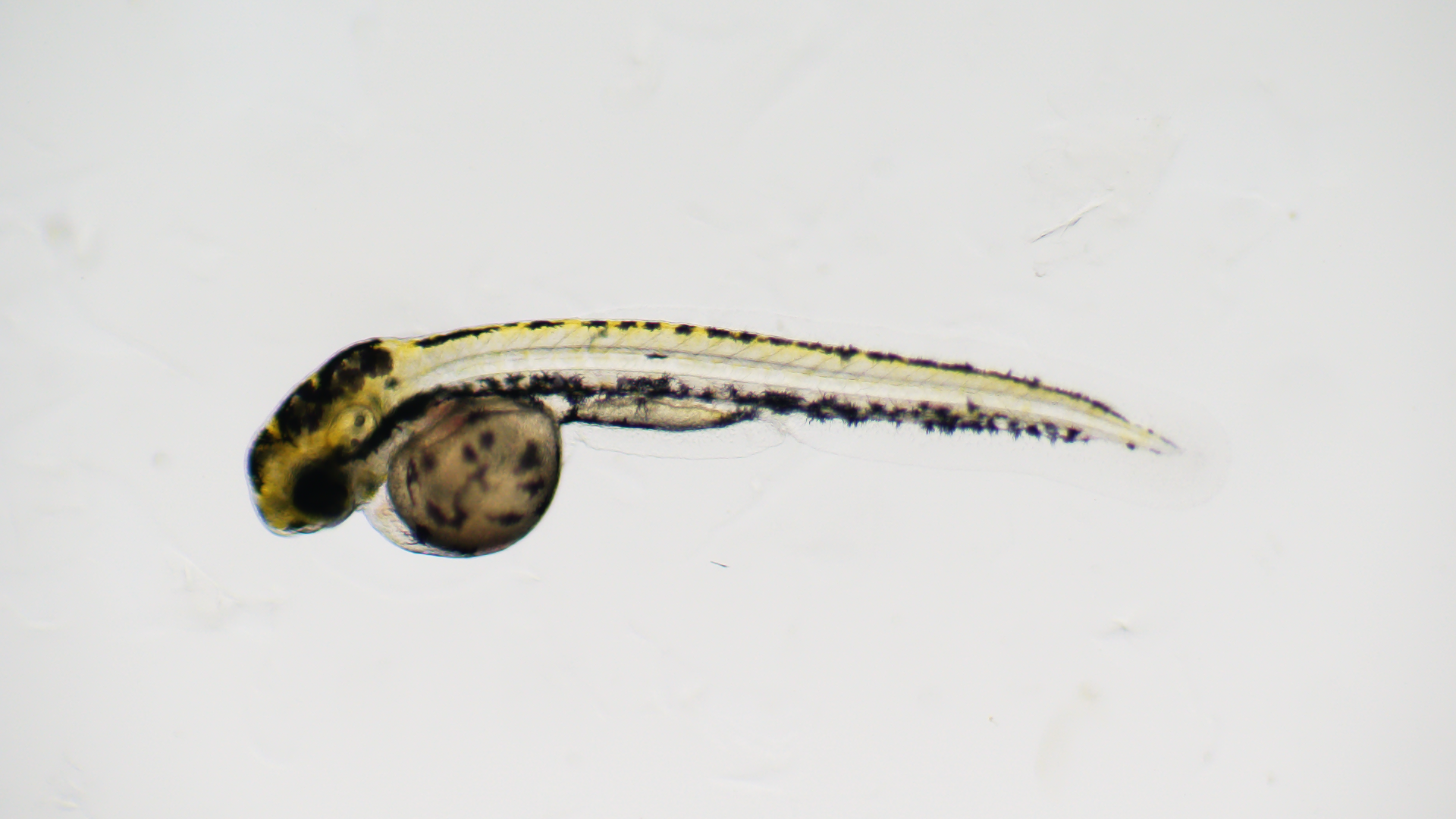

Supplement: Supplementary file 5 — Source data Fig. 4.2 [file 44321_2025_355_MOESM5_ESM.zip › path_2_3dpf.tif]

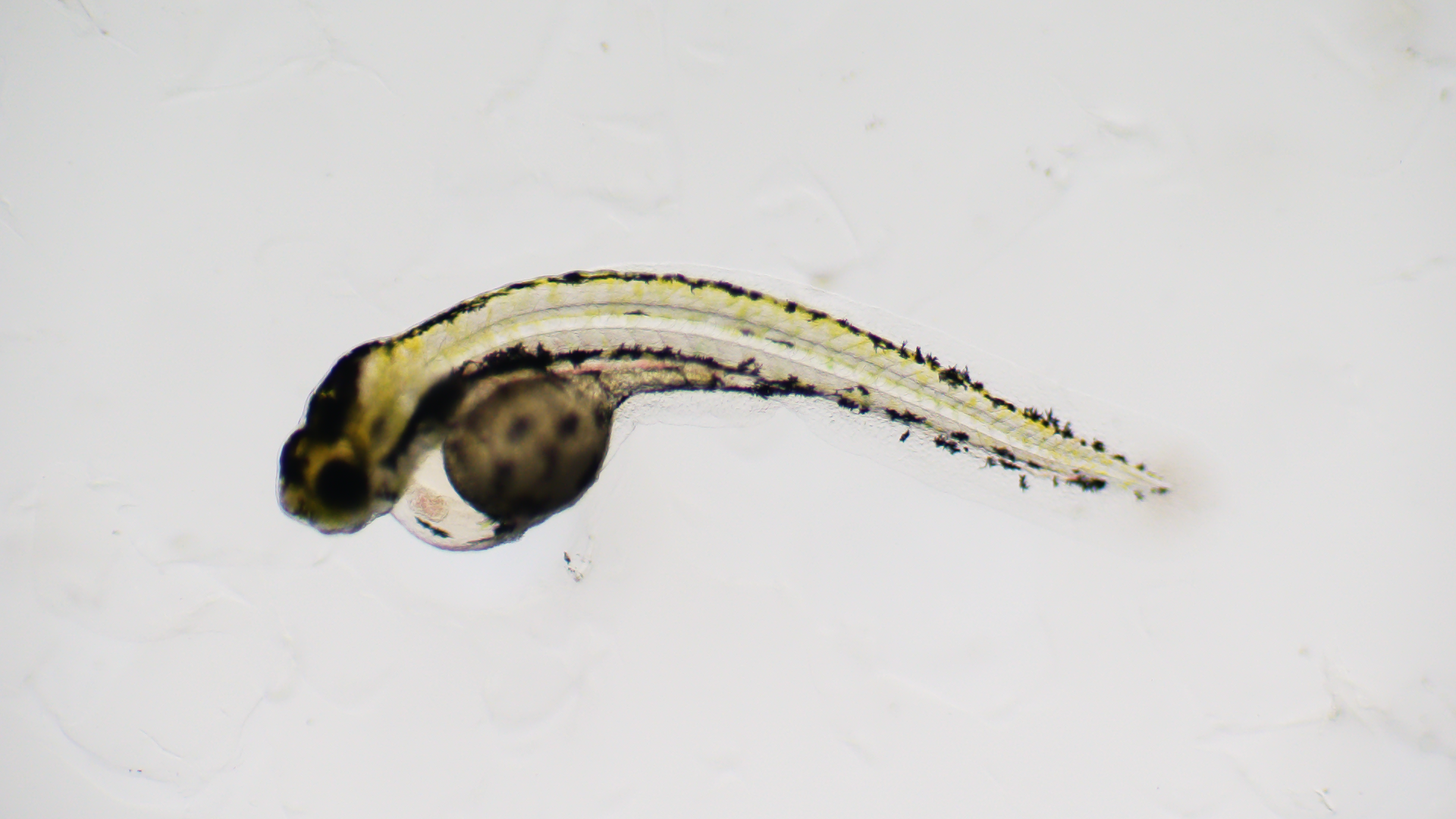

Supplement: Supplementary file 5 — Source data Fig. 4.2 [file 44321_2025_355_MOESM5_ESM.zip › path_2_4dpf.tif]

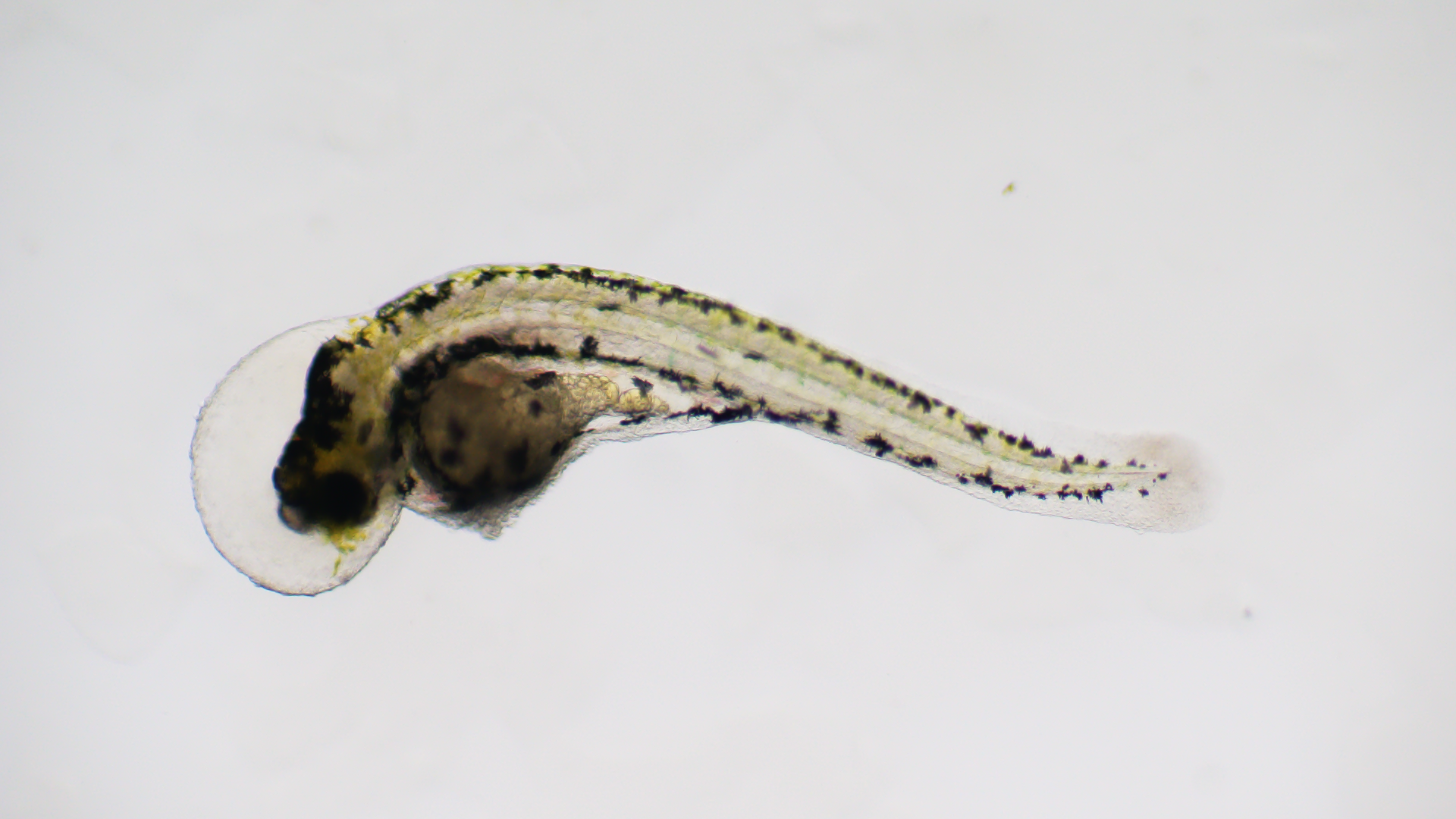

Supplement: Supplementary file 5 — Source data Fig. 4.2 [file 44321_2025_355_MOESM5_ESM.zip › path_2_5dpf.tif]

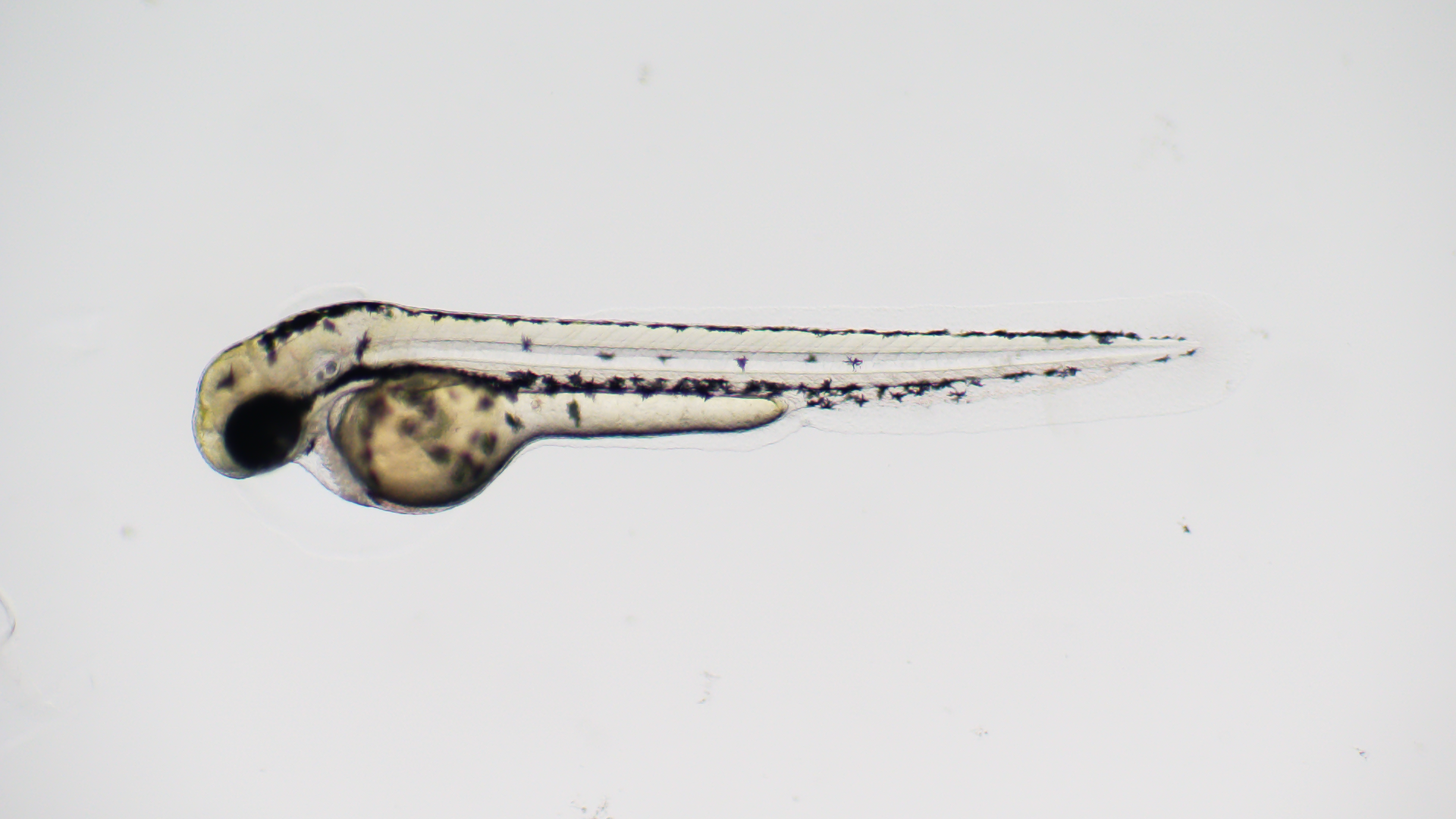

Supplement: Supplementary file 5 — Source data Fig. 4.2 [file 44321_2025_355_MOESM5_ESM.zip › S262I_2dpf.tif]

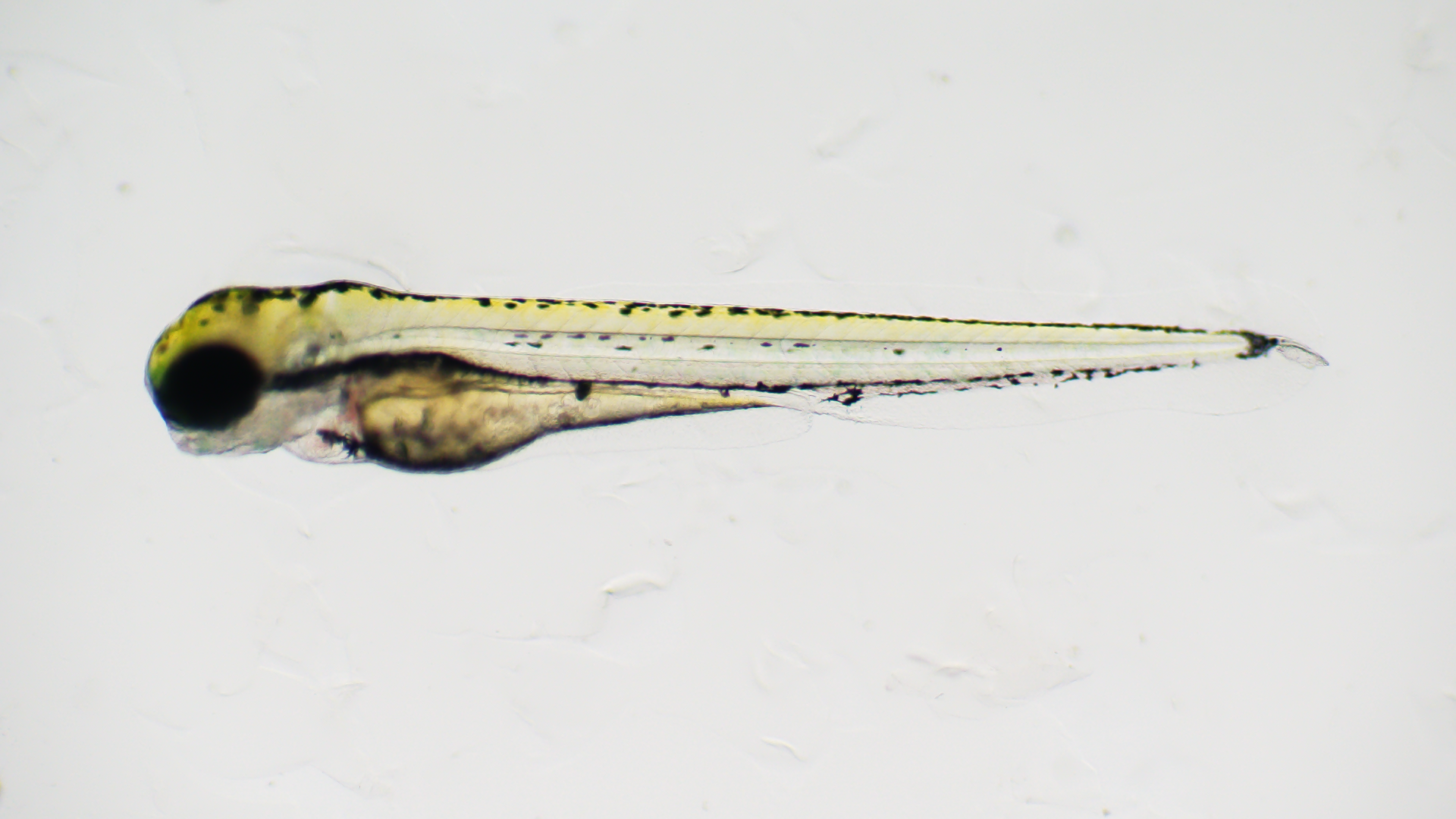

Supplement: Supplementary file 5 — Source data Fig. 4.2 [file 44321_2025_355_MOESM5_ESM.zip › S262I_3dpf.tif]

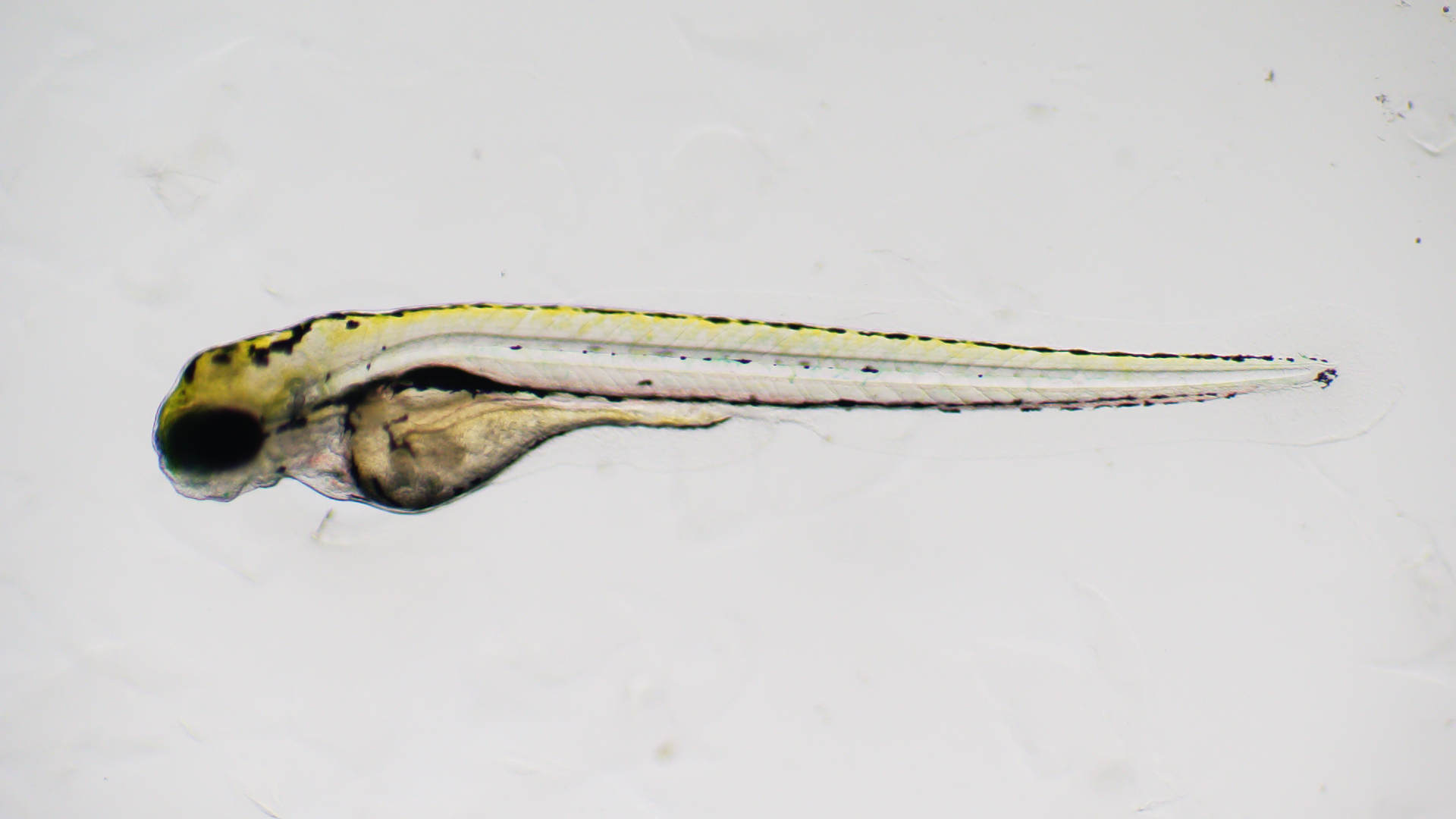

Supplement: Supplementary file 5 — Source data Fig. 4.2 [file 44321_2025_355_MOESM5_ESM.zip › S262I_4dpf.tif]

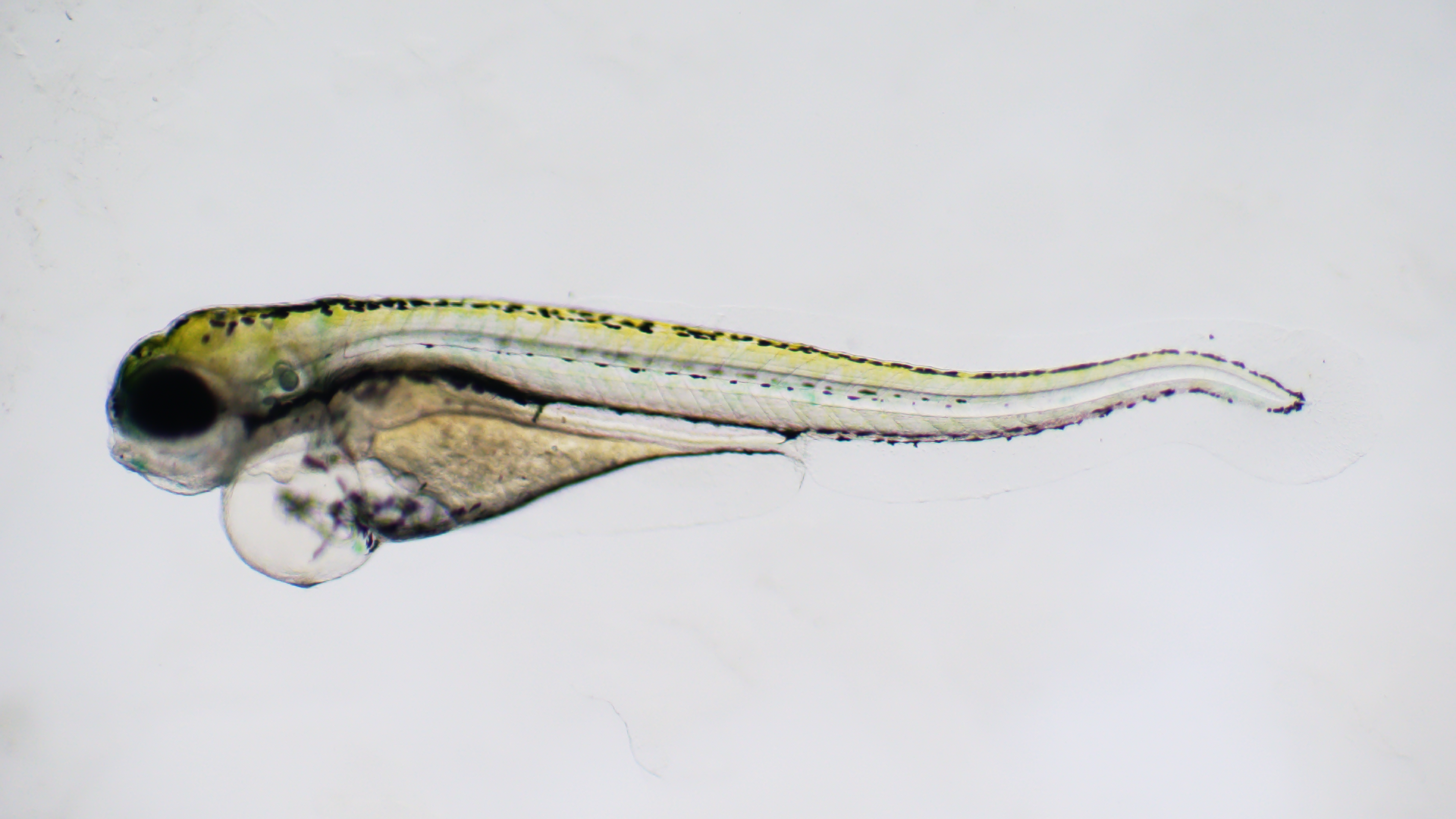

Supplement: Supplementary file 5 — Source data Fig. 4.2 [file 44321_2025_355_MOESM5_ESM.zip › S262I_5dpf.tif]

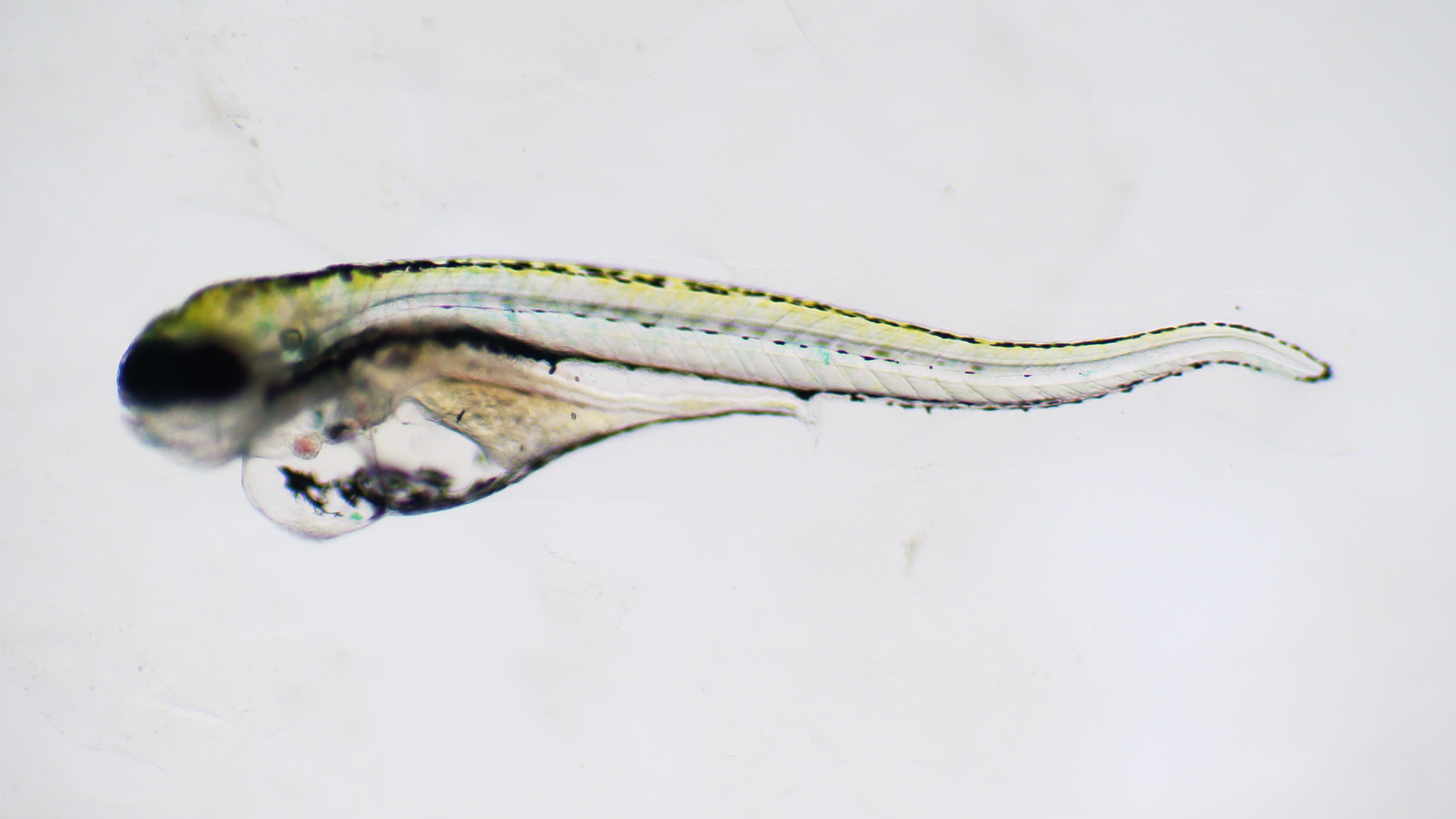

Supplement: Supplementary file 5 — Source data Fig. 4.2 [file 44321_2025_355_MOESM5_ESM.zip › S262I_6dpf.tif]

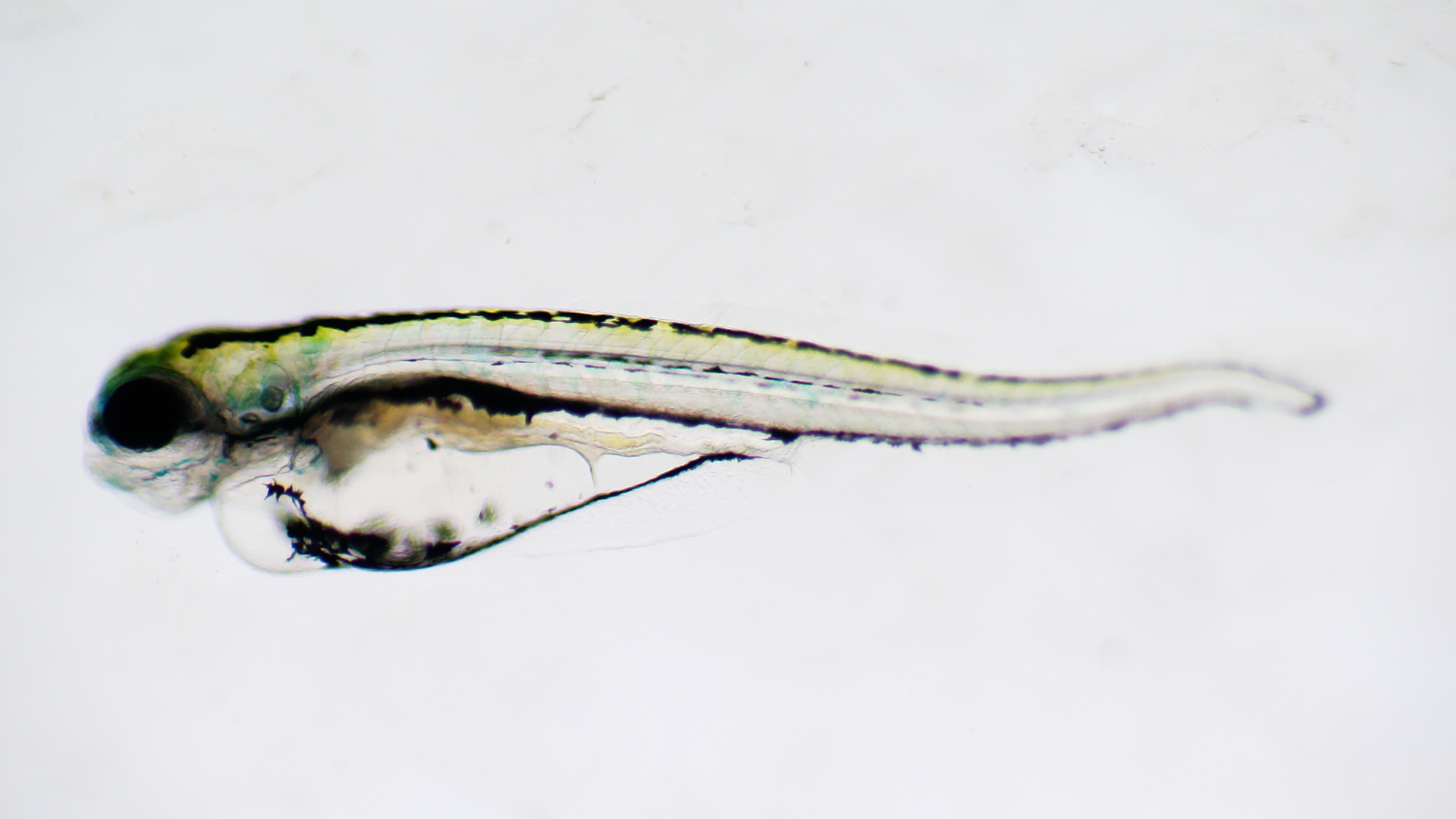

Supplement: Supplementary file 5 — Source data Fig. 4.2 [file 44321_2025_355_MOESM5_ESM.zip › S262I_7dpf.tif]

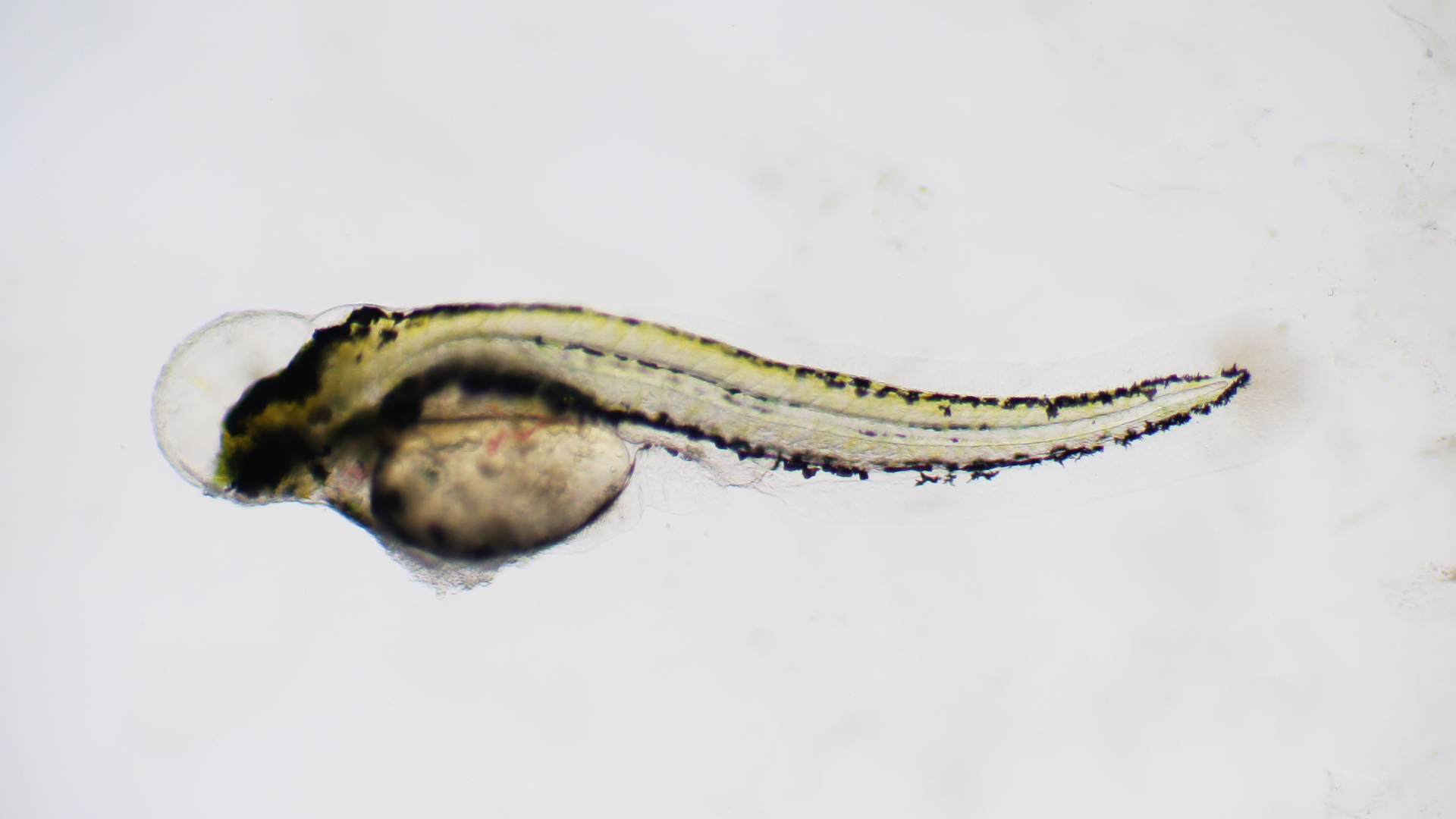

Supplement: Supplementary file 5 — Source data Fig. 4.2 [file 44321_2025_355_MOESM5_ESM.zip › S262I_8dpf.tif]

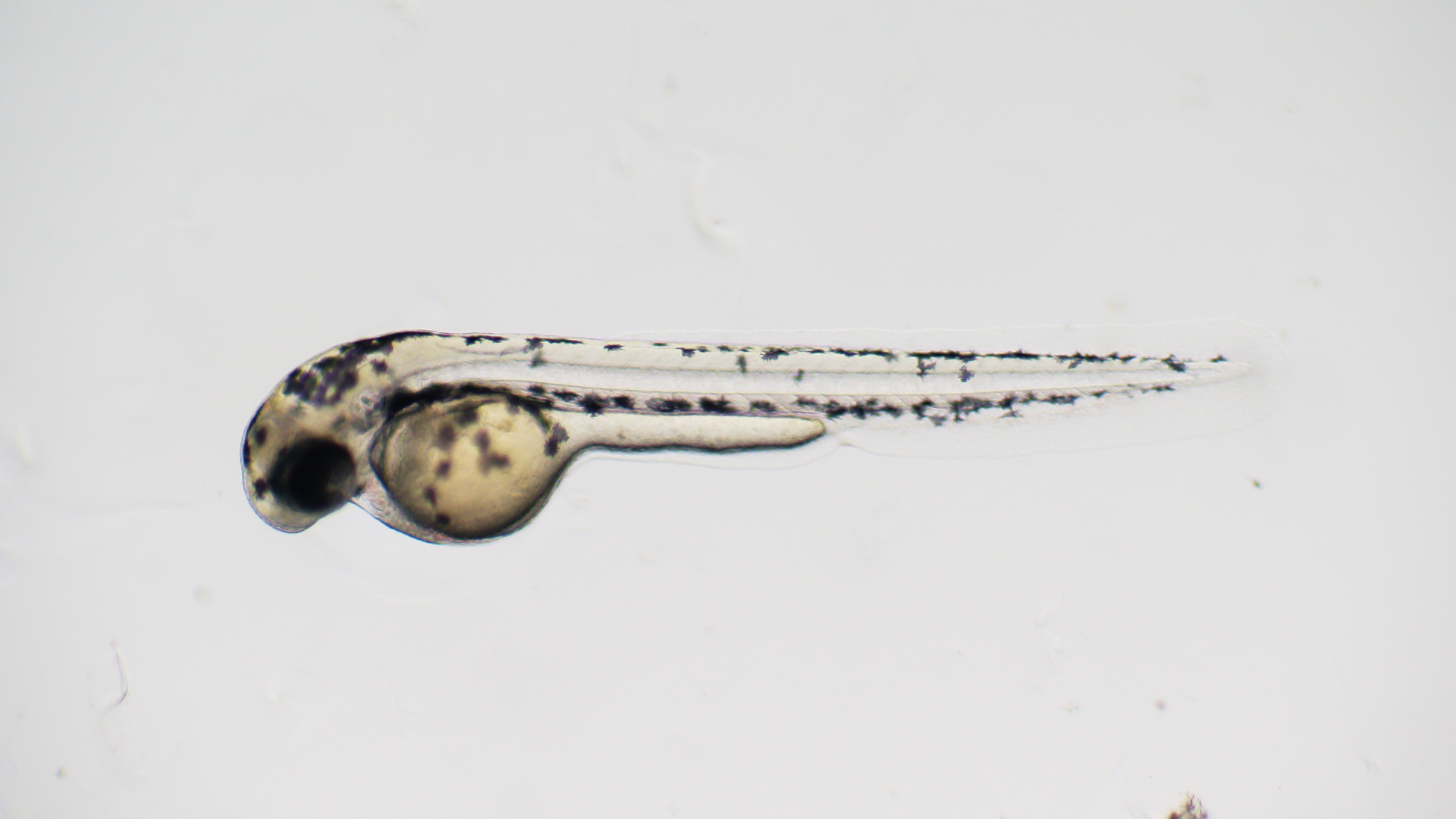

Supplement: Supplementary file 5 — Source data Fig. 4.2 [file 44321_2025_355_MOESM5_ESM.zip › smn_null_2dpf.tif]

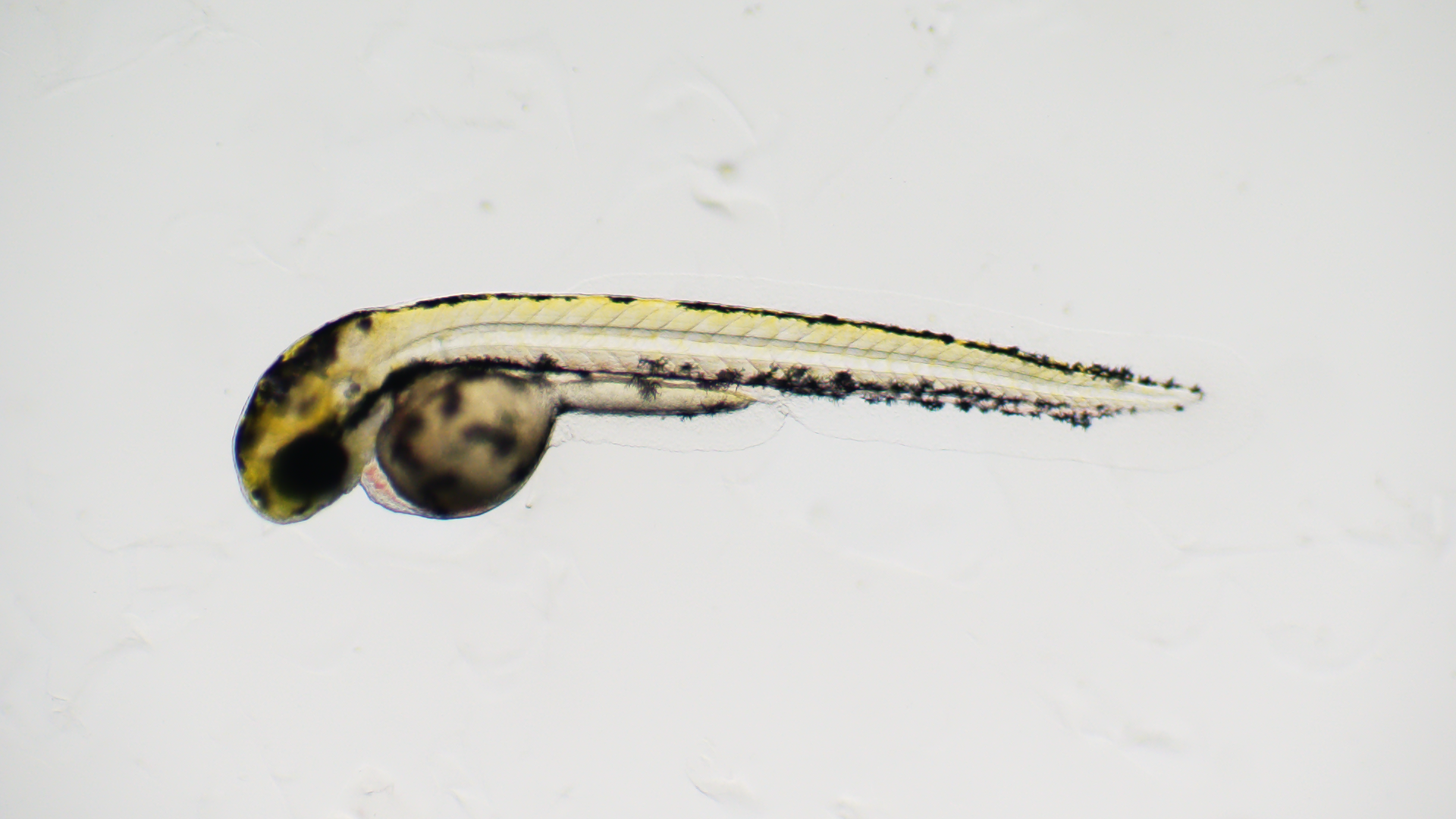

Supplement: Supplementary file 5 — Source data Fig. 4.2 [file 44321_2025_355_MOESM5_ESM.zip › smn_null_3dpf.tif]

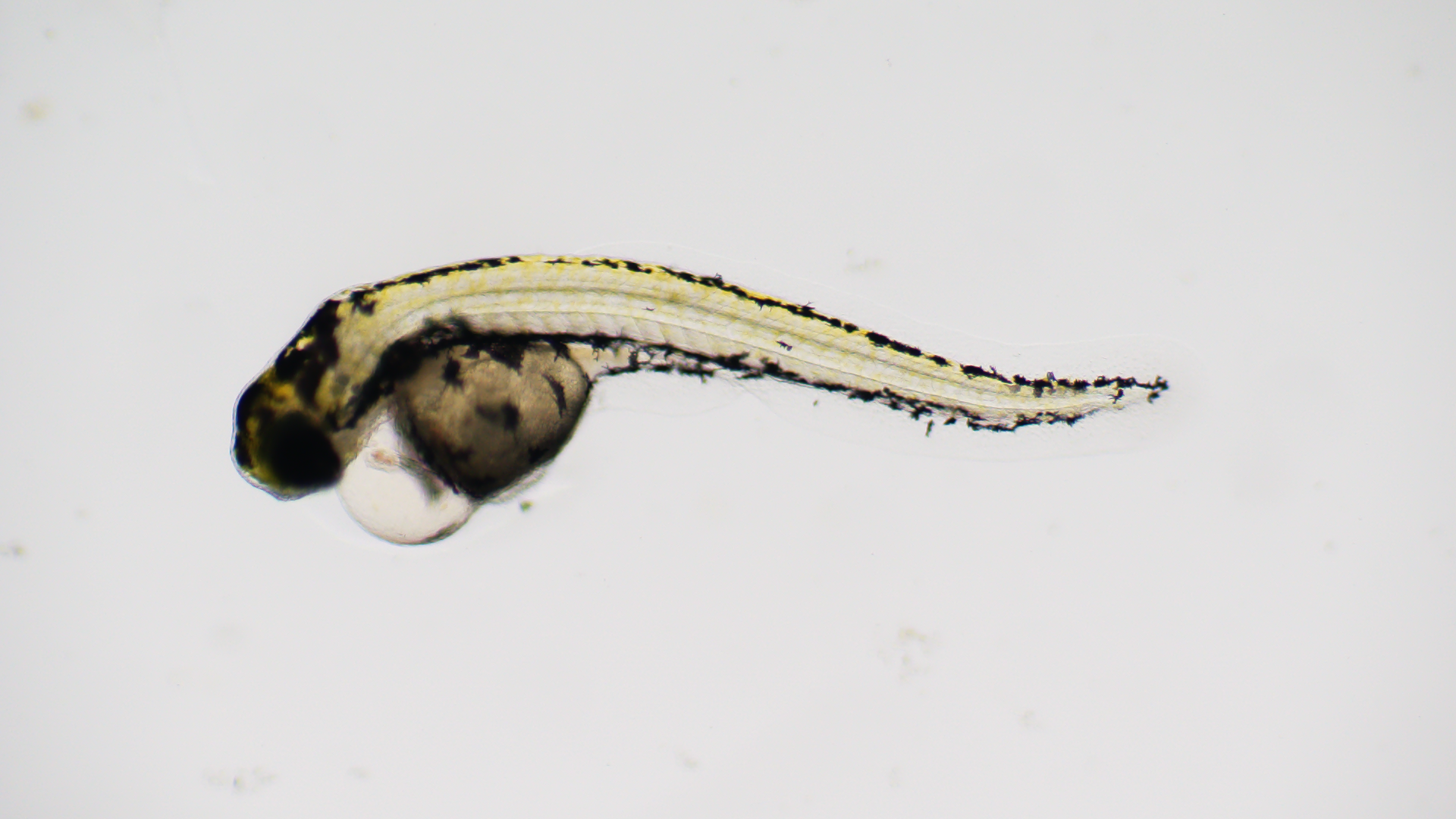

Supplement: Supplementary file 5 — Source data Fig. 4.2 [file 44321_2025_355_MOESM5_ESM.zip › smn_null_4dpf.tif]

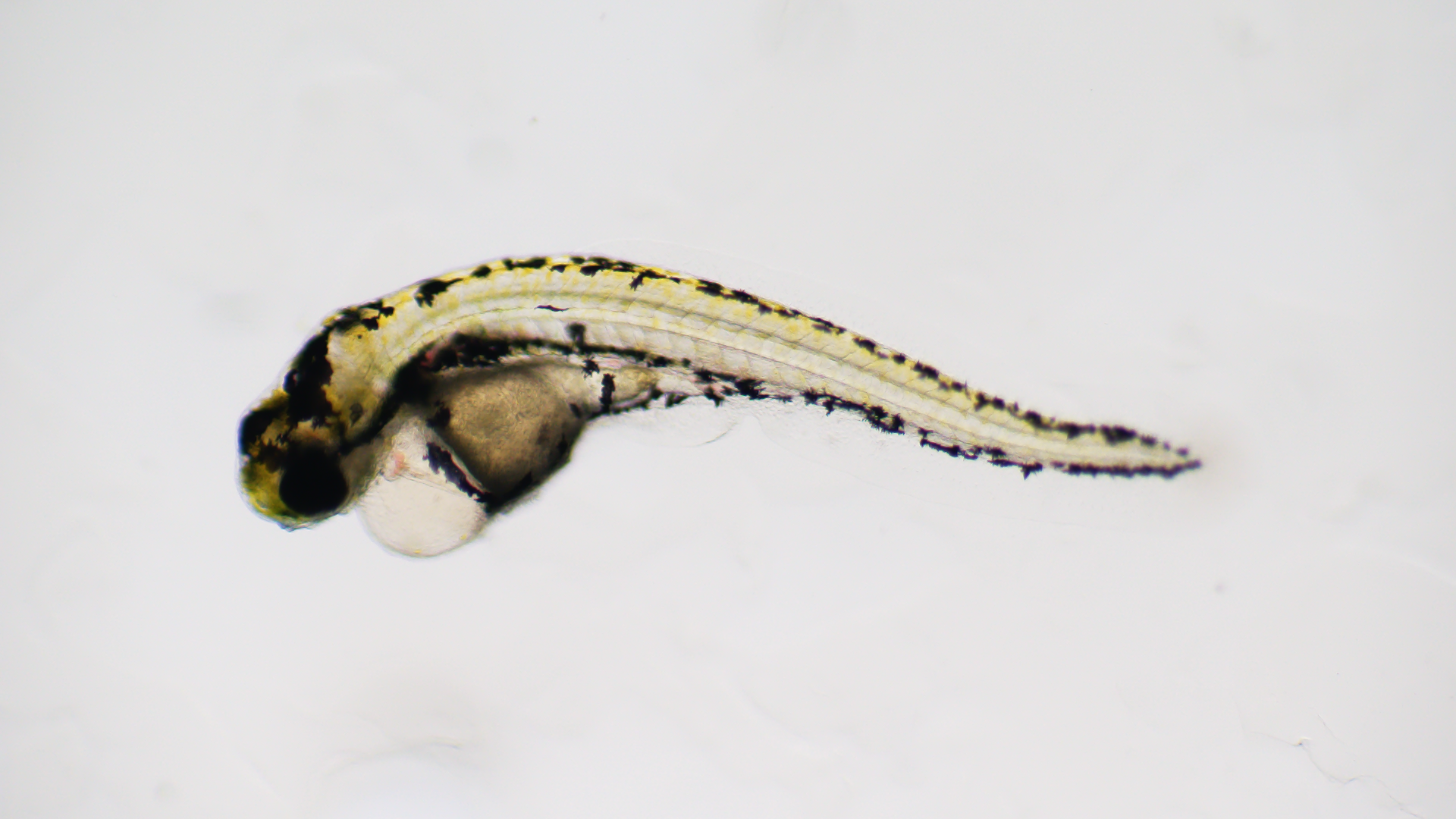

Supplement: Supplementary file 5 — Source data Fig. 4.2 [file 44321_2025_355_MOESM5_ESM.zip › smn_null_5dpf.tif]

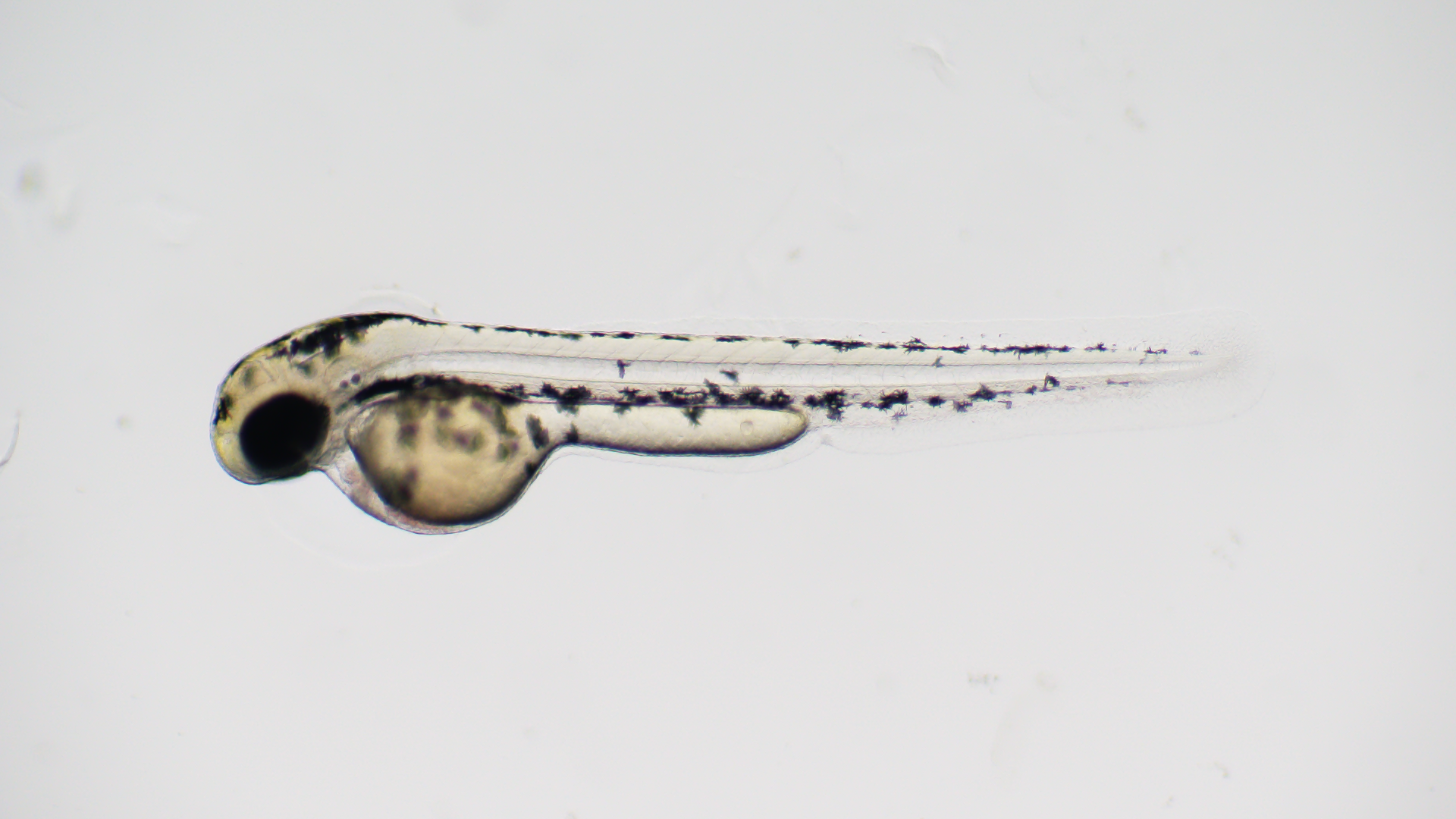

Supplement: Supplementary file 5 — Source data Fig. 4.2 [file 44321_2025_355_MOESM5_ESM.zip › T274I_2dpf.tif]

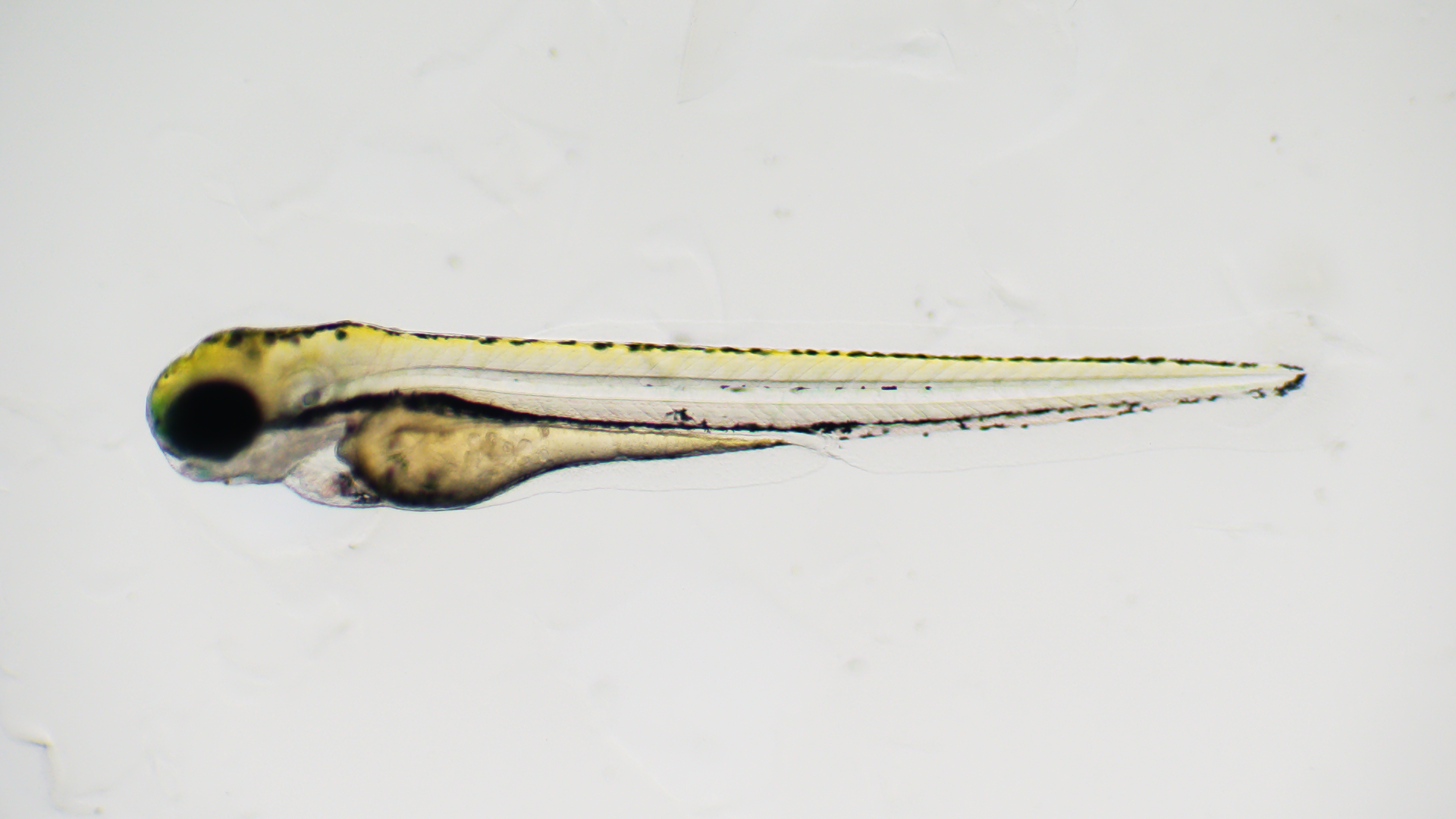

Supplement: Supplementary file 5 — Source data Fig. 4.2 [file 44321_2025_355_MOESM5_ESM.zip › T274I_3dpf.tif]

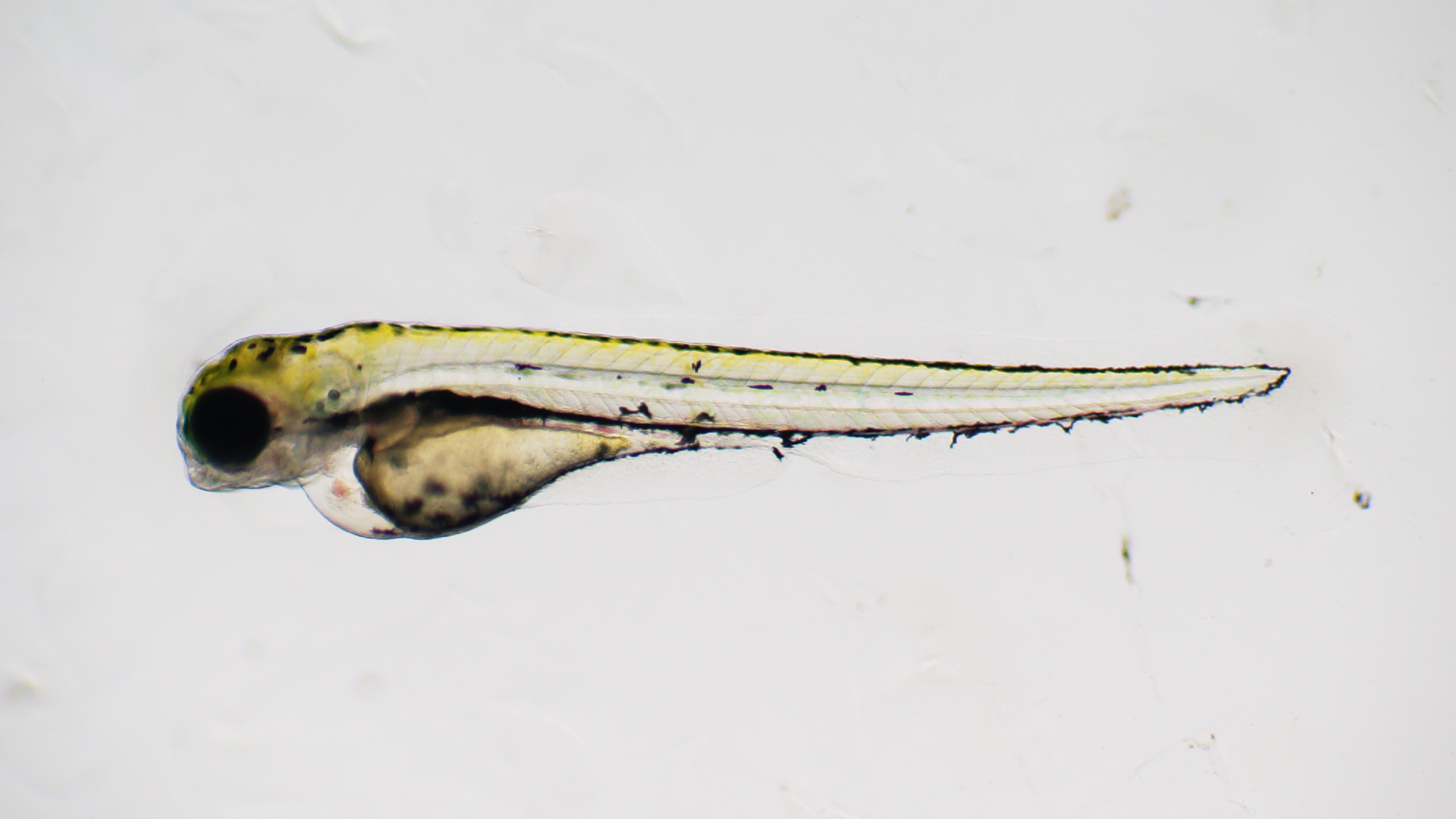

Supplement: Supplementary file 5 — Source data Fig. 4.2 [file 44321_2025_355_MOESM5_ESM.zip › T274I_4dpf.tif]

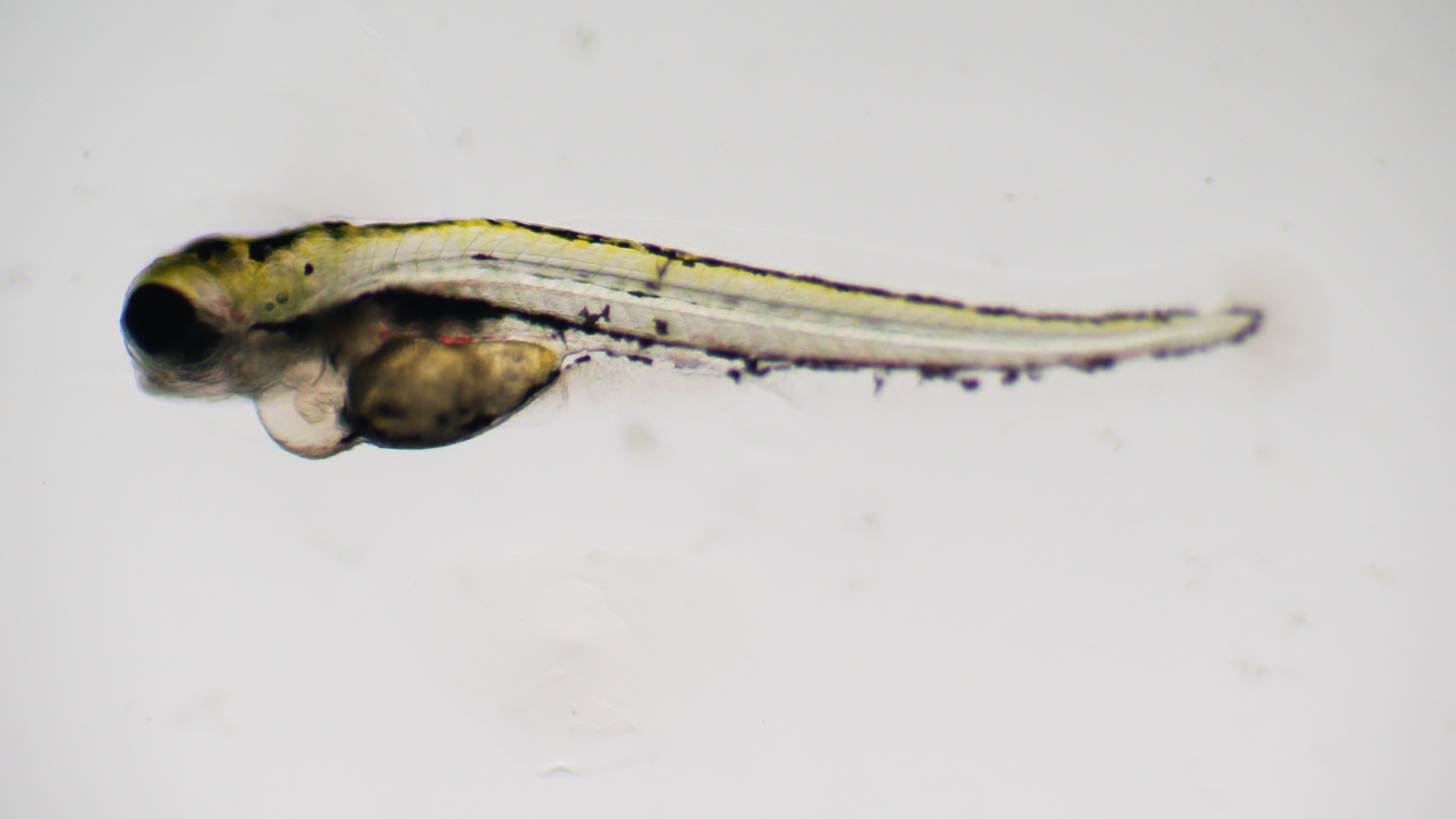

Supplement: Supplementary file 5 — Source data Fig. 4.2 [file 44321_2025_355_MOESM5_ESM.zip › T274I_5dpf.tif]

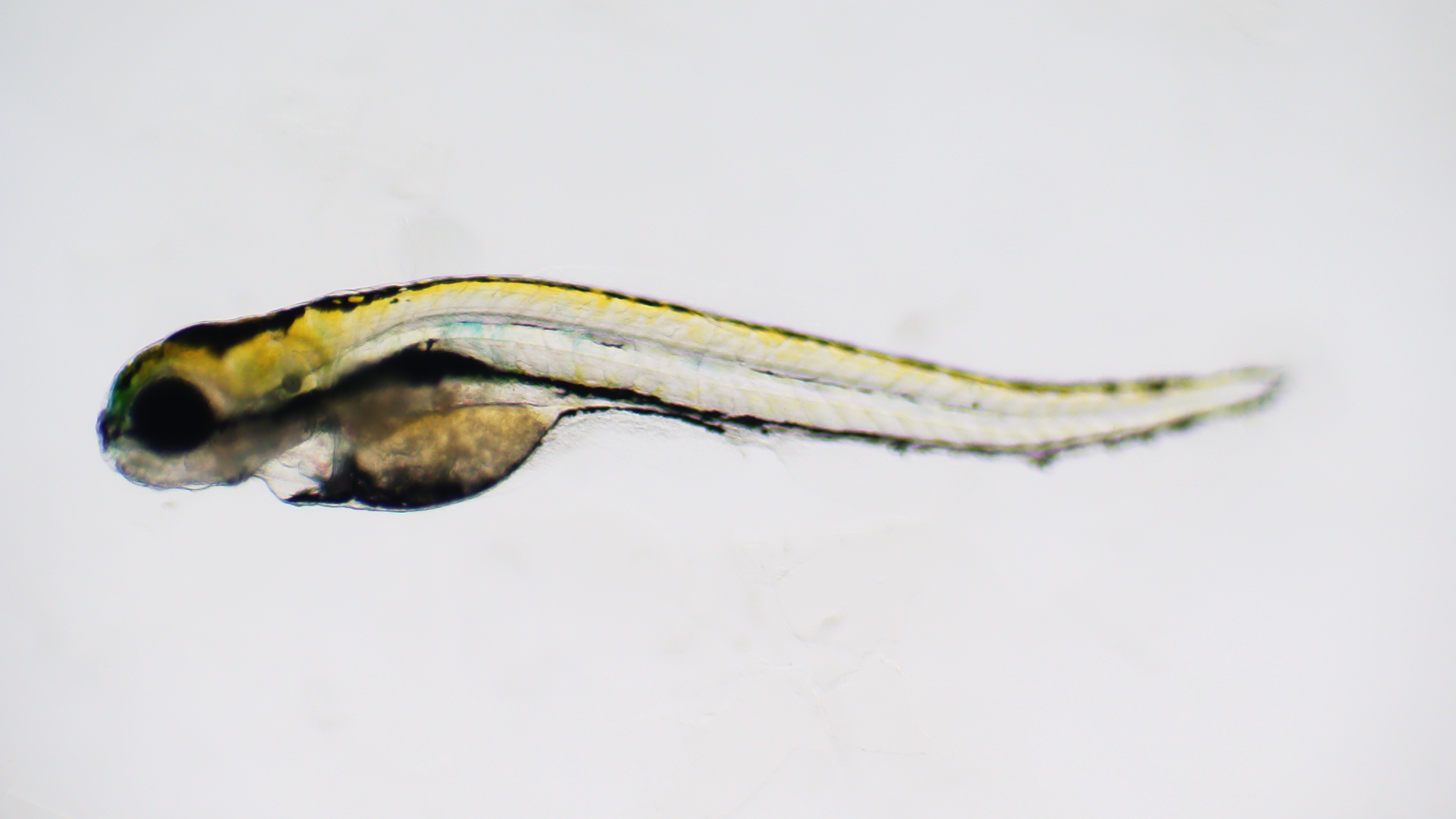

Supplement: Supplementary file 5 — Source data Fig. 4.2 [file 44321_2025_355_MOESM5_ESM.zip › T274I_6dpf.tif]

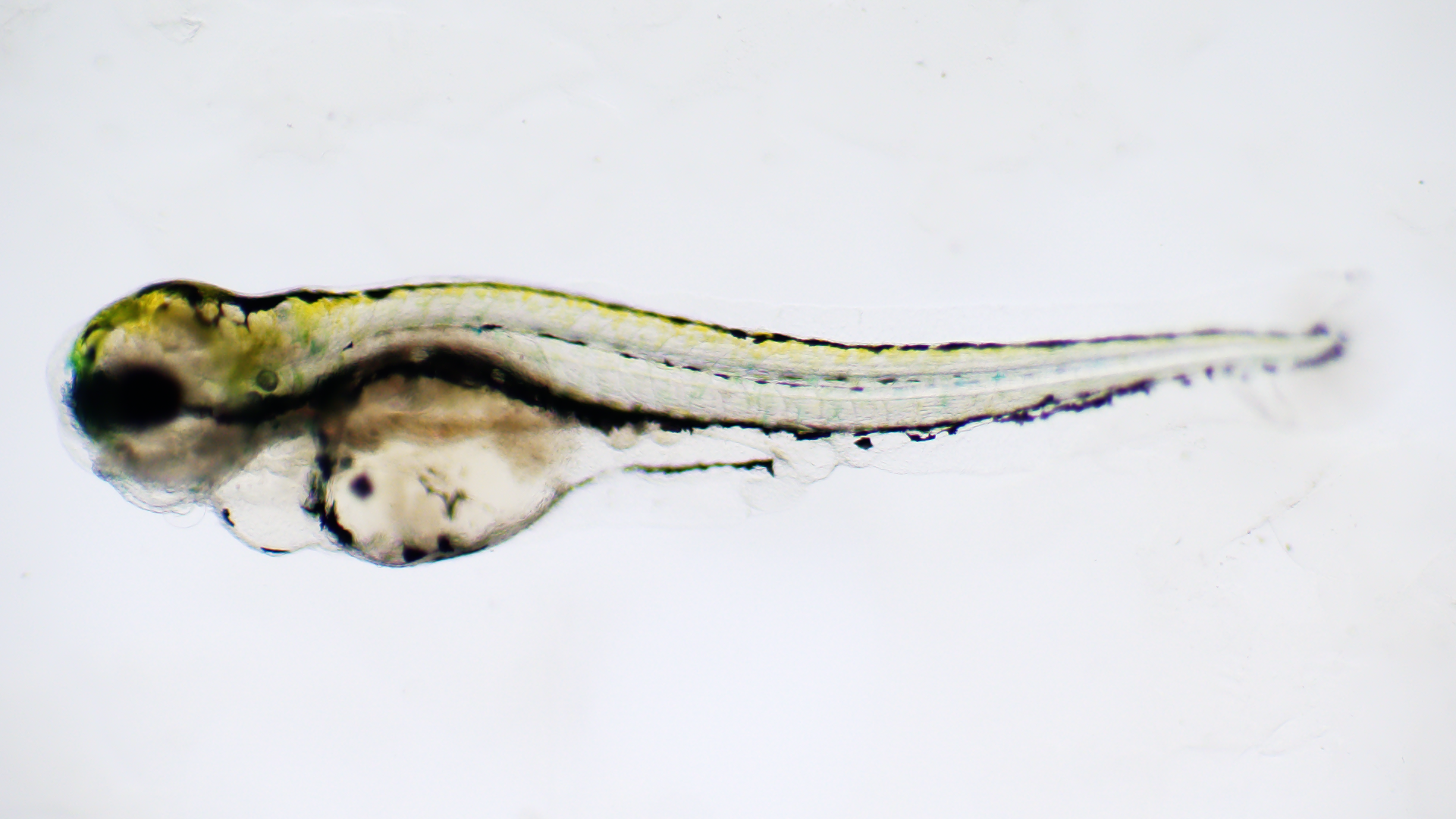

Supplement: Supplementary file 5 — Source data Fig. 4.2 [file 44321_2025_355_MOESM5_ESM.zip › T274I_7dpf.tif]

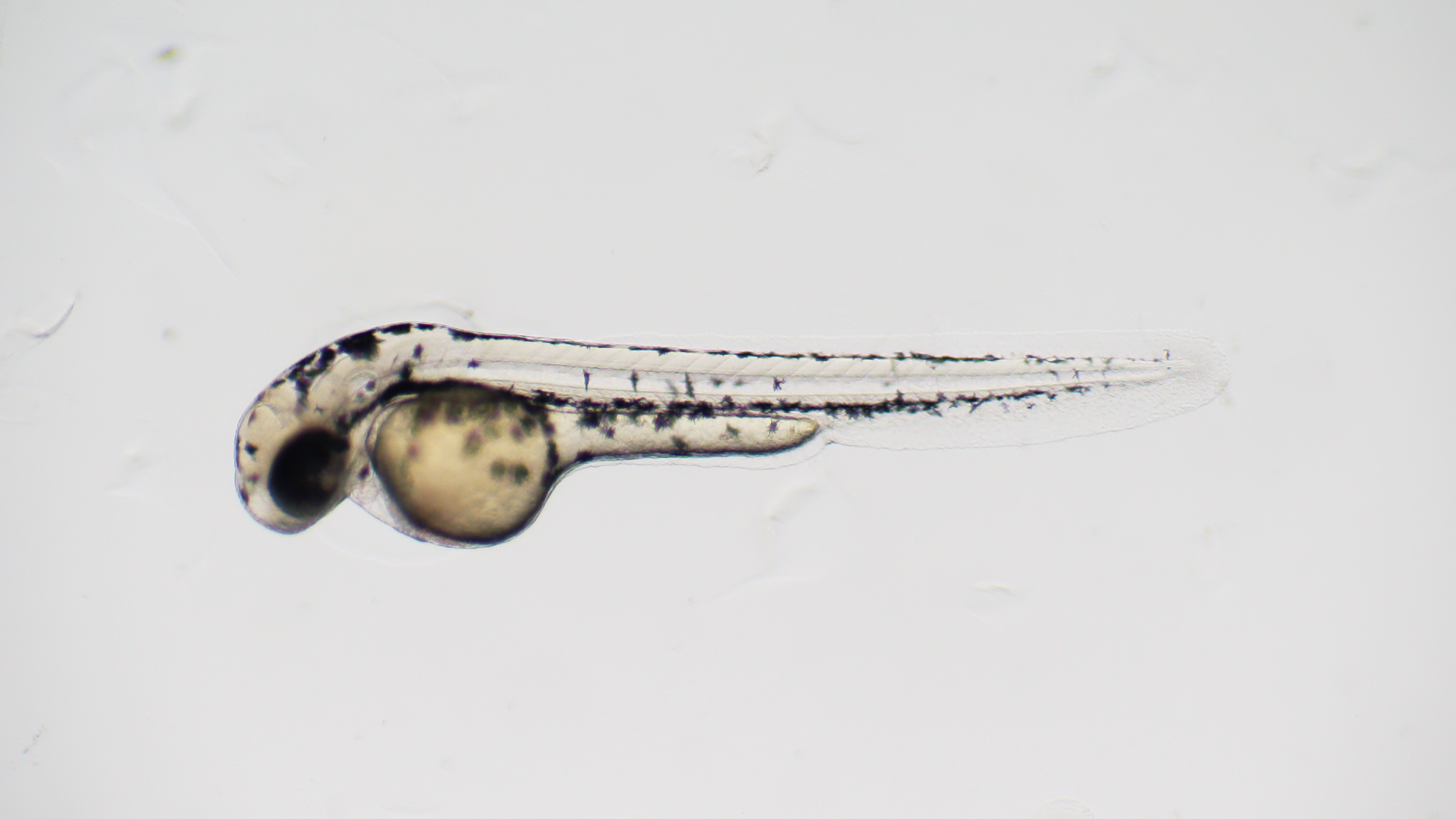

Supplement: Supplementary file 5 — Source data Fig. 4.2 [file 44321_2025_355_MOESM5_ESM.zip › Tg(SMN1)_2dpf.tif]

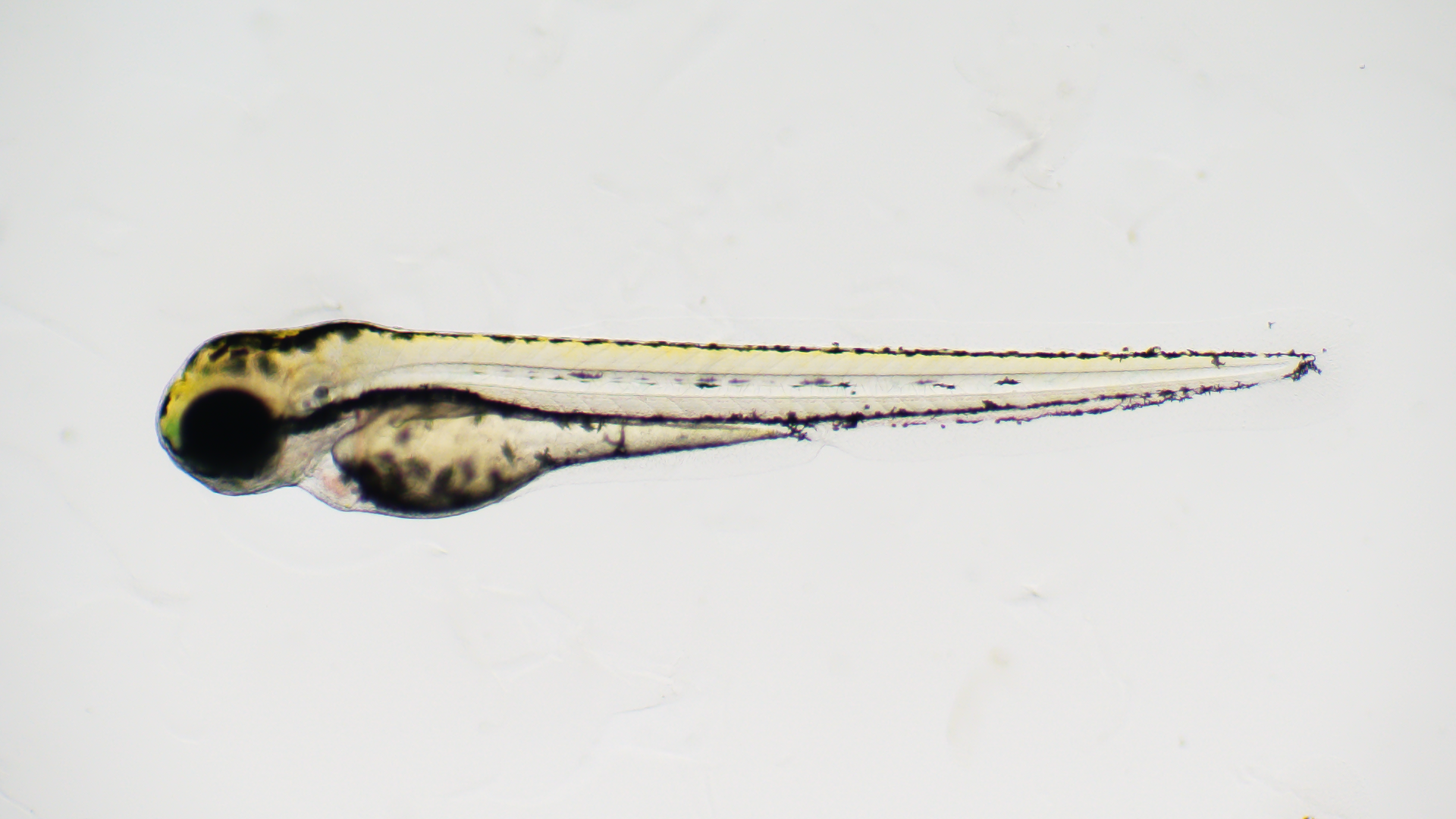

Supplement: Supplementary file 5 — Source data Fig. 4.2 [file 44321_2025_355_MOESM5_ESM.zip › Tg(SMN1)_3dpf.tif]

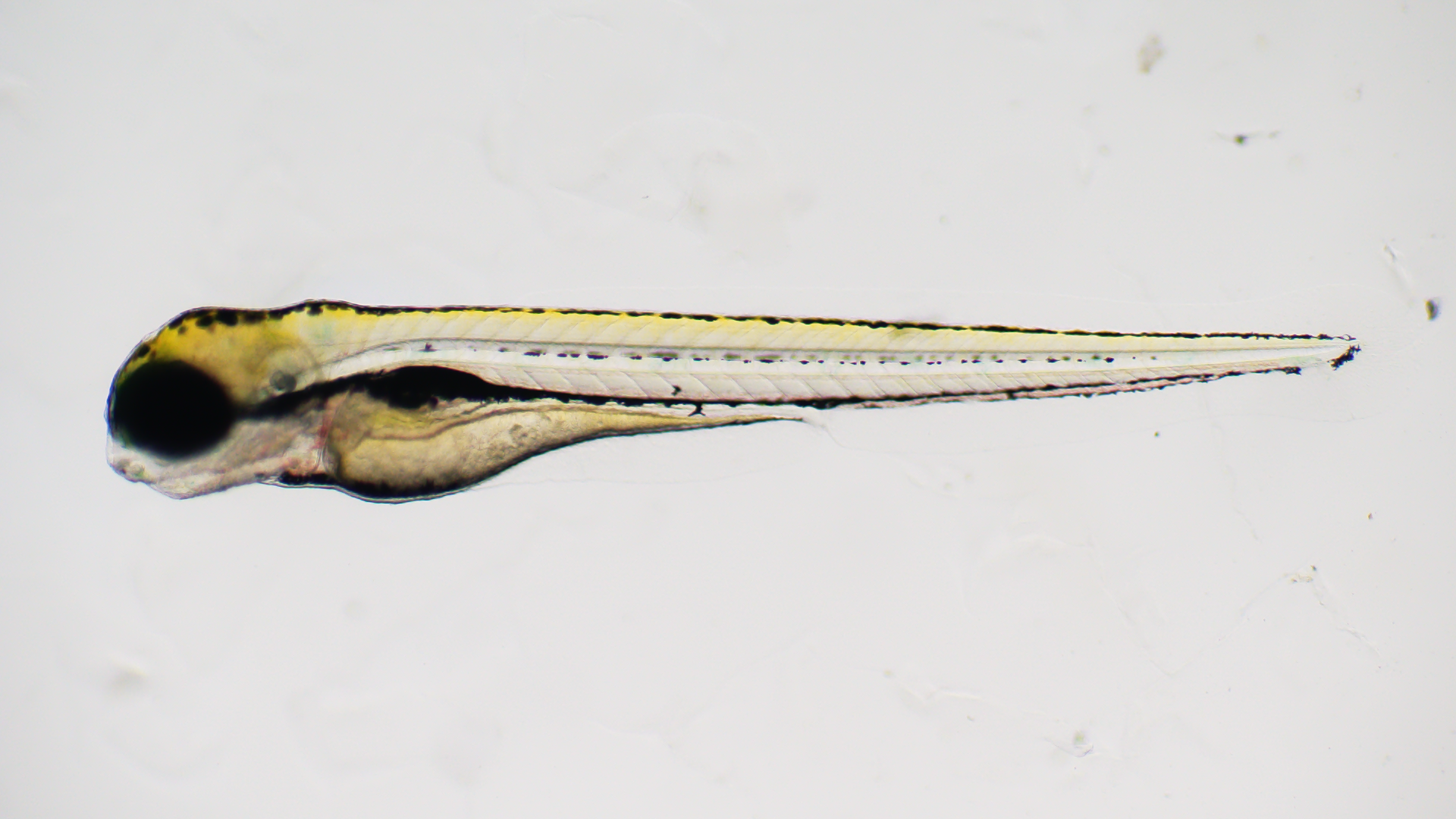

Supplement: Supplementary file 5 — Source data Fig. 4.2 [file 44321_2025_355_MOESM5_ESM.zip › Tg(SMN1)_4dpf.tif]

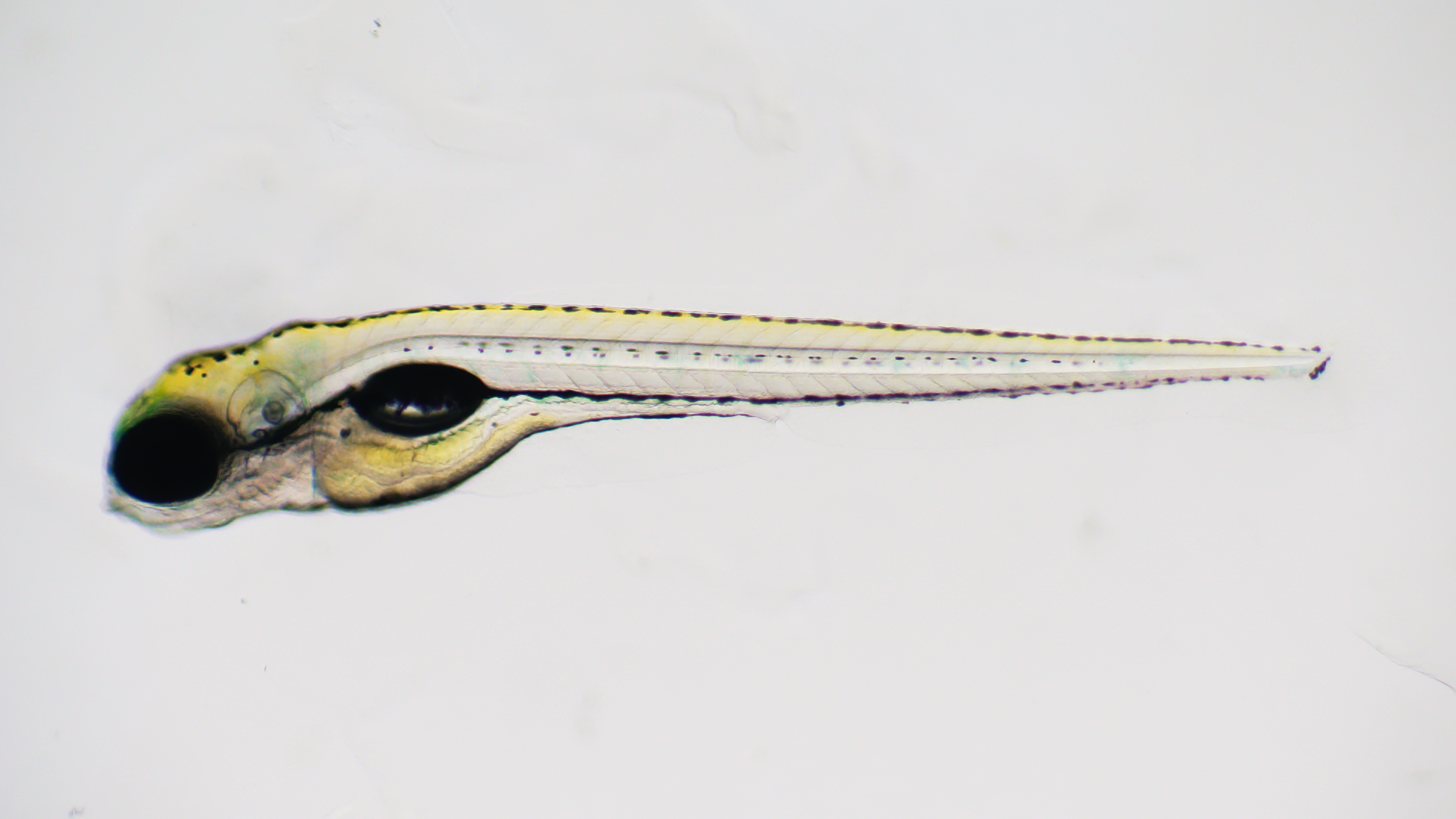

Supplement: Supplementary file 5 — Source data Fig. 4.2 [file 44321_2025_355_MOESM5_ESM.zip › Tg(SMN1)_5dpf.tif]

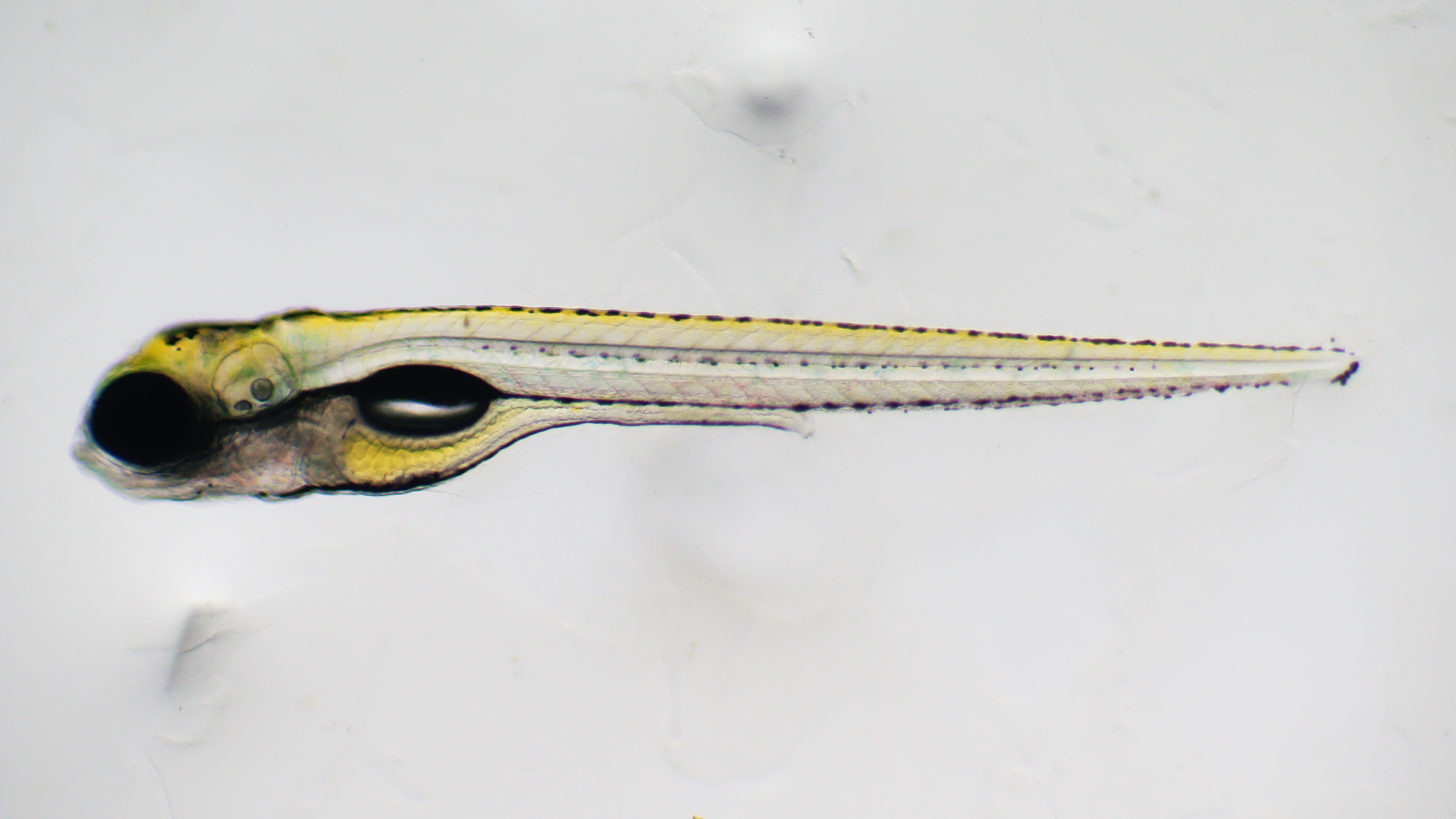

Supplement: Supplementary file 5 — Source data Fig. 4.2 [file 44321_2025_355_MOESM5_ESM.zip › Tg(SMN1)_6dpf.tif]

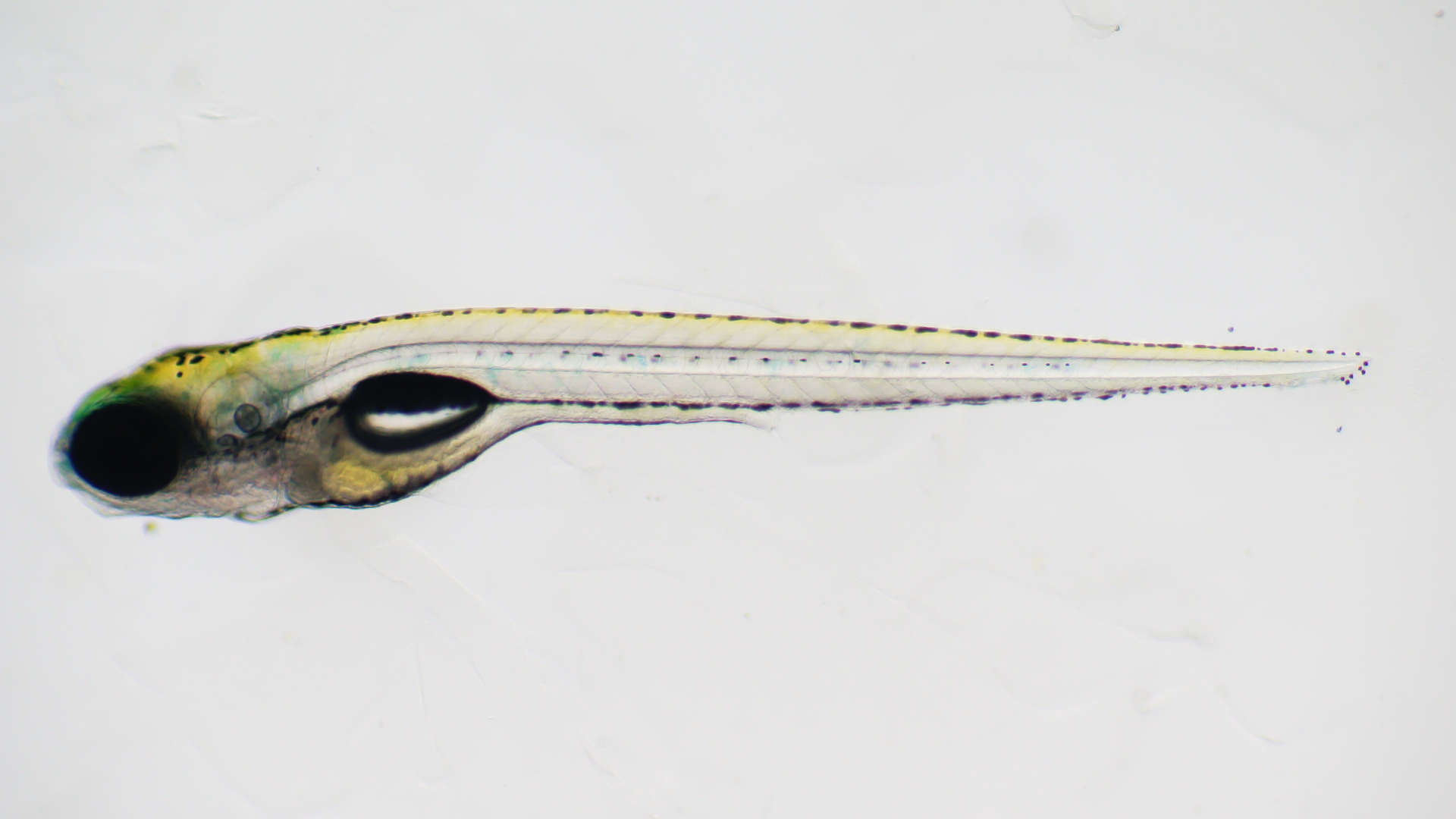

Supplement: Supplementary file 5 — Source data Fig. 4.2 [file 44321_2025_355_MOESM5_ESM.zip › Tg(SMN1)_7dpf.tif]

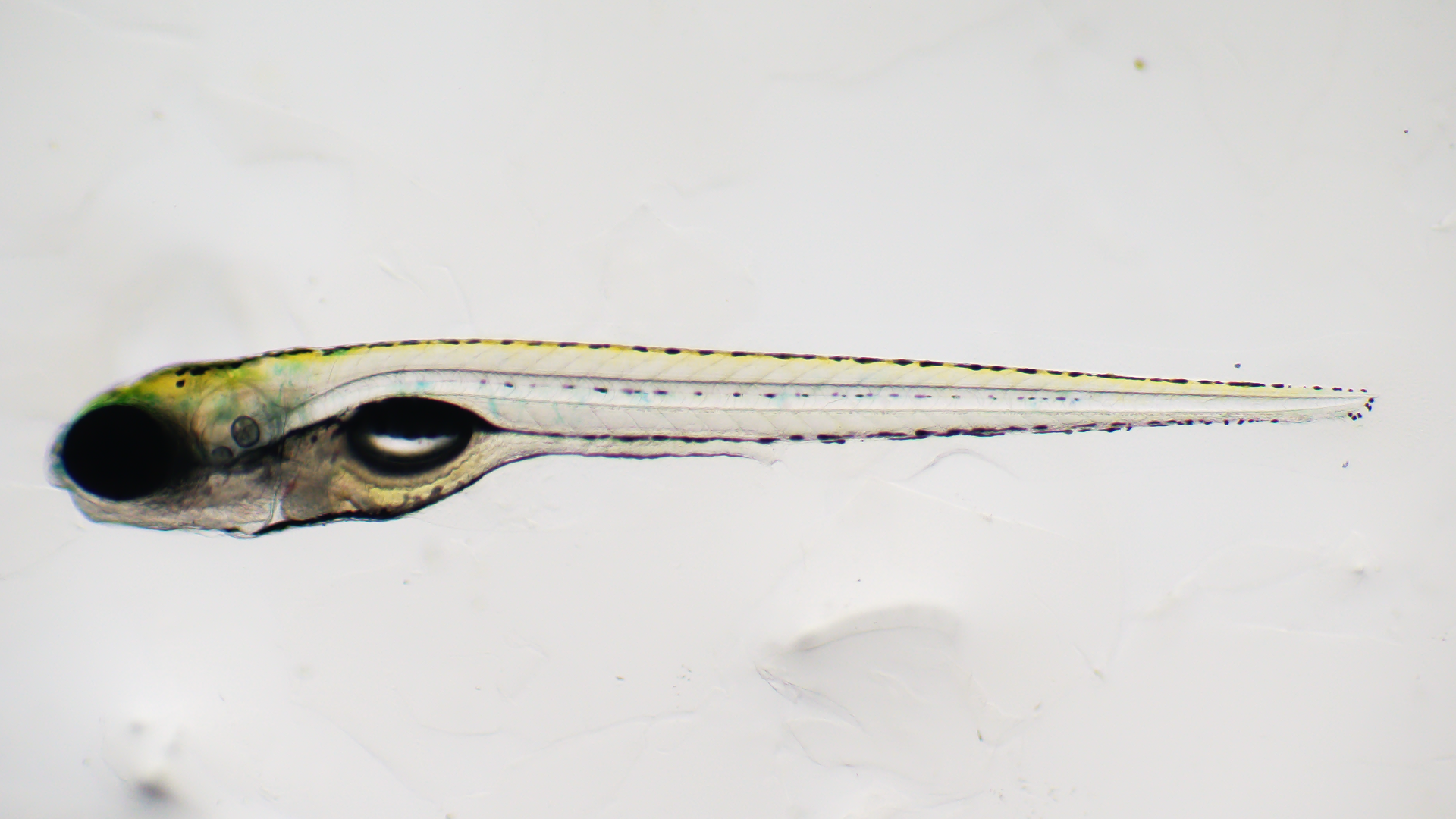

Supplement: Supplementary file 5 — Source data Fig. 4.2 [file 44321_2025_355_MOESM5_ESM.zip › Tg(SMN1)_8dpf.tif]

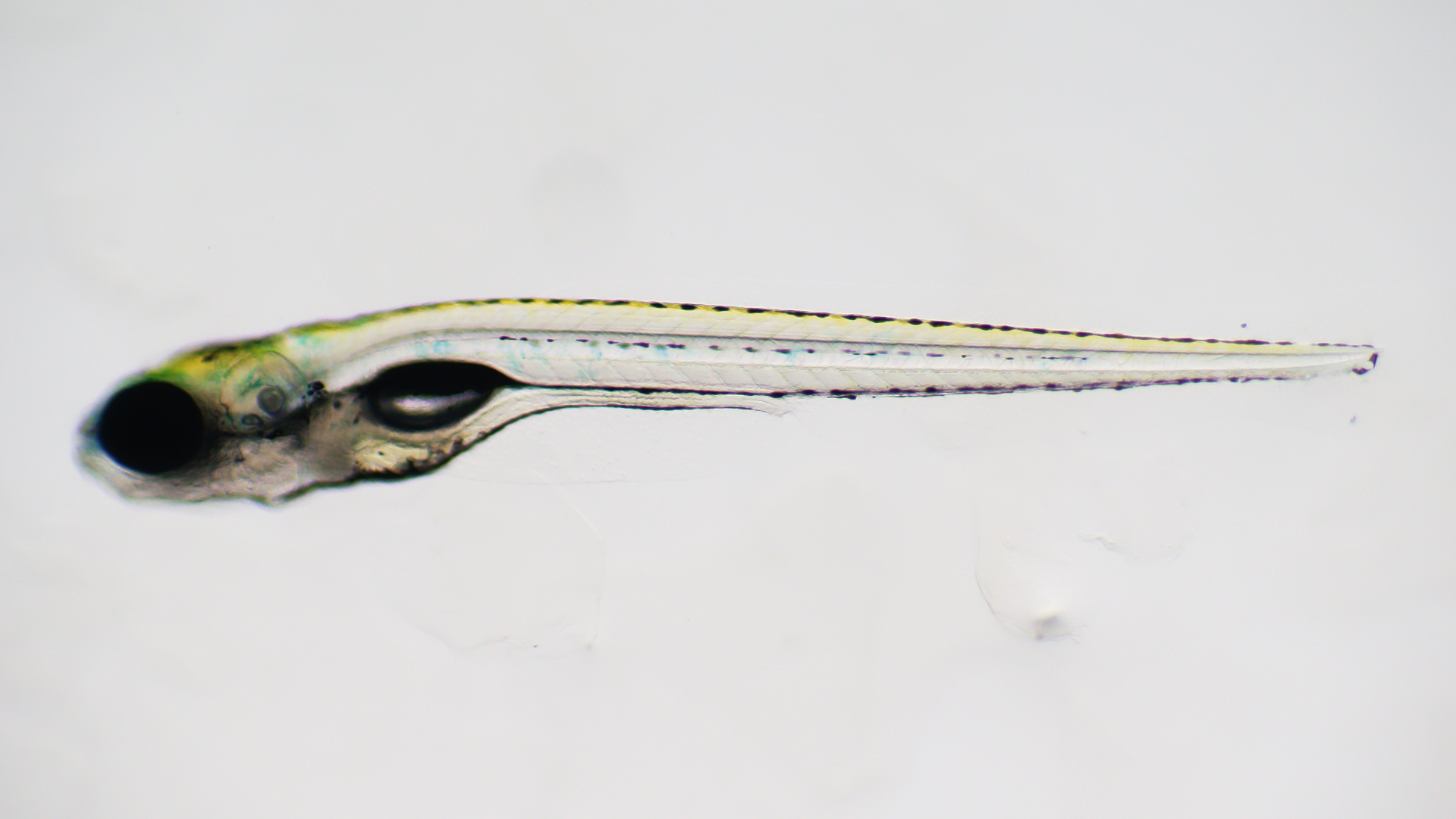

Supplement: Supplementary file 5 — Source data Fig. 4.2 [file 44321_2025_355_MOESM5_ESM.zip › Tg(SMN1)_9dpf.tif]

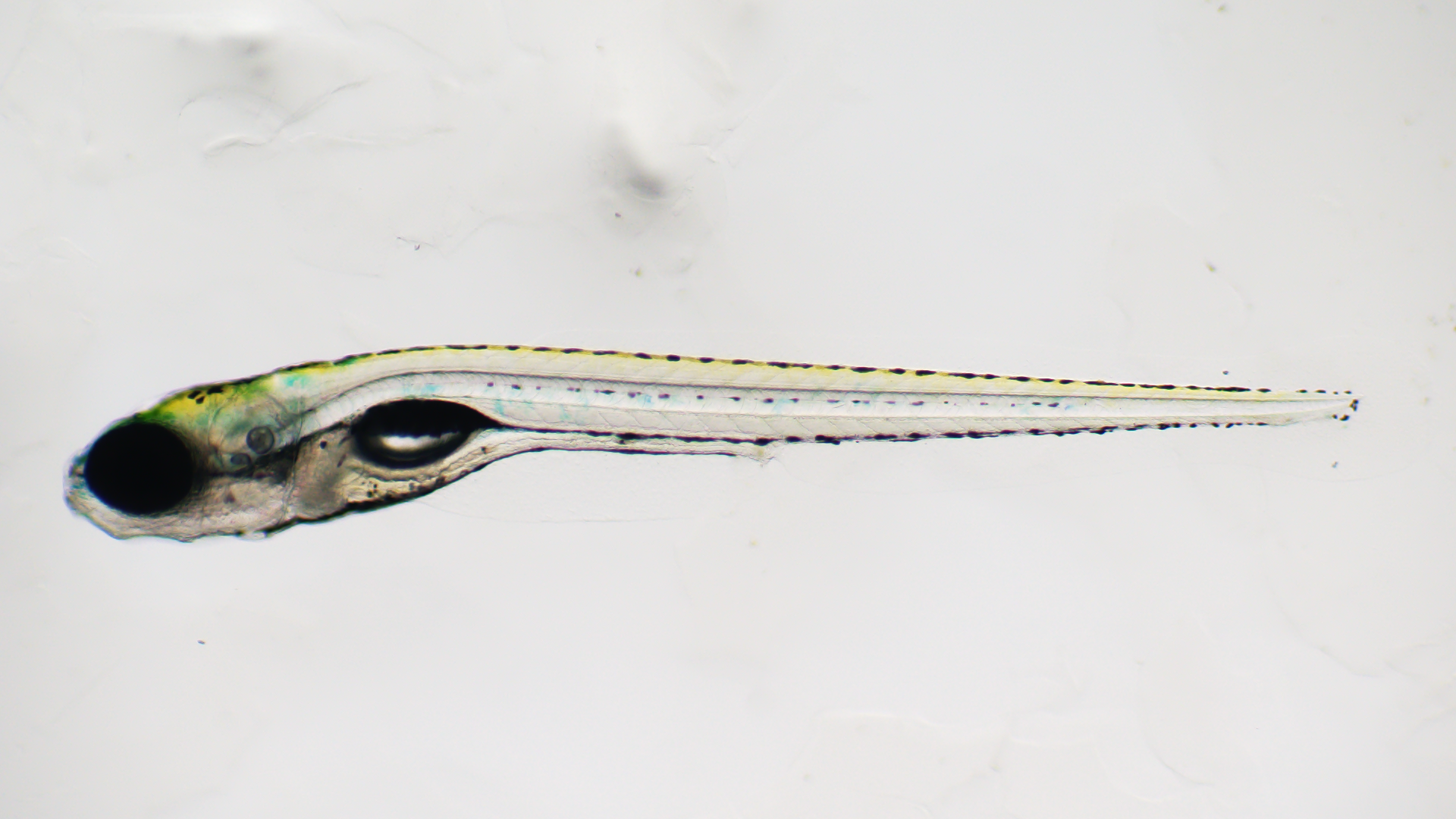

Supplement: Supplementary file 5 — Source data Fig. 4.2 [file 44321_2025_355_MOESM5_ESM.zip › Tg(SMN1)_10dpf.tif]

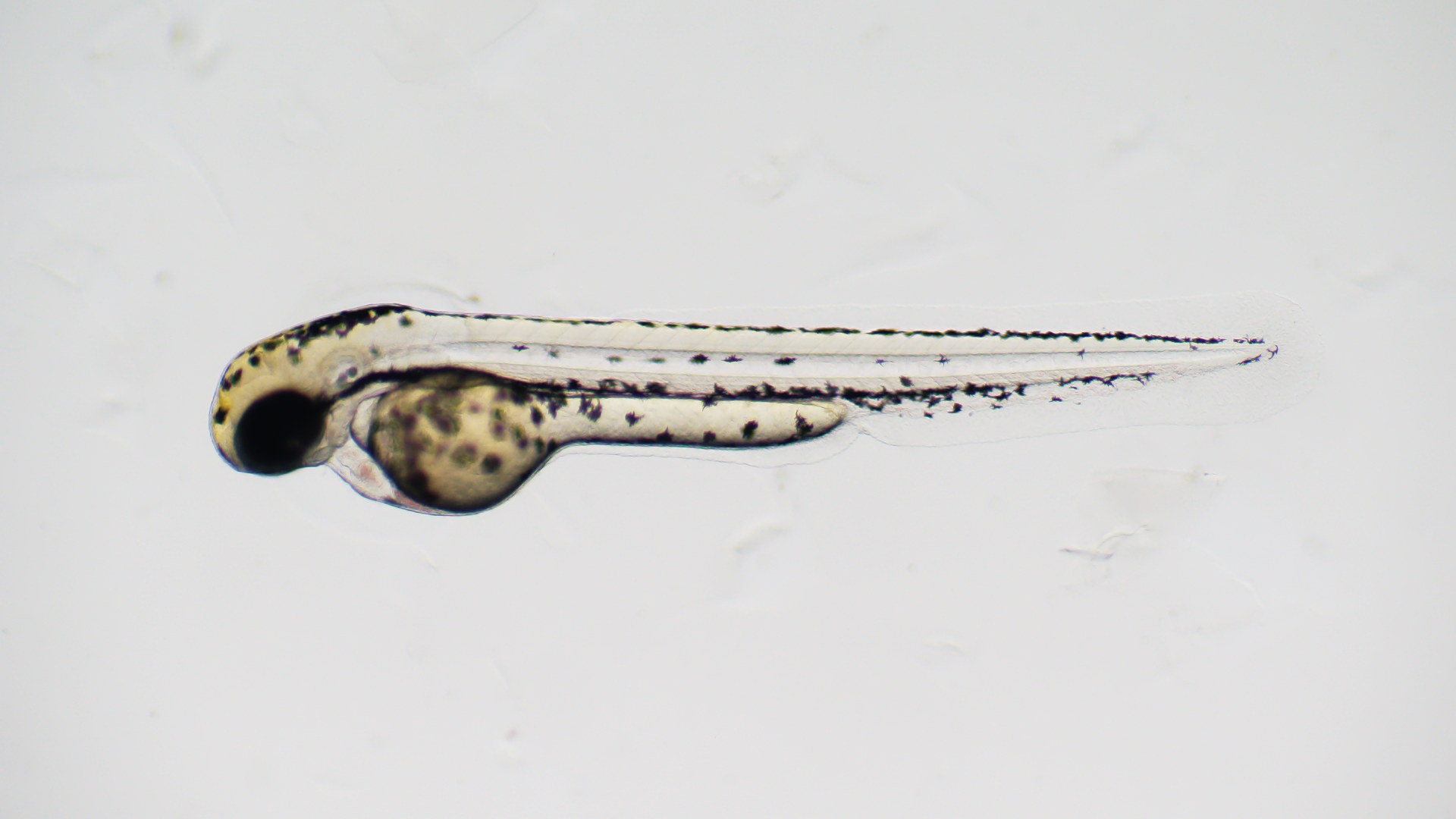

Supplement: Supplementary file 5 — Source data Fig. 4.2 [file 44321_2025_355_MOESM5_ESM.zip › WT_2dpf.tif]

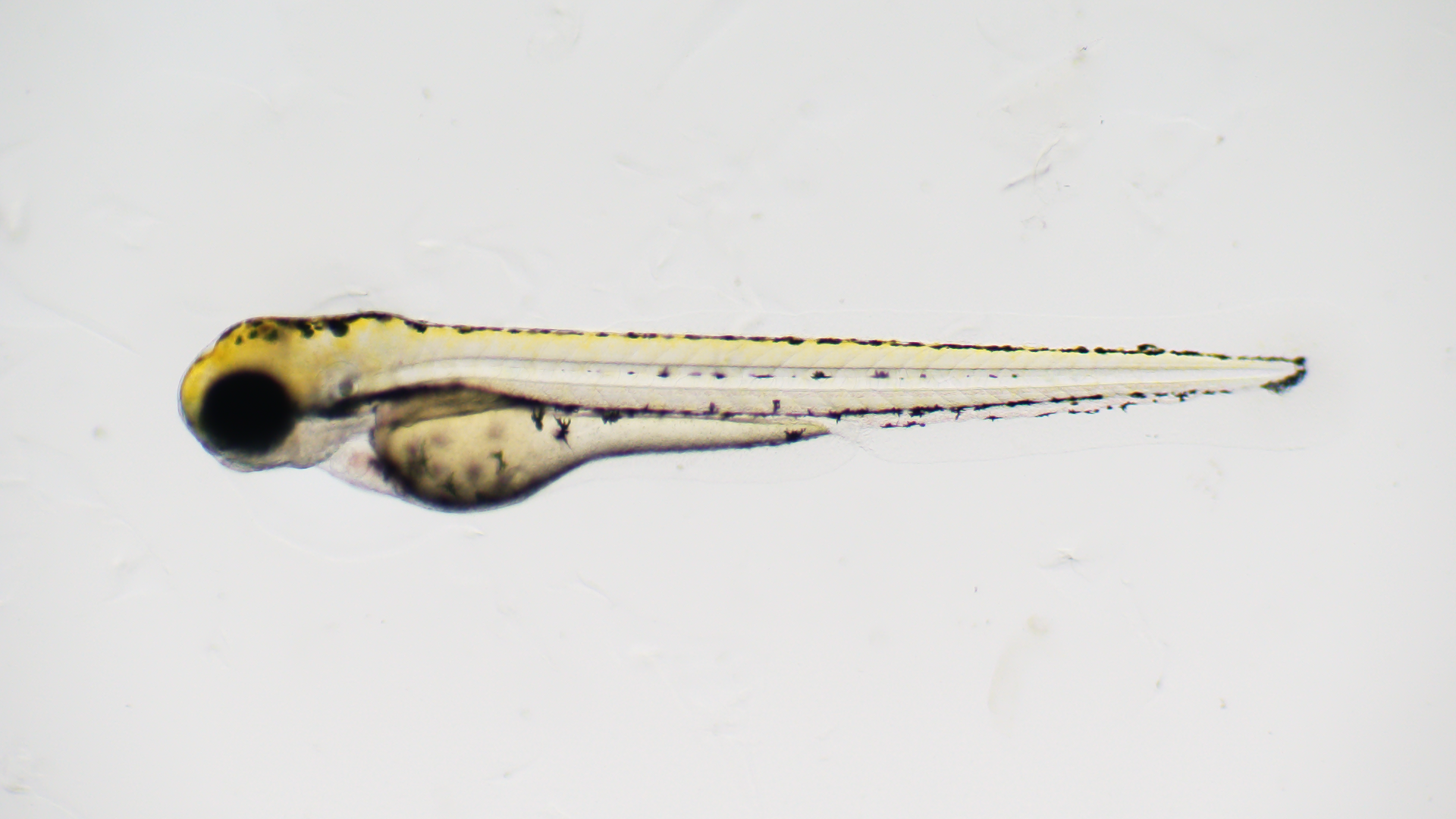

Supplement: Supplementary file 5 — Source data Fig. 4.2 [file 44321_2025_355_MOESM5_ESM.zip › WT_3dpf.tif]

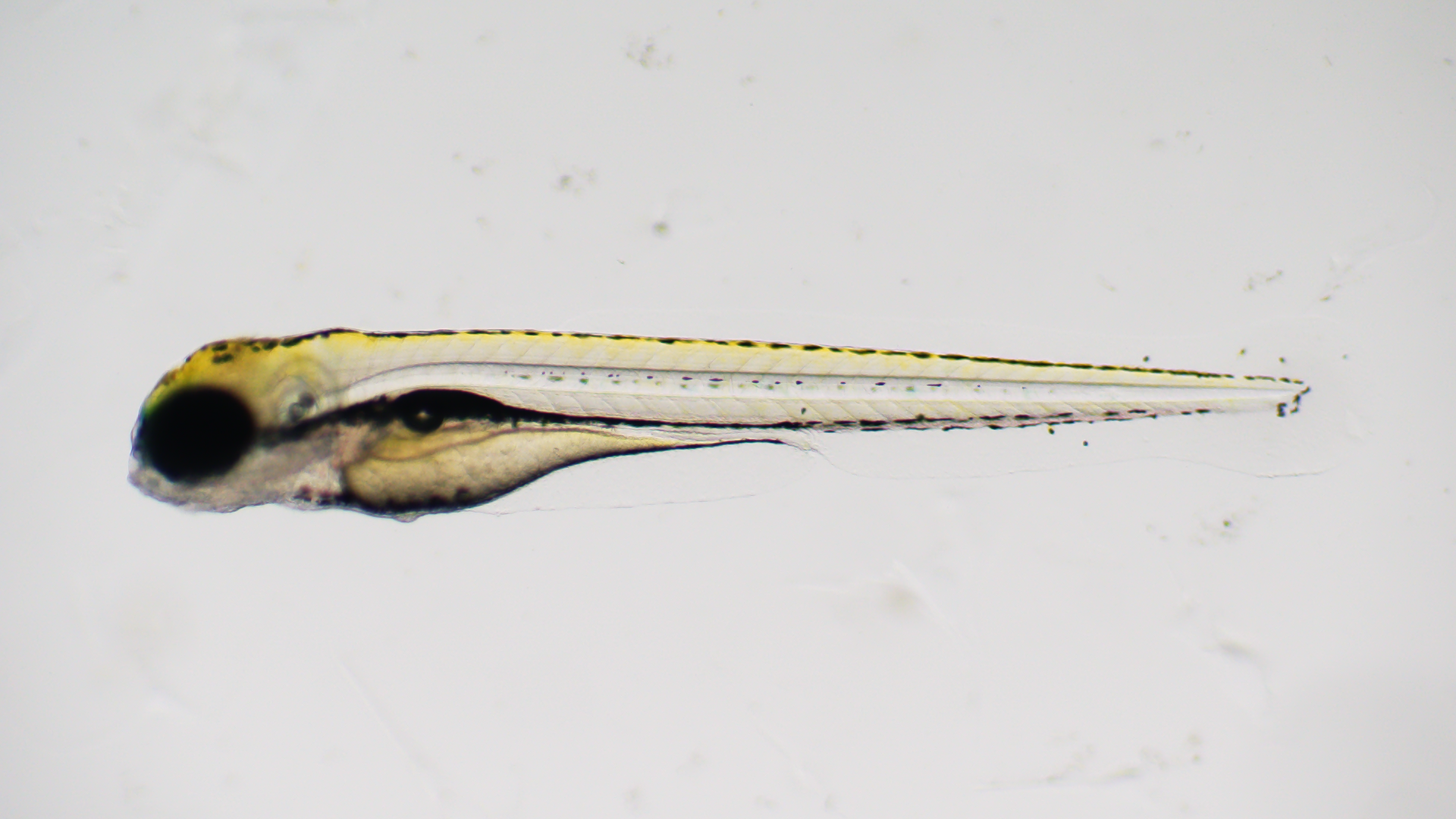

Supplement: Supplementary file 5 — Source data Fig. 4.2 [file 44321_2025_355_MOESM5_ESM.zip › WT_4dpf.tif]

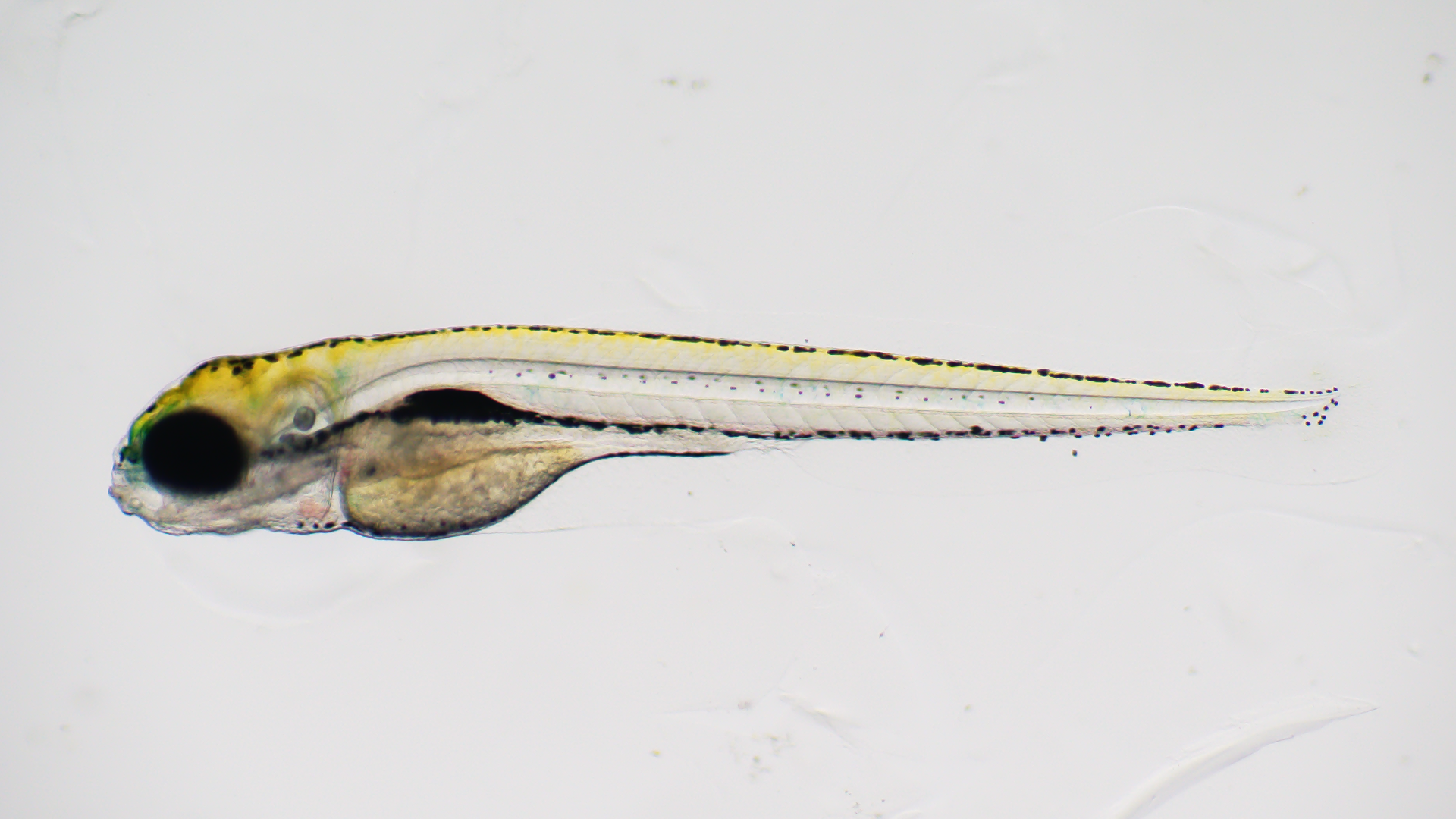

Supplement: Supplementary file 5 — Source data Fig. 4.2 [file 44321_2025_355_MOESM5_ESM.zip › WT_5dpf.tif]

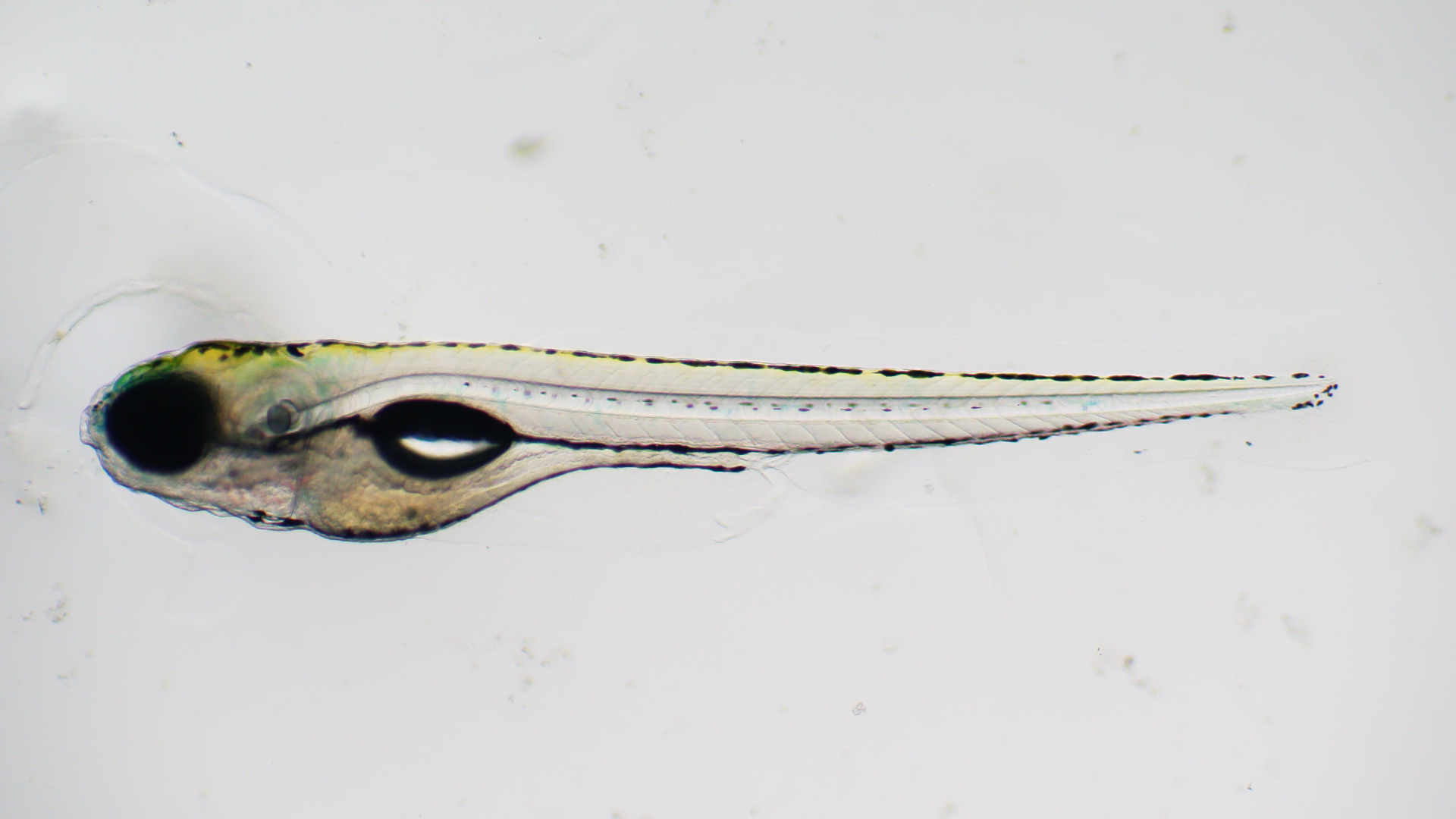

Supplement: Supplementary file 5 — Source data Fig. 4.2 [file 44321_2025_355_MOESM5_ESM.zip › WT_6dpf.tif]

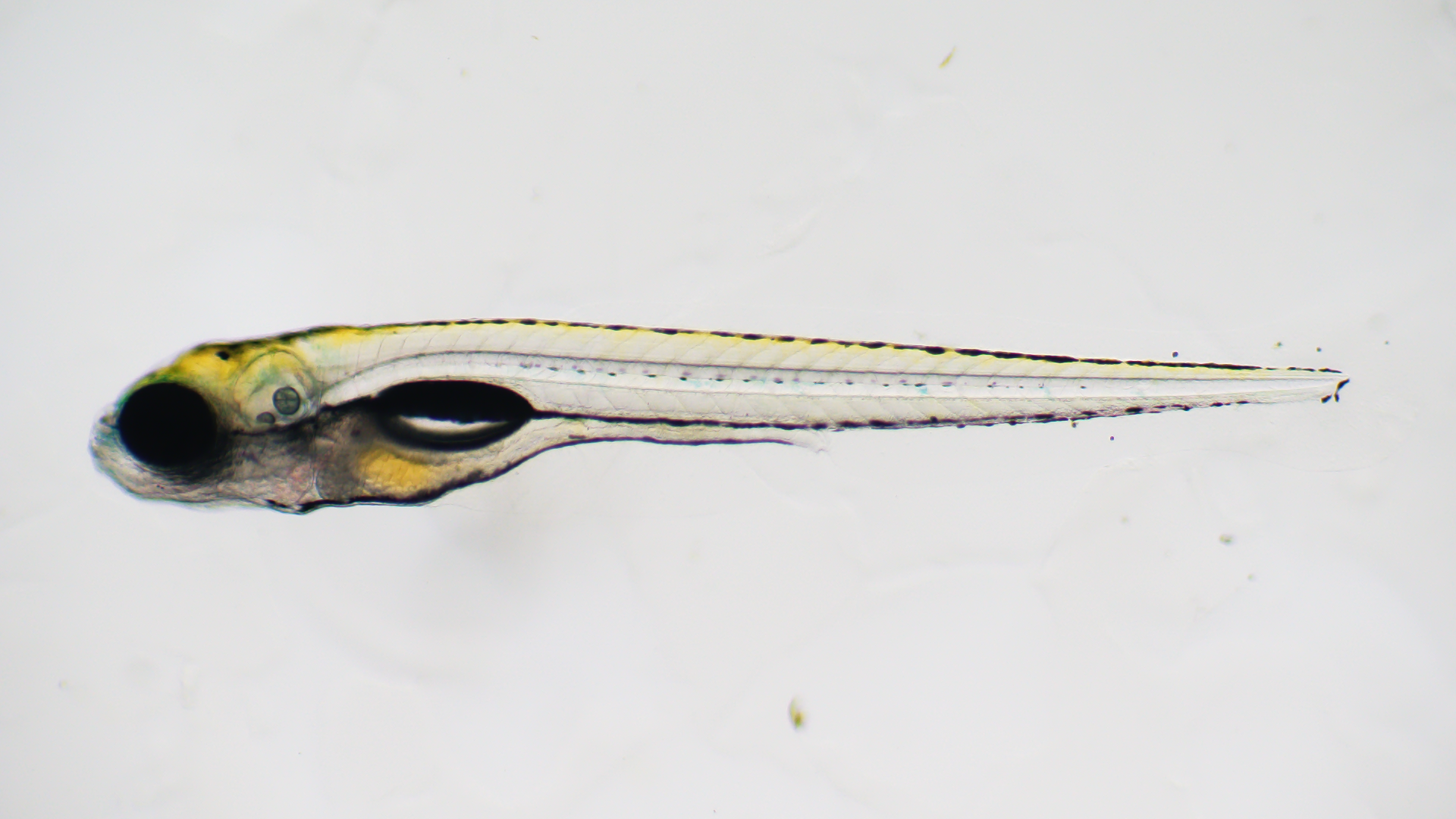

Supplement: Supplementary file 5 — Source data Fig. 4.2 [file 44321_2025_355_MOESM5_ESM.zip › WT_7dpf.tif]

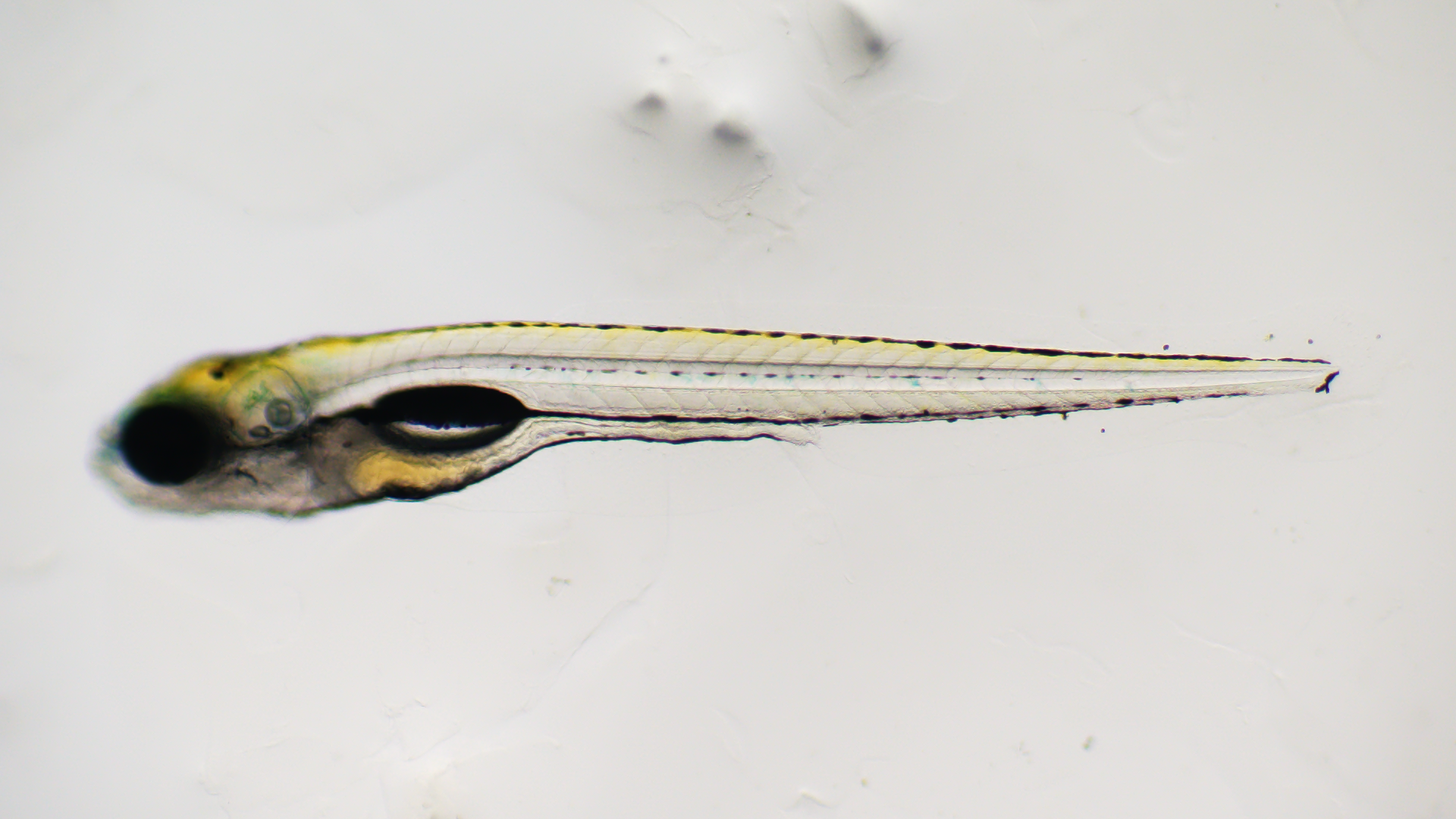

Supplement: Supplementary file 5 — Source data Fig. 4.2 [file 44321_2025_355_MOESM5_ESM.zip › WT_8dpf.tif]

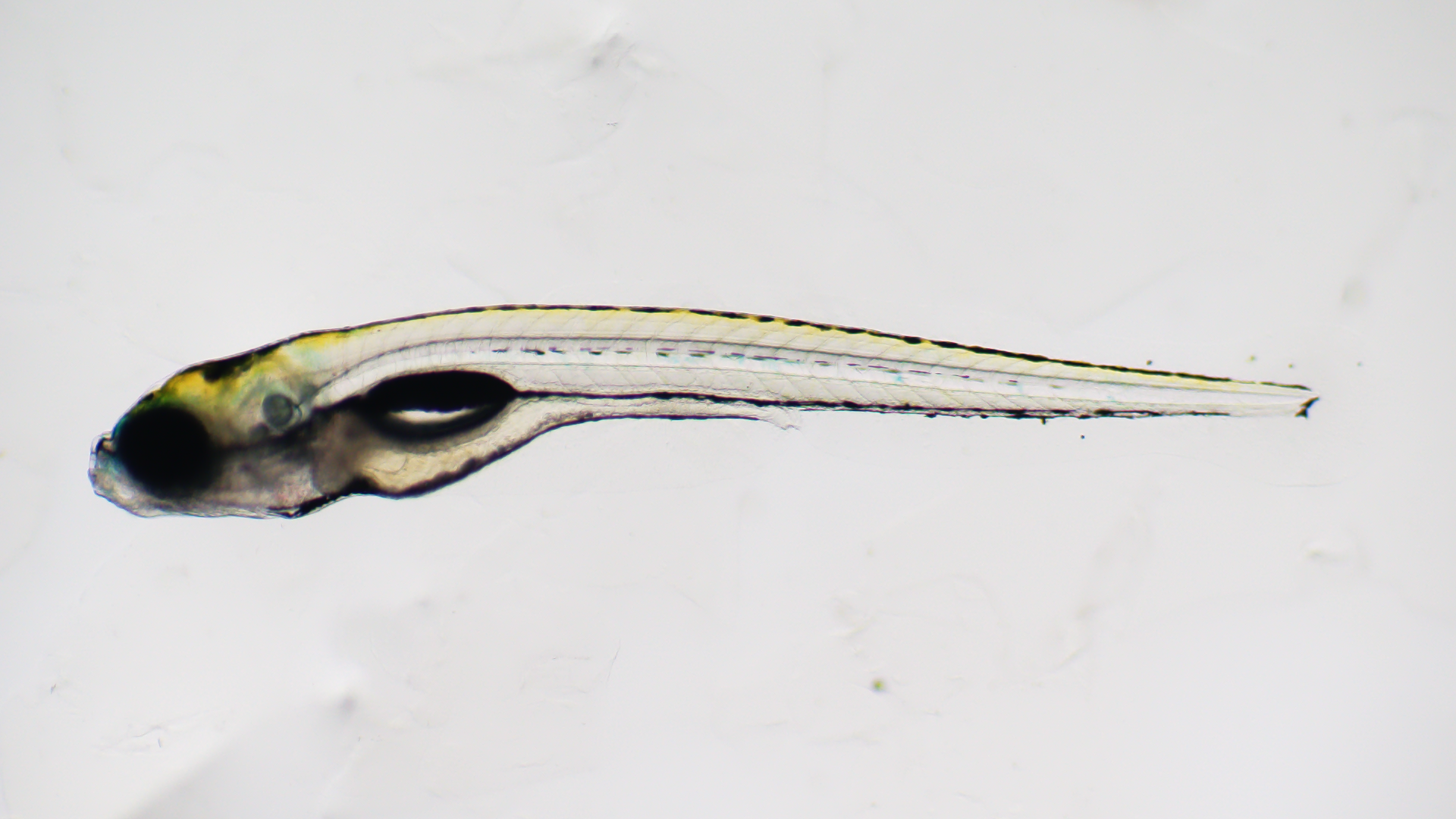

Supplement: Supplementary file 5 — Source data Fig. 4.2 [file 44321_2025_355_MOESM5_ESM.zip › WT_9dpf.tif]

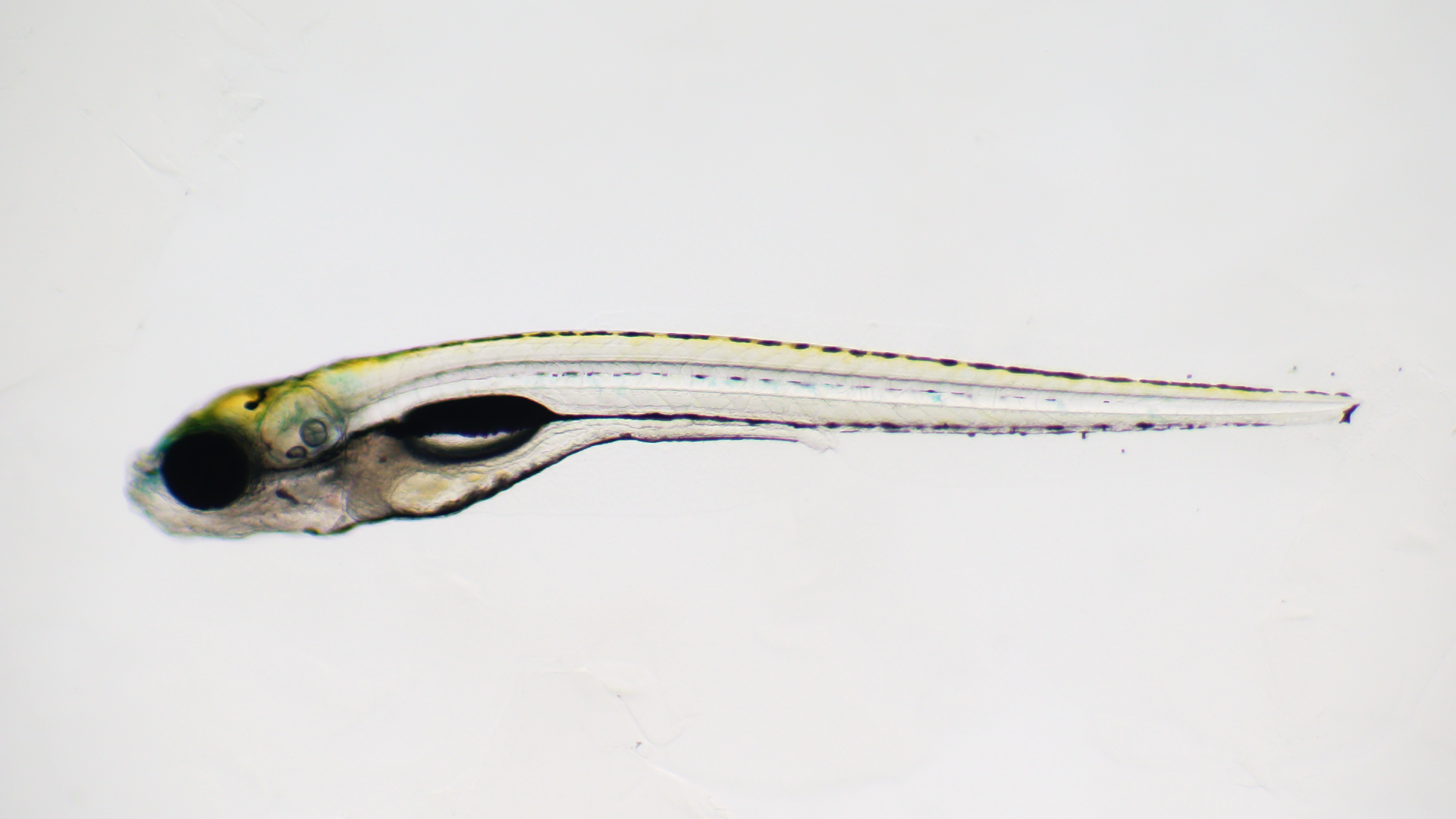

Supplement: Supplementary file 5 — Source data Fig. 4.2 [file 44321_2025_355_MOESM5_ESM.zip › WT_10dpf.tif]

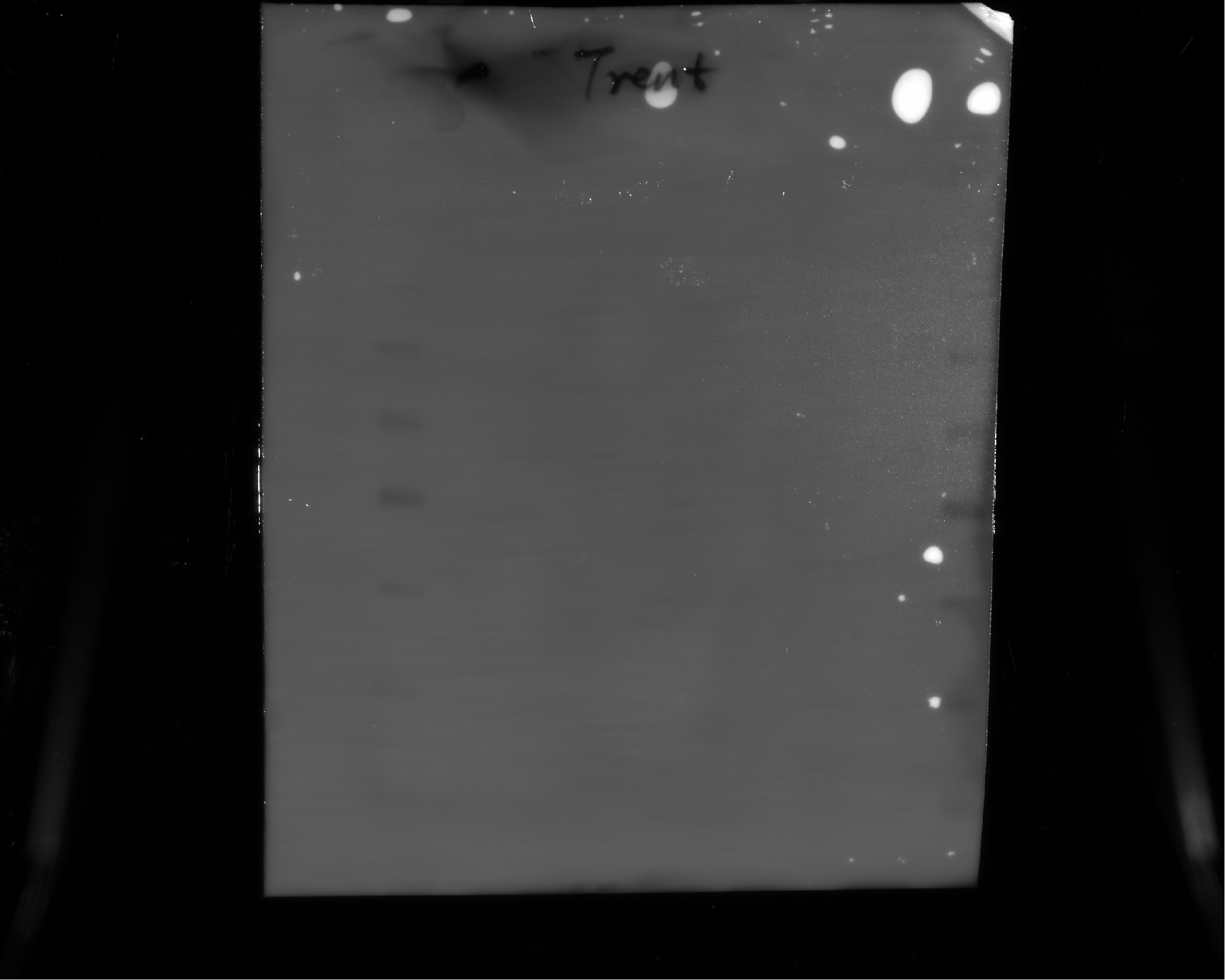

Supplement: Supplementary file 8 — Appendix Figure Source Data [file 44321_2025_355_MOESM8_ESM.zip › Appendix Figures/Appendix Figure S7/S7B/western MW markers.tif]

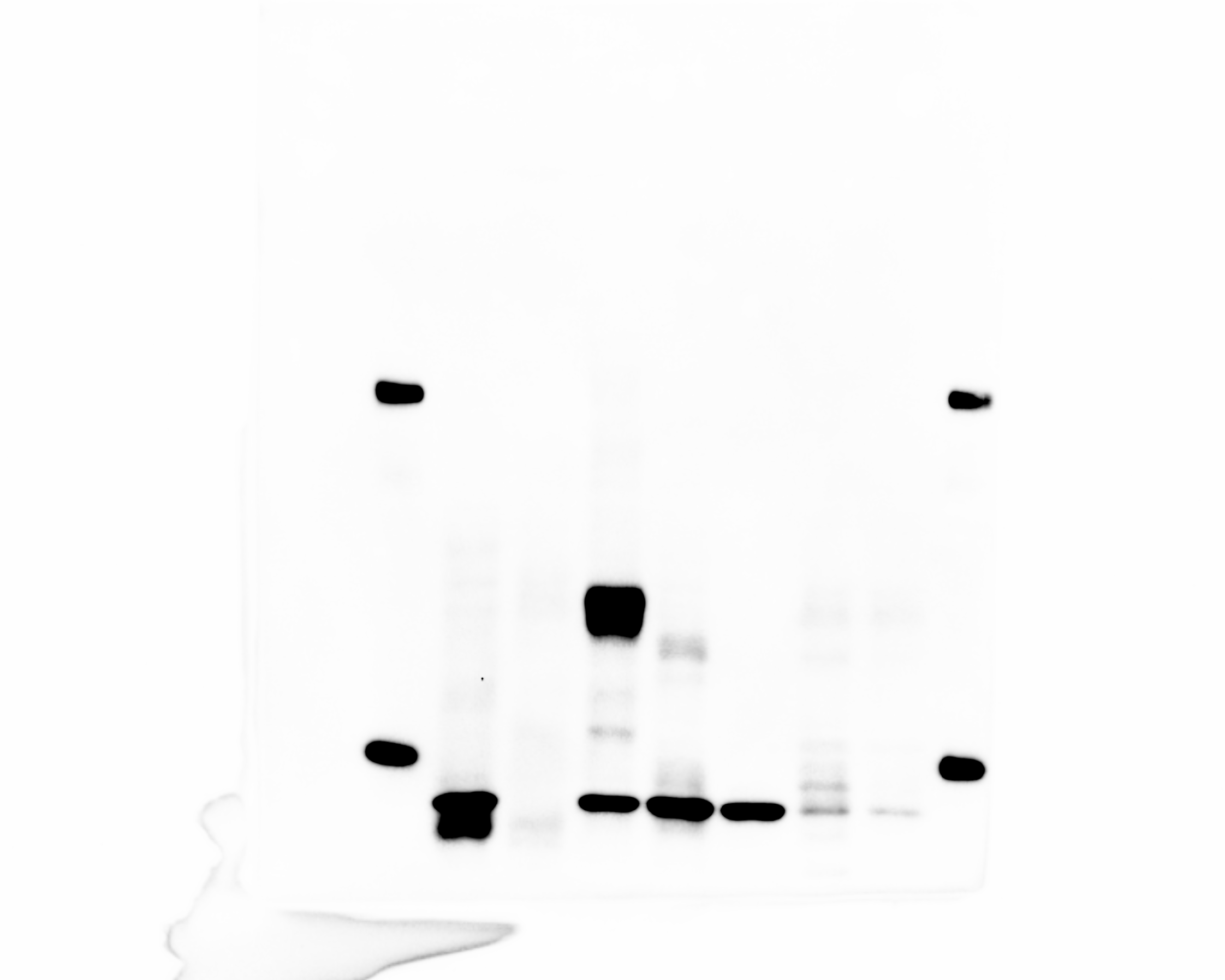

Supplement: Supplementary file 8 — Appendix Figure Source Data [file 44321_2025_355_MOESM8_ESM.zip › Appendix Figures/Appendix Figure S7/S7B/western SMN.tif]

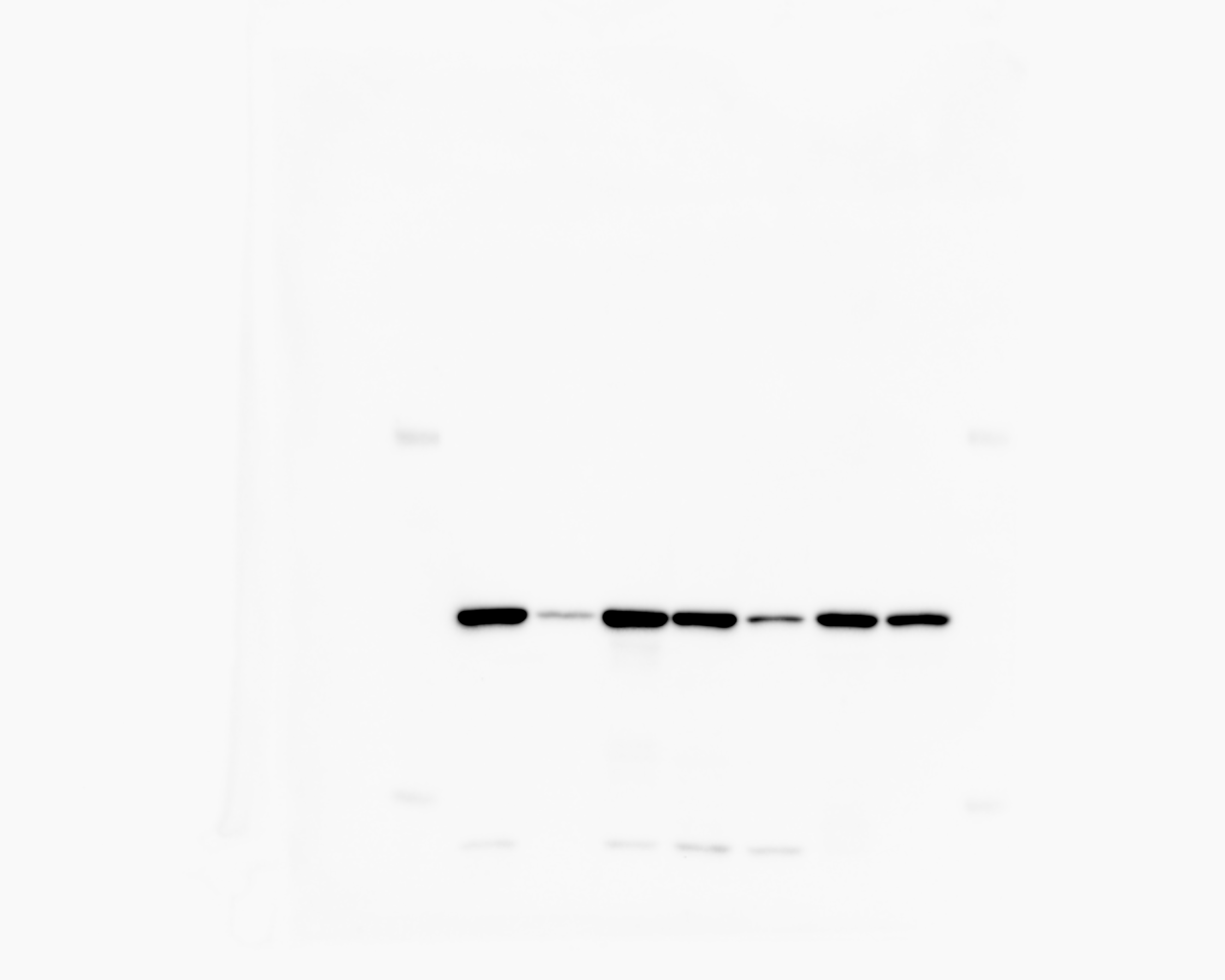

Supplement: Supplementary file 8 — Appendix Figure Source Data [file 44321_2025_355_MOESM8_ESM.zip › Appendix Figures/Appendix Figure S7/S7B/western b-actin.tif]

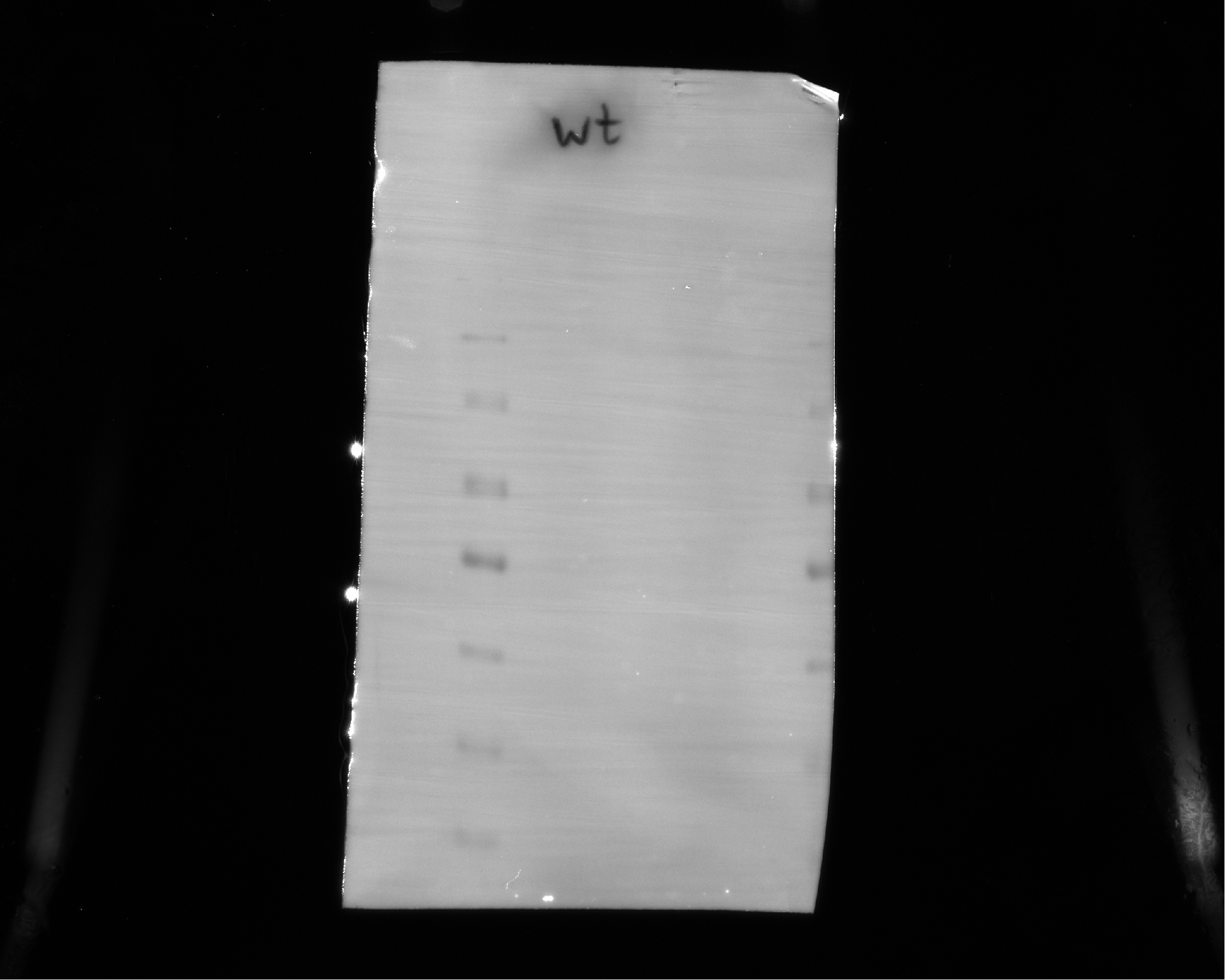

Supplement: Supplementary file 8 — Appendix Figure Source Data [file 44321_2025_355_MOESM8_ESM.zip › Appendix Figures/Appendix Figure S7/S7E/western MW markers.tif]

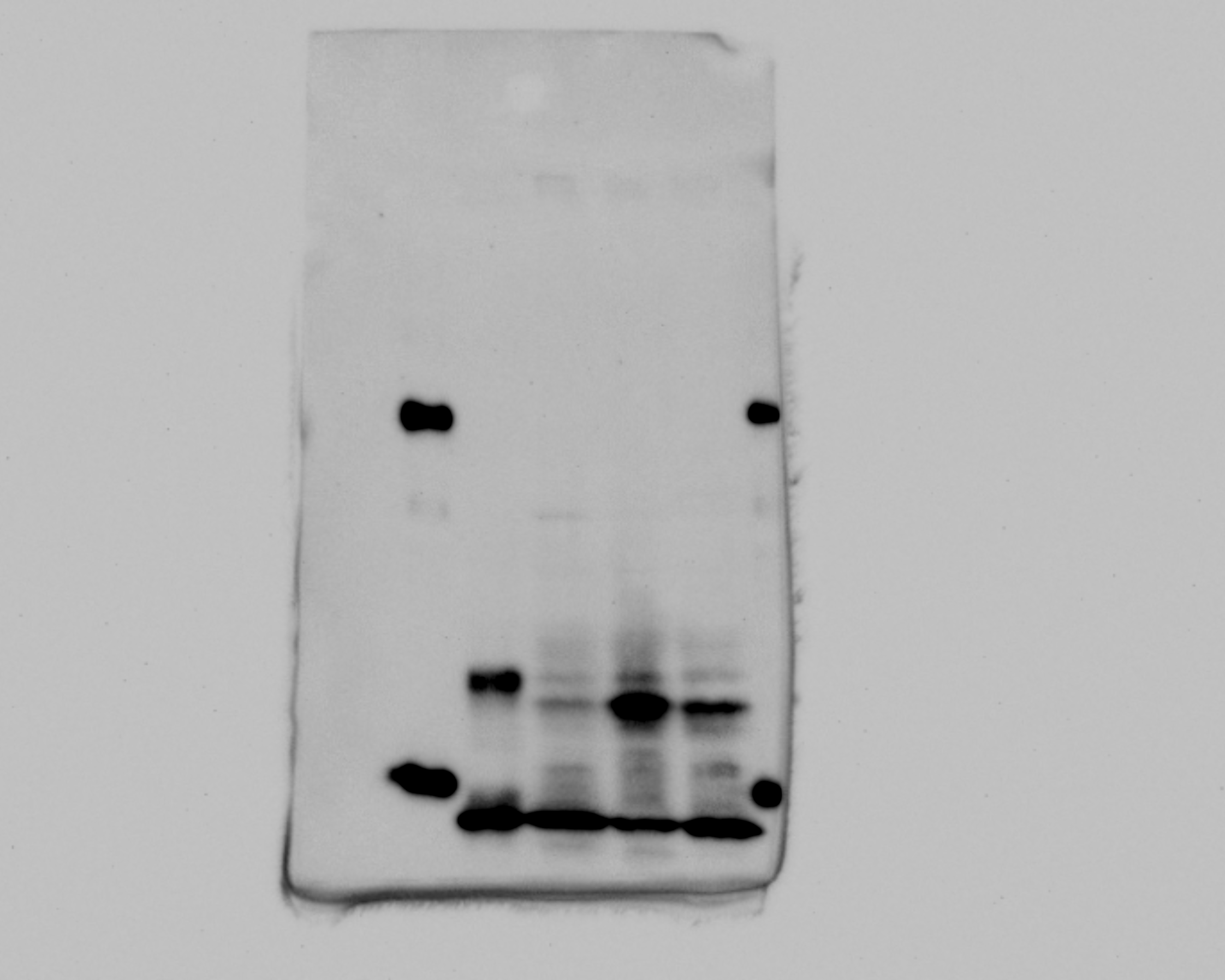

Supplement: Supplementary file 8 — Appendix Figure Source Data [file 44321_2025_355_MOESM8_ESM.zip › Appendix Figures/Appendix Figure S7/S7E/western SMN.tif]

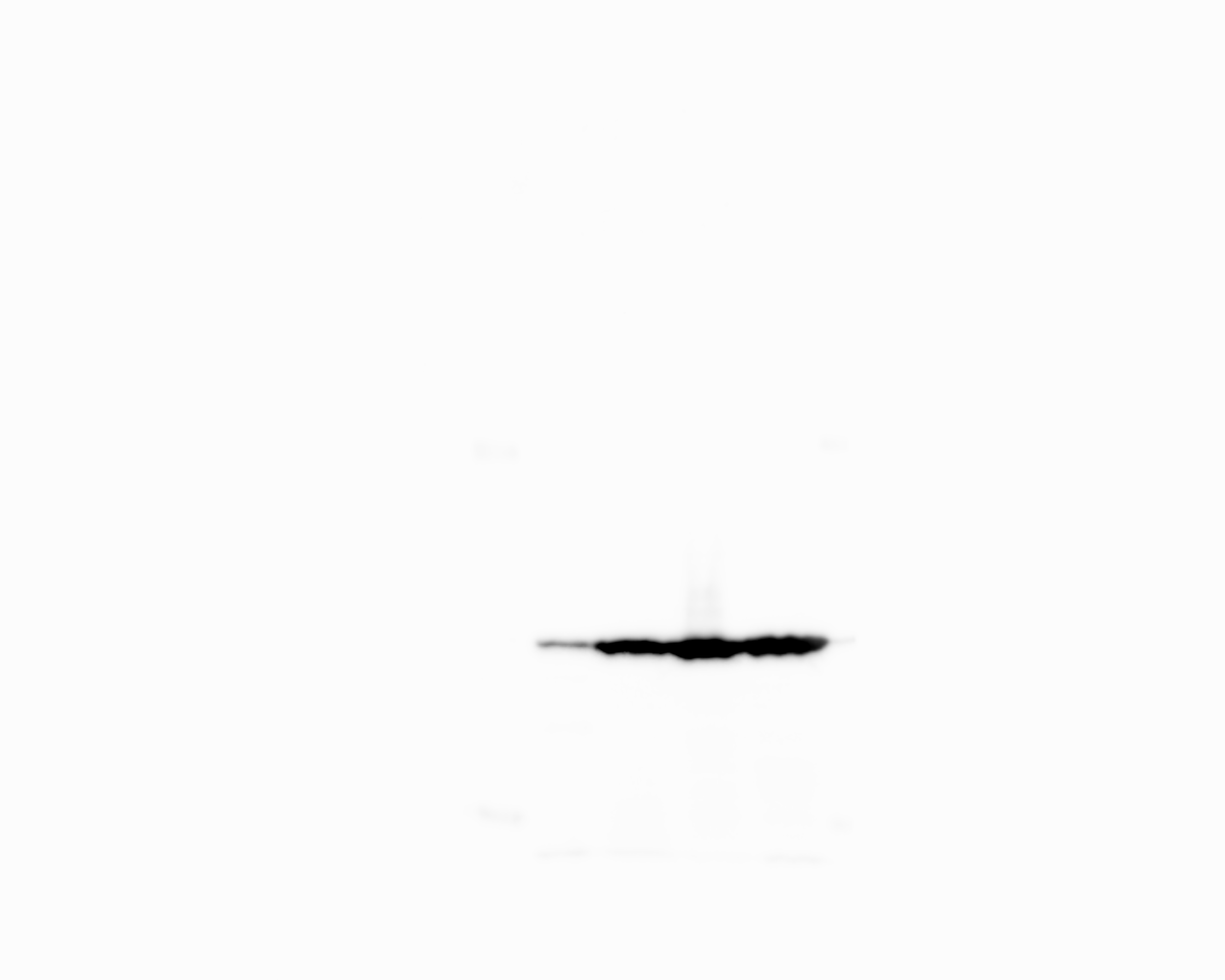

Supplement: Supplementary file 8 — Appendix Figure Source Data [file 44321_2025_355_MOESM8_ESM.zip › Appendix Figures/Appendix Figure S7/S7E/western b-actin.tif]

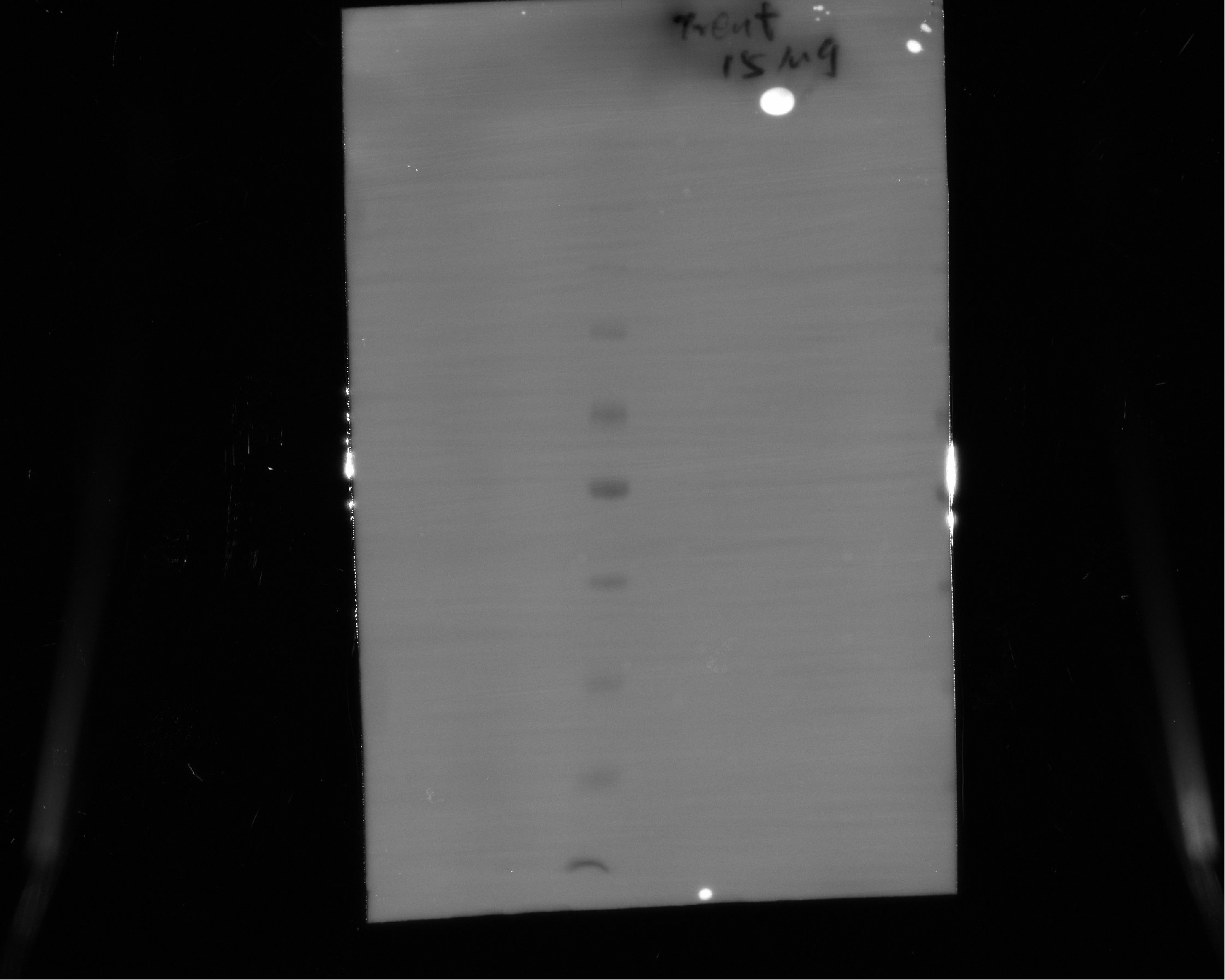

Supplement: Supplementary file 8 — Appendix Figure Source Data [file 44321_2025_355_MOESM8_ESM.zip › Appendix Figures/Appendix Figure S7/S7D/western MW markers.tif]

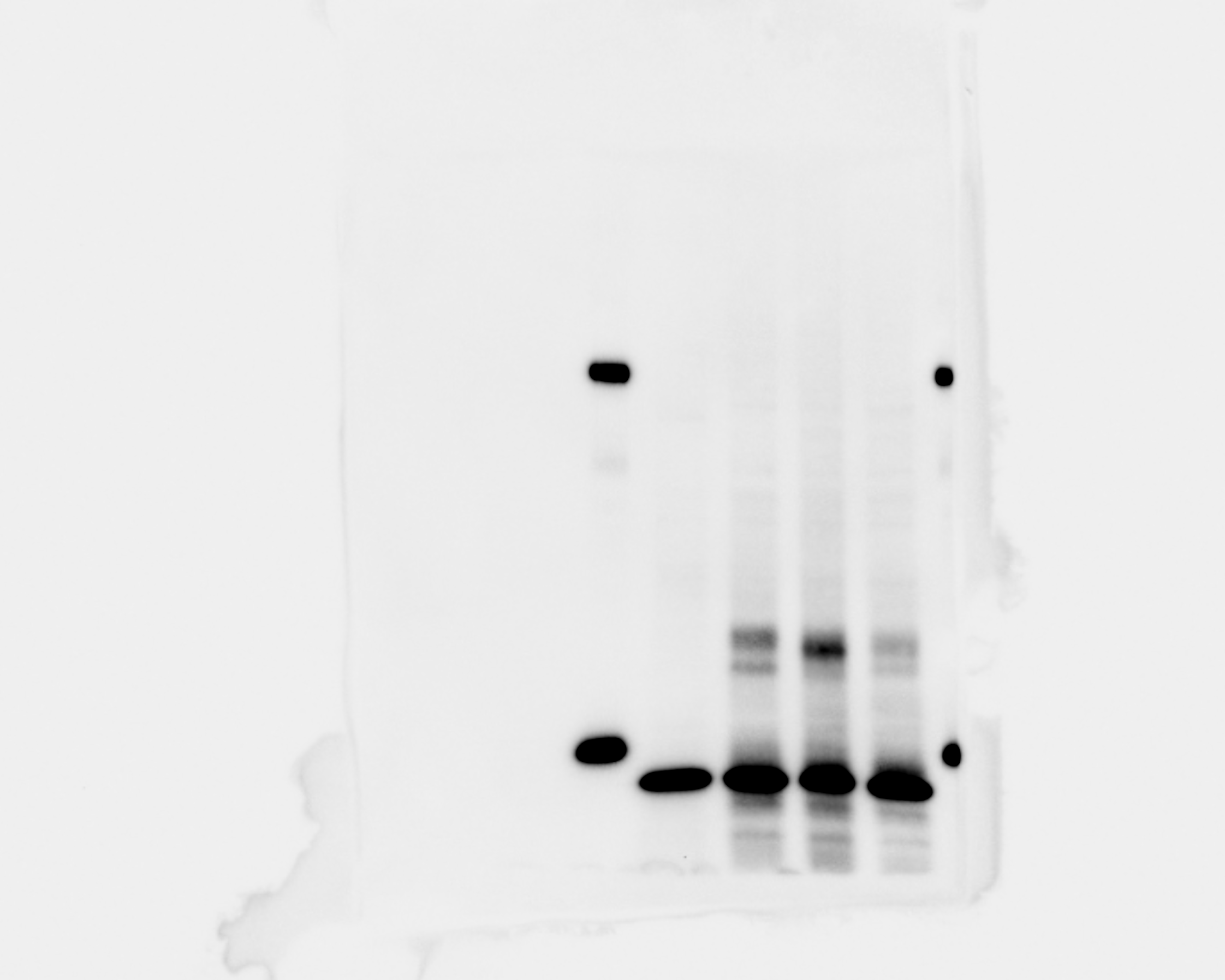

Supplement: Supplementary file 8 — Appendix Figure Source Data [file 44321_2025_355_MOESM8_ESM.zip › Appendix Figures/Appendix Figure S7/S7D/western SMN.tif]

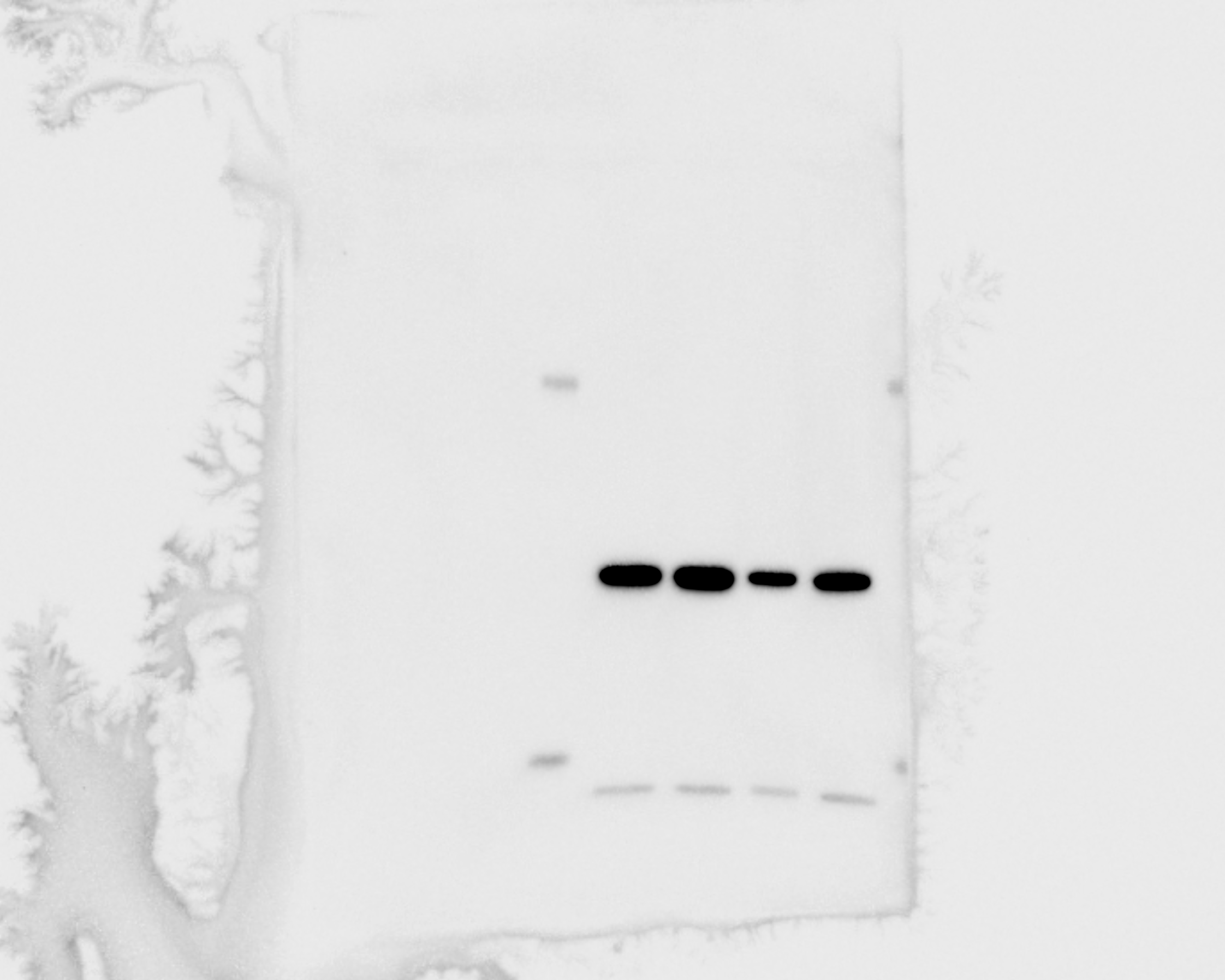

Supplement: Supplementary file 8 — Appendix Figure Source Data [file 44321_2025_355_MOESM8_ESM.zip › Appendix Figures/Appendix Figure S7/S7D/western b-actin.tif]

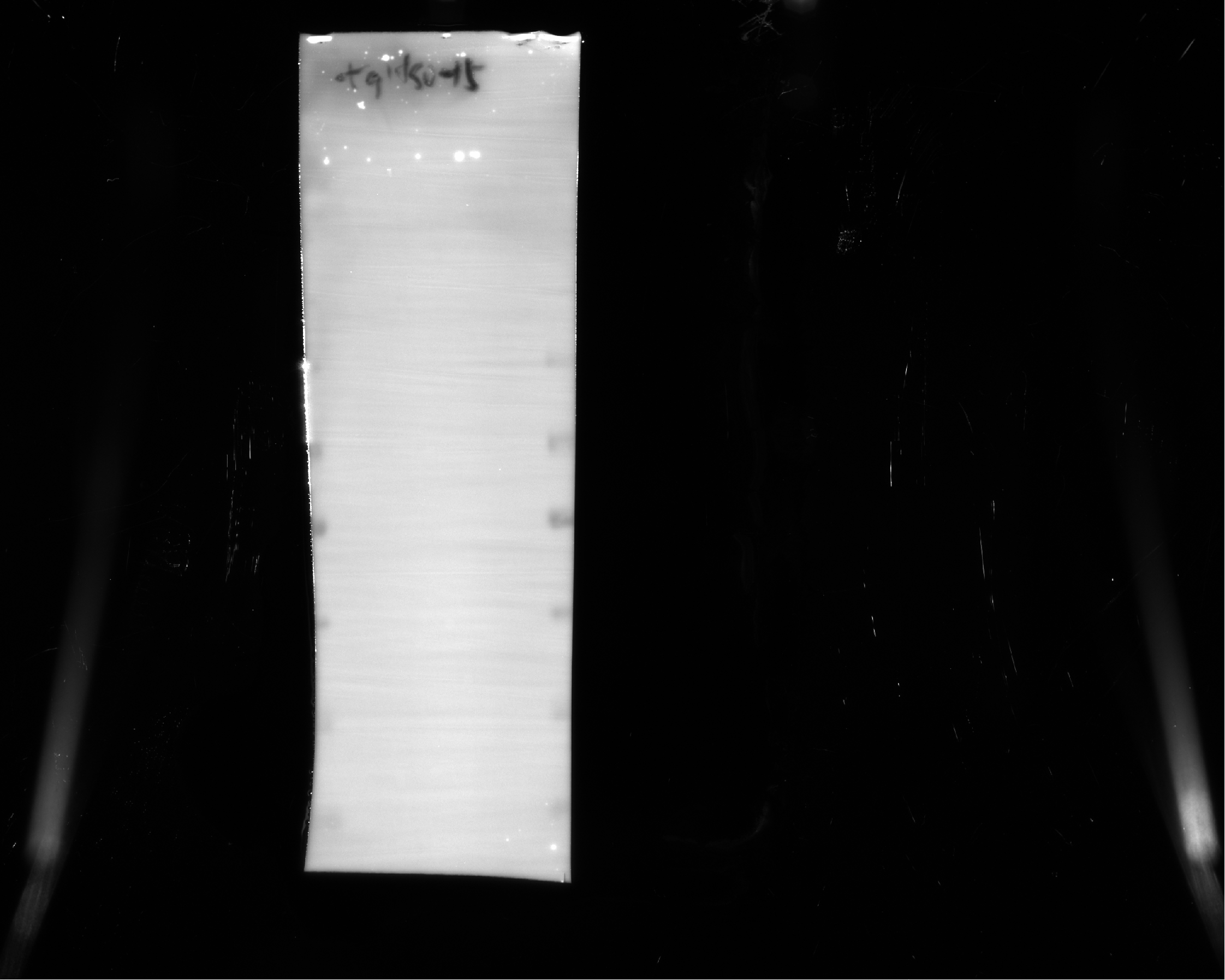

Supplement: Supplementary file 8 — Appendix Figure Source Data [file 44321_2025_355_MOESM8_ESM.zip › Appendix Figures/Appendix Figure S7/S7C/western MW markers.tif]

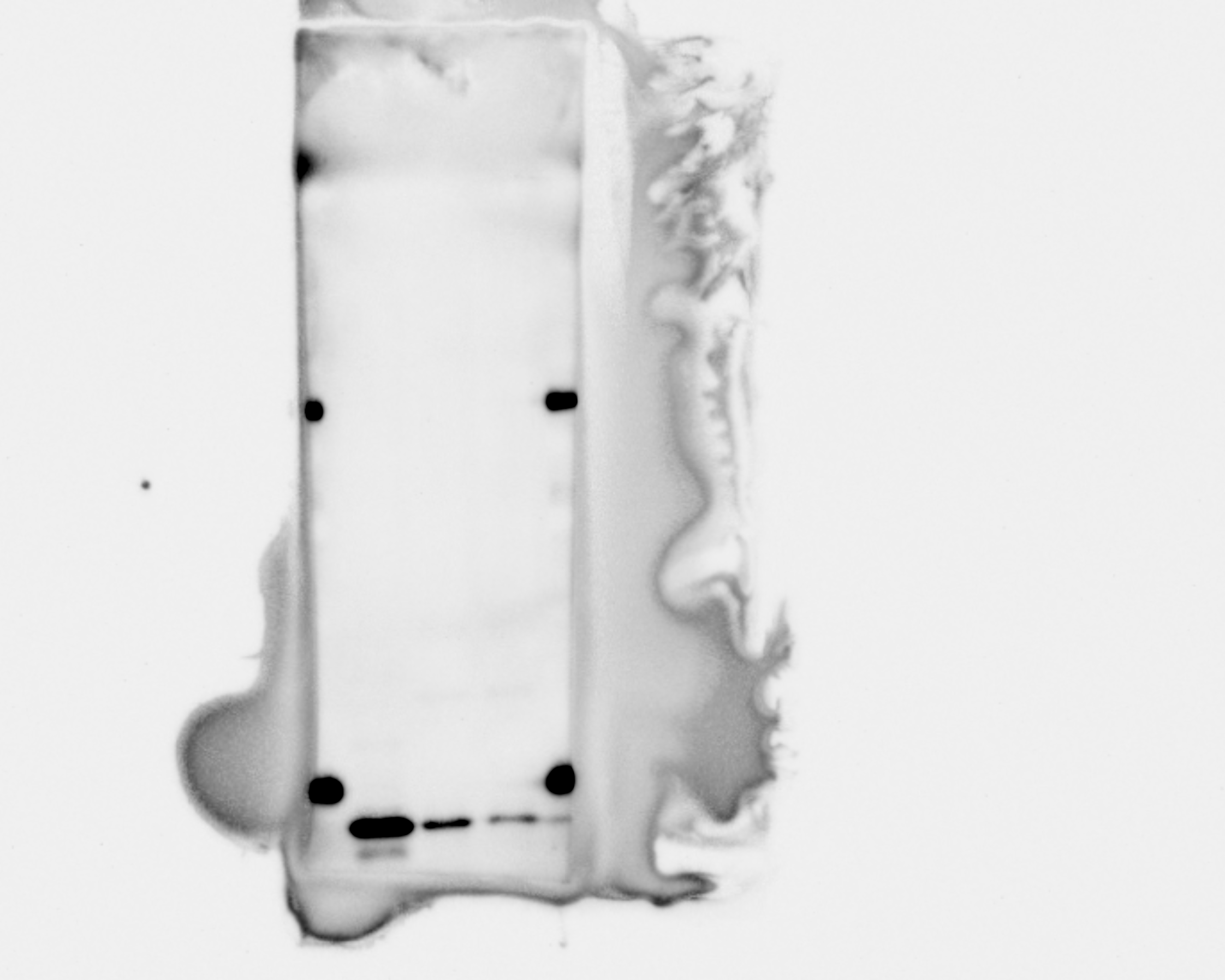

Supplement: Supplementary file 8 — Appendix Figure Source Data [file 44321_2025_355_MOESM8_ESM.zip › Appendix Figures/Appendix Figure S7/S7C/western SMN.tif]

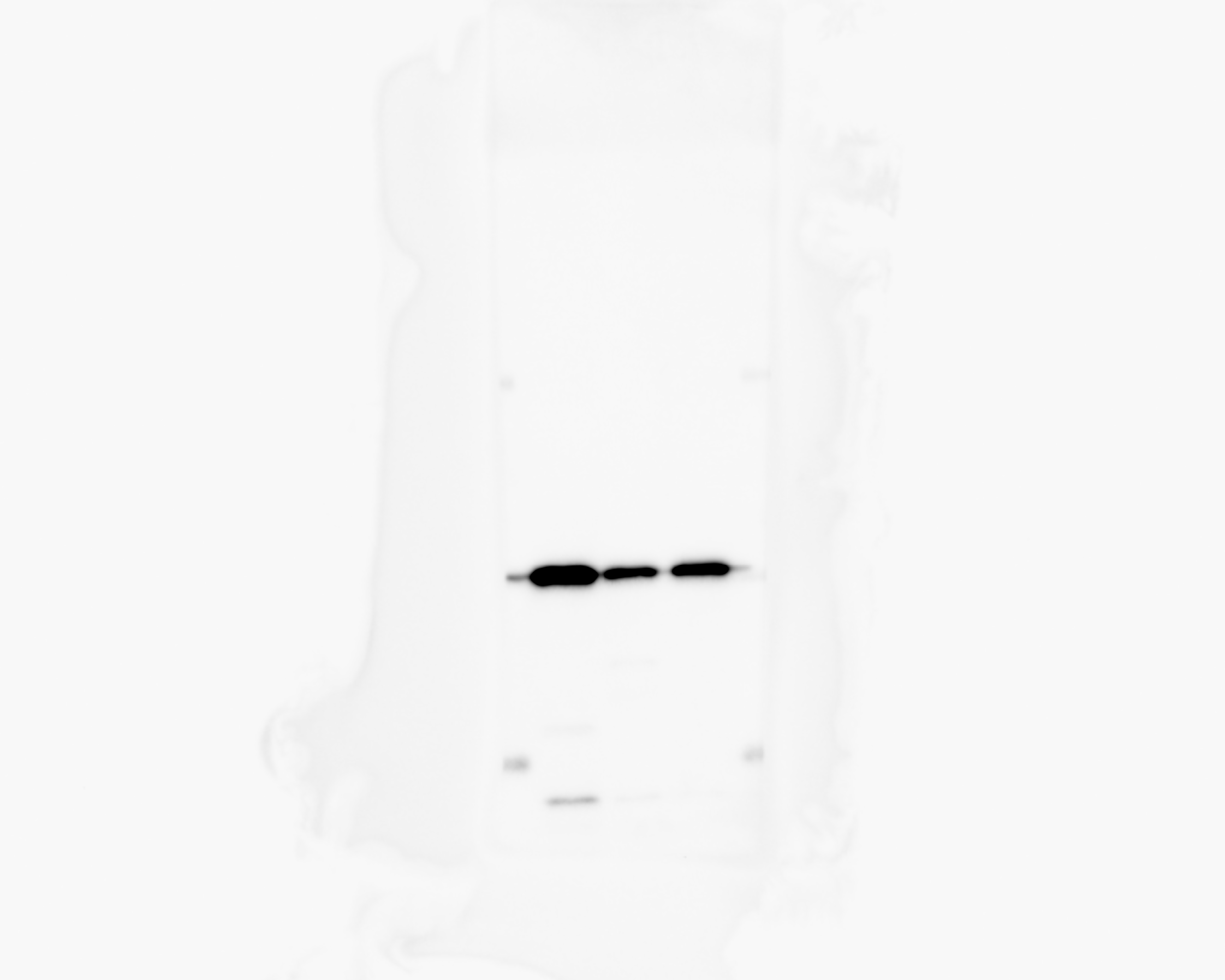

Supplement: Supplementary file 8 — Appendix Figure Source Data [file 44321_2025_355_MOESM8_ESM.zip › Appendix Figures/Appendix Figure S7/S7C/western b-actin.tif]
