# Supplementary material for: Clinical relevance of zebrafish for gene variants testing. Proof-of-principle with SMN1/SMA
Source: EMBO Mol Med. 2025 Dec 15;18(1):41–54. doi: 10.1038/s44321-025-00355-8 (PMC12808650; doi:10.1038/s44321-025-00355-8)
Supplement: Supplementary file 9 — Expanded View Figures [file 44321_2025_355_MOESM9_ESM.pdf]

Expanded View Figures

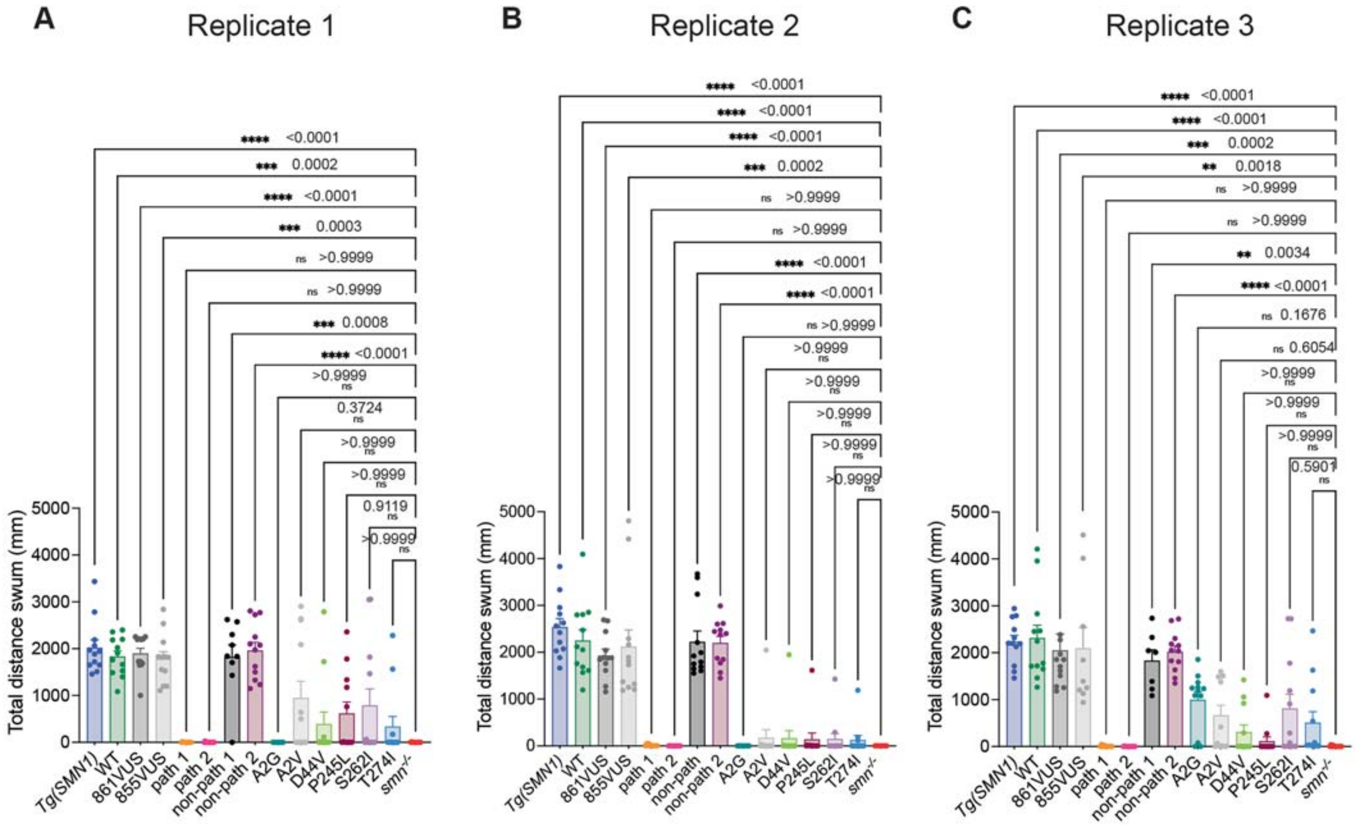

Figure EV1. mRNA injections of *SMN1* variants associated with SMA type II and III do not significantly improve the swimming ability of *smn*$^{-/-}$ zebrafish.

(**A–C**) While *smn*$^{-/-}$ zebrafish swam significantly better at 5 dpf when injected with *SMN1* wt mRNA (WT), non-pathogenic *SMN1* variant mRNA or mRNA from either VUS, those injected with SMA type II- or type III-associated *SMN1* variant mRNA did not. Graphs represent comparisons of the total distance swum by each cohort of fish over 24 min. Data from three replicate experiments are shown. Each data point represents one fish. Error bars represent standard error of the mean. Statistical significance was evaluated using the Kruskal–Wallis test with Dunn's correction for multiple comparisons. ****$P < 0.0001$; ***$P < 0.001$; **$P < 0.01$; ns, not significant. Exact *P* values are also shown. Source data are available online for this figure.

 © The Author(s)
